# Impact of Intensive Handwashing Promotion on Secondary Household Influenza-Like Illness in Rural Bangladesh: Findings from a Randomized Controlled Trial

**DOI:** 10.1371/journal.pone.0125200

**Published:** 2015-06-11

**Authors:** Pavani K. Ram, Margaret A. DiVita, Kaniz Khatun-e-Jannat, Manoshi Islam, Kimberly Krytus, Emily Cercone, Badrul Munir Sohel, Makhdum Ahmed, Abid Mahmud Quaiyum Rahman, Mustafizur Rahman, Jihnhee Yu, W. Abdullah Brooks, Eduardo Azziz-Baumgartner, Alicia M. Fry, Stephen P. Luby

**Affiliations:** 1 Department of Epidemiology and Environmental Health, State University of New York at Buffalo, Buffalo, NY, United States of America; 2 Centre for Communicable Diseases, International Centre for Diarrhoeal Diseases Research, Bangladesh, Dhaka, Bangladesh; 3 Department of Biostatistics, State University of New York at Buffalo, Buffalo, NY, United States of America; 4 Influenza Division, Centers for Disease Control and Prevention, Atlanta, GA, United States of America; Glaxo Smith Kline, DENMARK

## Abstract

**Rationale:**

There is little evidence for the efficacy of handwashing for prevention of influenza transmission in resource-poor settings. We tested the impact of intensive handwashing promotion on household transmission of influenza-like illness and influenza in rural Bangladesh.

**Methods:**

In 2009–10, we identified index case-patients with influenza-like illness (fever with cough or sore throat) who were the only symptomatic person in their household. Household compounds of index case-patients were randomized to control or intervention (soap and daily handwashing promotion). We conducted daily surveillance and collected oropharyngeal specimens. Secondary attack ratios (SAR) were calculated for influenza and ILI in each arm. Among controls, we investigated individual risk factors for ILI among household contacts of index case-patients.

**Results:**

Among 377 index case-patients, the mean number of days between fever onset and study enrollment was 2.1 (SD 1.7) among the 184 controls and 2.6 (SD 2.9) among 193 intervention case-patients. Influenza infection was confirmed in 20% of controls and 12% of intervention index case-patients. The SAR for influenza-like illness among household contacts was 9.5% among intervention (158/1661) and 7.7% among control households (115/1498) (SAR ratio 1.24, 95% CI 0.92–1.65). The SAR ratio for influenza was 2.40 (95% CI 0.68–8.47). In the control arm, susceptible contacts <2 years old (RR_adj_ 5.51, 95% CI 3.43–8.85), those living with an index case-patient enrolled ≤24 hours after symptom onset (RR_adj_ 1.91, 95% CI 1.18–3.10), and those who reported multiple daily interactions with the index case-patient (RR_adj_ 1.94, 95% CI 1.71–3.26) were at increased risk of influenza-like illness.

**Discussion:**

Handwashing promotion initiated after illness onset in a household member did not protect against influenza-like illness or influenza. Behavior may not have changed rapidly enough to curb transmission between household members. A reactive approach to reduce household influenza transmission through handwashing promotion may be ineffective in the context of rural Bangladesh.

**Trial Registration:**

ClinicalTrials.gov NCT00880659

## Introduction

Seasonal influenza has been increasingly recognized as an important cause of acute respiratory infection globally, estimated to cause 90 million new cases worldwide among young children in 2008,[1] and 19,244 disability-adjusted life years lost across all age groups[2]. The estimated risk of influenza is 3,500 episodes / 100,000 child-years among children living in low- and middle-income countries, three-fold higher than seen among children in high-income countries.[1]

Among all of the contacts of a person infected with influenza virus in and out of the home, household members are likely to be at highest risk of exposure to the virus. In the United States, 13% of household contacts of patients with confirmed and probable influenza A(H1N1)pdm09 infection (2009 H1N1) developed respiratory symptoms during the 7 days following the onset of influenza symptoms in the index case-patient.[3] In urban Bangladesh, household crowding is associated with respiratory infection in young children[4]. In places like Bangladesh, where the population density is one of the highest in the world (1049 persons per square kilometer), and homes are often crowded and poorly ventilated [5], respiratory pathogen transmission to household contacts may be much higher than observed in high-income settings.

High-income countries rely on a multi-pronged approach to seasonal and pandemic influenza prevention and control that includes vaccination, early use of antiviral drugs, and promotion of personal hygiene behaviors such as handwashing. The speed of progression of the 2009 H1N1 influenza pandemic to low- and middle-income countries worldwide clarified the importance of identifying strategies that can be applied quickly and effectively to prevent influenza virus transmission. Handwashing and other non-pharmaceutical interventions to prevent influenza transmission are recommended by the US Centers for Disease Control and Prevention as adjuncts to vaccination (http://www.cdc.gov/flu/protect/preventing.htm). Given other burdens on their health systems, and the need to focus on other routine health concerns, most low-income countries like Bangladesh do not prioritize the use of pharmaceutical interventions, such as vaccination and antiviral therapy, to prevent influenza. Thus, non-pharmaceutical interventions, especially handwashing promotion, necessarily become a principal strategy for influenza prevention.

However, existing data on the efficacy of non-pharmaceutical interventions for prevention of influenza transmission to household contacts comes largely from settings that are typically wealthier and less crowded than Bangladesh[6–9]; annually, approximately 10% of the population in Bangladesh seeks care and 130,000 persons are hospitalized as a result of influenza illnesses each year[10]. In Bangladesh, handwashing with soap is uncommon [11], particularly after respiratory secretion contact [12], and thus, the risk of transmission via direct contact has the potential to be high [13,14]. The lack of data on the impact of handwashing promotion on household influenza transmission from low-income settings reflects a substantial gap in our understanding of how to minimize the effects of epidemic and pandemic influenza among the most vulnerable populations. Therefore, in rural Bangladesh, we conducted a randomized controlled trial to examine whether intensive handwashing promotion upon identification of influenza or influenza-like illness in an index case-patient decreased transmission to susceptible household contacts. We also investigated risk factors for transmission of influenza and influenza-like illness among household contacts of index case-patients in the control arm.

## Materials and Methods

We conducted this trial in Kishoregonj, a rural area in Bangladesh served by district health centers, numerous pharmacies, and a tertiary care hospital with a catchment population of 407,276 persons [10]. There are annual influenza peaks between May and September; in a dedicated influenza surveillance activity conducted for two days each month in the tertiary hospital during May-September 2008, 24 (14%) of 167 samples tested from patients with severe acute respiratory infection were found to have PCR-confirmed influenza.[15]

### Participating health facilities and index case-patient data collection

We recruited index case-patients in three phases in 2009 and 2010, with data collection beginning each year after the confirmation of influenza in patients visiting the tertiary care hospital; in each year, we ended recruitment after influenza was no longer being detected. In 2009 and 2010, we screened patients who sought outpatient care for respiratory symptoms at the Jahurul Islam Medical College Hospital (JIMCH), a non-government tertiary care hospital providing both outpatient and inpatient services to the population of Kishoregonj district. In 2010, in an effort to increase recruitment and meet the targeted sample size, study physicians also screened patients seeking outpatient care for respiratory symptoms at two government-operated district health centers, and six local pharmacies. The study physician completed a screening checklist to evaluate whether the patient’s symptoms were consistent with eligibility criteria described below. Upon obtaining written informed consent from the index case-patient or his/her adult guardian, a study physician (KKJ, MI, MA, or AMQR) obtained an oropharyngeal swab from each index case-patient for influenza testing.

The eligibility criteria used for index case-patients in each phase are specified in detail in S1 Text and S1 Table. In brief, in 2009, we recruited index case-patients with symptom onset within 7 days preceding enrollment. After the influenza season in Bangladesh concluded in 2009, we became aware of findings from Cowling and colleagues[8], in which hand hygiene promotion was effective at preventing household influenza transmission only in the subgroup in which the index case-patient’s symptom onset was within 36 hours of enrollment. Given those findings, we used more restrictive eligibility criteria in 2010, with index case-patient symptom onset within 48 hours preceding enrollment. We used age-specific definitions of influenza-like illness (ILI). For persons ≥ 5 years old, ILI was defined as history of fever with cough *or* sore throat. For children < 5 years old, ILI was defined as fever; we used this relatively liberal case definition in order to include influenza cases with atypical presentations in children. After obtaining written informed consent, we obtained oropharyngeal swabs from index case-patients for laboratory testing for influenza.

A data collector accompanied the index case-patient (and guardian) to his/her household compound. Typically, in rural Bangladesh, a compound encompasses several households occupied by joint or extended families, with a common courtyard, and often, shared latrine, water source, and cooking facilities; occasionally, families that are not related also live in such a set-up. The study was explained to the head of the compound as well as all compound members present. The head of the compound provided written informed consent for enrollment of the compound members and if they so wished, individual households or individual members within the compound could refuse to take part in the study.

Upon obtaining informed consent, the data collector enumerated all members of the index case-patient household, and each secondary household within the compound. For each compound member, we recorded whether the person typically slept in the same room as the index case-patient. We considered contacts that did not have fever within the 7 days preceding enrollment as “susceptible”.

### Randomization

We used a block randomization, with a block size of four, in order to promote random and even allocation of household compounds to the two treatment arms. The list of random assignments was generated by an investigator with no contact with the human subjects. Once baseline data collection was complete, the data collector notified the field research officer, who consulted the block randomization list to make the assignment of the household compound to intervention (intensive handwashing promotion) or control (standard practices). Given the provision of a handwashing station as part of the intervention, it was not possible to ensure blinding of participants, intervention staff, or data collectors.

### Intervention

We initiated intervention activities within 18 hours after enrollment, and continued daily intervention visits until 10 days following the resolution of the index case-patient’s symptoms. All members of the Intervention compound, including adults and children of both sexes, were invited to take part in each intervention session. The intervention was designed following constructs of Social Cognitive Theory and the Health Belief Model.[16] Behavior change communication was developed utilizing social marketing concepts. Elements of the intervention included didactic and interactive group-level education and skills training with compound members, through which we described influenza symptoms, transmission, and prevention; promoted the health- and non-health benefits of handwashing with soap to participants; and identified barriers and proposed solutions to handwashing with soap. Also, on the first day, the staff set up a handwashing station in a central location using the following materials: a large water container with a tap, a plastic case for soap, and a bar of soap. Each day, the intervention staff weighed the soap, and replaced it if the bar weighed ≤20 grams. After checking for the presence of water in the container, s/he also re-supplied water in the container daily as needed. We posted cue cards in a common area in the courtyard depicting critical times to wash hands with soap: after coughing or sneezing, after cleaning one’s nose or a child’s nose, after defecation, after cleaning a child who has defecated, before preparing or serving food, and before eating. During intervention visits, compound residents were often asked to demonstrate handwashing with soap, both to reinforce the skill, as well as to model the behavior for each other.

### Surveillance for secondary transmission, and testing susceptible contacts with influenza-like illness

A data collector visited the compound each day until the 10^th^ day following the resolution of the index case-patient’s symptoms. For each contact (member of the household), the chief respondent (typically, the female head of household) was asked whether the contact had been present in the compound during the previous 24 hours and, if so, whether the contact had fever, cough, or sore throat. If a contact met the age-specific case-definition for influenza-like illness, the data collector phoned the medical officer to alert to the need for specimen collection for influenza testing. Study staff requested consent for specimen collection and testing from the susceptible contact meeting the case definition for influenza-like illness. When consent was obtained, then the medical officer obtained an oropharyngeal swab for testing for influenza.

### Laboratory methods

All swabs were inserted into viral transport media vials and placed on ice packs in a cool box. At the end of each working day, all vials collected on that day were placed into the liquid nitrogen chamber, which was transported weekly to the Virology Laboratory at icddr,b, in Dhaka, where they were tested by polymerase chain reaction (PCR) for influenza A and B, with further subtyping of influenza A isolates.[17]

### Ethical review

Index case-patients, or their guardians, and heads of household provided written informed consent for participation in this study. We obtained individual-level informed consent for all specimen collection.

The protocol for this investigation was reviewed and approved by the Research and Ethical Review Committees of the icddr,b. The trial was registered at www.clinicaltrials.gov (NCT00880659, http://clinicaltrials.gov/ct2/show/NCT00880659?term=NCT00880659&rank=1). The protocol for this trial and supporting CONSORT checklist are available as supporting information; see S1 Protocol and S1 Checklist.

### Sample size estimation

We performed sample size estimations to model the impact of intensive handwashing promotion on acquisition of influenza. We assumed 10 contacts per household (excluding the index case-patient). In previous studies, the secondary attack ratio of respiratory illness or influenza ranged from 8% to 17% among household contacts, with the 8% secondary attack ratio for influenza detected in a pilot study in Hong Kong of non-pharmaceutical interventions.[18–20] Since we did not already have secondary attack ratio data from Bangladesh, we performed sample size calculations based on estimates of 30%, 20%, and 10% SAR in the control group, and relative risk reductions of 50%. Sample sizes were estimated at the 95% confidence level to achieve 80% power. We introduced a design effect of 2.0 to account for clustering of secondary cases within compounds, and increased the estimated sample size by approximately 25% to allow for missing data and withdrawals, yielding a calculated sample size of 200 household compounds in total, 100 in each arm.[21,22] Since the influenza positivity rate was low in 2009, and additional funds became available, we ran the trial in 2010 as well in an effort to maximize the number of influenza-positive cases. Thus, the number of index case-patients overall was higher than the calculated sample size.

### Analytic approach

We describe the effects of the intervention on two outcome measures of interest among susceptible contacts: influenza-like illness and laboratory-confirmed influenza infection.

First, we performed an intent-to-treat analysis to investigate the effects of randomization to the intervention arm on secondary transmission of influenza-like illness. We calculated the secondary attack risk (SAR) for each arm by dividing the number of susceptible contacts identified with influenza-like illness by the total number of susceptible contacts under surveillance. We calculated the SAR ratio of the intervention arm compared to control (SAR_intervention_ / SAR_control_), using log binomial regression and accounting for clustering at the household and compound levels. In multivariable analysis, we estimated the adjusted SAR ratio by including variables that differed between intervention and control arms at baseline; specifically, we tested the effects of categorical variables for which there was a 10 percentage point difference between the two groups, or continuous variables for which there was a relatively 10% difference between the two groups. Since influenza infection was of particular interest, we also tested its effect on the SAR ratio for influenza-like illness.

Among contacts of index case-patients with confirmed influenza infection, we modeled the SAR ratio of laboratory-confirmed influenza among susceptible members in the intervention arm compared to control. Next, we performed similar analyses among only those contacts residing in the same household as the index case-patient, since residents of the index case-patient household may have been more susceptible due to proximity. Given the evolution of our eligibility criteria for index case-patients and household compounds (S1 Table), we performed restricted analyses using data from compounds enrolled in 2010, as well as those enrolled in 2009 with index case-patient symptom onset within 48 hours preceding enrollment.

To examine patterns of soap use during the intervention period, we subtracted each day’s soap weight from the weight recorded on the previous day to reflect the soap consumed during the intervening period. We identified the maximum number of grams of soap consumed for each compound and identified the day on which the maximum soap consumption was recorded. We divided the number of grams of soap consumed by the number of persons living in the compound to develop a per capita estimate of daily soap consumption.

To describe the relationship between individual-level risk factors and influenza-like illness (or confirmed influenza), we analyzed data from susceptible contacts in the control arm only. In addition to demographic characteristics and smoking status of the index case-patient and susceptible contact, we also evaluated the following potential risk factors: index case-patient cough during illness, duration from fever onset to enrollment, and number of minutes that each susceptible contact typically spends in the cooking space. We described the association between frequent exposure to the index case-patient and influenza-like illness (or confirmed influenza) by the following: relationship to index case-patient, residence in the index case-patient household, sleeping in the same room as the index case-patient, and reported multiple interactions daily with the index case-patient. Information on whether the contact shared the index case-patient’s sleeping space was collected only among residents of the index case-patient household. Information on frequency of interactions with the index case-patient was collected only among 2010 participants. We performed bivariate analyses using log binomial regressions, accounting for possible clustering at the household and compound levels. We tested all variables associated with the outcome at p<.20 in bivariate analysis in multivariable models. For multivariable analyses, we again used log binomial regressions, and entered variables manually, each time retaining the exposure variables that were independently and significantly associated with the outcome, and dropping the variables not associated with the outcome (defined as p<.05).

We used SAS v.9 (Cary, NC) for all analyses.

## Results

**Fig 1:**
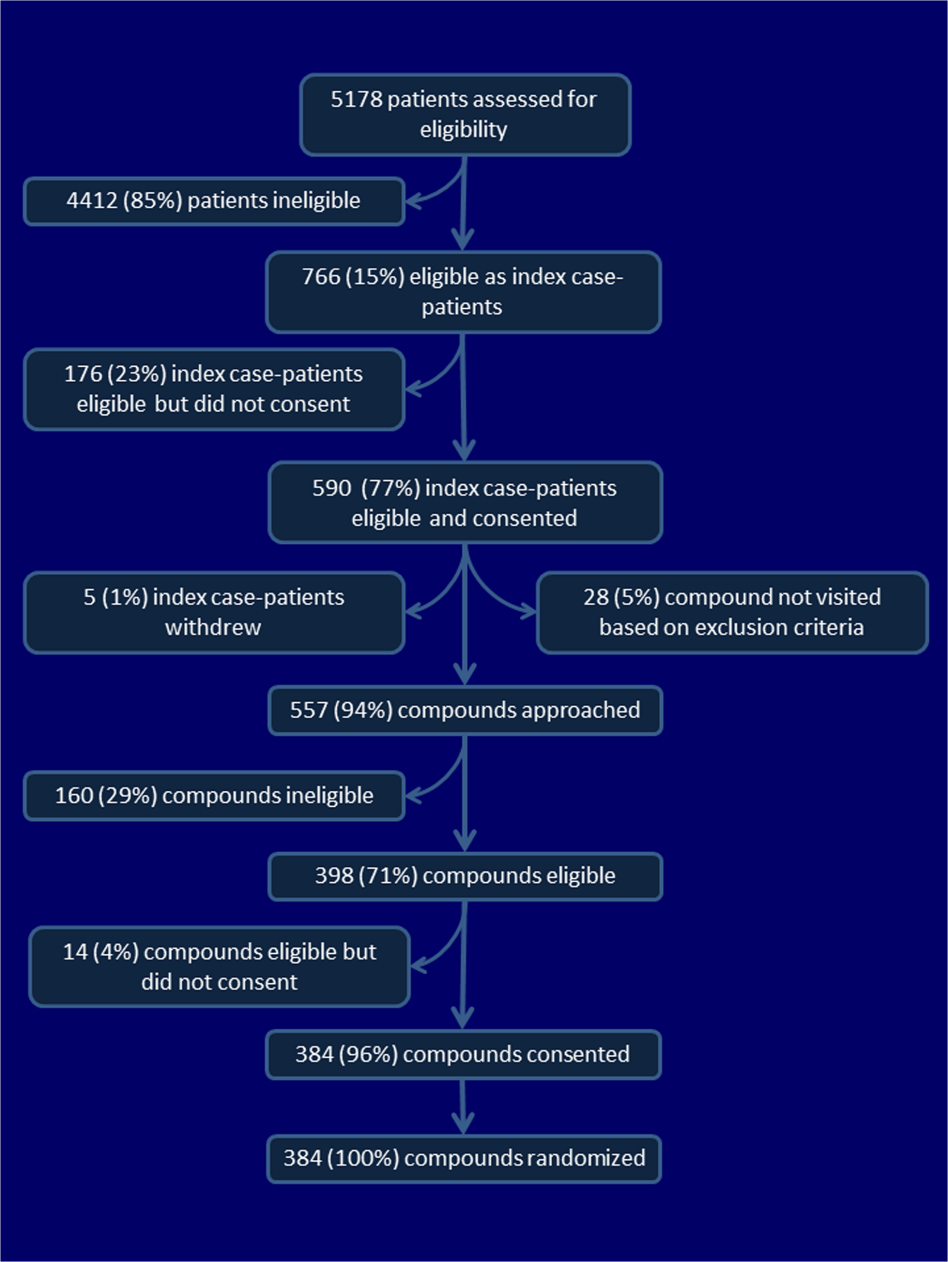
Flow diagram to describe screening, inclusion, exclusion, and randomization, Kishoregonj, Bangladesh, 2009–2010.

**Fig 2:**
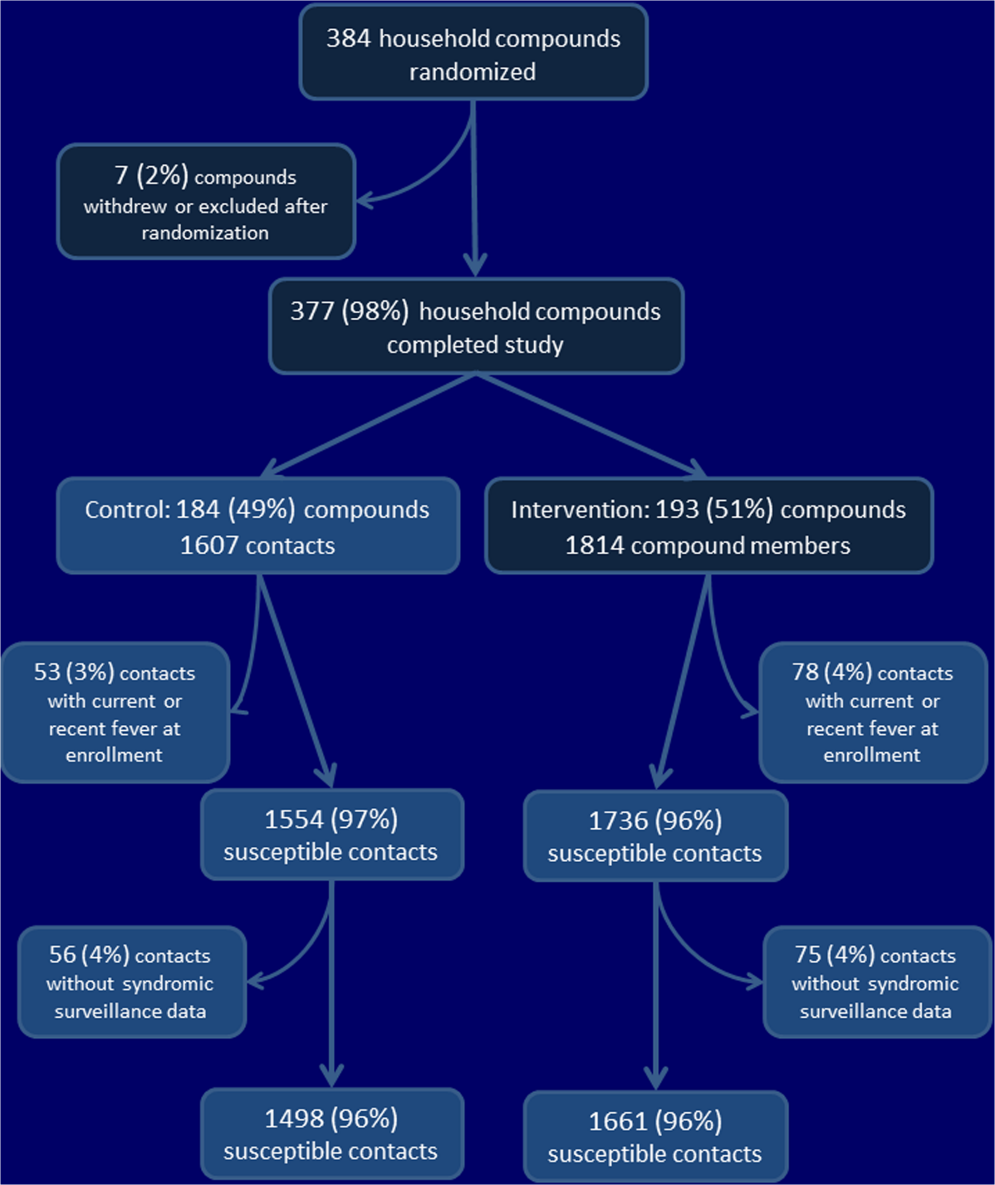
Randomization of household compounds, and exclusion of compounds and household members, Kishoregonj, Bangladesh, 2009–2010.

Study physicians screened 5178 index case-patients for eligibility (Fig 1). Among these, 766 (15%) index case-patients were eligible for enrollment; 590 (77%) consented to participate. The household compounds of 96% of index case-patients were found eligible and provided consent to participate. Among these, 195 were randomized to the intervention arm and 186 to the control arm (Fig 2). Similar numbers of index case-patients were randomized to the two treatment arms, in each study phase (S2 Table). We excluded three compounds from analysis: one intervention compound withdrew from the study, the location of one control compound required greater than 2 hours travel, and, in one control arm compound, the index case-patient was not present for more than 3 days. We confirmed influenza infection in 24 (12%) of 193 index case-patients in the intervention arm, and 36 (20%) of 184 index case-patients in the control arm. Recruitment into the trial ended on October 30, 2010 because influenza was rarely identified among outpatients at the Jahurul Islam Medical College Hospital by that time.

Compared to index case-patients in control compounds, intervention index case-patients were younger, had somewhat longer time from fever onset to study enrollment, and were less frequently identified with PCR-confirmed influenza infection (12% intervention and 20% control) (Table 1). The mean number of members per compound was 10.4 (SD 5.2) in the intervention arm and 9.7 (SD 5.2) in the control arm. Possession of household assets was largely similar in index case-patient households in the treatment arms.

**Table 1:** Baseline characteristics of index case-patients, household compounds, and household members, by treatment arm, Kishoregonj, Bangladesh, 2009–2010.

Characteristic	Intervention	Control
**Index influenza-like illness case-patients**	N = 193	N = 184
Mean age in months (SD)	121.2 (181.7)	92.5 (141.0)
# (%) of index case-patients less than 5 years old	119 (62%)	125 (68%)
# (%) of index case-patients less than 2 years old	64 (33%)	71 (39%)
Male sex	115 (60%)	112 (61%)
Cough at the time of presentation*	119 (75%)	114 (75%)
Sore throat at the time of presentation*	31 (20%)	20 (13%)
Mean interval from fever onset to study enrollment in days (SD)	2.6 (2.9)	2.1 (1.7)
Mean number of days of fever after enrollment (SD)	2.4 (2.5)	2.6 (3.2)
PCR-confirmed influenza	24 (12%)	36 (20%)
**Household compounds**	N = 193	N = 184
Mean number of households in compound (SD)	2.0 (1.1)	2.0 (1.0)
Mean number of persons living in the compound (SD)	10.4 (5.2)	9.7 (5.2)
Mean number of persons per sleeping room in index case-patient households (SD)	3.3 (1.9)	3.3 (1.4)
*Assets of index case-patient households*		
Electricity	123 (64%)	113 (61%)
Color television	48 (25%)	36 (20%)
Mobile phone	135 (70%)	129 (70%)
Watch	116 (60%)	85 (46%)
**Susceptible household members with syndromic surveillance data**	N = 1661	N = 1498
Mean age in years (SD)	24.9 (19.2)	25.7 (19.6)
# (%) < 2 years old	51 (3%)	49 (3%)
# (%) < 5 years old	175 (11%)	160 (11%)
Male sex	781 (47%)	694 (46%)
Current smoker	208 (13%)	208 (14%)
Mean number of minutes spent in cooking area per day (SD)	52 (73)	53.0 (70.0)
Multiple interactions per day with index case-patient**	750 (80%)	681 (77%)
Sleeps in the same room as index case-patient***	568 (66%)	513 (71%)

*Data not collected for 67 children, all of whom were under 5 years old and enrolled in 2009.

**Only queried in 2010; denominators were 934 for intervention arm and 890 for control arm.

***Only reported for members of index case-patient household; denominators were 863 in the intervention arm and 727 in the control group.

In the intent-to-treat analysis, 158 (9.5%) susceptible household members were identified with influenza-like illness in the intervention arm, compared to 115 (7.7%) in the control arm (Table 2). The SAR ratio was 1.24 (0.92–1.65), suggesting an elevated risk in the intervention group. The intracluster correlation was 0.37, yielding a design effect of 4.33. Among the subgroup of index case-patient household members, the SAR ratio was 1.49 (1.01–2.19). When restricted to those with index case-patient symptom onset within 48 hours preceding enrollment, there was no significant difference in the SAR of influenza-like illness between intervention and control arms. Among susceptible household members of index case-patients with PCR-confirmed influenza infection, the SAR for influenza infection was 9.6% in the intervention arm and 4.0% in the control arm (SAR ratio 2.40, 95% CI 0.68–8.47). Influenza infection was transmitted similarly among the two treatment arms to members of all households in the compounds, and to members of index case-patient households. Confidence intervals for SAR ratios of influenza-like illness were largely overlapping, across the three study phases (S3 Table).

**Table 2:** Impact of intensive handwashing promotion on secondary attack risks (SAR) of influenza-like illness, and influenza, among household compound members of index case-patients, Kishoregonj, Bangladesh, 2009–2010.

Index case-patient symptom onset	Within 7 days preceding enrollment (i.e. all participants)				Within 48 hours preceding enrollment			
Model	Intent-to-treat*		Index case-patient household members only		Overall		Index case-patient household members only	
**Secondary transmission of influenza-like illness**	Intervention	Control	Intervention	Control	Intervention	Control	Intervention	Control
**Index case-patients (N)**	193	184	189	183	136	139	136	139
**Susceptible household members (N)**	1661	1498	863	727	1232	1168	617	567
**Secondary attack risk (SAR)**	158/1661 (9.5%)	115/1498 (7.7%)	83/863 (9.6%)	47 / 727 (6.5%)	122 / 1232 (9.9%)	105 / 1168 (9.0%)	63 / 617 (10.2%)	41 / 567 (7.2%)
SAR ratio (95% CI)**	1.24 (0.93–1.65)		1.49 (1.01–2.19)		1.10 (0.81–1.50)		1.40 (0.91–2.16)	
p-value**	.14		.04		.54		.12	
**Secondary transmission of PCR-confirmed Influenza**	Intervention	Control	Intervention	Control	Intervention	Control	Intervention	Control
**Index case-patients (N) with PCR-confirmed influenza**	24	36	23	35	14	21	14	21
**Susceptible household members (N)**	177	250	96	117	102	133	64	78
**Secondary attack risk (SAR)**	17 / 177 (9.6%)	10 / 250 (4.0%)	9/96 (9.4%)	4 / 117 (3.4%)	11 / 102 (10.8%)	10/ 133 (7.5%)	6 / 64 (9.4%)	4/ 78 (5.1%)
SAR ratio (95% CI)**	2.40 (0.68–8.47)		2.74 (0.69–10.96)		1.43 (0.38–5.46)		1.83 (0.40–8.38)	
p-value**	.17		.15		.59		.44	

*All susceptible contacts in both index case-patient and secondary households included.

**Confidence intervals and P-values generated using log binomial regression model with generalized estimating equations to estimate significance of ratio of secondary attack risks in treatment arms.

No episodes of influenza infection were confirmed among susceptible contacts of index case-patients with PCR-confirmed influenza in Phase 1. The SAR ratio for influenza transmission in Phase 2 was 8.33 (95% CI 1.05–50.0) and 1.49 in Phase 3 (0.38–6.25).

We tested the effect on the SAR ratio in separate multivariable models of several variables, which differed at baseline between intervention and control arms by 10 percentage points for categorical variables or by a relative difference of 10% for continuous variables (Table 1). In models adjusting for each of these variables, the SAR ratio was similar to that found in the unadjusted analysis (Table 3). We found that confirmed influenza infection also did not affect the SAR ratio.

**Table 3:** Multivariable analysis of impact of intensive handwashing promotion on secondary attack risks (SAR) of influenza-like illness, and influenza, among household compound members of index case-patients, Kishoregonj, Bangladesh, 2009–2010.

Characteristic	Intervention	Control	Adjusted Relative Risk (95% CI)
**Intent-to-treat analysis**	-	-	1.24 (0.93–1.65)
Mean index case-patient age	121.2 (181.7)	92.5 (141.0)	1.25 (0.93–1.67)
Mean interval from fever onset to study enrollment in days (SD)	2.6 (2.9)	2.1 (1.7)	1.24 (0.94–1.65)
Watch ownership	116 (60%)	85 (46%)	1.29 (0.96–1.74)
PCR-confirmed influenza	24 (12%)	36 (20%)	1.24 (0.93–1.65)

**Fig 3:**
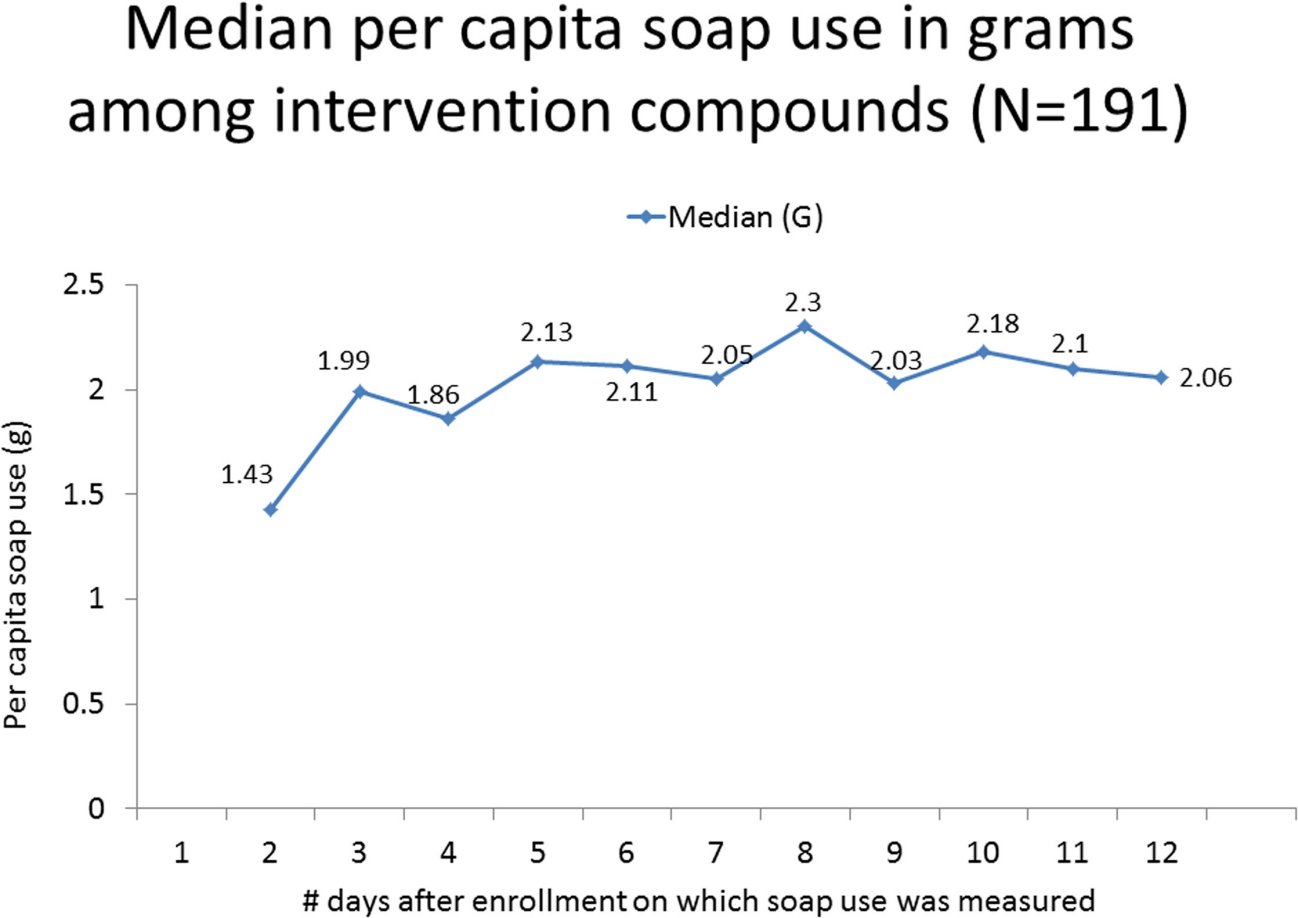
Median per capita soap use in grams, by day of enrollment, among intervention compounds, Kishoregonj, Bangladesh, 2009–2010 (N = 191).

We examined the presence or absence of soap and water at the handwashing station during each of the first 10 days of surveillance from 180 intervention household compounds. Soap was present at the handwashing station for at least 7 days in all 180 compounds and on all 10 days in 133 (74%). Soap and water together were present at the handwashing station for 7 or more of the first 10 days in 99% of household compounds, with water and soap observed together on all 10 days in 99 (55%) household compounds. We restricted soap use analysis to measurements of soap weight during the first 12 days of enrollment, since thereafter, data collection had stopped in 25% or more of intervention compounds based on the resolution of index case-patient symptoms. When examining the compound’s mean daily per capita soap use over the first 12 days, we found a median per capita soap consumption of 2.3 grams (interquartile range: 1.7 to 3.2 grams). Estimates of median daily per capita soap use for each day of measurement are shown in Fig 3. Maximal per capita soap use on any one of the first 12 days of enrollment was 4.6 grams and was observed on a median of the 7th day of participation (interquartile range: 5 to 9 days). We found no significant association between per capita soap use and risk of ILI transmission in the household.

In multivariable analysis of data from household members from the control arm, index case-patient illness onset occurring within the 24 hours prior to enrollment was significantly associated with influenza-like illness detection among susceptible contacts (Table 4). Susceptible contacts less than 2 years old were 5.51 times as likely as members 2 years or older to develop influenza-like illness (95% CI 3.43–8.85); index case-patient with fever <24 hours prior to enrollment (RR_adj_ 1.91, 95% CI 1.18–3.10) and multiple daily interactions with the index case-patient (RR_adj_ 1.95, 95% CI 1.17–3.26) were also associated with influenza like illness detection in susceptible contacts in the control arm, when adjusted for susceptible contact age less than 2 years. Susceptible contacts less than 5 years old were 4.88 times as likely as members 5 years or older to develop influenza-like illness during the surveillance period in a model adjusting for index case-patient fever onset within 24 hours prior to enrollment and reported multiple daily interactions with the index case-patient (95% CI 3.07–7.75).

**Table 4:** Individual-level risk factors for secondary transmission of influenza-like illness among susceptible household members in the control arm, Kishoregonj, Bangladesh, 2009–2010 (N = 1498).

Characteristic	Attack rate among exposed to characteristic	Attack rate among unexposed to characteristic	Attack rate ratio (95% CI, p-value) in bivariate analysis^1^	Adjusted attack rate ratio (95% CI, p-value) in multivariable analysis^1^ #
**Index case-patient characteristics**				
**Index case-patient < 2 years old**	47 / 598 (7.9%)	68 / 900 (7.6%)	1.04 (0.68–1.58) p = .85	
**Index case-patient < 5 years old**	88 / 1025 (8.6%)	27 / 473 (5.7%)	1.50 (0.80–2.84) p = .21	
**Male index case-patient**	59 / 857 (6.9%)	56 / 641 (8.7%)	1.26 (0.82–1.96) p = .28	
**Index case-patient a current smoker** ^**2**^	1 / 78 (1.3%)	26 / 395 (6.6%)	0.19 (0.03–1.50) p = .12	
**Index case-patient with cough during illness**	71 / 898 (7.9%)	30 / 351 (8.6%)	0.92 (0.56–1.53) p = .76	
**Index case-patient with fever onset 24 hours prior to enrollment**	64 / 598 (10.7%)	51 / 901 (5.7%)	1.89 (1.25–2.86) p = .002	1.91 (1.18–3.10) p = .01
**Susceptible household member characteristics**				
**Contact < 2 years old**	15 / 49 (30.6%)	100 / 1449 (6.9%)	4.44 (2.74–7.19) p<.0001	5.51 (3.43–8.85) p<.0001
**Contact < 5 years old**	39 / 160 (24.4%)	76 / 1338 (5.7%)	4.29 (2.83–6.52) p<.0001	4.88 (3.07–7.75) p<.0001
**Male sex**	56 / 694 (8.1%)	59 / 745 (7.3%)	0.90 (0.61–1.35) p = .64	
**Parent of index case-patient** ^**3**^	18 / 268 (6.7%)	81 / 935 (8.7%)	0.78 (0.48–1.24) p = .29	
**Living in index case-patient household**	47 / 727 (6.5%)	68 / 771 (8.8%)	0.73 (0.51–1.05) p = .09	
**Interacts multiple times daily with index case-patient** ^**4**^	72 / 681 (10.6%)	12 / 209 (5.7%)	1.84 (1.10 = 3.09) p = .02	1.94 (1.16–3.26) p = .01
**Sleeps in same room as index case-patient** ^**5**^	57 / 688 (8.3%)	42 / 513 (8.2%)	1.01 (0.68–1.51) p = .95	
**Current smoker**	10 / 208 (4.8%)	105 / 1290 (8.1%)	0.59 (0.31–1.12) p = .11	
**Reports spending any time in cooking space**	72 / 925 (7.8%)	43 / 573 (7.5%)	1.04 (0.70–1.54) p = .86	
**Reports spending 90 or more minutes in cooking space**	26 / 406 (6.4%)	89 / 1092 (8.2%)	0.79 (0.52–1.19) p = .26	

^1^ Attack rates for influenza-like illness calculated for susceptible members in the control arm who were exposed and unexposed to each characteristic at baseline. Attack rate ratios and confidence intervals generated using log binomial regression models, with generalized estimating equations to account for clustering among household members.

^**2**^Analysis restricted to household members of index case-patients > 5 years old.

^**3**^Information missing for 295 household members.

^**4**^Only queried in 2010.

^**5**^Only reported for members of index case-patient household.

# multivariable model includes the following variables: contact < 2 years old (or contact < 5 years old); Index case-patient with fever onset 24 hours prior to enrollment; and contact interacts multiple times daily with index case-patient.

Among the 250 susceptible contacts of the index case-patients with laboratory-confirmed influenza in the control arm, 10 were confirmed to have influenza infection, all of whom were contacts of index case-patients whose fever had begun during the 48 hours before enrollment into the study. In bivariate analyses, risk factors for transmission of influenza to susceptible contacts of index case-patients with PCR-confirmed influenza infection were index case-patient fever onset during the 24 hours preceding enrollment (RR 5.58, 95% CI 0.84–37.0, p = .07), susceptible contact age < 2 years (RR 4.52, 95% CI 1.23–16.55, p = .02), and susceptible contact age < 5 years (RR 2.88, 95% CI 1.06–0.57, p = .06).

## Discussion

In this study in a low-income rural area of Bangladesh, intensive handwashing promotion begun after the index case patient sought care was not associated with lower rates of influenza-like illness or laboratory-confirmed influenza virus infection among compound or household members. The findings were similar when restricting the analysis to susceptible contacts exposed early during the course of the index case-patient’s illness (within 48 hours of illness onset). The handwashing intervention conferred no differential protective effect for individuals residing in the same household, sharing the same cooking pot and sleeping space, as the index case-patient, compared to those residing in other households within the compound.

Young children and those reporting frequent contact with the index case-patient were most susceptible to secondary transmission of respiratory pathogens. In addition, with surveillance beginning immediately upon enrollment of the index case-patient, we found that contacts were more likely to develop influenza-like illness and influenza early in the course of the index case-patient’s illness, compared with those who were surveyed after an index case-patient had been ill for more than one day. Our findings confirm prior work suggesting that transmission begins early in the course of the respiratory infection[14].

Does handwashing actually prevent transmission of influenza and other respiratory pathogens? In contrast to our findings, other studies support the protective effects of handwashing for prevention of influenza and other respiratory infections. Handwashing promotion and soap provision resulted in a significant reduction in pneumonia risk of 50% among children in a cluster-randomized community-based trial in communities in Karachi, Pakistan.[23] Talaat and colleagues found significant reductions in diarrhea and PCR-confirmed influenza in a cluster-randomized controlled trial evaluating handwashing promotion in schoolchildren in Egypt.[24]

Several trials have examined the effect of handwashing promotion on household secondary transmission.[7–9] In a study designed similarly to ours in Hong Kong, hand cleansing promotion was shown to reduce intra-household influenza transmission in the subgroup for whom the intervention was applied within 48 hours following the onset of the index case-patient’s symptoms.[8] Studies from New York and Bangkok found results largely similar to ours.[7,9] There are several possible explanations for the lack of effect of handwashing promotion on influenza and respiratory pathogen transmission, as shown in our work. First, we may not have changed handwashing behavior in intervention compounds despite our intensive intervention. Measurements of soap consumption based on soap weights have shown that, absent a handwashing intervention, an individual in Bangladesh and Pakistan uses an estimated 1.5 to 2 grams of soap per day for various purposes, including but not limited to handwashing (Meghana Gadgil, personal communication)[23]. In our intervention compounds, we found that median per capita soap consumption ranged from 1.4 to 2.3 grams, although maximal per capita soap consumption was a median of 5 grams (on Day 7). Thus, on some intervention days, soap use was substantially increased in the intervention households but it appears that the intervention did not result in early and sustained increases in soap use well beyond the norm. The absence of an association between the per capita soap consumption and risk of ILI transmission suggests that, even in households with relatively high soap consumption, there was no clear protective effect. We did not discourage the use of study-provided soap for other purposes such as bathing, laundry, or dishwashing. We also did not track whether other soap was available in the household compounds through the intervention period and, thus, we cannot know whether overall soap use increased or not. The adoption of a regular handwashing habit may take time. In a year-long trial of handwashing promotion in Karachi, Pakistan, diarrhea rates were 50% lower in the intervention group over the course of a year of surveillance but did not differ at all during the first two months following the introduction of the intervention, suggesting that the behavior had not changed early on.[25] We do not have sufficient information regarding which household members increased handwashing behavior; since youngest children were at greatest risk of becoming infected, it is possible that handwashing behavior of older individuals increased but was insufficient to protect those who did not or could not wash their own hands to protect themselves. We did not measure behavior in the control group because we were sensitive to the possibility that handwashing would increase even in the absence of intervention solely because of reactivity to monitoring [26] and, thus, we cannot be certain whether or not handwashing behavior in the intervention group was similar to that in the control group.

It is also possible that transmission of influenza in crowded Bangladeshi homes occurs principally as a result of aerosolized virus in droplets, rather than by direct contact[14]. In this case, improving hand hygiene may little influence transmission between household members.

Our findings in the secondary analysis of risk factors for transmission in the control arm may be explained if the period of peak exposure was early during the course of the illness, affirming the findings of Cowling and colleagues that handwashing promotion is particularly effective when applied soon after illness onset, rather than later in the course of illness[8]. We may have enrolled index case-patients and household members too late to prevent transmission in most households. Handwashing may be most effective at preventing intra-household transmission of viral respiratory illness if performed thoroughly early in the course of the episode. The health worker seeking to prevent intra-household transmission may be unsuccessful if s/he attempts to influence hand hygiene practices upon seeing a patient already ill with influenza-like illness, either because transmission may have already occurred if the index case-patient has been ill for several days, or because changing a habitual behavior such as handwashing takes time. Since hand hygiene is often promoted routinely for diarrhea prevention, it may be most efficient and effective to incorporate viral respiratory illness prevention into ongoing promotion, rather than through targeting of individuals who are already ill with respiratory infection.

Finally, it is possible that our interactive, compound-level approach to handwashing promotion may have inadvertently increased transmission in the intervention household compounds. Similar findings were noted in three similar trials evaluating the effects of hand hygiene and face mask interventions on ILI transmission in households.[6–8] Risk estimates for the intent-to-treat model exceeded 1 suggesting higher transmission rates in the intervention compounds, compared to controls; there was a statistically significant increase in the SAR among index case-patient household members in the intervention group compared to controls. Our study objectives and design demanded a household- or compound-level intervention since we were seeking to stop transmission once at least one person in the household was already ill with influenza. However, the compound-level nature of our intervention, in which we gathered compound members together daily in order to promote handwashing, may have increased the potential for transmission via direct contact. However, at baseline, most compound members reported multiple daily interactions with the index case-patient, irrespective of the latter’s age, reflecting the frequent socialization among compound members that was likely occurring in both intervention and control arms. As part of the intervention, we placed a single handwashing station in the household compound and encouraged all household members to wash hands; it is possible that the handwashing station itself served as a fomite for pathogen transmission. Although it would not have been appropriate given our study design, mass media represents an approach to behavior change communication that would not necessarily risk bringing large groups of persons together.

It is biologically plausible that handwashing with soap interrupts influenza and other respiratory pathogen transmission. However, we have failed to show benefit from attempting to increase handwashing rapidly once a case was already present in the household. To apply handwashing promotion effectively for prevention of influenza prevention, if may be more helpful to be proactive rather than reactive. Efforts aimed at changing the social norm of handwashing, particularly at times of possible respiratory pathogen transmission, may be more effective if they are ongoing in order to prevent transmission of a variety of diseases, including influenza.

As evident by the need to modify enrollment criteria repeatedly, our study faced several limitations. Low enrollment rates and new information from another household transmission study[8] led us to change study inclusion and exclusion criteria two times over the course of the study. However, block randomization ensured that we enrolled similar numbers of intervention and control households in each Phase. Moreover, similar point estimates were observed for the SAR ratio in Phase 2 and Phase 3, which together represented 89% of all household compounds enrolled. Confidence intervals for the SAR ratio for Phase 1 overlapped those for Phases 2 and 3. There were some differences between treatment arms at baseline, despite block randomization. However, the increase in the SAR ratio in the multivariable models that included measured confounders is consistent with a higher rate of acquisition of influenza-like illness in the intervention arm, compared to the SAR ratio estimated in the intent-to-treat analysis. Therefore, the lack of beneficial effect of the handwashing intervention is likely not explained by unmeasured confounders as a result of imbalance between the treatment arms.

We completed data collection 10 days after the end of the index case-patient’s symptomatic period. Findings from early investigations of the 2009 H1N1 pandemic noted that the mean generation interval between onset of index case-patient symptoms and onset of the secondary case-patient’s symptoms was 2.6 days (95% CI 2.5 to 2.8 days), a 10-day interval following the conclusion of the index case-patient’s symptoms should have been sufficient to detect the bulk of secondary transmission. Secondary cases could certainly have contributed to tertiary cases and so on. The study design would not have captured the full extent of the intervention’s effects on tertiary transmission. Follow-up for longer than 10 days following index case-patient symptom resolution was beyond the logistical and funding scope of the project.

We relied on symptom reporting from the female head of the household compound in our surveillance of influenza-like illness; In this cultural context, where joint families live closely together, we judged that the female head of the compound would be most likely to be aware of symptoms among members of the compound. This approach was particularly relevant for individuals who were away from home at the time of the field worker’s visit to the compound. It is possible that individuals who were not at home at the time of surveillance were experiencing symptoms of which the female head of the compound was not aware and, hence, that these individuals were less likely to be counted as secondary cases. Findings from this study may be generalizable to other parts of rural South Asia, but may not be similarly applicable to less densely populated rural areas such as those often found in sub-Saharan Africa.

In conclusion, handwashing may reduce intra- and inter-household transmission of influenza and other respiratory pathogens but our findings indicate that a reactive approach to handwashing promotion, particularly several days after illness onset, may be ineffective in the context of rural Bangladesh.

## Supporting Information

S1 Checklist (DOC)

S1 Protocol (DOC)

S1 Text (DOCX)

S1 Table Phases of enrollment of index case-patients and susceptible contacts, Bangladesh Interruption of Secondary Transmission of Influenza Study (BISTIS), Kishoregonj, Bangladesh, 2009–2010. (DOCX)

S2 Table Randomization of household compounds, and exclusion of compounds and household members. (DOCX)

S3 Table Secondary attack ratios of influenza-like illness, and influenza, among household compound members of index case-patients, by study phase and in total, Kishoregonj, Bangladesh, 2009–2010. (DOCX)
