# Supplementary material for: Impact of Intensive Handwashing Promotion on Secondary Household Influenza-Like Illness in Rural Bangladesh: Findings from a Randomized Controlled Trial
Source: PLoS One. 2015 Jun 11;10(6):e0125200. doi: 10.1371/journal.pone.0125200 (PMC4465839; doi:10.1371/journal.pone.0125200)
Supplement: S1 Protocol — (DOC) [file pone.0125200.s002.doc]

| 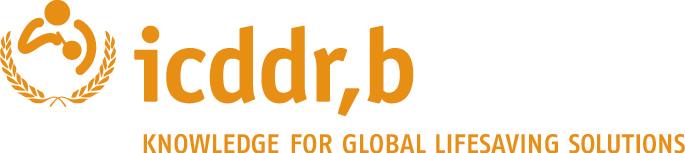 | | | | RRC APPLICATION FORM | | |
| --- | --- | --- | --- | --- | --- | --- |
| RESEARCH PROTOCOL **NUMBER: 2009-004** | **FOR OFFICE USE ONLY** | | | | | |
|  | RRC Approval: | | | Yes / | No | Date: 2 Feb 2009 |
|  | ERC Approval: | | | Yes / | No | Date: 31 Mar 2009 |
|  | AEEC Approval: | | | Yes / | No | Date: |
| **Protocol Title:** **Prevention of secondary transmission of human influenza by promoting handwashing with soap:**  **The Bangladesh Interruption of Secondary Transmission of Influenza Study (BISTIS)** | | | | | | |
| **Short title (in 50 characters including space):** **Bangladesh secondary transmission handwashing protocol** | | | | | | |
| **Theme: (Check all that apply)**  Nutrition  Emerging and Re-emerging Infectious Diseases  Population Dynamics  Reproductive Health  Vaccine Evaluation  HIV/AIDS | | | Environmental Health  Health Services  Child Health  Clinical Case Management  Social and Behavioural Sciences | | | |
| **Key words:** Influenza, secondary transmission, handwashing, Bangladesh | | | | | | |
| **Relevance of the Protocol:**  Influenza is an important cause of respiratory illnesses among children and adults in Bangladesh. The next influenza pandemic is expected to spread rapidly in resource-poor settings. Influenza viruses spread from human-to-human via large respiratory droplets. The greatest risk of transmission from personal contact comes from the household contact of an index case. However, there is no published data available on the secondary attack ratio or the risk factors for secondary transmission of influenza among household contacts from the index case-patients of low-income countries. Moreover, we do not have any evidence whether promoting handwashing with soap can reduce the risk of secondary transmission of influenza among household contacts. The proposed study will determine the secondary attack ratio and the risk factors for secondary transmission of influenza and assess the impact of handwashing intervention on influenza transmission among household contacts in Bangladesh. | | | | | | |
| **Centre’s Priority** **(as per Strategic Plan, to be imported from the attached Separate Word Sheet):**  4.1 Define the epidemiology and burden of selected infectious diseases and identify effective strategies for prevention and control.  4.4 Enhance the capacity to investigate, study, and manage outbreaks of communicable diseases in the region. | | | | | | |
| **Programmes:**  Child Health Programme  Nutrition Programme  Programme on Infectious Diseases & Vaccine Science  Poverty and Health Programme | | | Health and Family Planning Systems Programme    Population Programme  Reproductive Health Programme  HIV/AIDS Programme | | | |
| **Principal Investigator (Should be a Centre’s staff)**  Dr. Stephen P. Luby  **Address (including e-mail address):**  Head,  PIDVS, HSID, ICDDR,B.  Mohakhali, Dhaka 1212  Email: sluby@icddrb.org | | | **DIVISION:**  CSD  LSD  HSID  PHSD | | | |
| **Co-Principal Investigator(s): Internal** | | | | | | |
| **Co-Principal Investigator(s): External:**  (Please provide full official address including e-mail address and Gender)  **Pavani K. Ram, MD**  Assistant Professor  Department of Social and Preventive Medicine  School of Public Health and Health Professions  University at Buffalo  3435 Main Street, Rm. 273 Farber Hall  Buffalo, NY 14214  E-mail: [pkram@buffalo.edu](mailto:pkram@buffalo.edu)  Gender: Female | | | | | | |
| **Co-Investigator(s): Internal:**  Tasnim Azim, Eduardo Azizz-Baumgartner, W. Abdullah Brooks, Stephen Luby, Mustafizur Rahman and Rashid Uz Zaman | | | | | | |
| **Co-Investigator(s): External**  (Please provide full official address including e-mail address and Gender)  **Joe Bresee,** Influenza Division, CDC, 1600 Clifton road, Atlanta, GA 30333, USA. [jsb6@cdc.gov](mailto:jsb6@cdc.gov). Male  **Alicia Fry,** Influenza Division, CDC, 1600 Clifton road, Atlanta, GA 30333, USA. [agf1@cdc.gov](mailto:agf1@cdc.gov). Female | | | | | | |
| **Student Investigator(s): Internal (Centre’s staff):** | | | | | | |
| **Student Investigator(s): External:**  (Please provide full address of educational institution and Gender)  **Margaret DiVita,** PhD Candidate, Department of Social and Preventive Medicine, School of Public Health and Health Professions, University at Buffalo, 3435 Main Street, Rm. 273 Farber Hall, Buffalo, NY 14214, [mdivita@udsmr.org](mailto:mdivita@udsmr.org). Female | | | | | | |
| **Collaborating Institute(s):** Please Provide full address   | Country | USA | | --- | --- | | Contact person | **Pavani K. Ram, MD** | | Department  (including Division, Centre, Unit) | Department of Social and Preventive Medicine | | Institution  (with official address) | School of Public Health and Health Professions  University at Buffalo  3435 Main Street, Rm. 273 Farber Hall  Buffalo, NY 14214 | | Directorate  (in case of GoB i.e. DGHS) |  | | Ministry (in case of GoB) |  |   **Institution # 1** | | | | | | |
| **Institution # 2**   | Country | USA | | --- | --- | | Contact person | **Alicia Fry, MD MPH** | | Department  (including Division, Centre, Unit) | Influenza Division, National Centre for Immunization and Respiratory Diseases | | Institution  (with official address) | Centers for Disease Control and Prevention | | Directorate  (in case of GoB i.e. DGHS) |  | | Ministry (in case of GoB) |  | | | | | | | |
| **Institution # 3**   | Country |  | | --- | --- | | Contact person |  | | Department  (including Division, Centre, Unit) |  | | Institution  (with official address) |  | | Directorate  (in case of GoB i.e. DGHS) |  | | Ministry (in case of GoB) |  |   Note: If more than 3 collaborating institutions are involved in the research protocol, additional block(s) can be inserted to mention its/there particular(s). | | | | | | |
| **Population: Inclusion of special groups (Check all that apply):**   | Sex  Male  Female  Age  0 – 4 years  5 – 9 years  10 – 19 years  20 – 64 years  65 + | Pregnant Women  Fetuses  Prisoners  Destitutes  Service Providers  Cognitively Impaired  CSW  Others (specify      )  Animal | | --- | --- |   NOTE It is the policy of the Centre to include men, women, and children in all research projects involving human subjects unless a clear and compelling rationale and justification (e.g. gender specific or inappropriate with respect to the purpose of the research) is there. Justification should be provided in the `Sample Size’ section of the protocol in case inclusiveness of study participants is not proposed in the study. | | | | | | |
| **Project/study Site (Check all the apply):**  Dhaka Hospital  Matlab Hospital  Matlab DSS Area  Matlab non-DSS Area  Mirzapur  Dhaka Community  Chakaria  Abhoynagar | | Mirsarai  Patyia  Other areas in Bangladesh: **Jahurul Islam Medical College Hospital, Kishorgonj**  Outside Bangladesh  Name of Country:  Multi Centre Trial  (Name other countries involved): | | | | |
| Type of Study (Check all that apply):   | Case Control Study  Community-based Trial/Intervention  Program Project (Umbrella)  Secondary Data Analysis  Clinical Trial (Hospital/Clinic)  Family Follow-up Study | Cross Sectional Survey  Longitudinal Study (cohort or follow-up)  Record Review  Prophylactic Trial  Surveillance/Monitoring  Others: | | --- | --- |   NOTE: Does the study meet the definition of clinical studies/trials given by the International Committee of Medical Journal Editors (ICMJE)? Yes  No    Please note that the ICMJE defined clinical trial as “*Any research project that prospectively assigns human subjects to intervention and comparison groups to study the cause-and-effect relationship between a medical intervention and a health outcome*”.    If YES, after approval of the ERC, the PI should complete and send the relevant form to provide required information about the research protocol to the Committee Coordination Secretariat for registration of the study into websites, preferably at the [www.clinicaltrials.gov](http://www.clinicaltrials.gov/). It may please be noted that the PI would require to provide subsequent updates of the research protocol for updating protocol information in the website. | | | | | | |
| **Targeted Population (Check all that apply):**  No ethnic selection (Bangladeshi)  Bangalee  Tribal group | | Expatriates  Immigrants  Refugee | | | | |
| **Consent Process (Check all that apply):**  Written  Oral  None | | Bengali Language  English Language | | | | |
| **Proposed Sample Size:**  Sub-group (Name of subgroup (e.g. Men, Women) and Number   | Name | Number | Name | Number | | --- | --- | --- | --- | | (1) Index case patients (2009) | 200 | (3)Index case patients (2010) | 400 | | (2) Household contacts (2009) | 2000 | (4) Household Contacts (2010) | 4000 |   Total sample size: 6600 | | | | | | |
| a) Will the specimen be stored for future use? Yes  No  b) If yes, how long the specimens be preserved? _20 years.  c) Will consent be obtained from study participants Yes  No  NA  for the specimen be stored for future, for unrelated use  without further taking consent?  d) What types of tests will be carried out with the preserved samples? Tests will be for identification of respiratory illness other than influenza  e) Will the samples be shipped to other country(ies)? Yes  No  NA  f) If yes, name of institution(s) and country(ies): ______ **surplus** aliquots may be shipped to the CDC in Atlanta, GA, USA for confirmation of unsubtypable samples and for random quality control.___  g) Will the surplus/unused specimen be returned to the Centre? Yes  No  NA  h) Who will be the custodian of the specimen at the Centre  and when shipped outside of the country(ies)?: __Mustafizur Rahman , PhD, ICDD,B Influenza Laboratory__  i) Who will be the owner(s) of the samples? : _______________ICDDR,B  j) Has a MoU been made for the protocol covering the  specimen collection, storage, use and ownership? Yes  No  NA  k) If yes, please attach a copy. | | | | | | |
| **Determination of Risk: Does the Research Involve (**Check all that apply**):**   | Human exposure to radioactive agents?  Fetal tissue or abortus?  Investigational new device?  (specify:)  Existing data available from Co-investigator | Human exposure to infectious agents?  Investigational new drug  Existing data available via public archives/sources  Pathological or diagnostic clinical specimen only  Observation of public behaviour  New treatment regime | | --- | --- | | | | | | | |
| **Could the information recorded about the individual if it became known outside of the research:**   | Yes | No | Is the information recorded in such a manner that study participants can be identified from information provided directly or through identifiers linked to the study participants? | | --- | --- | --- | | Yes | No | Does the research deal with sensitive aspects of the study participants’ behaviour; sexual behaviour, alcohol use or illegal conduct such as drug use? |  | Yes | No | Place the study participants at risk of criminal or civil liability? | | --- | --- | --- | | Yes | No | Damage the study participants’ financial standing, reputation or employability, social rejection, lead to stigma, divorce etc.? | | | | | | | |
| **Do you consider this research (Check one):**  Greater than minimal risk  No more than minimal risk  Only part of the diagnostic test  Minimal Risk is "a risk where the probability and magnitude of harm or discomfort anticipated in the proposed research are not greater in and of themselves than those ordinarily encountered in daily life or during the performance of routine physical, psychological examinations or tests. For example, risk of drawing a small amount of blood from a healthy individual for research purposes is no greater than the risk of doing so as a part of routine physical examination". | | | | | | |
| **Yes/ No**  Is the proposal funded?  If yes, sponsor Name: (1) Centers for Disease Control and Prevention (CDC)    (2) | | | | | | |
| **Yes/No**  Is the proposal being submitted for funding?  If yes, name of funding agency: (1)  (2) | | | | | | |
| Do any of the participating investigators and/or member(s) of their immediate families have an equity relationship (e.g. stockholder) with the sponsor of the project or manufacturer and/or owner of the test product or device to be studied or serve as a consultant to any of the above?  ***IF YES, a written statement of disclosure to be submitted to the Centre’s Executive Director.***  Dates of Proposed Period of Support Cost Required for the Budget Period ($)  (Day, Month, Year - DD/MM/YY)   | **Years** | **Direct Cost** | **Indirect Cost** | **Total Cost** | | --- | --- | --- | --- | | **Year-1** | 127,040 | 40,125 | 166,573 | | **Year-2** |  |  | 0 | | **Year-3** |  |  | 0 | | **Year-4** |  |  | 0 | | **Year-5** |  |  | 0 | | **Total** | 127,040 | 40,125 | 167,165 |     Beginning Date : 01 May 2009    End Date : 31 Decmeber 2010 | | | | | | |
| **Certification by the Principal Investigator**  I certify that the statements herein are true, complete and accurate to the best of my knowledge. I am aware that any false, fictitious, or fraudulent statements or claims may subject me to criminal, civil, or administrative penalties. I agree to accept the responsibility for the scientific conduct of the project and to provide the required progress reports including updating protocol information in the SUCHONA (Form # 2) if a grant is awarded as a result of this application.  ___________ ____________  **Signature of PI Date** | | | | | | |
| **Approval of the Project by the Division Director of the Applicant**  The above-mentioned project has been discussed and reviewed at the Division level as well by the external reviewers. The protocol has been revised according to the reviewers’ comments and is approved.   |  |  |  | | --- | --- | --- | | Name of the Division Director | Signature | Date of Approval | | | | | | | |

[**RRC APPLICATION FORM 1**](#__RefHeading___Toc268031174)

[Project Summary 9](#__RefHeading___Toc268031175)

[Description of the Research Project 11](#__RefHeading___Toc268031176)

[Hypothesis to be Tested: 11](#__RefHeading___Toc268031177)

[Specific Aims: 12](#__RefHeading___Toc268031178)

[Background of the Project including Preliminary Observations 12](#__RefHeading___Toc268031179)

[Research Design and Methods 15](#__RefHeading___Toc268031180)

[Measures of interest 25](#__RefHeading___Toc268031181)

[Laboratory methods 28](#__RefHeading___Toc268031182)

[*Sample Size Calculation and Outcome Variable(s)* 31](#__RefHeading___Toc268031184)

[*Data Safety Monitoring Plan (DSMP)* 34](#__RefHeading___Toc268031185)

[*Data Analysis* 35](#__RefHeading___Toc268031186)

[*Ethical Assurance for Protection of Human Rights* 38](#__RefHeading___Toc268031187)

[Use of Animals 39](#__RefHeading___Toc268031188)

[*Literature Cited* 40](#__RefHeading___Toc268031189)

[*Dissemination and Use of Findings* 42](#__RefHeading___Toc268031190)

[*Collaborative Arrangements* 42](#__RefHeading___Toc268031191)

[Biography of the Investigators 42](#__RefHeading___Toc268031192)

[Budget Justifications 66](#__RefHeading___Toc268031195)

[Personnel 66](#__RefHeading___Toc268031196)

[Other Support 67](#__RefHeading___Toc268031197)

[Appendix 1: Figure and Details about Bari 68](#__RefHeading___Toc268031198)

[Appendix 2: Adult Consent Form: Specimen Collection 69](#__RefHeading___Toc268031203)

[Appendix 3: Parent or Guardian Consent Form: Specimen Collection 72](#__RefHeading___Toc268031204)

[Appendix 4: Child Assent Form: Specimen Collection 75](#__RefHeading___Toc268031205)

[Appendix 5: Consent Form: Study Enrollment, Household/Bari 77](#__RefHeading___Toc268031206)

[Appendix 6: Bari Eligibility Form 91](#__RefHeading___Toc268031214)

[Appendix 7: Bari Drawing Form 93](#__RefHeading___Toc268031215)

[Appendix 8: Household Contact Enumeration Form 95](#__RefHeading___Toc268031218)

[Appendix 8b: Enrollment Day Sick List for all Bari members 96](#__RefHeading___Toc268031219)

[Appendix 9: Household Level Questionnaire/Observation Form 97](#__RefHeading___Toc268031254)

[Appendix 10: Illness Tracking Form (for all ages) Version 18.5.10 135](#__RefHeading___Toc268031255)

[Appendix 18: BISTIS Follow Up Survey Form 158](#__RefHeading___Toc268031266)

[Appendix 19: Follow Up Soap Tracking Form 177](#__RefHeading___Toc268031267)

[Appendix 21a: Follow Up Phone Call Illness Tracking Form: Ages ≥ 5 Years Old, Page 1 180](#__RefHeading___Toc268031294)

[Appendix 21a: Follow Up Phone Call Illness Tracking Form: Ages ≥ 5 Years Old, Page 2 181](#__RefHeading___Toc268031295)

[Appendix 22: Responses to the comments from the external reviewers 202](#__RefHeading___Toc268031296)

Check here if appendix is included

| Project Summary Describe in concise terms, the hypothesis, objectives, and the relevant background of the project. Also describe concisely the experimental design and research methods for achieving the objectives. This description will serve as a succinct and precise and accurate description of the proposed research is required. This summary must be understandable and interpretable when removed from the main application. |
| --- |
| **Principal Investigator(s):** Dr. Eduardo Azizz-Baumgartner |
| **Research Protocol Title:** Prevention of secondary transmission of human influenza by promoting handwashing with soap: The Bangladesh Interruption of Secondary Transmission of Influenza Study (BISTIS) |
| **Total Budget US$:** 166,573 Beginning Date : 1 May 2009 Ending Date: 31 December 2010 |
| The next influenza pandemic is expected to spread rapidly in resource-poor settings. Influenza viruses spread from human-to-human via large respiratory droplets. Transmission via large-particle respiratory droplets is believed to be mediated by close contact between infected and susceptible persons or contact with droplet-contaminated fomites. Close contact between infected and susceptible persons may consist of skin-to-skin contact (e.g., via hands) or inhalation of respiratory droplets (e.g., due to talking, coughing, or sneezing by the infected person). Airborne transmission, which is expected to result in transmission over long distances (>1 meter) and which would be mediated by ventilation, is believed to be uncommon. Therefore, the greatest risk of transmission from personal contact comes from those people who are closest to an index case, such as contacts living in the same household. There are, to date, no published estimates of the secondary attack ratio of influenza among household contacts of index case-patients in low-income countries. Moreover, we do not have data on the risk factors for secondary transmission of influenza from index case-patients to their household contacts. There is some data for the benefits of promoting handwashing with soap on the risk of all-cause acute respiratory illness among children < 15 years old in a resource-poor setting in Pakistan. But, we do not have evidence that promoting handwashing with soap will acutely reduce the risk of secondary transmission. Therefore, we propose to conduct a study in rural Bangladesh to assess the following:  The secondary attack ratio of influenza among household contacts of an index case-patient with influenza  The risk factors for secondary transmission of influenza from an index case-patient to household contacts  The impact of promoting handwashing with soap on the risk of secondary transmission of influenza from an index case-patient to household contacts  The impact of a handwashing promotion intervention on handwashing behavior at 5-6 months following the intervention.  To complete our study objectives, we will conduct a randomized controlled trial in the Kishoregonj area of Bangladesh, building on ongoing influenza surveillance at the Jahurul Islam Medical College Hospital (JIMCH). We will identify eligible index case-patients with influenza at the JIMCH, two local Upazilla Health Complex (UHC) clinics, pharmacies and other local health care providers. Index case-patients will be identified as having influenza using a rapid diagnostic test for influenza (QuickVue®). Our study workers will visit the *bari*, obtain informed consent, and collect baseline information about the *bari*, including information on crowding, ventilation of the cooking space, and smoking status of *bari* residents. We will then assign *baris* to the intervention or control arm at random using a block randomization strategy. The intervention will consist of promotion of handwashing with soap and the provision of soap and a water vessel to facilitate handwashing; the intervention will be based on the Social Cognitive Theory. We will then follow up intervention and control *baris* for a total of 10 days following the resolution of the index case-patient’s illness, in order to track illness in each bari resident. At the conclusion of the illness tracking, control *baris* will be provided bars of soap. At a future time, the control baris will be provided a water vessel, and the same handwashing promotion session provided to intervention *baris*. We will follow-up with all enrolled baris 5-6 months after illness tracking was completed to assess handwashing behavior. We will use objective measures of handwashing behavior, and will also assess knowledge about influenza. |
| KEY PERSONNEL (List names of all investigators including PI and their respective specialties)     | **Name** | **Professional Discipline**  **/ Specialty** | **Role in the Project** | | --- | --- | --- | | Eduardo Azizz-Baumgartner | ICDDR,B | Co-Principal Investigator | | Pavani K. Ram | University at Buffalo | Co-Principal Investigator | | Tasnim Azim | ICDDR,B | Co-Investigator | | Joseph Bresee | CDC | Co-Investigator | | W. Abdullah Brooks | ICDDR,B | Co-Investigator | | Margaret DiVita | University at Buffalo | Student Investigator | | Alicia Fry | CDC | Co-Investigator | | Stephen Luby | ICDDR,B | Co-Investigator | | Mustafizur Rahman | ICDDR,B | Co-Investigator | | Rashid Uz Zaman | ICDDR,B | Co-Investigator | |

# Description of the Research Project

##

## **Hypothesis to be Tested:**

Concisely list in order, the hypothesis to be tested and the Specific Aims of the proposed study. Provide the scientific basis of the hypothesis, critically examining the observations leading to the formulation of the hypothesis.

There is secondary transmission of influenza from index case-patients to household contacts in a rural setting.

Promotion of handwashing with soap will reduce secondary transmission of influenza from index cases to household contacts.

Risk factors for intrahousehold transmission of influenza in a rural setting include young age of index case or of household contact, active or passive smoking, crowding, and poor ventilation.

Exposure to an intensive handwashing education intervention will result in sustained improvement in handwashing behavior change.

Exposure to an intensive handwashing education intervention will result in a reduced risk of respiratory infections, diarrhea, and influenza.

## **Specific Aims:**

Describe the specific aims of the proposed study. State the specific parameters, biological functions/ rates/ processes that will be assessed by specific methods.

To measure the secondary attack ratio of influenza among household contacts of index cases with influenza, in a rural setting in Bangladesh

To test the efficacy of an intervention promoting handwashing with soap for prevention of intrahousehold transmission of influenza virus

To identify risk factors for intrahousehold transmission of influenza in a rural setting in Bangladesh

To compare handwashing behavior among households who were exposed to the intervention promoting hanwashing with soap to handwashing behavior among households who were not exposed to the intervention 5-6 months after enrollment

To measure the longitudinal prevalence of respiratory infections, diarrhea, and influenza among intervention and control households 5-6 months after enrollment.

## **Background of the Project including Preliminary Observations**

Describe the relevant background of the proposed study. Discuss the previous related works on the subject by citing specific references. Describe logically how the present hypothesis is supported by the relevant background observations including any preliminary results that may be available. Critically analyze available knowledge in the field of the proposed study and discuss the questions and gaps in the knowledge that need to be fulfilled to achieve the proposed goals. Provide scientific validity of the hypothesis on the basis of background information. If there is no sufficient information on the subject, indicate the need to develop new knowledge. Also include the significance and rationale of the proposed work by specifically discussing how these accomplishments will bring benefit to human health in relation to biomedical, social, and environmental perspectives.

The emergence of the highly pathogenic avian influenza A (H5N1) among humans throughout South and Southeast Asia and Eastern Europe[1] , and the potential for a new global pandemic of H5N1 or another influenza subtype, highlight the immediate need to identify risk factors for influenza transmission in low-income settings and to assess the efficacy of interventions to reduce the transmission of influenza viruses in these settings.

Influenza viruses spread from human-to-human via large respiratory droplets.[2] Transmission via large-particle respiratory droplets is believed to be mediated by close contact between infected and susceptible persons or contact with droplet-contaminated fomites.[2-3] Close contact between infected and susceptible persons may consist of skin-to-skin contact (e.g., via hands) or inhalation of respiratory droplets (e.g., due to talking, coughing, or sneezing by the infected person).[4] Airborne transmission, which is expected to result in transmission over long distances (>1 meter) and which would be mediated by ventilation, is believed to be uncommon.[4] Therefore, the greatest risk of transmission from personal contact comes from those people who are closest to an index case, such as contacts living in the same household.

The incubation period of the influenza virus is short, lasting typically 1 to 3 days. The infectious period for adults may begin 1 day prior to the onset of symptoms, and last until 5 days after symptoms begin. Children can be infectious for up to 7 days after symptom onset. The epidemiology of influenza has been well documented and understood in industrialized countries, but the data on influenza in developing countries is minimal. Globally, influenza epidemics occur annually, with clinical attack rates ranging from 10 to 20 percent in the general population, and more than 50 percent in closed populations, such as schools. Hospitalizations and deaths typically occur in high risk groups such as the elderly, very young, and the immuno-compromised. The death toll associated with annual epidemics of influenza is estimated to reach nearly 1 million people per year[5].

In a low-income setting such as Bangladesh, 16% of children aged less than 13 years with fever and cough (and who tested negative for dengue infection) were found to have influenza type A or B infection.[6] The first recognized human case of A(H5N1) influenza in Bangladesh occurred in 2008 in a child in Kamalapur, a densely populated urban slum in Dhaka, the capital city.[7] In order to develop rational prevention strategies, it is essential that we identify the relationship between demographic, behavioral, and environmental factors and influenza transmission among contacts of infected persons in low-income settings. Poor hand hygiene[8], crowding[9], and tobacco use[10] are commonplace in Bangladesh and thought to contribute to respiratory illness and outbreaks of respiratory illness (refs).[11] Only 14% of primary caregivers of young children in rural Bangladesh were observed to wash hands with soap before preparing or serving food and none washed hands before eating.[12] In another study with a substantially larger sample size, the proportion of primary caregivers observed to wash hands with soap was less than 1 percent.[13] In rural Mirzapur, crowding was shown to be a risk factor for viral pneumonia among children < 24 months of age.[9] Nationwide**,** in Bangladesh, 40% of adult males and 21% of adult females were estimated to be tobacco users in 2001.[14] Passive exposure to tobacco smoke has been implicated as a risk factor for respiratory illness among children.[10-11, 15] We do not fully understand the relevance of these factors for transmission of Influenza virus from ill persons to their household contacts.

In high-income countries, annual vaccination of high risk groups is the principal measure of prevention and control of influenza illness.[2] These measures are not available at scale in resource-poor settings, where the next influenza pandemic is expected to have devastating consequences. In such settings, therefore, it is imperative to assess the efficacy of non-pharmaceutical interventions to prevent the spread of influenza. Indeed, non-pharmaceutical interventions such as handwashing with soap are already recommended for prevention of influenza transmission (http://www.cdc.gov/flu/protect/preventing.htm). However, there is no published empirical evidence for the efficacy or effectiveness of handwashing with soap for prevention of influenza transmission in resource-poor settings. A recent meta-analysis done by Aiello et al found that most studies assessing the effectiveness of hand washing interventions upon infectious disease treated any respiratory illness as the outcome, not specifically influenza virus, and that the majority of the studies found took place in high-income settings (67%).[16] Moreover, a systematic review of the literature done by Jefferson et al shows that the majority of trials assessing the impact of hand washing on respiratory illness were done in day care or hospital settings.[8] Since the risk of transmission is likely high among household contacts of ill persons in low-income settings, it is crucial that such interventions are tested within households.

Given that much influenza transmission occurs among close contacts, the critical role that non-vaccine interventions will play in prevention of pandemic influenza transmission in resource-poor settings, and the high likelihood that the next influenza pandemic will greatly impact such a setting, we propose to conduct a multi-pronged study in Bangladesh.

**Our objectives are:**

To measure the secondary attack ratio of influenza among household contacts of influenza-infected persons in a rural setting in Bangladesh

To test the efficacy of a handwashing promotion intervention for prevention of intrahousehold transmission of influenza virus in a rural setting in Bangladesh

To investigate risk factors for secondary transmission of influenza from index case-patients to household contacts

Since Bangladesh has high rates of influenza illness and vaccination of this population is virtually non-existent, it represents an ideal setting for the proposed study. While several studies regarding handwashing and respiratory illness, including influenza, have been and will be carried out in urban slum areas of Dhaka, there is little information on the role of handwashing and other risk factors for influenza transmission within households in rural areas. About ¾ of the Bangladeshi population lives in rural areas[17] and contact with poultry, a risk factor for avian influenza, is substantially more common in rural communities. For this reason, and because the Jahurul Islam Medical College Hospital is a high-functioning participant in an ongoing human influenza surveillance project, we have chosen this site.

We also propose to complete a follow-up study of all households enrolled in the original study. The intervention given is particularly intensive in nature because we seek to establish whether maximal improvement in handwashing behavior will prevent secondary transmission of influenza. Luby and colleague have undertaken intensive handwashing promotion in their studies in Karachi, Pakistan on the efficacy of handwashing for the presevention of diarrhea and pneumonia. Despite weekly visits to intervention households to promote handwashing and to provide soap over an entire year, Luby et al. found little sustained behavior change among these households [18]. Moreover, there was no difference in the disease risk among children in intervention households, compared to those in control households who had not received the intervention. The intervention proposed for this study is even more intensive than that used in the Karachi studies because it seeks to reinforce benefits, overcome barriers, and motivate group-level behavior change. This proof of concept study not only hopes to find a difference in the rate of secondary transmission of influenza during the intervention, but also hopes to yield sustainable improvements in handwashing behavior change. Therefore, we propose to complete a follow-up study to answer the following questions:

Does exposure to the intervention in the primary intervention study result in sustained handwashing behavior change?

Does exposure to the intervention in the primary intervention study result in a reduced risk of respiratory infections, diarrhea, and influenza in intervention households compared to control households.?

The proposed study will hereafter be referred to as the Bangladesh Interruption of Secondary Transmission of Influenza study (BISTIS).

## **Research Design and Methods**

Describe in detail the methods and procedures that will be used to accomplish the objectives and specific aims of the project. Discuss the alternative methods that are available and justify the use of the method proposed in the study. Justify the scientific validity of the methodological approach (biomedical, social, or environmental) as an investigation tool to achieve the specific aims. Discuss the limitations and difficulties of the proposed procedures and sufficiently justify the use of them. Discuss the ethical issues related to biomedical and social research for employing special procedures, such as invasive procedures in sick children, use of isotopes or any other hazardous materials, or social questionnaires relating to individual privacy. Point out safety procedures to be observed for protection of individuals during any situations or materials that may be injurious to human health. The methodology section should be sufficiently descriptive to allow the reviewers to make valid and unambiguous assessment of the project.

BISTIS builds on hospital-based surveillance for Influenza virus infection, which is ongoing in hospitals around Bangladesh, as part of the Hospital-based Influenza Surveillance (HBIS) and Surveillance for the Epidemiology of Influenza in Bangladesh (SEIB) projects. We intend to recruit patients identified at the Jahurul Islam Medical College Hospital in Kishoregonj, Bangladesh, where both HBIS and SEIB are in place. In this hospital 80% of all the patients who present with influenza-like illness (ILI) to the outpatient departments of Medicine and Pediatrics are from three upazillas of Kishorgonj district: Bajitpur, Kuliar char and Kotiadi. The distances of these three upazillas are within 30 minutes travel time from Jahurul Islam Medical College Hospital (one way) and hence these upazillas will serve as the primary catchment areas for BISTIS. The table below illustrates the number of ILI cases identified through HBIS and SEIB study at Jahurul Islam Medical College Hospital in 2008 and also the number and proportion among them who were tested PCR positive for influenza virus.


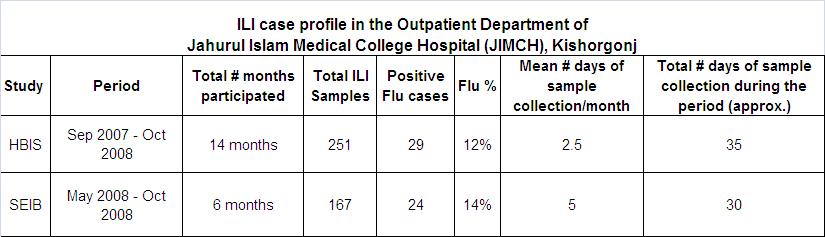


**N.B. most influenza-positive specimens were collected between May and September in 2007 and 2008**

We will also enroll patients who present to two local upazilla health complexes (UHCs), one in Bajitpur and one it Kuliar Char. These local health complexes see numerous patients a day from the rural areas surrounding the clinic. Patients who present to these clinics are more likely to have symptom onset within 24 hours of presentation then those patients who seek care at JIMCH, since patients may only want to go to the hospital if their illness has been severe and prolonged over several days. There has been published evidence that interventions on handwashing practices will only prevent influenza transmission in a household setting if the intervention is delivered within 36 hours of symptom onset [19]; therefore, the UHC sites are appropriate for enrollment of patients for BISTIS in addition to enrollment at JIMCH.

We will also enroll patients who present to pharmacies and other local health care providers in Bajitpur and Kuliarchar. During the enrollment in the early 2010 season, we have found that many individuals seeking care at the UHCs are waiting longer than 24 hours after symptom onset to visit the UHC. We hypothesize that individuals with symptom onset within 24 hours may seek early treatment at local pharmacies and other local health care providers, and only then visit the UHCs or JIMCH should their symptoms persist. By the time many individuals seek further treatment, there may be secondary cases within the bari. With secondary transmission occurring at the bari level prior to the individual seeking care at our enrollment sites, this greatly weakens our ability to determine whether our intervention does in fact prevent influenza transmission within the bari. Therefore, we propose to enroll index case-patients at local pharmacies and other local health care providers, in addition to the UHCs and JIMCH.

The medical officers will identify pharmacies and other local health care providers willing to allow placement of one of our FRAs for several hours on each working day. We will place one FRA at each participating site. The pharmacist or health care provider will identify individuals with fever, and will refer that person to our FRA. The FRA will then complete the Hospital Check List Form (Appedix 13) to determine the eligibitlity of the individual. When the FRA finds a person who meets the eligibility criteria, he/she will contact an MO by phone to confirm that the person does in fact meet eligibility. Once the MO confirms the eligibility, the FRA will obtain informed consent for a sample collection. If the person consents, the FRA should then contact the MT or MO to let them know that the participant requires specimen collection. The MT or MO can either meet the FRA and participant at the pharmacy for the specimen collection, or the FRA can travel to the bari with the participant and the MT or MO can meet them there, which ever is more convenient. The sample collection for the participant enrolled at the pharmacy or other local health care provider should take place no later than 24 hours after enrollment, although a concerted effort should be made to collect the sample the same day as enrollment.

**Specific Aim 1: To measure the secondary attack ratio (SAR) of influenza viruses among household contacts of index cases with influenza, in a rural setting in Bangladesh**

***Methods for Specific Aim 1***

In the ongoing SEIB and HBIS projects, patients at Jahurul Islam Medical College Hospital with influenza-like illness (ILI) or severe acute respiratory illness (SARI) and who are seen as outpatients are requested to provide nasal and oropharyngeal swab specimens for testing for influenza virus. The sample collections occur on 6 days per month in the outpatient population.

ILI and SARI are currently defined as follows:

Influenza-like illness (ILI): any patient presenting with history of fever and either cough *or* sore throat within the previous 7 days

Severe acute respiratory illness (SARI):

Patients > 5 years of age: hospitalized patient with acute lower respiratory tract illness consisting of fever

And cough or sore throat

And shortness of breath or difficulty breathing

Patients < 5 years of age: definition of pneumonia or severe pneumonia as per the Integrated Management of childhood Illness guidelines[20]

For BISTIS, we plan to daily identify patients meeting the following age-specific case definitions, and to request them to provide specimens (with the exception of Tuesdays when the hospital is closed) for the duration of the study.

The case definition for index case-patients is:

Persons, any age, with acute fever onset within 7 days preceding presentation to either JIMCH, the UHCs, the pharmacies, or other local health care providers.

Additional inclusion criteria for the proposed study are:

Return to home within 24 hours of presentation to Jahurul Islam Medical College Hospital, the UHCs, the pharmacies, or other local health care providers; i.e., the index case cannot be admitted at Jahurul Islam Medical College Hospital. If admitted, the patient would not be eligible for inclusion in this study.

At least two persons (in addition to the index case-patient) who intend to reside in the *bari* during the subsequent 20 days.

Residence within 2 hours travel time (one-way) from the Jahurul Islam Medical College Hospital or the UHCs

Patients who meet the case definition and additional inclusion criteria will hereafter be referred to as index case-patients.

In rural Bangladesh, homes are typically clustered into ***baris****,* with several homes in each *bari* (appendix 1 – figure and details about *baris*)*.* Typically, related individuals, with extended or joint family kinships, live in these homes and there is one head of the *bari* (usually the most elderly man). There is substantial contact between residents of different homes within a *bari*, with shared cooking spaces, play areas for children, toilets, and courtyards. Thus, most or all *bari* members are at risk for secondary transmission of influenza, irrespective of which specific home is occupied by an index case. For the purposes of this study, therefore, the household refers to the entire *bari* in which the index case-patient’s home sits. Hereafter, “household contact” refers to any member residing in the *bari*, apart from the index case-patient.

Within 4 hours of presentation to the Jahurul Islam Medical College Hospital, the UHCs, the pharmacies, or other local health care providers, the medical officer will approach patients who meet inclusion criteria in order to describe the study. The index case-patient or his/her guardian in the case of a child < 18 years old will be requested to provide informed consent for specimen collection for rapid testing for influenza (appendix 2 and 3 –consent for specimen collection and parental consent for specimen collection). Children between 7 and 17 years old will be requested to provide informed assent for specimen collection (appendix 4 – assent for specimen collection).

*Specimen collection and processing*

A trained study physician will procure a nasal swabfrom consenting index case-patients meeting the inclusion criteria above using a standardized method. This specimen will be used for a rapid antigen detection test (QuickVue® Influenza A + B). After the results of the QuickVue test are known, for patients ages 5 years and older a second nasal swab and an oropharyngeal swab will be take and both will be placed into a single tube containing viral transport media (VTM). The VTM will be kept at 4°C. All VTM will be transported to the ICDDR,B virology laboratory in Dhaka on a weekly basis. At the ICDDR,B virology laboratory, RT-PCR testing for Influenza A (H1N1), Influenza A ( H3N2), Novel Influenza A (H1N1) and Influenza B will be carried out. If Influenza A H1N1 and , novel H1N1, and A H3N2 are both all negative, RT-PCR testing for Influenza A H5N1 will be performed. The QuickVue® test materialswill be discarded using appropriate infection control procedures.

*Enumeration of bari contacts and questionnaire administration*

For all index case patients who meet the age-specific case defintions we will request that a field research assistant (FRA) accompany the index case-patient to the household. Once at the *bari*, the FRA will verify whether the *bari* meets the following inclusion criteria using the Bari Eligibility Form (Appendix 6):

Inclusion criteria for *baris* of index case-patients are:

At least two persons (in addition to the index case-patient) who intend to reside in the *bari* during the subsequent 20 days. Residence in the *bari* refers to sleeping in the *bari* at night, even if the individual works outside the *bari* during the day.

Written informed consent for the following study components from the head of *bari* on behalf of all *bari* residents (Appendix 5 – household consent) for each *bari* enrolled in the study

Questionnaire administration

Rapid observations of the household

Illness tracking

Random assignment to intervention or control group

The FRA will draw the bari (appendix 7: Drawing of Bari Form), recording the following items in the bari: housing structures, water source, toilet facilities, cooking areas, handwashing station (present before intervention), intervention handwashing station (to be filled in for intervention households only), the entrance to the bari, the entrance of the housing structures, and households.

The FRA will enumerate all eligible *bari* contacts (appendix 8 – enumeration sheet) and carry out assessments of ventilation, crowding, indoor air pollution, smoking, and socioeconomic status (appendix 9– household questionnaire/ observations . These measures are described in more detail below. The questionnaire will be administered to each household within the index case-patient’s bari. For the purposes of our study, we will define a “household” as individuals who share the same cooking pot. Some questions will be specific to each household or housing structure within the *bari,* in which case we will pose those questions to the male or female head of that particular household and structure. Housing structure is defined as where individuals sleep.

*Illness tracking among bari contacts*

Illness tracking will be carried out on each day for 10 days until after resolution of the index case-patient’s symptoms. Resolution will be defined as the lack of fever, cough, and sore throat for two consectutive days during the FRA’s daily illness tracking visit. Thus, if the index case-patient’s illness resolves on day 4 after enrollment, illness tracking will continue until day 14 after enrollment. The FRA will visit the patient’s home and record information regarding the presence or absence of ILI and SARI symptoms in each household contact using an individual illness tracking form (appendix 10– illness tracking form).

The case definitions for household contacts are:

Any *bari* resident with fever during the follow up visits of the index cases by the FRAs.

If any household contact meets the case definition, that person will be eligible for testing for influenza by the medical officer or technologist. If any household contact reports the following danger signs, the FRA will refer him/her immediately to the Jahurul Islam Medical College Hospital:

Persons > 5 years old: Cyanosis, severe respiratory distress, convulsions, altered mental status

Persons < 5 years old: Chest in-drawing, lethargy, cyanosis, inability to drink, convulsions

The FRA will pay for transport of the ill household contact and one or two accompanying family members to Jahurul Islam Medical College Hospital. S/he will provide a card indicating the ill household contact’s participation in BISTIS, so that when s/he arrives at the hospital, the BISTIS study physician will be contacted. Once the patient is deemed clinically stable by physicians at JIMCH, the BISTIS study physician will verify that the ill household contact meets the age-specific case definition and will request him/her to provide written informed consent for specimen collection (appendix 2 – specimen collection from household contact for adult > 18 years old). If the ill household contact is a child < 18 years old, informed consent will be obtained from the parent or guardian (appendix 3 – specimen collection from household contact for child < 18 years old). Children between 7 and 17 years old will be requested to provide informed assent for specimen collection (appendix 4 – assent for specimen collection). The medical technologists will collect information on the type of visit (secondary or follow up) and the date and time of the specimen collection on the specimen collection form (Appendix 11: Secondary/Follow Up Specimen Collection Form). We will store samples for 20 year for future testing of respiratory illnesses other than influenza; consent for specimen storage will be included in the specimen collection consent/assent form. If informed consent for specimen collection is provided, the study physician will collect nasal and oropharyngeal swabs from the ill household contact and place them in a single VTM vial. As with other specimens collected under BISTIS at the Jahurul Islam Hospital, the VTM vial will be kept at 4°C. Of course, it is the choice of the patient or guardian (in the case of an ill household contact < 18 years old) to decide whether or not to comply with the FRA’s recommendation to go to Jahurul Islam Medical College Hospital. In the event that the ill individual does not comply with the recommendation, the FRA will notify the BISTIS study physician. The physician will visit the *bari* within 24 hours to assess the ill household contact and provide further recommendations regarding treatment.

If a household contact meets the case definition and does NOT have any danger signs, the FRA will contact the MO to travel to the bari to obtain written informed consent for specimen collection (appendix 2– Adult Consent Form: Specimen Collection, Appendix 3—Parental Guardian Consent Form: Specimen Collection, Appendix 4—Child Assent Form: Specimen Collection). The medical officer, who will visit the home no later than the following day in order to collect nasal and oropharyngeal swabs from the ill household contact. She will immediately place both swabs into VTM, which will then be placed into a cool box, containing ice and a thermometer to ensure temperatures < 4^0^C.

All specimens collected in the field will be placed in a liquid nitrogen dewer and will be transported to the ICDDR,B laboratory within two weeks of sample collection. At the ICDDR,B virology laboratory, all specimens for household contacts in baris where the index case patient tested QuickVue positive will be tested using RT-PCR for Influenza A (H1N1), A (H3N2), novel Influenza A (H1N1) and Influenza B (and A (H5N1) if appropriate). For household contacts of index case patients who tested QuickVue negative, the specimens will only be rt-PCR tested for influenza if the index case’s rt-PCR test is positive for influenza.

Illness tracking among household contacts will continue in each household until the 10^th^ full day following the resolution of the index case-patient’s symptoms, irrespective of whether any household contact develops illness or not.

**Specific Aim 2: To test the efficacy of a handwashing promotion intervention for prevention of intrahousehold transmission of influenza virus**

***Methods for Specific Aim 2***

To address this specific aim, we will conduct a randomized controlled trial. Households of index case-patients with influenza-like illness who are not admitted at Jahurul Islam Medical College Hospital or the UHCs will be randomized to the intervention group or the routine practices group. The two groups will be defined as:

Intervention Households: intensive promotion of handwashing with soap, and provision of facilitating tools, to the index-case-patient and all available household contacts

Routine practices Households: continuation of the household’s usual handwashing and respiratory hygiene practices

Randomization

We will carry out block randomization of households of index case-patients to ensure random and even assignment to the intervention group or the routine practices group. Using a block size of four, the US-based co-Principal Investigator (PKR) will generate a list of random assignments to the routine practice or intervention groups. There will be two randomization sheets, one for the index case patients who test QuickVue positive and one for the index case patient’s who test QuickVue negative, to ensure equal assignment of the intervention among households with known positive influenza cases. The lists will be shared with the ICDDR,B PI and the FROs based at JIMCH. The FRO will consult the assignment list in order to determine whether the next enrolled household should be allocated to the intervention group or the routine practices group.

Delivery of the intervention

After the head of the bari has signed the informed consent for bari enrollment in the study, the FRA will telephone the FRO and tell the FRO that the bari has been enrolled. The FRO will consult the appropriate randomization sheet, and, if the bari is assigned to the intervention group, the FRO will assign an FIS to visit the household later in the day, after the FRA has finished with data collection.

For promotion of handwashing with soap to intervention households, the FIS will be trained to carry out a structured intervention that will follow constructs of Social Cognitive Theory (SCT)[21]. SCT addresses the reciprocal interaction between individuals, their environment, and health behaviors. Given that intervention will occur at the *bari* level, group-mediated constructs such as observational learning and reinforcements are highly relevant. We have included below a table of the major constructs of SCT, their definitions, and application of the constructs within this intervention.

In addition, the FIS will work with the household to identify the most convenient place to wash hands with soap and water. If no water container, or sink is present, the FIS will provide a water container that has a spout for running water

FISs will visit the intervention households on a daily basis for 10 days after the resolution of the index case-patient’s illness in order to encourage handwashing with soap at the recommended times. The FIS and FRA will coordinate their visits to the household so that they do not arrive at the same time. This will decrease the possibility of observer bias by the FRA should s/he see the intervention being implemented in the household while s/he is tracking illness symptoms. S/he will also note daily whether soap and water are available at the convenient handwashing station and, if not, s/he will again take necessary steps to assist the family with complying with a fully stocked handwashing station. The FIS will track both compliance with maintenance of a fully stocked handwashing station and the provision of additional soap or a tippy tap to the home. (Appendix 12 – facilitating tools tracking).

Routine practices households will also be exposed to the intervention, but only upon completion of the study. An FIS will visit the routine practices household around 6 months after the completion of the study in order to encourage handwashing with soap at the recommended times, andto provide a water container as needed.

Blinding

Due to the physical nature of the intervention and no feasible placebo control, we will not be able to blind participants or FRAs during illness tracking. However, FRAs will be blinded to the intervention assignment until after the first day’s data collection. We accept this limitation in order to achieve the most intensive handwashing intervention possible.

Detection of outcome of interest

As noted above, under Specific Aim 1, an FRA will visit the home of the index case-patient daily for 10 days after the resolution of the index case-patient’s symptoms in order to record age-specific case defining symptoms. When a household contact meets the age-specific case definition, nasal and oropharyngeal swabs will be collected for RT-PCR testing for Influenza A and Influenza B viruses at ICDDR,B.

**Specific aim 3: To identify risk factors, other than handwashing with soap, for intrahousehold transmission of influenza in a rural setting in Bangladesh**

***Methods for Specific Aim 3***

To address Specific Aim 3, we will conduct a nested cohort study to assess risk factors for intrahousehold transmission of influenza viruses. Here, the cohort under investigation is the routine practices group, as defined under Specific Aim 2. All data required to address this Specific Aim 3 will have been collected as part of the data collection described above under Specific Aim 1.

A case will be defined as: RT-PCR confirmed Influenza virus infection (A or B) in a household contact of an RT-PCR confirmed Influenza virus infection (A or B) index case-patient during 10 days of follow-up after resolution of the index case-patient’s symptoms

**Specific aim 4: To assess whether exposure to the BISTIS intervention results in sustained improvements in handwashing behavior.**

***Methods for Specific Aim 4***

To address Specific Aim 4, we will visit each bari that was enrolled in the intervention study 4 - 7 months after illness tracking is complete. Baris were told at the time of enrollment into the primary BISTIS study that field workers would be visiting again in several months for further data collection. An FRA will visit the bari and explain the objectives and methods of the follow-up study and request written voluntary informed consent (Appendix 16: BISTIS Follow Up Study Enrollment Consent Form, Household/Bari).

After consent is taken, at the first visit, the FRA will measure handwashing behavior at the bari. We will complete a structured observation of the bari’s common handwashing behaviors. This observation will last for one and a half hours. We will record information on handwashing opportunities, such as after defecation or before preparing meals, and hand washing behaviors. Handwashing behavior will be also measured by the following methods (Appendix 18: BISTIS Follow Up Survey Form) :

The presence of a handwashing station that has both soap AND water

The presence of any soap available in the bari, available within one minute of fieldworker’s request to see the soap

Demonstration of use of soap to wash hands after hypothetical respiratory secretions contact event

For baris who were randomized to the intervention group, the fieldworker will assess whether cue cards provided as part of the intervention are visible in the bari and, if not visible, available within one minute of the fieldworker’s request to see them

The FRA will return to the bari 4-6 weeks later and collect the same information. This repeated collection will help us to determine if there is any reactivity to our follow-up visit in changing handwashing behavior.

Two to three months after the initial follow-up visit, the FRA will return to the bari and once again collect the same information on the handwashing behavior but will also provide SmartSoap to the bari. A SmartSoap is a bar of soap with a motion sensor embedded in the soap that will allow us to measure the number of times the soap is used per day per capita. The FRA will collect the SmartSoap three days later. Data from the SmartSoap will be used to calculate the number of soap use events in the bari. In total an FRA will visit the household a total of four times, three visits for data collection and one visit to collect the SmartSoap.

The FRA will use Appendix 19: Follow Up Soap Tracking Form to record information on the soap that is given to the bari, such as the date the soap was given, and the location the soap was placed within the bari.

**Specific aim 5: To assess if exposure to the BISTIS intervention results in a reduced risk of respiratory infections, diarrhea, and influenza.**

***Methods for Specific Aim 5***

The measurements of the health outcomes will be done in two different ways. At the first visit, after the handwashing behavior information is collected, the FRA will record whether each member of the bari has had symptoms of fever, cough, sore throat, difficulty breathing, or diarrhea in the previous 48 hours (Appendix 20: BISTIS Follow Up Visit Illness Tracking Form). At this time the FRA will ask about known danger signs of respiratory illness or diarrhoea, and if any member has these signs, the FRA will refer him/her to JIMCH for care.

Symptoms of respiratory illness and diarrheoa will be collected at each subsequent visit.

At the third visit, in April 2010, the FRA will record mobile phone numbers of two or three bari members. The FRA will identify a key informant, who will be able to provide information regarding fever in any bari member. The FRA will phone the bari once each week during the influenza season and speak with the key informant once per week to assess whether any bari member has had fever during the previous 3 days (Appendix 21a: BISTIS Follow Up Study Phone Call Illness Tracking Form: Ages ≥ 5 Years Old). If any member is reported to have a fever, we will dispatch an MO or lab/medical technician to the bari to obtain nasopharyngeal swab from that member for flu testing by PCR. A case will be defined as: RT-PCR confirmed Influenza virus infection (A or B) (Appendix 11: Secondary/Follow Up Case Specimen Collection Form). The medical technologist will obtain informed consent/assent for specimen collection for all follow up contacts (appendix 2– Adult Consent Form: Specimen Collection, Appendix 3—Parental Guardian Consent Form: Specimen Collection, Appendix 4—Child Assent Form: Specimen Collection).

The FRA will use Appendix 17: Follow Up Bari Eligibility Form to track the eligibility of the bari for the follow up study, as well as to track any drop outs from the follow up phase.

# Measures of interest

The following factors are potential risk factors for intrahousehold transmission of influenza. We have excluded here routine demographic factors, such as age and number of years of education since those will be collected directly using a structured questionnaires and the household contact enumeration sheet (appendix 8 – enumeration of household contacts, appendix 9– household questionnaire/ observations).

**Socioeconomic status**: We will construct an SES index using principal components analysis based on previously described methods.[22] Asset scores from principal components analysis will allow us to assign households to SES quartiles.

**Crowding**: We will calculate a crowding index based on the number of persons residing in a structured divided by the number of rooms (excluding the kitchen and bathroom) available in that structure. Since, in *baris*, there are multiple structures and a given individual typically sleeps and lives within only one of those structures, the crowding index for each household contact will take into account the total number of persons living in his/her structure and the number of rooms within that structure.

**Ventilation**: Ventilation of the home will be assessed by a tool used elsewhere in Bangladesh. In each structure in the *bari*, we will identify the kitchen space and the main sleeping space of the index case. In each of those, we will count the number of walls, the availability of doors and windows, and the presence of spaces between walls and ceilings, and spaces between walls and floors. We will develop a scale using this information to assign kitchen spaces and sleeping spaces to strata of ventilation.

**Indoor air pollution**: We will use the UCB Particle Monitor, which has been validated and used in research studies in India, Nepal, and elsewhere in sub-Saharan Africa and Latin America.[23-24] The UCB Particle Monitor measures concentrations of particulate matter < 50 microns (PM50). The monitor will be placed for 24 hours in the sleeping space of the index case-patient . Placement of the monitor will occur within 72 hours of enrollment into the study to ensure that we measure particulate matter concentrations when influenza transmission is most likely to occur. (Appendix 13: Air Monitor Form). For baris that contain a fully enclosed cooking area (four full walls with an entrance) we will also collect AQM data within the cooking area.

**Respiratory hygiene**: We will inquire about whether the respondent sneezes or coughs directly into her hands, or into a kerchief or other cloth. In addition, we will inquire about whether the respondent washes hands after they come into contact with nasal secretions. This information is self-reported.

**Active smoking**: Smoking related behaviors will be elicited using questions from the Global Adult Tobacco Survey (GATS) and the Global Youth Tobacco Survey (GYTS), which are validated tools being utilized internationally in 15 countries, as part of the Bloomberg Global Initiative to Reduce Tobacco Use. We will inquire about whether the individual used any smoked tobacco products within the previous 30 days and determine where the individual ever used those products inside the home.

**Passive exposure to smoking**: Since we will collect information about direct smoking by all household contacts, we will be able to determine whether passive exposure to smoking occurs within each structure in the *bari*.

**Availability of soap and water at a handwashing station**: We will identify the location, if any, that the household primarily uses to wash hands. We will inspect whether any cleansing agent (e.g., soap, detergent, mud) and water are available at that location.

Application of Social Cognitive Theory constructs to handwashing promotion for prevention of secondary transmission of influenza[21]

| **Construct** | **Definition** | **Example of application in proposed intervention** |
| --- | --- | --- |
| Environment | Factors physically external to the person | The FIS will ensure that the environment facilitates handwashing behavior by ensuring, on a daily basis, that the tools of soap and water are present at a convenient handwashing station in the *bari.* |
| Situation | Person’s perception of the environment | The FIS will strive to understand perceived barriers to handwashing with soap and influenza prevention among *bari* members, and help the *bari* members to overcome these barriers. Expected barriers include perceptions of soap affordability, inability to keep soap at an outdoor handwashing station for fear of theft by humans or crows, lack of time to wash hands during busy household or child-rearing tasks, and perception that influenza / respiratory illness transmission within household members is inevitable. |
| Behavioral capability | Knowledge and skill to perform a given behavior | The FIS will demonstrate, on a daily basis, the proper way to wash hands with soap. She will remind all *bari* members daily about the critical times to wash hands, as outlined below. |
| Expectations | Anticipatory outcomes of a behavior | The FIS will reinforce to *bari* members the need to prevent influenza transmission, and the role that handwashing is expected to play in preventing influenza transmission. |
| Expectancies | The values that the person places on a given outcome; incentives | The FIS will assess the positive and negative expectancies, as perceived by *bari* members. She will emphasize the positive potential health, educational, and economic consequences of preventing influenza transmission to *bari* members, especially the young, the elderly, and those working outside the home |
| Observational learning | Behavioral acquisition that occurs by watching the actions and outcomes of others’ behavior | The FIS will demonstrate on a daily basis, if needed, the appropriate way to wash hands with soap following critical times. More importantly, she will engage key *bari* members, such as the head of the *bari,* mothers, and school-aged children to act as models of good handwashing behavior in order to provide vicarious reinforcement to those under their sphere of influence. |
| Reinforcements | Responses to a person’s behavior that increase or decrease the likelihood of reoccurrence | The FIS will provide direct reinforcement by complimenting, on a daily basis, the maintenance of a designated handwashing station fully stocked with water and soap, and the demonstration of appropriate handwashing with soap by *bari* members. She will engage children and mothers to provide direct reinforcements to fellow *bari* members in her absence. |
| Self-efficacy | The person’s confidence in performing a particular behavior and in overcoming barriers to that behavior | By engaging *bari* members in active learning of handwashing with soap during her daily visits, the FIS will encourage self-efficacy. In addition, ensuring the availability of a fully stocked convenient handwashing station will promote self-efficacy. |
| Reciprocal determinism | The dynamic interaction of the person, behavior, and the environment in which the behavior is performed | The FIS will highlight, on a daily basis, the positive steps being taken by individuals and the *bari* as a whole to move all *bari* members towards better handwashing behavior. She will provide positive reinforcement directly and encourage *bari* members themselves to provide mutual reinforcement in order to achieve the common good of improved handwashing behavior. The FIS will ensure that, on a daily basis, the facilitating tools necessary to wash hands will be in place. |

**These constructs and definitions are verbatim from Glanz et al, Health Behavior and Health Education: Theory, Research, and Practice, 3^rd^ edition.[21]**

Critical times to wash hands with soap are:

After coughing or sneezing

After cleaning one’s nose or the nose of a child

After defecation

After cleaning a child who has defecated

Before preparing food

Before eating

Facilitating tools for handwashing are:

A designated handwashing station at a convenient location for most or all *bari* members

Soap available daily at the handwashing station

Water available daily at the handwashing station

# Laboratory methods

Nasal swab and oropharyngeal swab specimens will be collected using methods already in place at the Jahurul Islam Hospital for HBIS. Compared to nasopharyngeal washes, nasal swabs have been shown to have sensitivity and specificity of 91% and 100% in detecting influenza among children 2 weeks to 15 years old in a small study from Finland.[25] Also, among children < 18 years (median age 1.1 year) nasal and throat swab pairs performed similarly (92% sensitivity) to nasopharyngeal washes in identifying influenza A virus.[26] Among adults, throat swabs were less sensitive in identifying influenza than nasopharyngeal aspirates, when viral culture was the gold standard (47% sensitivity).[27] Among children < 18 years, throat swabs were less sensitive (83%) than nasopharyngeal swab for detecting respiratory viruses with nucleic acid amplification.[28] There is no published data on the yield of nasal swabs or nasal and throat swab pairs compared to naspharyngeal wash or swab for seasonal influenza detection in persons older than 15 years old. Oropharyngeal swabs are preferred over nasal or nasophayngeal swab for detection of human H5N1 infection.[29] Nasal and throat swabs are used in HBIS due to increased acceptability by patients and less technical expertise to perform compared to nasopharyngeal washes or swabs, and increased likelihood of detecting all influenza viruses (including A(H5N1).

From index case-patients, a nasal swab for testing using the QuickVue® (Quidel® Corporation). The QuickVue® uses monoclonal antibodies for direct detection of Influenza A and B and can be used with nasal swabs, nasopharyngeal swabs, nasal washes, or nasal aspirates. The QuickVue test has been shown to have a sensitivity of 78% when using specimens collects with nasal swabs; the specificity has been shown to be 97 to 98%[30]. During the influenza season, QuickVue® has a positive predictive value of 94%, when compared with RT-PCR, for detection of influenza for residents of the Kamalapur slum in Dhaka, Bangladesh (WA Brooks, personal communication). Since the PPV of QuickVue® is estimated to be so high, we are not adjusting the required sample size of index case-patients in order to allow for false positive QuickVue® tests.

After the QuickVue test, another nasal swab and an oropharyngeal swab will be collected from index case-patients and immediately placed into VTM. The VTM will be placed in a cool box (4⁰C) and transferred to a liquid nitrogen dewer at the end of the day for eventual transport to ICDDR,B for RT-PCR testing.

From ill household contacts meeting the case definition, a nasal swab and an oropharyngeal swab will be collected and placed into VTM. This will occur for ill contacts during follow up as well. The VTM will be placed immediately in a cool box with ice (4⁰C). The VTM will then be placed in a liquid nitrogen dewer at the end of the day for eventual transport to the ICDDR,B virology laboratory in Dhaka for RT-PCR testing. Samples will also be stored for future testing of non-influenza respiratory illnesses.

At ICDDR,B, RT-PCR will be done to detect Influenza A (H1N1), novel Influenza A (H1N1) Influenza A (H3N2), Influenza B, and Influenza A (H5N1), with appropriate using standardized laboratory methods already in place.[31]

**Timeline:**

Updated Timeline:

| **Activities** | **2009** | | | **2010** | | | | | | | | | | | | **2011** | | | | | | |
| --- | --- | --- | --- | --- | --- | --- | --- | --- | --- | --- | --- | --- | --- | --- | --- | --- | --- | --- | --- | --- | --- | --- |
|  | **10** | **11** | **12** | **1** | **2** | **3** | **4** | **5** | **6** | **7** | **8** | **9** | **10** | **11** | **12** | **1** | **2** | **3** | **4** | **5** | **6** | **7** |
| Update protocol to include follow-up phase |  |  |  |  |  |  |  |  |  |  |  |  |  |  |  |  |  |  |  |  |  |  |
| Development of follow-up phaseforms, budget |  |  |  |  |  |  |  |  |  |  |  |  |  |  |  |  |  |  |  |  |  |  |
| RRC/ERC Submission |  |  |  |  |  |  |  |  |  |  |  |  |  |  |  |  |  |  |  |  |  |  |
| RRC/ERC Approval |  |  |  |  |  |  |  |  |  |  |  |  |  |  |  |  |  |  |  |  |  |  |
| Data Collection Intervention Study First Season |  |  |  |  |  |  |  |  |  |  |  |  |  |  |  |  |  |  |  |  |  |  |
| Training Staff: Follow Up Phase |  |  |  |  |  |  |  |  |  |  |  |  |  |  |  |  |  |  |  |  |  |  |
| Data Collection Follow Up Phase |  |  |  |  |  |  |  |  |  |  |  |  |  |  |  |  |  |  |  |  |  |  |
| Training Staff Intervention Study: Second Season |  |  |  |  |  |  |  |  |  |  |  |  |  |  |  |  |  |  |  |  |  |  |
| Data Collection Intervention Study: Second Season |  |  |  |  |  |  |  |  |  |  |  |  |  |  |  |  |  |  |  |  |  |  |
| Data Analysis |  |  |  |  |  |  |  |  |  |  |  |  |  |  |  |  |  |  |  |  |  |  |
| Report Dissemination |  |  |  |  |  |  |  |  |  |  |  |  |  |  |  |  |  |  |  |  |  |  |
| Manuscript Preparation |  |  |  |  |  |  |  |  |  |  |  |  |  |  |  |  |  |  |  |  |  |  |

**Figure: Study flow diagram**

#
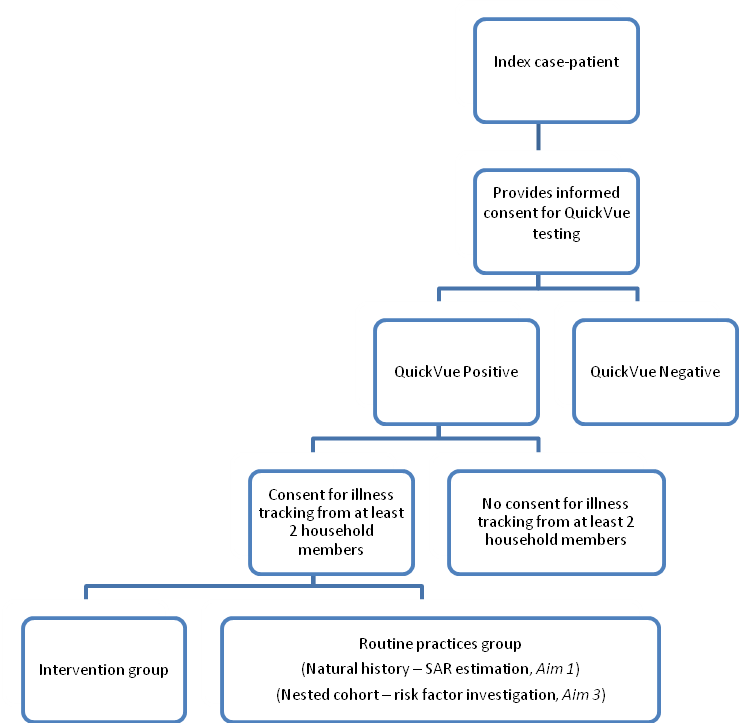


# *Sample Size Calculation and Outcome Variable(s)*

***Sample size calculation for Specific Aim 1***

This sample size calculation is based an estimated 10 potential contacts per *bari*. This is a gross estimate and is likely an underestimate of the number of persons residing in a *bari* in most cases, based on our experience. Literature from high-income countries, including Hong Kong, suggests that the secondary attack ratio of influenza or respiratory illness among household contacts of patients with influenza ranges between 8% and 17%.[32-33] For this sample size calculation, we allowed the largest population size allowable in Epi6 software, and assumed secondary attack ratios of 20% and 10% with varying acceptable error rates. The assumptions that we have made here with respect to the number of contacts per *bari* and the secondary attack ratios are underestimations; we have done so deliberately in order to yield the most conservative sample size requirements. These figures reflect the 95% confidence level.

| Population size | Expected secondary attack ratio | Acceptable error | # household contacts | # households if 10 contacts / hh |
| --- | --- | --- | --- | --- |
| 999,999 | 20% | 3% | 682 | 68 |
| 999,999 | 20% | 7% | 125 | 13 |
| 999,999 | 20% | 10% | 61 | 6 |
| 999,999 | 10% | 3% | 384 | 38 |
| 999,999 | 10% | 5% | 138 | 14 |
| 999,999 | 10% | 7% | 71 | 7 |

As detailed below, Specific Aim 2 entails promotion of handwashing with soap and improved respiratory hygiene in an intervention group of index case-patients and their households. Therefore, Specific Aim 1 will be answered based on the natural history of intrahousehold transmission, which will be evident in the routine practices group. If, as proposed for Specific Aim 2, we enroll 100 households in the routine practices group, we will be able to describe all of the scenarios in the table above.

***Sample size calculation for Specific Aim 2***

Sample size calculations for this controlled intervention study were based on the proportion of household contacts of index cases in each group that will become secondary cases. We assumed that standard deviations in the proportion of household contacts that become secondary cases will be the same in the intervention and routine practices groups. Sample sizes below were estimated at the 95% confidence level to achieve 80% power. We assume 10 contacts per household (excluding the index case-patient), as described under *Sample size calculation for Specific Aim 1*. In previous studies, the secondary attack ratio of respiratory illness or influenza has ranged from 8% to 17% among household contacts, with the 8% SAR for influenza detected in a pilot study in Hong Kong of non-pharmaceutical interventions.[32-34] Since we do not already have SAR data from Bangladesh, we performed sample size calculations based on estimates of 30%, 20%, and 10% SAR in the routine practices group, and relative risk reductions of 50% and 33%. We propose a design effect of 2.0 to account for clustering of secondary cases within households.[17, 35]

| **Routine practices group* (%)** | **Intervention group* (%)** | **Relative risk reduction (%)** | **# household contacts in each arm** | **# households in each arm** | **# households in both arms** | **# households in both arms after applying design effect of 2.0** |
| --- | --- | --- | --- | --- | --- | --- |
| 30 | 15 | 50 | 119 | 12 | 24 | 48 |
| 30 | 20 | 33 | 292 | 29 | 58 | 117 |
| 20 | 10 | 50 | 195 | 20 | 39 | 78 |
| 20 | 13.4 | 33 | 497 | 50 | 99 | 199 |
| 10 | 5 | 50 | 424 | 42 | 85 | 170 |
| 10 | 6.7 | 33 | 1093 | 109 | 219 | 437 |

*This is the secondary attack ratio among household contacts of index case-patients.

After taking into account a design effect of 2.0, we propose a minimum sample size of 39 households per study arm or a total of 78 households.

We expect that, in one influenza season at Jahurul Islam hospital, we will identify about 80 Influenza A and B cases per month. Assuming that there are three months during the 2009 influenza season (this appears to vary a bit from year to year), we anticipate a total of 240 Influenza cases detected during 2009 at Jahurul Islam Hospital. A number of these patients may not be the index case-patients in the home and, thus, would not be eligible for the study. Weighing sample size calculations with eligibility criteria and logistical realities, we estimate that we will be able to enroll a maximum sample size of 100 households per study arm or 200 households. This would allow us to detect all of the differences in the SAR shown above except for the last scenario.

***Sample size calculations for Specific Aim 3***

The sample size calculation for this cohort investigation is based on the proportion of index cases that are less than 5 years old (Tsolia, Vaccine, 2006). In that study set in Greece, 31% of siblings of children with influenza who were less than 5 years old developed respiratory illness, compared to 19% of siblings of children with influenza who were 5 years or older. We do not have published data on the relative burden of influenza on persons > 5 years old, compared to persons < 5 years old from Bangladesh. Thus, we assume that 33% of our index case-patients are < 5 years old. We expect 10 susceptible contacts per household, as outlined under *Sample size calculation for Specific Aim 1*, and assume that the secondary attack ratios of influenza among siblings and other household contacts are similar. We performed sample size calculations based on several estimated SARs, and relative risks of 2.0, 1.5, and 1.2 for each estimated SAR. These findings are expected to be significant at p=.05 with 80% power. To account for clustering of secondary transmission within households, we propose a design effect of 2.0.

Here, exposure is defined as the index case-patient’s age being < 5 years. Non-exposure is defined as the index case-patient’s age being > 5 years. As appropriate for cohort studies, we indicate the attack rate among exposed and the attack rate among non-exposed, and the resultant relative risk. The SAR indicates the secondary attack ratio of RT-PCR confirmed Influenza virus infection among all household contacts of the index case-patient.

| **SAR among non-exposed (%)** | **SAR among exposed (%)** | **Relative risk** | **# household contacts of index case-patients > 5 years old** | **# household contacts of index case-patients < 5 years old** | **Total # household contacts** | **Total # households** | **Total # households after applying design effect of 2.0** |
| --- | --- | --- | --- | --- | --- | --- | --- |
| 20 | 40 | 2 | 132 | 66 | 198 | 20 | 40 |
| 20 | 30 | 1.5 | 460 | 230 | 690 | 69 | 138 |
| 20 | 24 | 1.2 | 2576 | 1288 | 3864 | 386 | 773 |
| 30 | 60 | 2 | 72 | 36 | 108 | 11 | 22 |
| 30 | 45 | 1.5 | 260 | 130 | 390 | 39 | 78 |
| 30 | 36 | 1.2 | 1484 | 742 | 2226 | 223 | 445 |
| 40 | 80 | 2 | 40 | 20 | 60 | 6 | 12 |
| 40 | 60 | 1.5 | 160 | 80 | 240 | 24 | 48 |
| 40 | 48 | 1.2 | 940 | 470 | 1410 | 141 | 282 |

Therefore, if, as proposed for Specific Aim 2, we enroll 100 households in the routine practices arm, we will have sufficient sample size to detect the following at the 95% confidence level, with 80% power.

| **SAR among non-exposed (%)** | **SAR among exposed (%)** | **Relative risk** | **# household contacts of index case-patients > 5 years old** | **# household contacts of index case-patients < 5 years old** | **Total # household contacts** | **Total # households** | **Total # households after applying design effect of 2.0** |
| --- | --- | --- | --- | --- | --- | --- | --- |
| 20 | 40 | 2 | 132 | 66 | 198 | 20 | 40 |
| 20 | 30 | 1.5 | 460 | 230 | 690 | 69 | 138 |
| 30 | 60 | 2 | 72 | 36 | 108 | 11 | 22 |
| 30 | 45 | 1.5 | 260 | 130 | 390 | 39 | 78 |
| 40 | 80 | 2 | 40 | 20 | 60 | 6 | 12 |
| 40 | 60 | 1.5 | 160 | 80 | 240 | 24 | 48 |

***Facilities Available***

Describe the availability of physical facilities at the place where the study will be carried out. For clinical and laboratory-based studies, indicate the provision of hospital and other types of patient’s care facilities and adequate laboratory support. Point out the laboratory facilities and major equipment that will be required for the study. For field studies, describe the field area including its size, population, and means of communications.

Jahurul Islam Medical College Hospital, Kishorgonj has been selected for identifying the index cases for the study. This is a 400 bed non-government hospital located in the rural village of Bajitpur upazilla in district Kishorgonj. This is one of the high performing hospitals participating in the ongoing National Hospital Based Influenza Surveillance and Surveillance for Epidemiology of Influenza in Bangladesh.

# *Data Safety Monitoring Plan (DSMP)*

This protocol will be approved by the Research and Ethics Review Committees of the ICDDR,B and the Institutional Review Board (IRB) of the Centers for Disease Control and Prevention before its initiation. This study is expected to pose no more than minimal risk to participants. However, we appreciate the importance of ensuring the rights and safety of participants and thus will implement the following data safety monitoring plan.

Responsibility for data safety monitoring will rest with Dr. Eduardo Azziz-Baumgartner, a physician serving as the Principal Investigator responsible for overseeing data collection in the field. Serious adverse events (SAEs) directly related to the implementation of this study are not anticipated given the nature of the intervention (handwashing promotion) and the nature of study measures (questionnaire, rapid observation of the household environment, and tracking of illness symptoms among household contacts of index case-patients). However, since influenza can result in hospitalization and death, we will track and report all cases of hospitalization and death among contacts of participating households. The expected adverse event (AE) is dermatologic reaction to soap, which will be provided to intervention households.

All study staff involved in data collection and behavioral intervention in the field will be trained on this data safety monitoring plan. Data for all AEs and SAEs will be collected on standard case report forms. All SAEs, irrespective of whether they are related to the project, will be reported to Dr. Sohel within 24 hours of becoming aware of the event. Dr. Azziz-Baumgartner will report all SAEs to the Ethics Review Committee of the ICDDR,B, and Dr. Fry will report all SAEs to the CDC HRPO using established mechanisms for reporting of SAEs to the respective review committees. Reporting of SAEs to the respective review committees will occur within one week, or sooner, as required by the review committee at the time that the protocol is approved. Non-serious AE information will be captured and summarized during data analysis.

All data will be collected by trained research assistants using paper-based questionnaires, which will be maintained in a locked cabinet accessible only by research personnel. Data will be entered into password-protected databases. Entered data will be stripped of identifying information and will, instead, be coded using unique identification codes to ensure confidentiality. All data collection is expected to be completed within one year and we do not expect sufficient power to demonstrate significant differences between intervention and control groups in interim analyses. Although we intend to conduct data analysis to establish equivalence between intervention and control groups, we do not plan to conduct interim analysis of secondary attack ratios.

The Principal Investigators assume primary responsibility for monitoring data and safety aspects of this study, and will report on these issues in the annual applications to the respective review committees.

Per the Research Review Committee of the ICDDR,B, a data safety monitoring board is not indicated, given the nature of this study.

# *Data Analysis*

Describe plans for data analysis. Indicate whether data will be analyzed by the investigators themselves or by other professionals. Specify what statistical software packages will be used and if the study is blinded, when the code will be opened. For clinical trials, indicate if interim data analysis will be required to monitor further progress of the study.

***Data analysis for Specific Aim 1***

We will describe clinical features of index case-patients and household contacts with RT-PCR confirmed Influenza virus infection. Information from index case-patients with Quickvue®-positive but RT-PCR negative nasal swabs will be excluded from further data analysis.

The household demographics, socioeconomic status, self-reported respiratory hygiene, active and passive smoking status, ventilation, and availability of soap and water at handwashing stations will be described. We will report means and standard deviations of continuous variables and frequencies of categorical variables.

For Specific Aim 1, the following secondary attack ratios (SAR) will be the outcome of interest.

The SAR for RT-PCR confirmed influenza will be defined as follows:

# household contacts with RT-PCR confirmed Influenza virus infection detected during follow-up visit

Total # household contacts of index cases of RT-PCR confirmed Influenza virus infection

***Data analysis for Specific Aim 2***

Baseline characteristics of the intervention and routine practices groups will be compared using chi-squares for categorical variables, t-tests for normally distributed continuous variables, and the Wilcoxon rank sums test for non-normally distributed continuous variables.

Outcome of interest

For Specific Aim 2, the outcome of interest is the secondary attack ratio (SAR) of RT-PCR confirmed Influenza virus infection among household contacts of index case-patients in the intervention and the routine practices groups. As defined for Specific Aim 1, the SAR will be defined as

# household contacts with RT-PCR confirmed Influenza virus infection detected during follow-up visit

Total # household contacts of index cases of RT-PCR confirmed Influenza virus infection

The comparison of intervention and routine practices groups with respect to the SAR of RT-PCR confirmed Influenza virus infection among household contacts of index case-patients will be performed using multivariable linear regression, with adjustment for within-household correlation of illness. Intra-cluster correlation of illness will be estimated based on the between-cluster variance and the total variance of the SAR. We will use generalized estimating equations (GEE) to evaluate the difference in the SAR between intervention and control groups, given the possibility of household-level clustering of secondary illness. Primarily, analysis will be based on the intent to treat (i.e., irrespective of the handwashing practices following the administration of the intervention). Secondarily, we will also conduct per-protocol analysis based on the adherence of the intervention household to maintaining a fully stocked handwashing station with soap and water present during the majority of illness tracking days (i.e. until 10 days after the resolution of the index case-patient’s symptoms).

***Data analysis for Specific Aim 3***

Explanatory variables of interest

Since we will examine whether handwashing with soap is associated with reduced risk of intrahousehold transmission of influenza, we will focus on other potential explanatory factors in our analysis for Specific Aim 3. Specifically, we will examine whether cases differ from controls with respect to the following explanatory variables (described under *Measures of interest* below):

Age of the index child

Age of the secondary case-patient

Self-reported respiratory hygiene

Passive or active smoking among household contacts

Crowding

Ventilation

Socioeconomic status

To account for clustering of secondary illness within households, we will perform generalized estimating equations in order to test relationships between explanatory variables and case status. All variables associated with case status on univariate analysis, at a p-value < 0.10, will be tested in multivariate analysis. We will add variables to the multivariate model one-by-one, retaining only those variables that are significantly associated with case status after adjusting for potential confounders. Tests for interaction will also be conducted for possible effect modifiers.

***Data analysis for Specific Aim 4***

For specific aim 4, there will be two main outcomes of interest:

Rate of compliance with intervention

Baris that are found to have both soap and water present at the handwashing station will be considered compliant

Number of times soap is used per day per capita

We will replace all bars of soap actively being used in the bari with bars of soap containing motion sensors. The motion sensors will be set to begin data analysis at 11:59 pm on the day of placement. The data collector will retrieve the bars of soap with the motion sensor on the third day, after two full days (24-hour periods) of data collection have been completed. The motion sensors use accelerometer technology and measure movement in three dimensions: if the soap with motion sensor is still or moving slowly, its orientation with respect to gravity can be inferred. Algorithms for analysis of motion sensor data decode whether the bar of soap changes orientation in any of the three dimensions. Movements are classified as non-use when the soap is still, when motion does not involve two or more dimensions, or when motion takes place for <1 second. Movements are classified as genuine soap use when movement in two or three dimensions occurs for > 1 second. Soap movement data will be analyzed accordingly to estimate number of soap use events in each 24-hour period.

Differences in the rate of compliance at the first follow up visit in baris by intervention groups compared to control groups at follow up will be tested using chi-squared analysis. Differences in the mean number of times soap is used per day per capita between the intervention and control groups at follow up will be tested using t-tests..

***Data analysis for Specific Aim 5***

For specific aim 2, there will be three main outcomes of interest:

Longitudinal prevalence of respiratory illness (presence of fever, cough, sore throat, or difficulty breathing within last 48 hours)

Longitudinal prevalence of diarrhea (presence of diarrhea within the last 48 hours)

Cumulative incidence of influenza, as deteremined by RT-PCR

Longitudinal prevalence will be defined as:

Number of person-days with symptoms

Total number of person-days observed

The comparison of intervention and routine practices baris with respect to the longitudinal prevalence among household contacts will be performed using multivariable linear regression, with adjustment for within-household correlation of illness. Intra-cluster correlation of illness will be estimated based on the between-cluster variance and the total variance of the prevalence. We will use generalized estimating equations (GEE) to evaluate the difference in the prevalence between intervention and control groups at follow up, given the possibility of household-level clustering of illness.

Incidence of influenza, as determined by RT-PCR will be defined as:

# of participants with RT-PCR confirmed Influenza A or B during the follow up study

Total number of participants in the follow up study

We will use the same analysis plan as above, for the longitudinal prevalence data, here for incidence.

# *Ethical Assurance for Protection of Human Rights*

Describe in the space provided the justifications for conducting this research in human subjects. If the study needs observations on sick individuals, provide sufficient reasons for using them. Indicate how subject’s rights are protected and if there is any benefit or risk to each subject of the study.

#### Risks: The level of risk encountered by subjects in this study is no greater than minimal. Our research procedures include promotion of handwashing with soap, questionnaire administration, rapid observations of the household environment, and query regarding symptoms of influenza-like illness and severe acute respiratory illness. The research procedures that will be used represent routine data sources for conducting syndromic surveillance and measuring hygiene behaviors in both research studies and in program evaluations. Collection of nasal and oropharyngeal swab specimens is a routine clinical procedure used in medical practices worldwide and is associated with minor irritation or discomfort.

**Benefits:** Households in the intervention group will receive soap and will be exposed to the promotion of handwashing with soap. While the proposed study intends to assess the impact of this intervention on influenza transmission, previously published data supports the beneficial effects of promotion of handwashing with soap on diarrhea and respiratory illness risk, particularly among young children.[36] Although there is good evidence that handwashing with soap can prevent diarrhea and respiratory disease among children, the benefit of handwashing with soap for prevention of secondary transmission of influenza within households has not been previously studied in Bangladesh or similar low-income countries. [36-37] Because we recognize the benefit of handwashing with soap for childhood diarrhea and respiratory disease prevention, we propose to provide the standard practices group with handwashing promotion and two bars of soap upon conclusion of illness tracking (which will occur for 10 days following the resolution of the index case-patient’s symptoms).

Since antiviral medications are not routinely available for the treatment of Influenza in Bangladesh, identification of rapid test positive influenza will not change the physician’s clinical management of the illness. Therefore, rapid test results do NOT represent a benefit to the index case-patient and will not be reported to the treating physician.

#### Adverse events: The study involves interview and observation of index case-patients and their households. The observational and interview techniques are non-invasive and we do not anticipate serious adverse events; however all serious events will be monitored and the principal investigator will report on these issues in the annual applications to the respective review committees.

**Consent:** Participation in the study will be voluntary. This study involves the collection of information related to illness and hygiene practices in the household. These questions are generally not perceived to be of a sensitive nature in Bangladeshi culture. All household contacts of potential case-patients will be informed about the purposes and intent of the study, and the voluntary nature of their participation. Written informed consent will be obtained from each potential index case-patient for specimen collection; from each head of the bari, for household contact enumeration, questionnaire administration, rapid observations, and assignment to the intervention and control group; and from each ill household contact for specimen collection (Appendix x, y, and z). The consent forms will be translated into Bangla by a bilingual study staff person who holds a graduate degree. The interviewer will read the consent form aloud since the literacy rate among Bangladeshis remains very low. The potential participant’s questions and concerns will be addressed. If a potential participant agrees to take part, s/he will sign the consent form or provide a thumbprint in lieu of signature.

**Scientific and Ethical Review**: The protocol will require approval by the Research Review Committee and the Ethical Review Committee of ICDDR,B, the Human Research Protection Office at the Centers for Disease Control and Prevention, and the Institutional Review Board (IRB) of the University at Buffalo before its initiation. The committees will be notified of all protocol deviations.

**Confidentiality**: The consent form will contain the full names of the index case-patient, his/her Unique ID number, and a household Unique ID number. The questionnaire and rapid observation form will have the index case-patient’s first name, his/her Unique ID number, and the household Unique ID number. Each illness tracking form will contain the household Unique ID number, and the full name and respective Unique ID numbers of the household contact. All paper documents, once entered into a computer database, will be kept in a locked cabinet at Jahurul Islam Medical College Hospital, the partner institution located in Kishoregonj. The research investigator from ICDDR,B will have sole access to the locked cabinet.

## **Use of Animals**

Describe in the space provided the type and species of animals that will be used in the study. Justify with reasons the use of particular animal species in the experiment and the compliance of the animal ethical guidelines for conducting the proposed procedures.

Not applicable

# *Literature Cited*

Identify all cited references to published literature in the text by number in parentheses. List all cited references sequentially as they appear in the text. For unpublished references, provide complete information in the text and do not include them in the list of Literature Cited. There is no page limit for this section, however exercise judgment in assessing the “standard” length.

##

# *Dissemination and Use of Findings*

Describe explicitly the plans for disseminating the accomplished results. Describe what type of publication is anticipated: working papers, internal (institutional) publication, international publications, international conferences and agencies, workshops etc. Mention if the project is linked to the Government of the People’s Republic of Bangladesh through a training programme.

The findings of the study will be shared with the government and the participating hospital. The summary results will be published in the Health and Science Bulletin. The findings will be disseminated through presenting in the appropriate scientific conference. A manuscript will be prepared summarizing the principle measures of interest for publishing in an international peer reviewed scientific journal.

As per ICDDR,B data access policy the data will be made available to other researchers within 3 years of completion of the study.

# *Collaborative Arrangements*

Describe briefly if this study involves any scientific, administrative, fiscal, or programmatic arrangements with other national or international organizations or individuals. Indicate the nature and extent of collaboration and include a letter of agreement between the applicant or his/her organization and the collaborating organization.

This study will build upon the collaboration between ICDDR,B, University at Buffalo, and CDC, Atlanta.

# Biography of the Investigators

Give biographical data in the following table for key personnel including the Principal Investigator. Use a photocopy of this page for each investigator.

(Note: Biography of the external Investigators may, however, be submitted in the format as convenient to them)

**Biography of the Investigators**

| NAME  Ram, Pavani Kalluri | | POSITION TITLE  Assistant Professor | | |
| --- | --- | --- | --- | --- |
| eRA COMMONS USER NAME  n/a | |
| EDUCATION/TRAINING *(Begin with baccalaureate or other initial professional education, such as nursing, and include postdoctoral training.)* | | | | |
| INSTITUTION AND LOCATION | DEGREE  *(if applicable)* | | YEAR(s) | FIELD OF STUDY |
| Columbia College, Columbia University, New York, NY | AB | | 1989-1993 | Middle East Languages and Cultures |
| Mount Sinai School of Medicine, New York, NY | MD | | 1994-1998 | Medicine |
| Washington University School of Medicine, St. Louis, MO | -- | | 1998-2001 | Internal medicine |
| Centers for Disease Control and Prevention, Atlanta, GA | n/a | | 2001-2003 | Epidemiology |

**A. Positions & Honors**

**Positions and Employment**

7/01 – 7/03 Epidemic Intelligence Service, US Centers for Disease Control and Prevention, Atlanta, GA

7/03 – 7/05 Medical Epidemiologist, Foodborne and Diarrheal Diseases Branch, National Center for Infectious Diseases, US Centers for Disease Control and Prevention, Atlanta, GA

9/05 – 1/08 Research Assistant Professor, Department of Social and Preventive Medicine, University at Buffalo, State University of New York, Buffalo, NY

1/08 – present Assistant Professor, Department of Social and Preventive Medicine, University at Buffalo, State University of New York, Buffalo, NY

**Honors and Awards**

7/88 Governor’s Scholar, Public Issues, New Jersey

5/92 Dean Hawkes Memorial Prize for Excellence in Humanities, Columbia College

5/93 Magna Cum Laude, Columbia College, Columbia University

6/01 Teaching award, Washington University School of Medicine, in recognition of outstanding teaching of medical students on the Internal Medicine Clerkship

10/01 Crisis Response Award, US Public Health Service, for participation in post-9/11 activities

11/01 Crisis Response Award, US Public Health Service, for participation in response to anthrax bioterrorism

2/04 Achievement Medal, US Public Health Service, for exemplary performance of duty in implementation of the Safe Water System in Afghanistan

6/04 Unit Commendation, US Public Health Service, for rapid response to an outbreak of foodborne botulism

5/05 10^th^ Anniversary Hero, National Foundation for the Centers for Disease Control and Prevention

**B. Selected peer-reviewed publications**

2004 Naheed A, **Kalluri P**, Talukder KA, et al. Fluoroquinolone resistant *Shigella dysenteriae* Type 1 in northeastern Bangladesh. Lancet Infectious Diseases 2004; 4: 607-608.

2006 **Kalluri P**, Naheed A, Rahman S, et al. An evaluation of three rapid diagnostic test kits for cholera: does the skill level of the technician matter? Tropical Medicine & International Health 2006; 11: 49-55.

2006 **Ram PK**, Naheed A, Brooks WA et al. Risk factors for typhoid fever in a densely populated slum in Dhaka, Bangladesh. Epidemiology and Infection; epub Aug 8, 2006:1-8.

2007 Gupta S, Sheikh M, Islam M, Rahman K, Jahan N, Rahman M, Hoekstra R, Johnston R, **Ram P**, Luby S. Usefulness of the hydrogen sulfide test for assessment of water quality in Bangladesh. Journal of Applied Microbiology. 2007; epub ahead of print.

2008 **Ram PK**, Choi M, Blum LS, Wamae AW, Mintz ED, Bartlett AV. Declines in case management of diarrhoea among children < 5 years old. Bulletin of the World Health Organization. 2008; epub ahead of print

2008 **Ram PK**, Crump, JA, Gupta SK, Miller MA, Mintz ED. Analysis of Data Gaps Pertaining to *Shigella* Infections in Low- and Medium Human Development Index Countries, 1984-2005. Epidemiology and Infection. 2008; 136:577-603.

In Press Naheed A, **Ram PK**, Brooks WA, Mintz ED, Hossain A, Bird M, Luby SP, Breiman RF. Clinical Value of Tubex^TM^ and Typhidot® Rapid Diagnostic Tests for Typhoid Fever in an Urban Community Clinic in Bangladesh. Diagnostic Microbiology and Infectious Diseases.

**C. Research Support**

**ACTIVE**

Validation and improvement of measures of handwashing behavior 2/08 – 6/08

Water and Sanitation Program, The World Bank

Role: Principal Investigator

Objective: Validation and improvement measures of handwashing behavior and technical advice on measuring handwashing as part of monitoring and evaluation of four-country scale-up of handwashing promotion.

**Biography of the Investigators**

Give biographical data in the following table for key personnel including the Principal Investigator. Use a photocopy of this page for each investigator.

1 Name : Tasnim Azim

2 Present position : Scientist, Head HIV/AIDS Programme and

Virology Laboratory

3 Educational background : Ph.D., 1989, Immunology/Virology, University of

London, UK

(last degree and diploma & training relevant to the present research proposal)

4. List of ongoing research protocols

(Start and end dates; and percentage of time)

**As Principal Investigator**

| Protocol Number | Starting date | End date | Percentage of time |
| --- | --- | --- | --- |
| 2007-055 | 01-12-07 | 30-11-08 | 10% |
| Activity no. 00021 Bhutan Surveillance | 29/05/2006 | 28/11/2009 | 17% |
| Activity No. 00236 GFATM-IDU | 02/02/2008 | 31/05/2008 | 5% |
| Activity No. 00237-GFATM-Care & Support | 02/02/2008 | 30/04/2009 | 3% |
| Activity No. 00248, GFATM –OR Round 6 | 07/02/2008 | 30/04/2009 | 8.5% |
| Activity No. 00225 UNODC-ROSA H13 | 01-12-07 | 30-11-08 | 10% |

**As Co-Principal Investigator**

| Protocol Number | Starting date | End date | Percentage of time |
| --- | --- | --- | --- |
| Activity No.00231, PPTCT | 01/01/2008 | 31/12/2008 | 5% |
|  |  |  |  |

**As Co-Investigator**

| Protocol Number | Starting date | Ending date | Percentage of time |
| --- | --- | --- | --- |
| 2003-030 | 31/10/2003 | 29/09/2008 | 5% |
| 2007-002 | 01/01/2007 | 29/09/2008 | 5% |
| 2006-044 | 01-01-2007 | 31/12/08 | 15% |
| 2008-007 | 30/09/2007 | 30/03/2009 | 5% |

5. Publications

| Types of publications | Numbers |
| --- | --- |
| a) Original scientific papers in peer-review journals | 58 |
| b) Peer reviewed articles and book chapters | 2 |
| c) Papers in conference proceedings | >25 |
| c) Letters, editorials, annotations, and abstracts in peer-reviewed journals | 6 |
| Working papers |  |
| Monographs/reports | 8 |

6 Five recent publications including publications relevant to the present research protocol

Rahman M, Hassan ZM. Zafrul H, Saiada F, Banik A, Faruque ASG, Delbeck T, Matthijnssens J, van Ranst M, **Azim T**. Sequence analysis and evolution of group B rotaviruses. Virus Research 2007 125:219-225.

Brooks WA, Terebuch P, Bridges C, Klimov A, Goswami D, Sharmeen AT**, Azim T,** Erdman D, Hall H, Luby S, Breiman RF. **I**nfluenza A and B in children inurban slum, Bangladesh. Emerg Inf Dis 2007 (Oct).

Brooks WA, Erdman D, Terebuch P, Klimov A, Goswami D, Sharmeen AT**, Azim T,** Hall H, Luby S, Bridges C, Breiman RF. Human metapneumovirus infection among children, Bangladesh. Emerg Inf Dis 2007 13:1611-1613.

**Azim T,** Rahman M, Alam MS, Chowdhury IA, Khan R, Reza M, Rahman M, Chowdhury EI, Hanifuddin M, , Rahman ASMM., Bangladesh moves from being a low prevalence nation for HIV to one with a concentrated epidemic in injecting drug users. Int J STD AIDS 2008 (in press).

Sarker MS, Rahman M, Yirrell D, Khan R, Campbell E, Islam LN, **Azim T.** Molecular evidence for polyphyletic origin of human immunodeficiency virus type 1 subtype C in Bangladesh. Virus Research 2008 (in press).

**Biography of the Investigators**

Give biographical data in the following table for key personnel including the Principal Investigator. Use a photocopy of this page for each investigator.

**1 Name:** Eduardo Azziz-Baumgartner

**2 Present Position:** Scientist, Programme on Infectious Diseases and Vaccine Sciences, ICDDR,B

3 Educational background:

(last degree and diploma & training

relevant to the present research proposal)

**Bachelors in Science**, Molecular Biology (1993)

University of Michigan, Ann Arbor, Michigan

**Medical School,** (1997)

University of Alabama Medical School, Birmingham, Alabama

**Residency**, Family Medicine (2000)

University of Texas at San Antonio, San Antonio, Texas

**Fellowship in Minority Health Policy, Commonwealth** Fund (2003)

Harvard Medical School, Boston, Massachusetts

**Masters in Public Health**, Family and Community Medicine Health (2003)

Harvard School of Public, Boston, Massachusetts

**Epidemic Intelligence Service,** National Center for Environmental Health Division of Environmental Hazards and Health Effects, Health Studies Branch (2003-2005)

4.0 List of ongoing research protocols

(start and end dates; and percentage of time)

As Principal Investigator

| Protocol Number | Starting date | End date | Percentage of time |
| --- | --- | --- | --- |
|  |  |  |  |

As Co-Principal Investigator

| Protocol Number | Starting date | End date | Percentage of time |
| --- | --- | --- | --- |
|  |  |  |  |

As Co-Investigator

| Protocol Number | Starting date | End date | Percentage of time |
| --- | --- | --- | --- |
|  |  |  |  |

5 Publications

| Types of publications | Numbers |
| --- | --- |
| Original scientific papers in peer-review journals | 13 |
| Peer reviewed articles and book chapters |  |
| Papers in conference proceedings | 1 |
| Letters, editorials, annotations, and abstracts in peer-reviewed journals |  |
| Working papers | 0 |
| Monographs |  |

6 Five recent publications including publications relevant to the present research protocol

**Eduardo Azziz-Baumgartner**, **George Luber**, **Helen Schurz Rogers**, L. Backer, M. Belson, **Stephanie Kieszak**, K. Caldwell, B Lee, R Jones, R Todd, and **Carol Rubin**. Exposure assessment of a mercury spill in a Nevada school—2004Clinical Toxicology. 2007. Volume 45. 1–5**.**

**Heather Strosnider, Eduardo Azziz-Baumgartner, Marianne Banziger, Ramesh V. Bhat, Robert Breiman, Marie-Noel Brune, Kevin DeCock, Abby Dilley, John Groopman, Kerstin Hell, Sara H. Henry, Daniel Jeffers, Curtis Jolly, Pauline Jolly, Gilbert N. Kibata, Lauren Lewis, Xiumei Liu, George Luber, Leslie McCoy, Patience Mensah, Marina Miraglia, Ambrose Misore, Henry Njapau, Choon-Nam Ong, Mary T.K. Onsongo, Samuel W. Page, Douglas Park, Manish Patel, Timothy Phillips, Maya Pineiro, Jenny Pronczuk, Helen Schurz Rogers, Carol Rubin, Myrna Sabino, Arthur Schaafsma, Gordon Shephard, Joerg Stroka, Christopher Wild, Jonathan T. Williams, and David Wilson.** Workgroup Report: Public Health Strategies for Reducing Aflatoxin Exposure in Developing Countries. Environmental Health Perspectives. 2006. Volume 114. 1898–1903

Alberto B. Broce; Ludek Zurek; James A. Kalisch; Robert Brown; David L. Keith; David Gordon; Janis Goedeke; Cal Welbourn; John Moser; Ronald Ochoa; **Eduardo Azziz-Baumgartner**; Fuyuen Yip; Jacob Weber. *Pyemotes herfsi* (Acari: Pyemotidae), a Mite New to North America as the Cause of Bite Outbreaks. Journal of Medical Entomology. 2006. Volume 43. (3) 610 – 613.

**Eduardo Azziz-Baumgartner**; Wolkin, Amy; Sanchez, Carlos; Bayleyegn, Tesfaye; Young, Stacy; Kieszak, Stephanie; Oberst, Kathleen; Batts, Dahna; Thomas, Charles C.; Rubin, Carol. Impact of Hurricane Ivan on Pharmacies in Baldwin County, Alabama. [Journal of the American Pharmacists Association](http://www.ingentaconnect.com/content/apa/japha;jsessionid=34b8nm6n649vt.victoria). 2005. Volume 45. (6) 670-675.

**Eduardo Azziz-Baumgartner, Kimberly Lindblade, Karen Gieseker, Helen Schurz Rogers, Stephanie Kieszak, Henry Njapau, Rosemary Schleicher, Leslie F. McCoy, Ambrose Misore, Kevin DeCock, Carol Rubin, Lawrence Slutsker, and the Aflatoxin Investigative Group. Case-Control Study of an Acute Aflatoxicosis Outbreak - Kenya--2004 Environmental Health Perspectives 2005. Vol. 113 (12) 1779-1783.**

**Biography of the Investigators**

Give biographical data in the following table for key personnel including the Principal Investigator. Use a photocopy of this page for each investigator.

**1 Name: Joseph S. Bresee**

**2 Present Position:** Chief, Epidemiology and Prevention Branch, Influenza Division, Centers for Disease Control and Prevention, Atlanta, GA USA

**3 Educational background:**

1986-1990 M.D., Baylor College of Medicine, Houston, TX

1990-1993 **Internship/Residency in Pediatrics**, University of Washington School of Medicine/Children's Hospital and Medical Center, Seattle, WA

1993-1995 **Epidemic Intelligence Service** (EIS)

Influenza Branch

Division of Viral and Rickettsial Diseases

National Center for Infectious Diseases

Centers for Disease Control and Prevention

(last degree and diploma & training

relevant to the present research proposal)

**4.0** **List of ongoing research protocols**

(start and end dates; and percentage of time)

Dr. Bresee is Chief, Epidemiology and Prevention Branch, Influenza Division at the Centers for Disease Control and Prevention in Atlanta. The EPB is responsible for conducting influenza surveillance, working to understand influenza disease burden, helping to derive appropriate vaccine and antiviral use policies to prevent seasonal influenza, detecting and preventing avian influenza and pandemic influenza, and providing technical expertise to global public health partners. As such, Dr. Bresee is a collaborator on several influenza research studies, but only those related to Bangladesh are listed below.

As Principal Investigator

| Protocol Number | Starting date | End date | Percentage of time |
| --- | --- | --- | --- |
|  |  |  |  |
|  |  |  |  |
|  |  |  |  |
|  |  |  |  |

As Co-Principal Investigator

| Protocol Number | Starting date | End date | Percentage of time |
| --- | --- | --- | --- |
|  |  |  |  |
|  |  |  |  |
|  |  |  |  |
|  |  |  |  |

As Co-Investigator

| Protocol Number | Starting date | End date | Percentage of time |
| --- | --- | --- | --- |
| CDC IRB #4030 | 2003 | Sept 2008 | 5% |
| CDC IRB#4314 | 2003 | Dec 2007 | 2% |
|  |  |  |  |
|  |  |  |  |

Both of these protocols are ICDDR,B studies. CDC IRB#4030 is a surveillance study in Kamalpur, (PI Dr. Brooks). CDC IRB# 4314 is a study of effects of air pollution on respiratory disease (PI – Dr. Brooks)

**5 Publications**

| Types of publications | Numbers |
| --- | --- |
| Original scientific papers in peer-review journals | 85 |
| Peer reviewed articles and book chapters | 135 |
| Papers in conference proceedings | 5 |
| Letters, editorials, annotations, and abstracts in peer-reviewed journals | 3 |
| Working papers | 10 |
| Monographs | 0 |

**Biography of the Investigators**

Give biographical data in the following table for key personnel including the Principal Investigator. Use a photocopy of this page for each investigator.

NAME **W. Abdullah Brooks, MD, MPH**

POSITION TITLE

Head, Infectious Diseases Unit, Division of Health Systems and Vaccine Sciences, ICDDR,B – Centre for Health and Population Research

Asst. Scientist , Department of International Health, Johns Hopkins University School of Hygiene and Public Health

EDUCATION

| **Institution/Location** | **Degree** | **Year Conferred** | **Field of Study** |
| --- | --- | --- | --- |
| Stanford University | MD | 1991 | Medicine |
| The New York Hospital / Cornell Medical Center | Diploma | 1994 | Pediatrics |
| Johns Hopkins University | MPH | 1995 | International Health |
| Johns Hopkins University | Diploma | 1996 | Preventive Medicine |

EXPERIENCE AND APPOINTMENTS

| Year | Activity |
| --- | --- |
| 2007 – Present | Co-Investigator: National influenza hospital surveillance, Bangladesh |
| 2006 – 2007 | Co-PI: Efficacy Zinc in outpatient bronchiolitis in children < 2 y/o |
| 2006 – Present | Co-Inv: Role of prolactin in diagnosis of pneumonia in children |
| 2003 – Present | Principal Investigator: Efficacy Zinc in Outpatient Pneumonia in Children < 2 y/o |
| 2003 – Present | Principal Investigator: Pneumococcal disease burden urban study |
| 2003 – Present | Principal Investigator: Influenza, other respiratory virus burden of diesase |
| 2002 – 2004 | Typhoid burden of disease and risk factor study |
| 2002 – 2003 | Principal Investigator: Safety, Immunogenicity, Tolerability CAIV-T (Influenza) vaccine and MMR |
| 2002 – 2003 | Prevalence & risk factors for asthma among urban children, Dhaka |
| 2002 – Present | Unit Head, Infectious Diseases; Division of Health Systems & Infectious Disease |
| 2001 – 2002 | Co-PI: Safety, Immunogenicity, Tolerability CAIV-T (Influenza) vaccine and OPV |
| 2001 – Present | Co-PI: Shigella burden of disease study |
| 2001 – 2002 | Principal Investigator: Typhoid burden of disease in urban Dhaka |
| 2000 – Present | Principal Investigator: Community-based Emergency Epidemiological Study Dengue |
| 1999 – 2001 | Principal Investigator: Hospital-based study of efficacy zinc as adjuvant therapy in management severe pneumonia, hospitalised children < 2 y/o |
| 1998 – Present | Principal Investigator: Demographic Surveillance System, Kamalapur, Dhaka |
| 1998 – 2001 | Principal Investigator: Community-based study to prevent pneumonia/diarrhoea with zinc in children less than 2 years old |
| 1997 – Present | Regional Medical Consultant: International SOS, Singapore |
| 1997 – 2001 | Principal Investigator: Hospital-based study to test efficacy zinc in acute watery diarrhoea in children less than 6 months old |
| 1997 – Present | Paediatric Consultant, UNOCAL |
| 1997 – Present | Regional Medical Consultant: International SOS, Singapore |
| 1997 – 2001 | Paediatric Consultant, US Embassy, Dhaka |
| 1996 – 1997 | Chief Resident Preventive Medicine Program, Johns Hopkins University |
| 1996 | EPI Technical Advisor: National measles vaccination campaign children 12 - 59 months, PAHO, EPI/SVI, Georgetown, Guyana |
| 1996 | Epidemiologist: Consultants in Epidemiology and Occupational Health, Washington, DC |
| 1995 – 1997 | Paediatric Emergency Room Attending, St. Joseph’s Hospital, Baltimore |
| 1995 – 1997 | Paediatric consultant: Urban school-based clinics for Baltimore County |
| 1994 – 1997 | Paediatric On-Call Physician, Kennedy-Krieger Institute, Johns Hopkins |
| 1995 | Multicentre Study on Lower Osmolar ORS, International Centre for Diarrhoeal Disease Research, Bangladesh (ICDDR,B) |
| 1992 | Investigator: Community-based dysentery intervention urban slum children, Salvador, Bahia, Brazil |
| 1990 | Epidemiology Intelligence Service Medical Student Clerkship, Enteric Branch, Centers for Disease Control and Prevention |
| 1986 | Investigator: Isolation of *L. major* attachment proteins, National Institutes of Health |
| 1985-1986 | Principal Investigator: Identification of heat-shock proteins in *F.hepatica*, Stanford University |

Honours and Awards (Selected)

Time Magazine 2005, ‘The year in medicine’ recognition of Brooks et al. Lancet, 2005. 366(9490): p. 999-1004 contribution to child mortality reduction

Awardee Office of Dietary Supplements, National Institutes of Health, 100 most significant advances in Annual Bibilography of Significant Advances in Dietary Supplement Research 2004 for Brooks et al. Lancet, 2004. 363(9422): p. 1683-8.

Chief Residency, Preventive Medicine, Johns Hopkins University School of Public Health 1996 – 7

Awardee Health & Child Survival Scholarship to Johns Hopkins University 1994 – 5

Selectee Ciba-Geigy Medical Student (competitive) Scholarship for internship NIH 1985 while at Stanford Medical School

PUBLICATIONS (Selected)

**Brooks, W.A**., Breiman, R. F., Goswami, D., Hossain, A., Alam, K., Saha, S. K., Nahar, K., Nasrin, D., Ahmed, M. D., Arifeen, S. E., Naheed, A., Sack, D. A., Luby, S. , Invasive pneumococcal disease burden, seasonality, and antimicrobial resistance patterns, and implications for vaccine policy in urban Bangladesh. *In Press, 2007*.

Ram, P.K., Naheed, A., **Brooks, W.A.**, Hossain, M.A., Mintz, E.D., Breiman, R.F., and Luby, S.P., Risk factors for typhoid fever in a slum in Dhaka, Bangladesh. Epidemiol Infect, 2007. 135(3): p. 458-65.

Islam, M.S., **Brooks, A**., Kabir, M. S., Jahid, I. K., Islam, M. S., Goswami, D., Nair, G. B., Larson, C., Yukiko, W., Luby, S., Faecal contamination of drinking water sources of Dhaka during the 2004 flood in Bangladesh and use of disinfectants for water treatment. J Appl Microbio, 2006.

**Brooks, W.A.**, Santosham, M., and Black, R.E., Zinc, infectious diseases, and low birth weight. Am J Clin Nutr, 2006. 84(3): p. 667.

Harris, J.B., Baresch-Bernal, A., Rollins, S.M., Alam, A., LaRocque, R.C., Bikowski, M., Peppercorn, A.F., Handfield, M., Hillman, J.D., Qadri, F., Calderwood, S.B., Hohmann, E., Breiman, R.F., **Brooks, W.A.,** and Ryan, E.T., Identification of in vivo-induced bacterial protein antigens during human infection with Salmonella enterica serovar Typhi. Infect Immun, 2006. 74(9): p. 5161-8.

**Brooks, W.A.,** Santosham, M., Naheed, A., Goswami, D., Wahed, M.A., Diener-West, M., Faruque, A.S., and Black, R.E., Effect of weekly zinc supplements on incidence of pneumonia and diarrhoea in children younger than 2 years in an urban, low-income population in Bangladesh: randomised controlled trial. Lancet, 2005. 366(9490): p. 999-1004.

**Brooks, W.A**., Hossain, A., Goswami, D., Nahar, K., Alam, K., Ahmed, N., Naheed, A., Nair, G.B., Luby, S., and Breiman, R.F., Bacteremic typhoid fever in children in an urban slum, Bangladesh. Emerg Infect Dis, 2005. 11(2): p. 326-9.

**Brooks, W.A**., Santosham, M., Roy, S.K., Faruque, A.S., Wahed, M.A., Nahar, K., Khan, A.I., Khan, A.F., Fuchs, G.J., and Black, R.E., Efficacy of zinc in young infants with acute watery diarrhea. Am J Clin Nutr, 2005. 82(3): p. 605-10.

LaRocque, R.C., Breiman, R.F., Ari, M.D., Morey, R.E., Janan, F.A., Hayes, J.M., Hossain, M.A., **Brooks, W.A**., and Levett, P.N., Leptospirosis during dengue outbreak, Bangladesh. Emerg Infect Dis, 2005. 11(5): p. 766-9.

**Brooks, W.A**., Yunus, M., Santosham, M., Wahed, M.A., Nahar, K., Yeasmin, S., and Black, R.E., Zinc for severe pneumonia in very young children: double-blind placebo-controlled trial. Lancet, 2004. 363(9422): p. 1683-8.

RELEVANT MANUSCRIPTS UNDER REVIEW

**Brooks, W.A.,** Terebuh, P., Bridges, C., Klimov, A., Goswami, D., Sharmeen., Azim, T., Erdman, D., Hall, H., Luby, S., Breiman, R.F., Influenza A and B infection among children in an urban slum of Dhaka, Bangladesh: A pilot study. *Manuscript submitted and under review, 2007*.

**Brooks, W.A.,** Erdman, D., Terebuh, P., Klimov, A., Goswami, D., Sharmeen., Azim, T., Luby, S., Bridges, C., Breiman, R.F., Human metapneumovirus infection among children in an urban slum of Dhaka, Bangladesh: A pilot study. *Manuscript submitted and under review, 2007*.

BOOKS/GUIDELINES

Guidelines for the control of shigellosis, including Shigella dysenteriae type 1. WHO 2005

GRANTS

**Health and Human Services:** Population-based influenza surveillance among children < 5 years old in Kamalapur in Dhaka, Bangladesh. **US $250,000** 2006 - 2007

**Thrasher Research Foundation:** Efficacy of Zinc in the Treatment of Outpatient Pneumonia Among Urban Slum Children Less than Two Years Old. **US $250,000**; 2004 – 2007

**Centers for Disease Control and Prevention:** Surveillance for influenza and the viral aetiologies of influenza-like febrile illnesses in an urban slum in Dhaka, Bangladesh **US $392,133**; 2004-2008

**Accelerated Development and Introduction Plan (ADIP):** Burden of Pneumococcal Disease in children in Bangladesh: A Project to Enhance Laboratory Capacity and Create Awareness, and to Prepare for Introduction of a Pneumococcal Vaccine. **US $599,816**; 2004 – 2008

**Bill & Melinda Gates Foundation:** Efficacy of Zinc in the Treatment of Outpatient Pneumonia Among Urban Slum Children Less than Two Years Old. **US $250,591**; 2004 – 2007

**Wyeth:** A Prospective, Randomized, Double Blind, Placebo-Controlled, Trial to Assess Safety, Efficacy, Tolerability and Immunogenicity of Influenza Virus Vaccine, Trivalent, Types A & B, Live Cold-Adapted, Liquid Formulation (CAIV-T), Administered Concomitantly with a Combination Live, Attenuated, Mumps, Measles, and Rubella Vaccine in Healthy Children Aged 11 – 24 Months Protocol No. D153 P522. **US $116,419**; 2002 – 2003

**National Institutes of Health (ICIDR):** Emergency epidemiological study of dengue and dengue haemorrhagic fever in Dhaka, Bangladesh. **US $100,097**; 2002 – 2003

**Wyeth:** A prospective, randomised, partially-blinded, placebo-controlled, Phase III, multicentre trial to assess safety, tolerability and imunogenicity of liquid influenza virus vaccine, trivalent, types A & B, live cold-adapted (liquid CAIV-T) administered concomitantly with live, attenuated, poliovirus vaccine in healthy children. US **$185,845**; 2001 – 2002

**International Vaccine Institute:** Population based evaluation of *Shigella* infections in an urban area of Dhaka, Bangladesh **US $459,672**; 2001 – 2004

**United States Agency for International Development/Johns Hopkins University**: Efficacy of Zinc in the Prevention of Pneumonia in Urban Slum Children in Dhaka, Bangladesh. **US $115,102**; 1998 – 2001

**Swiss Development Corporation:** Efficacy of Zinc in the Prevention of Pneumonia in Urban Slum Children in Dhaka, Bangladesh. **US $50,000**; 1998 - 2001

**United States Agency for International Development (USAID):** Efficacy of Zinc in the Prevention of Pneumonia in Urban Slum Children in Dhaka, Bangladesh. **US $65,000**; 1998 – 2001

**United States Agency for International Development (USAID):** Efficacy of Zinc in the Treatment of Severe Pneumonia Among Hospitalised Children less than Two Years Old. **US $62,365**; 1999 – 2001

**United States Agency for International Development (USAID)/Johns Hopkins University:** Efficacy of zinc in treatment of acute watery diarrhoea in infants less than six months old. **US $13,355;** 1998 – 2000

**Biography of the Investigators**

Give biographical data in the following table for key personnel including the Principal Investigator. Use a photocopy of this page for each investigator.

| NAME  DiVita, Margaret Anne | | POSITION TITLE  PhD Candidate | | |
| --- | --- | --- | --- | --- |
| eRA COMMONS USER NAME  n/a | |
| EDUCATION/TRAINING *(Begin with baccalaureate or other initial professional education, such as nursing, and include postdoctoral training.)* | | | | |
| INSTITUTION AND LOCATION | DEGREE  *(if applicable)* | | YEAR(s) | FIELD OF STUDY |
| SUNY Geneseo | BA | | 1999-2002 | Anthropology and Spanish |
| SUNY Binghamton | MS | | 2002-2004 | BioMedical Anthropology |
| SUNY Buffalo | -- | | 2004- current | Epidemiology and Community Health |

**A. Honors**

**Honors and Awards**

5/2002 Magna Cum Laude, SUNY Geneseo

**Biography of the Investigators**

Give biographical data in the following table for key personnel including the Principal Investigator. Use a photocopy of this page for each investigator.

**Alicia M. Fry**

**Centers for Disease Control and Prevention**

**1600 Clifton Road, Mailstop A-34**

**Atlanta, GA 30333**

**Phone: (404) 639-2680**

**Email:** [**afry@cdc.gov**](mailto:afry@cdc.gov)

**December 2006**

**EDUCATION AND TRAINING** (Relevant to proposal)

Postgraduate

7/93- 6/95 Infectious Diseases Fellowship (overlapping with Molecular Medicine Fellowship)

University of California, San Francisco

7/90 – 6/93 Internal Medicine Internship and Residency

The Johns Hopkins Hospital, Baltimore, Maryland

**Graduate/Undergraduate**

6/98-5/99 Masters of Public Health (Epidemiology)

University of California, Berkeley, School of Public Health

9/84-5/90 Doctorate of Medicine

University of Cincinnati College of Medicine, Cincinnati, Ohio

**CURRENT POSITION**

**Centers for Disease Control and Prevention**

Medical Epidemiologist

Division of Viral Diseases,

Epidemiology Branch,

**National Center for Immunization and Respiratory Diseases**

**5 PEER-REVIEWED PUBLICATIONS (Relevant to proposal)**

Fry AM, Lu X, Peret TDT, Chittaganpitch M, Fischer J, Erdman DD, Olsen SJ. Human bocavirus: a novel parvovirus epidemiologically associated with hospitalized pneumonia in Thailand. *J Infect Dis* (in press)

Fry AM, Curns A, Harbour K, Anderson LJ. Seasonal Trends of Human Parainfluenza Viruses in the United States; National Respiratory Virus Surveillance Data, 1990-2002, *Clin Inf Dis* 2006 43(8):1016-22.

Fry, AM, Shay DK, Holman R, Curns A, Anderson LJ. Trends in hospitalizations for pneumonia among persons 65 years of age and older in the United States, 1988–2002. *JAMA* 2005; 294(21):2712-2719.

Fry AM, Udeagu CC, Soriano-Gabarro M, Fridkin S, Musinski D, LaClaire L, Elliott J, Cook DJ, Kornblum J, Layton M, Whitney CG.. Persistence of a fluoroquinolone-resistant multidrug-resistant *Streptococcus pneumoniae* in a long-term care facility: Efforts to decrease transmission, *Infection Control and Hospital Epidemiology* 2005;23: 239-47.

Fry AM, Facklam R, Whitney CG, Plikaytis BD, and Schuchat A. Multi-state evaluation of invasive pneumococcal diseases in adults with HIV infection: serotype and antimicrobial resistance patterns in the United States, *J Infect Dis* 2003: 181; 643.

**Biography of the Investigators**

Give biographical data in the following table for key personnel including the Principal Investigator. Use a photocopy of this page for each investigator.

**1 Name: Stephen P Luby**

**2 Present Position:** Head, Programme on Infectious Diseases and Vaccine Sciences.

University of Texas Southwestern Medical School at Dallas

MD, 1986

University of Rochester Strong Memorial Hospital

Internship and residency in Internal Medicine.

Centers for Disease Control -- Epidemic Intelligence Service 1990

Completed Preventive Medicine Residency 1993.

**3 Educational background:**

(last degree and diploma & training

relevant to the present research proposal)

**4.0** **List of ongoing research protocols**

(start and end dates; and percentage of time)

As Principal Investigator

| Protocol Number | Starting date | End date | Percentage of time |
| --- | --- | --- | --- |
| 2003-024 | 1 Sep 2003 | 31 Dec 2006 | 5 |
| 2005-026 | 1 Oct 2005 | 31 Dec 2006 | 5 |
| 2005-023 | 1 Feb 2006 | 31 Dec 2007 | 5 |
| 2006-043 | 1 Nov 2006 | 31 July 2007 | 5 |
| 2007-003 | 1 May 2007 | 31 Apr 2008 | 3 |
| 2007-002 | 1 May 2007 | 30 Sep 2008 | 5 |
| 2007-010 | 1 July 2007 | 30 Sep 2008 | 5 |
| 2007-004 | 1 May 2007 | 30 Sep 2008 | 1 |
| 2007-030 | 1 Sep 2007 | 31 Dec 2008 | 5 |

As Co-Principal Investigator

| Protocol Number | Starting date | End date | Percentage of time |
| --- | --- | --- | --- |
| 2003-024 | Jun 2004 | June 2006 | 5 |
| 2003-002 | June2003 | Dec 2006 | 5 |

4.3 As Co-Investigator

| Protocol Number | Starting date | End date | Percentage of time |
| --- | --- | --- | --- |
|  |  |  |  |
|  |  |  |  |
|  |  |  |  |
|  |  |  |  |

**5 Publications**

| Types of publications | Numbers |
| --- | --- |
| Original scientific papers in peer-review journals | 118 |
| Peer reviewed articles and book chapters | 9 |
| Papers in conference proceedings | 1 |
| Letters, editorials, annotations, and abstracts in peer-reviewed journals | 4 |
| Working papers | 0 |
| Monographs | 0 |

**Five recent publications including publications relevant to the present research protocol**

Luby SP, Agboatwalla M, Feikin DR, Painter J, Billhimer W, Altaf A, Hoekstra RM. Effect of handwashing on child health: a randomised controlled trial. Lancet. July 15, 2005; 366:225-33.

Ram PK, Naheed A, Brooks WA, Hossain MA, Mintz ED, Breiman RF, Luby SP. Risk factors for typhoid fever in a densely populated slum in Dhaka, Bangladesh. *Epidemiology and Infection*. 2007 135:458-65.

Bowen A, Huilai M, Ou J, Billhimer W, Long T, Mintz E, Hoekstra RM, Luby S. A Cluster-Randomized Controlled Trial Evaluating the Effect of a Handwashing Promotion Program in Chinese Primary Schools, *American Journal of Tropical Medicine and Hygiene*. June 2007 76(6):1166-1173.

Brooks WA, Breiman RF, Goswami D, Hossain A, Alam K, Saha S, Narar K, Nasrin D, Ahmed N, El Arifeen S, Naheed A, Sack D, Luby S. Invasive pneumococcal disease burden and implications for vaccine policy in urban Bangladesh. *American Journal of Tropical Medicine and Hygiene*. 2007 Nov 77(5): 795–801.

Luby SP, Halder AK. Associations among handwashing indicators, wealth, and symptoms of childhood respiratory illness in urban Bangladesh. Tropical Medicine and International Health. 2008 Jun 13(6):835-844.

**Biography of the Investigators**

Give biographical data in the following table for key personnel including the Principal Investigator. Use a photocopy of this page for each investigator.

**1 Name: Mustafizur Rahman**

**2 Present Position:** Senior Research Officer, Virology Laboratory, LSD, ICDDR,B.

1995 M.Sc. in Microbiology, University of Dhaka. Bangladesh.

2001-2002 Training in Virology, University of Leuven, Belgium.

2002 Training in Bio-informatics in Charles University, Prague, Czech Republic.

2007 IEIP/GDD Laboratory Workshop for Respiratory Disease, Centers for Disease Control, Atlanta, USA.

**3 Educational background:**

(last degree and diploma & training

relevant to the present research proposal)

**4.0** **List of ongoing research protocols**

(start and end dates; and percentage of time)

As Principal Investigator

| Protocol Number | Starting date | End date | Percentage of time |
| --- | --- | --- | --- |
|  |  |  |  |
|  |  |  |  |
|  |  |  |  |
|  |  |  |  |

As Co-Principal Investigator

| Protocol Number | Starting date | End date | Percentage of time |
| --- | --- | --- | --- |
|  |  |  |  |
|  |  |  |  |
|  |  |  |  |
|  |  |  |  |

As Co-Investigator

| Protocol Number | Starting date | End date | Percentage of time |
| --- | --- | --- | --- |
|  |  |  |  |
|  |  |  |  |
|  |  |  |  |
|  |  |  |  |

**5 Publications**

| Types of publications | Numbers |
| --- | --- |
| Original scientific papers in peer-review journals | 25 |
| Peer reviewed articles and book chapters | 1 |
| Papers in conference proceedings | 0 |
| Letters, editorials, annotations, and abstracts in peer-reviewed journals | 0 |
| Working papers | 5 |
| Monographs | 0 |

**6 Five recent publications including publications relevant to the present research protocol**

**Rahman, M**., J. Matthijnssens, X. Yang, T. Delbeke, I. Arijs, K. Taniguchi, M. Iturriza-Gomara, N. Iftekharuddin, T. Azim, and M. Van Ranst. 2007. Evolutionary history and global spread of the emerging G12 human rotaviruses. J Virol 81:2382-90.

**Rahman, M.**, R. Sultana, G. Ahmed, S. Nahar, Z. M. Hassan, F. Saiada, G. Podder, A. S. G. Faruque, A. K. Siddique, D. A. Sack, J. Matthijnssens, M. Van Ranst, and T. Azim. 2007. Prevalence of G2P[4] and G12P[6] rotavirus, Bangladesh. Emerg Infect Dis 13:18-24.

**Rahman, M**., Z. M. Hassan, H. Zafrul, F. Saiada, S. Banik, A. S. G. Faruque, T. Delbeke, J. Matthijnssens, M. Van Ranst, and T. Azim. 2007. Sequence analysis and evolution of group B rotaviruses. Virus Res 125:219-25.

**Rahman, M**., J. Matthijnssens, T. Goegebuer, K. De Leener, L. Vanderwegen, I. Van der Donck, L. Van Hoovels, S. De Vos, T. Azim, and M. Van Ranst. 2005. Predominance of rotavirus G9 genotype in children hospitalised for rotavirus gastroenteritis. J Clin Virology 33:1-6.

**Rahman, M.**, J. Matthijnssens, S. Nahar, G. Podder, D. A. Sack, T. Azim, and M. Van Ranst. 2005. Characterization of a novel P[24],G11 human group A rotavirus. J Clin Microbiol 43:3208-12.

# Biography of the Investigators

Give biographical data in the following table for key personnel including the Principal Investigator. Use a photocopy of this page for each investigator.

(Note: Biography of the external Investigators may, however, be submitted in the format as convenient to them)

**1 Name:** Rashid Uz Zaman

**2 Present Position:** Research Investigator, PIDVS, ICDDR,B

**3 Educational background:**

Post Graduate Diploma in Health Economics (PGDHE), University of Dhaka

Bachelor of Medicine and Surgery (MBBS), University of Dhaka

(last degree and diploma & training

relevant to the present research proposal)

**4.0** **List of ongoing research protocols**

(start and end dates; and percentage of time)

As Principal Investigator

| Protocol Number | Starting date | End date | Percentage of time |
| --- | --- | --- | --- |
|  |  |  |  |
|  |  |  |  |
|  |  |  |  |
|  |  |  |  |

As Co-Principal Investigator

| Protocol Number | Starting date | End date | Percentage of time |
| --- | --- | --- | --- |
| 2007-002 | Apr 2007 | Apr 2009 | 70% |
|  |  |  |  |
|  |  |  |  |
|  |  |  |  |

As Co-Investigator

| Protocol Number | Starting date | End date | Percentage of time |
| --- | --- | --- | --- |
| 2006-054 | Mar 2007 | Jan 2009 | 10% |
| 2007-010 | Jul 2007 | Jul 2008 | 5% |
| 2007-031 | Oct 2007 | Oct 2008 | 5% |
| 2008-001 | May 2008 | Apr 2008 | 10% |

**5 Publications**

| Types of publications | Numbers |
| --- | --- |
| Original scientific papers in peer-review journals | 2 |
| Peer reviewed articles and book chapters |  |
| Papers in conference proceedings | 2 |
| Letters, editorials, annotations, and abstracts in peer-reviewed journals |  |
| Working papers | 1 |
| Monographs |  |

**6 Five recent publications including publications relevant to the present research protocol**

1) ICDDR,B, 2008, Hospital based surveillance revealed high prevalence of influenza in Bangladesh. Health Sci Bull 2008; 6(1):1-5

2) Begum B, Zaman R, Ahmed SMU, Ali S. Burst abdomen – a preventable morbidity. Mymensingh Med J. 2008 Jan; 17(1): 63-6

3) Hossain N, Zaman RU, Banks N, Gierbo HC. The incentives and constrains of Government Doctors in Primary Healthcare Facilities in Bangladesh. BRAC/RED Research Report: November 2007

4) Ahmed SMU, Kakehi Y, Zaman RU, Hasan AU. Role of Brachytherapy in curbing prostatic cancer. Bang Med J (Khulna) 2007; 40(1 & 2); 16-19

5) Begum B, Uddin KU, Rahman MA, Habib A, Yeasmin S, Zaman RU. Better management expectation for Pelvic Inflammatory Disease. Bangladesh Medical Journal 2004; 33(4): 132-135

# Detailed Budget


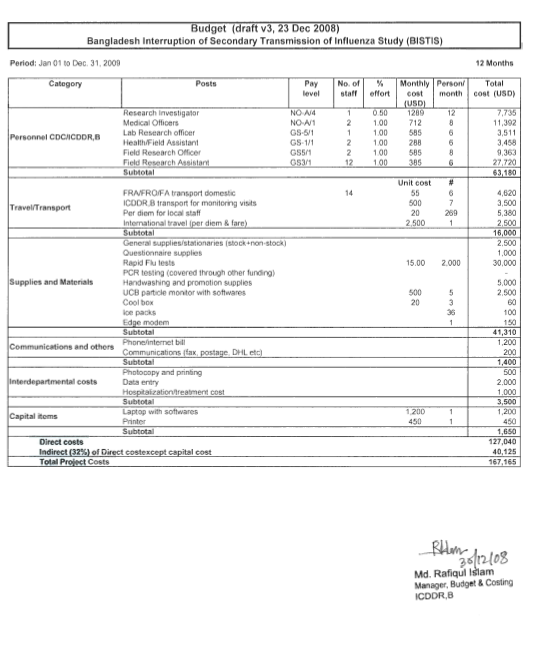


# Budget Justifications

­­

Please provide one page statement justifying the budgeted amount for each major item. Justify use of human resources, major equipment, and laboratory services.

# Personnel

PD/PI: A Research Investigator from ICDDR,B will allot 50% of his/her time to oversee all the activities of the study.

Medical Officer: Two full time (100%) Medical Officers will be hired for 8 months on CSA basis who will be stationed in the field site. They will be responsible for identifying index case patients from the selected hospital and collect their samples from the departments of medicine and pediatrics. They will also provide other technical supports to the study including field management.

Lab Research Officer: A full time Research Officer will be hired to support the lab activities during the overall project time period.

Health assistant: Two full time health assistants will be employed to assist in daily logistical and sample collection activities including transport of specimens.

Field research officer: Two FROs are budgeted 100% time to ensure implementation and coordinate the research activities at the community level and also support and oversee the overall activities of the FRAs.

Field research assistants: 12 full time FRAs have been budgeted who will be responsible for field implementation of the planned activities and to collect data from the community. They will work by dividing into two groups (FIS and FRA).

**Travel**

Local travel: We have budgeted $4,620 to cover the local travel costs associated with the movement by the FROs and FRAs across villages in 3-4 upazillas. This cost will also cover the cost of specimen transport by the FAs from field to ICDDR,B lab.

Per diem for staff: The MOs, FROs, FRAs and the FAs will be stationed in the field site. However their trainings and meetings will be conducted in Dhaka. Moreover, the PI and the other investigators will often pay monitoring visits to the field. For this we have allocated $5,380 to cover all their per diem during the life of the project. The average per diem for all level staff was considered about $20 per night.

**Supplies and lab supplies**

We have allocated a total of $41,310 for general stock and non-stock items, rapid tests, for handwashing and promotion supplies and for UCB particle monitors and cool boxes. We have also allocated money to buy an EDGE modem for the field staff for communication via internet.

**Communications and others**

We have budgeted $1200 for internet and mobile phone bill for our field staff and $200 for fax, postage, DHL etc.

**Equipment**

We will provide a laptop computer with software to the field staff for internet communication and for tracking the field activities. We have kept $1200 for this purpose.

**Interdepartmental cost**

A total amount of $3,500 is budgeted to cover some of interdepartmental cost like data entry, photocopy and hospitalization/treatment cost of the patients.

# Other Support

Describe sources, amount, duration, and grant number of all other research funding currently granted to PI or under consideration.

# Appendix 1: Figure and Details about Bari

| 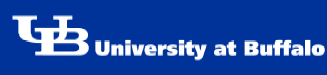 | The Bangladesh Interruption of Secondary Transmission of Influenza Study (BISTIS)Jahurul Islam Medical College Hospital, Kishorgonj | 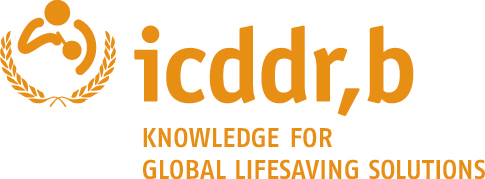 |
| --- | --- | --- |

Instructions: Draw the overall layout of the bari, including the location of the following:

Entry / entries to bari

Each household within bari

Latrine(s)

Cooking space(s)

**Below is an example of a drawing of two baris. You should label your drawing, as shown in the bari on the bottom left.**

#
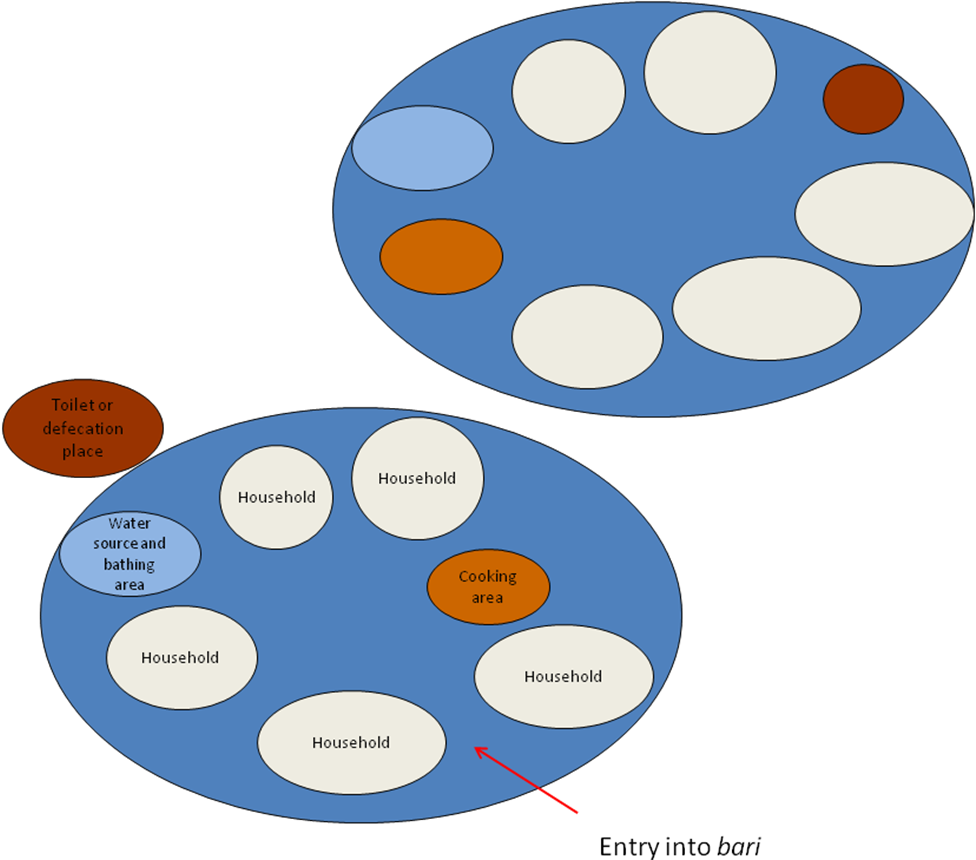


#

# Appendix 2: Adult Consent Form: Specimen Collection

**Protocol Number: 2009-004**

**Protocol Title:** Prevention of secondary transmission of human influenza by promoting handwashing with soap: The Bangladesh Interruption of Secondary Transmission of Influenza Study (BISTIS)

**Investigator’s name**: Dr. Stephen P. Luby

**Organization:** ICDDR,B

Name of Index Case Patient: _____________________________________

Unique ID: **___ ___ ___ ___ ___ ___ ___ ___ ___ ___ ___**

**Introduction**

You are invited to take part in a research trial. Scientists from the International Centre for Diarrhoeal Diseases Research, Bangladesh (ICDDR,B), Centers for Disease Control and Prevention (CDC), and University at Buffalo, a university in the USA, are doing a research study to understand factors associated with the spread of influenza virus in a rural population.

**Purpose of the research**

We are trying to understand factors that are connected to the spread of influenza virus within a bari. We would also like to understand whether washing hands with soap prevents the spread of this illness. Around 400 baris will be part of the study. We will aim to find out whether the spread of influenza is linked to specific behaviors.

**Why selected**

We are asking you to participate in this study because, in the past 7 days, you have had symptoms of cough and/or sore throat and a fever. These symptoms may be related to an illness called influenza. We are interested in studying this illness.

**What is expected from the patient/respondent?**

For probable index case-patients ONLY

We ask you to allow one of our trained research personnel to take a swab from your nose. This swab will then be tested for influenza.

We will then take a second swab from your nose and also a swab from your throat. We will use these swabs to test for the type of influenza you may have. If you agree, we will store the material from the swabs for future testing for twenty years, after which the samples will be destroyed. Such testing may include tests for respiratory illnesses other than influenza. The results of the future testing will not affect medical care, and, thus, these results will not be reported. You can ask to have the material from your swabs removed from storage at any time by calling Dr. Stephen P. Luby at 8860523-32 # 2502.

For probable secondary/follow-up case-patients ONLY

We ask you to allow one of our trained research personnel to take a swab from your nose and throat. These swabs will then be tested for influenza at a later time. If you agree, we will store the material from the swabs for future testing for twenty years, after which the samples will be destroyed. Such testing may include tests for respiratory illnesses other than influenza. The results of the future testing will not affect medical care, and, thus, these results will not be reported. You can ask to have the material from your swabs removed from storage at any time by calling Dr. Stephen P. Luby at 8860523-32 # 2502.

**Risk and benefits**

One of our trained research personnel will have to place a swab into your nose and a different swab into your throat. This may be uncomfortable. There are no other known risks for this procedure. There is no specific benefit to you having this test done. Because the results of the swab will not affect your medical care in any way, we will not be providing you with the results of your swab. But, information from the swab that we take from you will help us to understand how the influenza virus is spread within a bari.

**Privacy, anonymity and confidentiality**

All of the information we collect about you will be kept private and confidential. We will keep all paper documents in a locked cabinet. We will not give any information about you to anyone not involved in the study. Your name will never be used in reports of this study.

**Future use of information**

If the information we collect needs to be used for future use by other researchers, we will not supply any personal information and will maintain strict privacy.

**Right not to participate**

You may choose to allow us to take swabs from your nose and throat or not to allow us to take these swabs. You may choose to ask us to stop taking the specimen or to discard the specimen before testing it. Refusal to participate will involve no penalty or loss of benefits at the hospital. Even if you do not allow us to take this specimen, you will still receive the usual care at the hospital. You will not be denied any treatments or benefits for which you would otherwise be eligible. If you choose to allow us to take these swabs, you may choose to allow us to store the specimens or not allow us to store the specimens. You may still participate in the study even if you refuse to allow us to store your specimens. Refusal to allow us to store your specimens will involve no penalty or loss of benefits at the hospital. Even if you do not allow us to store your specimens, you will still receive the usual care at the hospital. You will not be denied any treatments or benefits for which you would otherwise be eligible.

**Principle of compensation**

There is no cost to you for allowing us to take a nose and/or throat specimen. There will also be no compensation to you.

**Persons to Contact:**

If you have questions during the procedure, ask at any time. If you have any additional questions about the surveillance you may contact:

Dr. Stephen P. Luby, Programme on Infectious Diseases and Vaccine Sciences (PIDVS), ICDDR,B, Mohakhali, Dhaka 1212. Phone: 8860523-32 # 2502,

If you have questions about your rights in regards to being part of this research surveillance or if you think some harm has been done to you because of the surveillance you may contact:

Mr. M. A. Salam, Research and Project Support Department (RPSD), ICDDR,B, Mohakhali, Dhaka 1212. Phone: 9886489, 01711428989

If you agree to our proposal of obtaining a nose and/or throat specimen, please indicate that by putting your signature or your left thumb impression at the specified space below.

Thank you for your cooperation

_______________________________________ ____________________

Signature or left thumb impression of subject Date

_______________________________________ ____________________

Signature or left thumb impression of the witness Date

_______________________________________ ___________________

Signature of the PI or his/her representative Date

### Appendix 3: Parent or Guardian Consent Form: Specimen Collection

**Protocol Number: 2009-004**

**Protocol Title:** Prevention of secondary transmission of human influenza by promoting handwashing with soap: The Bangladesh Interruption of Secondary Transmission of Influenza Study (BISTIS)

**Investigator’s name**: Dr. Stephen P. Luby

**Organization:** ICDDR,B

Name of Index Case Patient: _____________________________________

Unique ID: **___ ___ ___ ___ ___ ___ ___ ___ ___ ___ ___**

**Introduction**

Your child is invited to take part in a research trial. Scientists from the International Centre for Diarrhoeal Diseases Research, Bangladesh (ICDDR,B), Centers for Disease Control and Prevention (CDC), and University of Buffalo, a university in the USA, are doing a research study understand factors associated to the spread of influenza virus in a rural population.

**Purpose of the research**

We are trying to understand factors that are connected to the spread of influenza virus within a bari. We would also like to understand whether washing hands with soap prevents the spread of this illness. Around 400 baris will be part of the study. We will aim to find out whether the spread of influenza is linked to specific behaviors.

**Why selected**

We are asking your child to participate in this study because he/she has had in the past 7 days a cough and/or sore throat and a fever. These symptoms are related to an illness called influenza. We are interested in studying this illness.

**What is expected from the patient/respondent?**

For probable index case-patients ONLY

We ask you to allow one of our trained research personnel to take a swab from your child’s nose. This swab will then be tested for influenza.

We will then take a second swab from your child’s nose and also a swab from your child’s throat. We will use these swabs to test for the type of influenza your child may have. If you agree, we will store the material from the swabs for future testing for twenty years, after which the samples will be destroyed. Such testing may include tests for respiratory illnesses other than influenza. The results of the future testing will not affect medical care, and, thus, these results will not be reported. You can ask to have the material from your child’s swabs removed from storage at any time by calling Dr. Stephen P. Luby

at 8860523-32 # 2502.

For probable secondary/follow-up case-patients ONLY

We ask you to allow one of our trained research personnel to take a swab from your child’s nose and throat. These swabs will then be tested for influenza at a later time. If you agree, we will store the material from the swabs for future testing for twenty years, after which the samples will be destroyed. Such testing may include tests for respiratory illnesses other than influenza. The results of the future testing will not affect medical care, and, thus, these results will not be reported. You can ask to have the material from your child’s swabs removed from storage at any time by calling Dr. Stephen P. Luby

at 8860523-32 # 2502.

**Risk and benefits**

One of our trained research personnel will have to place a swab into your child’s nose and a different swab into your child’s throat. This may be uncomfortable. There are no other know risks for this procedure. There is no specific benefit to you having this test done. Because the results of the swab will not affect your child’s medical care in any way, we will not be providing you with the results of your child’s swab. But, information from the swab that we take from your child will help us to understand how the influenza virus is spread within a bari.

**Privacy, anonymity and confidentiality**

All of the information we collect about your child will be kept private and confidential. We will keep all paper documents in a locked cabinet. We will not give any information about your child to anyone not involved in the study. Your child’s name will never be used in reports of this study.

**Future use of information**

If the information we collect needs to be used for future use by other researchers, we will not supply any personal information and will maintain strict privacy.

**Right not to participate**

You may choose to allow us to take swabs from your child’s nose and throat or not allow us to take these swabs. You may choose to ask us to stop taking the specimen or to discard the specimen before testing it. Refusal to participate will involve no penalty or loss of benefits at the hospital. Even if you do not allow us to take this specimen, your child will still receive the usual care at the hospital. Your child will not be denied any treatments or benefits for which he/she would otherwise be eligible. If you choose to allow us to take these swabs from your child, you may choose to allow us to store the specimens or not allow us to store the specimens. You may still participate in the study even if you refuse to allow us to store your child’s specimens. Refusal to allow us to store your child’s specimens will involve no penalty or loss of benefits at the hospital. Even if you do not allow us to store your child’s specimens, your child will still receive the usual care at the hospital. Your child will not be denied any treatments or benefits for which you would otherwise be eligible.

**Principle of compensation**

There is no cost to you or your child for allowing us to take a nose or throat specimen. There will also be no compensation to you or your child.

**Persons to Contact:**

If you have questions during the procedure, ask at any time. If you have any additional questions about the surveillance you may contact:

Dr. Stephen P. Luby, Programme on Infectious Diseases and Vaccine Sciences (PIDVS), ICDDR,B, Mohakhali, Dhaka 1212. Phone: 8860523-32 # 2502,

If you have questions about your rights in regards to being part of this research surveillance or if you think some harm has been done to you because of the surveillance you may contact:

Mr. M. A. Salam, Research and Project Support Department (RPSD), ICDDR,B, Mohakhali, Dhaka 1212. Phone: 9886489, 01711428989

If you agree to our proposal of obtaining a nose and/or throat specimen from your child, please indicate that by putting your signature or your left thumb impression at the specified space below.

Thank you for your cooperation.

_______________________________________ ____________________

Signature or left thumb impression Date

of attendant/Guardian

_______________________________________ ____________________

Signature or left thumb impression of the witness Date

_______________________________________ ___________________

Signature of the PI or his/her representative Date

# Appendix 4: Child Assent Form: Specimen Collection

**Protocol Number: 2009-004**

**Protocol Title:** Prevention of secondary transmission of human influenza by promoting handwashing with soap: The Bangladesh Interruption of Secondary Transmission of Influenza Study (BISTIS)

**Investigator’s name**: Dr. Stephen P. Luby

**Organization:** ICDDR,B

Name of Index Case Patient: _____________________________________

**Who are we?**

My name is ________ and I work for ____________.

**Why are we meeting with you?**

We want to tell you about a study that we are doing that involves people with certain symptoms: cough and/or sore throat and fever. These symptoms are associated with an illness called influenza. We are interested in studying this illness.

**Why are we doing this study?**

We are trying to understand factors that are connected to the spread of influenza within a bari. We want to know if the spread of this illness is connected to any specific behaviors.

**What will happen to you if you are in the study?**

For possible index case-patients ONLY

We ask you to allow one of our trained research personnel to put a swab into your nose. We will test this swab to test for the influenza virus.

A second nose swab and also a throat swab will then be taken. We will use these swabs to test for the type of influenza you may have. If you agree, we will store the material from the swabs for twenty years. We may test the swabs later for respiratory illnesses other than influenza. The results of the future testing will not affect medical care, and, thus, these results will not be reported. You can ask to have the material from your swabs removed from storage at any time. You can do this by calling Dr. Stephen P. Luby

at 8860523-32 # 2502.

For probable secondary/follow-up case-patients ONLY

We ask you to allow one of our trained research personnel to put a swab into your nose and throat. We will test these swabs to test for the influenza virus at a later time. If you agree, we will store the material from the swabs for twenty years. We may test the swabs later for respiratory illnesses other than influenza. The results of the future testing will not affect medical care, and, thus, these results will not be reported. You can ask to have the material from your swabs removed from storage at any time. You can do this by calling Dr. Stephen P. Luby at 8860523-32 # 2502.

**What are the good things and bad things that may happen to you?**

One of our trained research personnel will have to place a swab into your nose and throat; this may be uncomfortable, but this should not hurt you.

**Do you have to allow us to take this specimen?**

No, you do not. No one will get upset or angry with you if you do not want to do this. Just tell us if you do not want to take part. Remember, you can change your mind later if you decide you don’t want to take part in the study anymore. You also do not have to let us store your specimens. No one will get upset or angry with you if you do not want this. Just tell us if you do not want us to store your specimens. You can still take part in the study even if you do not want us to store your specimens. Remember, you can change your mind later if you decide you no longer want us to store your specimens.

**Do you have any questions?**

You can ask them at any time. You can ask now, or you can ask later. You can talk to me or you can talk to someone else at any time during the study. You can also call the person below.

Dr. Stephen P. Luby, Programme on Infectious Diseases and Vaccine Sciences (PIDVS), ICDDR,B, Mohakhali, Dhaka 1212. Phone: 8860523-32 # 2502

If you have questions about your rights in regards to being part of this research surveillance or if you think some harm has been done to you because of the surveillance you may contact:

Mr. M. A. Salam, Research and Project Support Department (RPSD), ICDDR,B, Mohakhali, Dhaka 1212. Phone: 9886489, 01711428989

If you want to take part in the study and allow us to take these specimens, please indicate that by putting your signature or your left thumb impression at the specified space below

Thank you for your cooperation

_______________________________________ ____________________

Signature or left thumb impression of subject Date

_______________________________________ ____________________

Signature or left thumb impression Date

of attendant/Guardian

_______________________________________ ____________________

Signature or left thumb impression of the witness Date

_______________________________________ ___________________

Signature of the PI or his/her representative Date

# Appendix 5: Consent Form: Study Enrollment, Household/Bari

**Protocol Number: 2009-004**

**Protocol Title:** Prevention of secondary transmission of human influenza by promoting handwashing with soap: The Bangladesh Interruption of Secondary Transmission of Influenza Study (BISTIS)

**Investigator’s name**: Dr. Stephen P. Luby

**Organization:** ICDDR,B

**Introduction**

Your Bari is invited to take part in a research trial. Scientists from the International Centre for Diarrhoeal Diseases Research, Bangladesh (ICDDR,B), Centers for Disease Control and Prevention (CDC), and University at Buffalo, a university in the USA, are doing a research study understand factors associated to the spread of influenza virus in a rural population.

**Purpose of the research**

We are trying to understand factors that are connected to the spread of influenza virus within a bari. We would also like to understand whether washing hands with soap prevents the spread of this illness. Around 400 baris will be part of the study. We will aim to find out whether the spread of influenza is linked to specific behaviors.

**Why selected**

One of the members of your Bari has been identified as having symptoms that are associated with influenza. This is why we are inviting your Bari to help us.

**What is expected from the members of your Bari?**

If you agree to enrolling your Bari in the study:

I will identify all the members within each bari.

We will also observe aspects of your Bari that may be related to the spread of influenza-like illness. We will ask questions about each household within the bari and observe the physical characteristics of the households.

We will visit your Bari every day until ten days after the symptoms of the person with influenza-like illness resolve. During those daily visits, we will ask about whether each member of your Bari has influenza-like symptoms.

During our visits, if a member of your bari shows symptoms of influenza-like illness, we will ask that person to allow us to take swabs from the nose and throat. These swabs will be used to test for influenza at a later time.

This study is an intervention trial. This means half the Baris will be taught some new behaviors and given soap during the time period we will be visiting the bari. The remaining Baris will be given soap after we complete the daily visits. The remaining Baris will also be taught the behaviors at a future time. Baris will be assigned to the group that will receive the teaching and soap randomly, so it is beyond our control when each bari receives the teaching and soap.

**Risk and benefits**

In people who have skin reactions to the soap available at the market, the soap we give you may cause similar reactions. These people should not use the soap we give you. The process of having someone visit your home may be uncomfortable to you. However, we do not expect any harm to come to you or your family because of being visited.

All Baris that take place in the study will receive the benefit of teaching and bars of soap; however there will be no other immediate benefits. However, this study will help us better understand what factors are associated with the spread of influenza within a bari. This study will also help us test prevention methods that may stop the spread of influenza. This study may help us understand how to stop the spread of influenza-like illness within baris in rural Bangladesh.

For those people who have symptoms and who allow us to take a nose and throat swab, one of our trained research personnel will have to place a swab into their nose and a different swab into their throat. This may be uncomfortable. There are no other known risks for this procedure.

The results of the influenza testing will not be available for one or more months after the specimen is collected. The results of the test will not alter in any way the treatment of the person who has influenza related symptoms.

**Privacy, anonymity and confidentiality**

All of the information we collect about the members of your community will be kept private and confidential. We will keep all data in a locked cabinet. We will not give any information about your community to anyone not involved in the study.

**Future use of information**

If the information we collect needs to be used for future use by other researchers, we will not supply any personal information and will maintain strict privacy.

**Right not to participate and withdraw**

You may choose to allow your Bari to take part or not to take part in this study. You may refuse to take part at any time. You may also withdraw your Bari from the study at any time. Refusal to participate or withdrawal from the study will involve no penalty or loss of benefits for the members of your community at the clinic or hospital. Even if you do not enroll your Bari in the study, everyone in your Bari will still receive the usual care at the clinic. Each individual in your Bari may choose to participate or not participate, and may choose to withdraw from the study at any time.

**Principle of compensation**

There is no cost to you or your bari for participation in this study. Other than receiving free soap, you will not receive any compensation for being in the study.

**Persons to Contact:**

If you have questions during the procedure, ask at any time. If you have any additional questions about the surveillance you may contact:

Dr. Stephen P. Luby, Programme on Infectious Diseases and Vaccine Sciences (PIDVS), ICDDR,B, Mohakhali, Dhaka 1212. Phone: 8860523-32 # 2502

If you have questions about your rights in regards to being part of this research surveillance or if you think some harm has been done to you because of the surveillance you may contact:

Mr. M. A. Salam, Research and Project Support Department (RPSD), ICDDR,B, Mohakhali, Dhaka 1212. Phone: 9886489, 01711428989

If you agree to enrolling your Bari in our study, please indicate that by putting your signature or your left thumb impression at the specified space below.

Thank you for your cooperation.

_______________________________________ ____________________

Signature or left thumb impression of subject Date

_______________________________________ ____________________

Signature or left thumb impression Date

of attendant/Guardian

_______________________________________ ____________________

Signature or left thumb impression of the witness Date

_______________________________________ ___________________

Signature of the PI or his/her representative Date

cwiwkó-2t cÖvßeq¯‹‡`i m¤§wZcÎt bgybv msMÖn

M‡elYv b¤^i: 2009-004

M‡elbvi bvgt mvevb w`‡q nvZ †avqvi gva¨‡g †ivM ciewZ© gvby‡li g‡a¨ msµwgZ Bbd¬z‡qÄv msµgb cÖwZ‡iva|

†ivM cieZx© gvby‡li g‡a¨ msµwgZ/gvbe Bbd¬z‡qÄv msµgb cÖwZ‡iva msµvš— M‡elbv evsjv‡`k (wemwUm)|

úªavb M†elK: Wvt w÷‡db wc. jzwŸ|

cÖwZôvbt Avš—©RvwZK D`ivgq M‡elYv †K›`ª, evsjv‡`k (AvBwmwWwWAvi,we)|

cÖv_wgK ‡ivMxi bvg t ÑÑÑÑÑÑÑÑÑÑÑÑÑÑÑÑÑÑÑÑÑÑÑÑÑÑÑÑÑÑÑÑÑÑÑÑÑÑÑ

BDwbK AvB wWt ÑÑÑÑÑÑÑÑÑÑÑÑÑÑÑÑÑÑÑÑÑÑÑÑÑÑÑÑÑÑÑÑÑÑÑÑÑÑÑÑÑÑÑÑ

M‡elYvi f~wgKvt

Avgiv Avgv‡`i M‡elbvq Ask MÖnY Kivi Rb¨ Avcbv‡K Avgš¿b Rvbvw”Q| evsjv‡`‡ki MÖvgvÂ‡ji gvby‡li g‡a¨ Bbd¬z‡qÄv fvBivm Qov‡bvi Rb¨ †Kvb †Kvb welq `vqx †m m¤ú‡K© Rvbvi Rb¨ evsjv‡`‡ki Avš—©RvwZK D`ivgq M‡elYv †K›`ª (AvBwmwWwWAvi,we), hy³iv‡óªi †ivM wbqš¿b I cÖwZ‡iva †K›`ª Ges Av‡gwiKv‡Z Aew¯’Z GwU ev‡d‡jv BDwbfvwm©wU mw¤§wjZfv‡e GKwU M‡elbv Ki‡Q|

## M‡elbvi D‡Ïk¨t

## GKUv evwoi g‡a¨ Bbd¬z‡qÄv fvBivm Qov‡bvi Rb¨ wK wK welq RwoZ Avgiv Zv eyS‡Z †Póv KiwQ| Avgiv Av‡iv eyS‡Z ‡Póv KiwQ †h, mvevb w`‡q nvZ ay‡q Bbd¬z‡qÄv fvBivmRwbZ †iv‡Mi we¯—vi †iva Kiv m¤¢e wKbv| cÖvq 400 evwo‡K Avgiv Avgv‡`i M‡elbvq Aš—©f~³ Kie| Avgv‡`i j¶¨ _vK‡e mywbw`©ó wKQz AvPib Bbd¬z‡qÄv msµvg‡bi mv‡_ RwoZ wKbv Zv Ly‡Ru †ei Kiv|

**Avcbv‡K †Kb GB M‡elYvq AskMÖn‡Yi Rb¨ Aby‡iva KiwQ?**

GB M‡elYvq AskMÖn‡Yi Rb¨ Avcbv‡K Avgiv Aby‡iva KiwQ KviY MZ 7 w`‡bi g‡a¨ R¡‡ii mv‡_ mv‡_ Avcbvi Kvuwk/Mjve¨_v DcmM© j¶¨ Kiv †M‡Q| GB j¶b ¸‡jv Bbd¬z‡qÄv bvgK Amy¯’Zvi mv‡_ RwoZ _vK‡Z cv‡i | Avgiv GB Amy¯’ZvwU wb‡q M‡elbv Ki‡Z AvMÖnx|

**‡ivMx / DËi`vZvi KvQ †_‡K Avgiv wK f~wgKv Avkv Kie?**

‡KejgvÎ m¤¢ve¨ cÖv_wgK †ivMxi †¶‡Î cÖ‡hvR¨t

Avgv‡`i GKRb cÖwkw¶Z M‡elbvKgx© Avcbvi bvK †_‡K bgybv msMÖn Ki‡e Ges Zv Bbd¬z‡qÄv fvBiv‡mi Rb¨ cix¶v Ki‡e| Avgiv Ge¨vcv‡i Avcbvi AbygwZ Kvgbv KiwQ|

wØZxqevi Avevi Avgiv Avcbvi bvK †_‡K Ges Mjv †_‡KI bgybv msMÖn Kie|AvgivGB bgybv¸‡jv †_‡K Bbd¬z‡qÄvi aib cix¶v K‡i ‡ei Ki‡ev, hv Avcbvi _vK‡Z cv‡i|

Avcwb ivwR _vK‡j Avgiv bgybvwU fwel¨‡Z cix¶v K‡i †`Lvi Rb¨ wek eQi msi¶b Kie Zvici Zv bó K‡i †djv n‡e| †mB cix¶v ¸‡jv n‡Z cv‡i Bbd¬z‡qÄv **Qvov** k¦vmZ‡š¿i Amy¯’Zvi Ab¨vb¨ Kvib Rvbvi Rb¨| fwel¨‡Z GB cix¶vi djvdj †Kvb fv‡e Avcbvi wPwKrmv‡K cÖfvweZ Ke‡e bv ZvB Avgiv Avcbv‡K †Kvb djvdjI cÖ`vb Kie bv| Avcwb msMÖwnZ bgybvwU †h †Kvb mgq msiw¶Z Ae¯’v †_‡K mwi‡q †dj‡Z ejvi Rb¨ †dvb Ki‡Z cv‡ib t Wvt w÷‡db wc. jzwŸ, 8860523-32 #2502|

‡KejgvÎ cieZx~‡Z AvµvšÍ †ivMx†`i †¶‡Î cÖ‡hvR¨t

Avgv‡`i GKRb cÖwkw¶Z M‡elbvKgx© Avcbvi Mjv Ges bvK †_‡K bgybv msMÖn Ki‡e,Avwg G e¨vcv‡I Avcbvi AbygwZ cÖv_©bv KiwQ| GB bgybv mg~y‡n Bbd¬y‡qÄv Av‡Q wKbv Zv cix¶v K‡i †`Lv n‡e cieZx© mg‡q|

Avcwb ivwR _vK‡j Avgiv bgybvwU fwel¨‡Z cix¶v K‡i †`Lvi Rb¨ wek eQi msi¶b Kie Zvici Zv bó K‡i †djv n‡e| †mB cix¶v ¸‡jv n‡Z cv‡i Bbd¬z‡qÄv **Qvov** k¦vmZ‡š¿i Amy¯’Zvi Ab¨vb¨ Kvib Rvbvi Rb¨| fwel¨‡Z GB cix¶vi djvdj †Kvb fv‡e Avcbvi wPwKrmv‡K cÖfvweZ Ke‡e bv ZvB Avgiv Avcbv‡K †Kvb djvdjI cÖ`vb Kie bv| Avcwb msMÖwnZ bgybvwU †h †Kvb mgq msiw¶Z Ae¯’v †_‡K mwi‡q †dj‡Z ejvi Rb¨ †dvb Ki‡Z cv‡ib t Wvt w÷‡db wc. jzwŸ, 8860523-32 #2502|

**SzwKu Ges myweav:**

Avgiv Avcbvi Mjv I bvvK †_‡K gv_vq Zzjv c¨uvPv‡bv GKwU KvwVi mvnv‡h¨ wKQy bgybv msMÖn Kie| G‡Z Avcbvi wKQyUv A¯^w¯— n‡Z cv‡i; wKš‘ c×wZUv wbivc` Ges G‡Z Avcbvi †Kvb ¶wZi m¤¢vebv †bB| GB cix¶vwU Kivi Rb¨ Avcbvi †Kvb mywbw`©ó myweav †bB| †h‡nZz GB cix¶vi djvdj †Kvb fv‡e Avcbvi wPwKrmv‡K cÖfvweZ Ke‡e bv Avgiv Avcbv‡K †Kvb djvdjI cÖ`vb Kie bv| Z‡e Avcbvi KvQ †_‡K msMÖwnZ bgybv Avgv‡`i eyS‡Z mvnvh¨ Ki‡e wKfv‡e GKwU evoxi g‡a¨ Bbd¬z‡qÁv Qovq|

**‡MvcbxqZv:**

Avcbvi †`qv mKj Z_¨ ‡Mvcb ivLv n‡e| Avcbvi mg¯— Z_¨ I cix¶v- wbix¶vi djvdj dvBjeÜx K‡i ivLv n‡e| M‡elYvi Kv‡R mswk­ó bq Ggb KvD‡K Avgiv Avcbvi Z_¨ †`e bv| Avcbvi bvg KLbB M‡elbvi djvd‡j cÖKvwkZ n‡e bv|

fwel¨‡Z Z‡_¨i e¨envi t

Avgiv †h Z_¨ msMÖn Ki‡ev Zv fwel¨‡Z Ab¨ M‡elK e¨envi Ki‡Z Pvq Z‡e †Kvb e¨w³MZ Z_¨ Zv‡`i †`e bv G&es K‡Vvi fv‡e †MvcbxqZv i¶v Kie|

**M‡elYvq AskMÖnb bv Kiv Ges cÖZ¨vnv‡ii AwaKvi:**

Avcwb Avcbvi Mjv I bvvK †_‡K bgybv msMÖn Kivi AbygwZ w`‡Z cv‡ib Avevi bvI w`‡Z cv‡ib| Avcwb Avcbvi KvQ †_‡K bgybv msMÖn Kiv eÜ K‡i w`‡Z cv‡ib A_ev msMÖnK…Z bgybv cix¶v Kivi c~‡e© †d‡j w`‡ZI ej‡Z cv‡ib| Avcwb GB M‡elYvq AskMÖn‡b Am¤§Z n‡jI Gi Rb¨ Avcbv‡K †Kvb kvw¯Í ev Rwigvbv w`‡Z n‡e bv Ges nvmcvZv‡j cÖvc¨ myweav †_‡KI Avcwb ewÂZ n‡eb bv| GgbwK Avcwb Avgv‡`i‡K bgybv msMÖn Kivi AbygwZ bv w`‡jI nvmcvZvj †_‡K h_vh_ †mev cv‡eb| Avcwb wPwKrmv †mev A_ev Ab¨vb¨ my‡hvM-myweav †_‡K ewÂZ n‡eb bv|

**¶wZc~iY ev cÖ‡`q:**

bvK I Mjv †_‡K bgybv msMÖn Ki‡Z †`Iqvi Rb¨ Avcbvi †Kvb LiP †bB| GKBfv‡e Avcbv‡K †Kvb ¶wZc~ibI †`qv n‡e bv|

**‡hvMv‡hvMi e¨vw³t**

GB cÖwµqv PjvKvjxb Avcbvi †Kvb cÖkœ _vK‡j †h †Kvb mgq Ki‡Z cv‡ib| hw` Avcbvi M‡elbv m¤ú‡K© †Kvb AwZwi³ cÖkœ _v‡K Avcwb †hvMv‡hvM Ki‡Z cv‡ib t

Wvt w÷‡db wc. jzwŸ, †cÖvMÖvg Ab Bb‡dKkvm wWwRR G¨vÛ †fKwmb mvB‡Ým (wc,AvvB.wW.wf.Gm) AvB.wm.wW.wW.Avi.we, gnvLvjx, XvKv 1212, †dvb t 8860523-32 # 2502|

GB M‡elbv Ask wnmv‡e Avcbvi AwaKvi m¤ú‡K© hw` Avcbvi †Kvb cÖkœ _v‡K A_ev hw` M‡elbvi Rb¨ Avcbvi †Kvb ¶wZ n‡q‡Q e‡j g‡b nq Zvn‡j †hvMv‡hvM Ki“b t

wgt Gg.G.mvjvg, wimvP© G¨Û cÖ‡R± mv‡cv©U wWcvU©‡g›U (Avi.wc.Gm.wW) AvB.wm.wW.wW.Avi.we, gnvLvjx, XvKv– 1212, †dvb t 9886489, # 01711428989|

hw` Avcwb/Avcbvi †ivMx hw` Avgv‡`i bvK I Mjv †_‡K bgybv msMÖ‡ni cÖ¯Ív‡e ivwR _v‡Kvb Zvn‡j AbyMÖn K‡i i Avcbvi ¯^v¶i w`‡q A_ev Avcbvi evg nv‡Zi ey‡ov Av½y‡ji Qvc w`‡q Zv wb‡`©k Ki“b|

Avcbvi mn‡hvMxZvi Rb¨ ab¨ev`|

___________________________________________

¯^v¶i/ AskMÖnbKvixi evg nv‡Zi e„Øv½yjxi Qvc ZvwiL

___________________________________________

¯^v¶xi ¯^v¶vi / A_ev evg nv‡Zi e„Øv½yjxi Qvc ZvwiL

___________________________________________

cÖavb M‡elK ev Zvi cÖwZwbwai ¯^v¶i ZvwiL

cwiwkó-3t wcZv gvZv A_ev AwffveK‡`i m¤§wZcÎt bgybv msMÖn

M‡elYv b¤^i: 2009-004

M‡elbvi bvgt mvevb w`‡q nvZ †avqvi gva¨‡g †ivM ciewZ© Bbd¬z‡qÄv msµgb cÖwZ‡iva|

†ivM cieZx© Bbd¬z‡qÄv msµgb cÖwZ‡iva msµvš— M‡elbv evsjv‡`k (wemwUm)|

úªavb M†elK: Wvt w÷‡db wc. jzwŸ|

cÖwZôvbt Avš—©RvwZK D`ivgq M‡elYv †K›`ª, evsjv‡`k (AvBwmwWwWAvi,we)|

cÖv_wgK ‡ivMxi bvg t ÑÑÑÑÑÑÑÑÑÑÑÑÑÑÑÑÑÑÑÑÑÑÑÑÑÑÑÑÑÑÑÑÑÑÑÑÑÑÑ

BDwbK AvB wWt ÑÑÑÑÑÑÑÑÑÑÑÑÑÑÑÑÑÑÑÑÑÑÑÑÑÑÑÑÑÑÑÑÑÑÑÑÑÑÑÑÑÑÑÑ

M‡elYvi f~wgKvt

Avgiv Avgv‡`i M‡elbvq Ask MÖnY Kivi Rb¨ Avcbvi wkï‡K Avgš¿b Rvbvw”Q| evsjv‡`‡ki MÖvgvÂ‡ji gvby‡li g‡a¨ Bbd¬z‡qÄv fvBivm Qov‡bvi Rb¨ †Kvb †Kvb welq `vqx †m m¤ú‡K© Rvbvi Rb¨ evsjv‡`‡ki Avš—©RvwZK D`ivgq M‡elYv †K›`ª (AvBwmwWwWAvi,we), hy³iv‡óªi †ivM wbqš¿b I cÖwZ‡iva †K›`ª Ges ev‡d‡jv BDwbfvwm©wUi M‡elKiv wg‡j mw¤§wjZfv‡e GKwU M‡elbv Ki‡Q|

## **M‡elbvi D‡Ïk¨t**

## GKUv evwoi g‡a¨ Bbd¬z‡qÄv fvBivm Qov‡bvi Rb¨ wK wK welq RwoZ Avgiv Zv eyS‡Z †Póv KiwQ| Avgiv Av‡iv eyS‡Z ‡Póv KiwQ †h, mvevb w`‡q nvZ ay‡q Bbd¬z‡qÄv fvBivmRwbZ †iv‡Mi we¯—vi †iva Kiv m¤¢e wKbv| cÖvq 200 evwo‡K Avgiv Avgv‡`i M‡elbvq Aš—f~©³ Kie| Bbd¬z‡qÄv we¯—v‡ii mv‡_ wbw`©ó †Kvb AvPib RwoZ wKbv Zv Lyu‡R †ei Kivi j‡¶¨ Avgiv KvR Kie|

‡Kb g‡bvwbZ Kiv n‡q‡Q ?

GB M‡elYvq AskMÖn‡Yi Rb¨ **Avcbvi wkï‡K** Avgiv Aby‡iva KiwQ Kvib MZ 7 w`‡bi g‡a¨ R¡‡ii mv‡_ mv‡_ Zvi Kvwk/Mjve¨_v DcmM© j¶¨ Kiv †M‡Q| Zvi G mKj DcmM© Bbd¬z‡qÄv bvgK Amy¯’Zvi mv‡_ m¤úwK©Z Avgiv GB Amy¯’ZvwU wb‡q M‡elbv Ki‡Z AvMÖnx|

**‡ivMx / DËi`vZvi KvQ †_‡K Avgiv wK f~wgKv Avkv Kie ?**

‡KejgvÎ m¤¢ve¨ cÖv_wgK †ivMxi †¶‡Î cÖ‡hvR¨t

Avgv‡`i GKRb cÖwkw¶Z M‡elbvKgx© Avcbvi wkïi bvK †_‡K bgybv msMÖn Ki‡e Ges Zv Bbd¬z‡qÄv fvBiv‡mi Rb¨ cix¶v Ki‡e| Avgiv G e¨vcv‡i Avcbvi AbygwZ Kvgbv KiwQ|

Avgiv wØZxqevi Avevi I Zvi bvK I Mjv †_‡K bgybv msMÖn Kie| Avcbvi wkï wK ai‡bi Bbd¬y‡qÄv Øviv AvµvšÍ n‡q _vK‡Z cv‡i Zv GB bgybv w`‡q cix¶v Kiv n‡e|

Avcwb ivwR _vK‡j Avgiv bgybvwU fwel¨‡Z cix¶v K‡i †`Lvi Rb¨ wek eQi msi¶b Kie Zvici Zv bó K‡i †djv n‡e| †mB cix¶v ¸‡jv n‡Z cv‡i Bbd¬z‡qÄv **Qvov** k¦vmZ‡š¿i Amy¯’Zvi Ab¨vb¨ Kvib Rvbvi Rb¨| fwel¨‡Z GB cix¶vi djvdj †Kvb fv‡e Avcbvi wkïi wPwKrmv‡K cÖfvweZ Ke‡e bv ZvB Avgiv Avcbv‡K †Kvb djvdjI cÖ`vb Kie bv| Avcwb msMÖwnZ bgybvwU †h †Kvb mgq msiw¶Z Ae¯’v †_‡K mwi‡q †dj‡Z ejvi Rb¨ †dvb Ki‡Z cv‡ib t Wvt w÷‡db wc. jzwŸ, 860523-32 #2502|

‡KejgvÎ cieZx©‡Z AvµvšÍ †ivMx†`i †¶‡Î cÖ‡hvR¨t

Avgv‡`i GKRb cÖwkw¶Z M‡elbvKgx© Avcbvi wkïi bvK I Mjv †_‡K bgybv msMÖn Ki‡e,Avwg G e¨vcv‡iI Avcbvi AbygwZ cÖv_©bv KiwQ| GB bgybv mg~y‡n Bbd¬y‡qÄv Av‡Q wKbv Zv cix¶v K‡i †`Lv n‡e|

Avcwb ivwR _vK‡j Avgiv bgybvwU fwel¨‡Z cix¶v K‡i †`Lvi Rb¨ wek eQi msi¶b Kie Zvici Zv bó K‡i †djv n‡e| †mB cix¶v ¸‡jv n‡Z cv‡i Bbd¬z‡qÄv **Qvov** k¦vmZ‡š¿i Amy¯’Zvi Ab¨vb¨ Kvib Rvbvi Rb¨| fwel¨‡Z GB cix¶vi djvdj †Kvb fv‡e Avcbvi wkïi wPwKrmv‡K cÖfvweZ Ke‡e bv ZvB Avgiv Avcbv‡K †Kvb djvdjI cÖ`vb Kie bv| Avcwb msMÖwnZ bgybvwU †h †Kvb mgq msiw¶Z Ae¯’v †_‡K mwi‡q †dj‡Z ejvi Rb¨ †dvb Ki‡Z cv‡ib t Wvt GWyqv©Wy AvwRR †evgMvU©bvi | 8860523-32 #2500|

**SzwKu Ges myweav:**

Avgiv Avcbvi Mjv I bvvK †_‡K gv_vq Zzjv c¨uvPv‡bv GKwU KvwVi mvnv‡h¨ wKQy bgybv msMÖn Kie| G‡Z Avcbvi wKQyUv A¯^w¯— n‡Z cv‡i; wKš‘ c×wZUv wbivc` Ges G‡Z Avcbvi †Kvb ¶wZi m¤¢vebv †bB| GB cix¶vwU Kivi Rb¨ Avcbvi †Kvb mywbw`©ó myweav †bB| †h‡nZz GB cix¶vi djvdj †Kvb fv‡e Avcbvi wPwKrmv‡K cÖfvweZ Ke‡e bv Avgiv Avcbv‡K †Kvb djvdjI cÖ`vb Kie bv| Z‡e Avcbvi KvQ †_‡K msMÖwnZ bgybv Avgv‡`i eyS‡Z mvnvh¨ Ki‡e wKfv‡e GKwU evoxi g‡a¨ Bbd¬z‡qÁv Qovq|

**‡MvcbxqZv:**

Avcbvi wkïi/Avcbvi mKj Z_¨ hv Avgiv msMÖn K‡iwQ Zv †Mvcb ivLv n‡e| mg¯— Z_¨ I cix¶v- wbix¶vi djvdj dvBjeÜx K‡i ivLv n‡e| M‡elYvi mv‡_ RwoZ bq Ggb KvD‡K Avcbvi wkïi †Kvb Z_¨ †`Iqv n‡e bv| M‡elYv welqK †Kvb Av‡jvPbvq wKsev M‡elbvi dj cÖKv‡ki mgq Avcbvi wkïi bvg KL‡bv e¨eüZ n‡e bv|

Z_¨mg~‡ni fwel¨Z e¨envi:

hw` Avgv‡`i msMªwnZ Z_¨ fwel¨‡Z Ab¨ M‡elK†`i cª‡qvRb nq Zvn‡j Avgiv Avcbv‡`i †Kvb e¨vw³MZ Z_¨ Zv‡`i‡K †`e bv Ges KwVb †MvcbxqZv i¶v Kie|

**M‡elYvq AskMÖnb bv Kiv Ges cÖZ¨vnv‡ii AwaKvi:**

Avcwb Avcbvi wkïi Mjv I bvK †_‡K bgybv msMÖn Kivi AbygwZ w`‡Z cv‡ib Avevi bvI w`‡Z cv‡ib| Avcwb Avcbvi wkïi KvQ †_‡K bgybv msMÖn Kiv eÜ K‡i w`‡Z cv‡ib A_ev msMÖnK…Z bgybv cix¶v Kivi c~‡e© †d‡j w`‡ZI ej‡Z cv‡ib| Avcwb Avcbvi wkï‡K GB M‡elYvq AskMÖnb Ki‡Z w`‡Z Am¤§Z n‡jI Gi Rb¨ Avcbv‡K †Kvb kvw¯Í ev Rwigvbv w`‡Z n‡e bv Ges nvmcvZv‡j cÖvc¨ myweav †_‡KI Avcbvi wkï ewÂZ n‡e bv| GgbwK Avcwb Avgv‡`i‡K bgybv msMÖn Kivi AbygwZ bv w`‡jI Avcbvi wkï nvmcvZvj †_‡K h_vh_ †mev cv‡e| †m wPwKrmv †mev A_ev Ab¨vb¨ my‡hvM-myweav †_‡K ewÂZ n‡e bv|

**¶wZc~iY ev cÖ‡`q:**

bvK I Mjv †_‡K bgybv msMÖn Ki‡Z †`Iqvi Rb¨ Avcbv‡K/ Avcbvi wkïi †Kvb LiP †bB| GKBfv‡e Avcbv‡K ev Avcbvi wkï‡K I †Kvb ¶wZc~ib †`qv n‡e bv|

**‡hvMv‡hvMi e¨vw³t**

GB cÖwµqv PjvKvjxb Avcbvi †Kvb cÖkœ _vK‡j †h †Kvb mgq Ki‡Z cv‡ib| hw` Avcbvi M‡elbv m¤ú‡K© †Kvb AwZwi³ cÖkœ _v‡K Avcwb †hvMv‡hvM Ki‡Z cv‡ib t

Wvt w÷‡db wc. jzwŸ, †cÖvMÖvg Ab Bb‡dKkvm wWwRR G¨vÛ †fKwmb mvB‡Ým (wc,AvvB.wW.wf.Gm) AvB.wm.wW.wW.Avi.we, gnvLvjx, XvKv 1212, †dvb t 8860523-32 # 2502|

GB M‡elbv Ask wnmv‡e Avcbvi AwaKvi m¤ú‡K© hw` Avcbvi †Kvb cÖkœ _v‡K A_ev hw` M‡elbvi Rb¨ Avcbvi †Kvb ¶wZ n‡q‡Q e‡j g‡b nq Zvn‡j †hvMv‡hvM Ki“b t

wgt Gg.G.mvjvg, wimvP© G¨Û cÖ‡R± mv‡cv©U wWcvU©‡g›U (Avi.wc.Gm.wW) AvB.wm.wW.wW.Avi.we, gnvLvjx, XvKv– 1212, †dvb t 9886489, # 01711428989|

hw` Avcwb/Avcbvi †ivMx hw` Avgv‡`i bvK I Mjv †_‡K bgybv msMÖ‡ni cÖ¯Ív‡e ivwR _v‡Kvb Zvn‡j AbyMÖn K‡i i Avcbvi ¯^v¶i w`‡q A_ev Avcbvi evg nv‡Zi ey‡ov Av½y‡ji Qvc w`‡q Zv wb‡`©k Ki“b|

Avcbvi mn‡hvMxZvi Rb¨ ab¨ev`|

_______________________________________

ïkªlvKvix / AwffveK Gi evg nv‡Zi e„Øv½yjxi Qvc ZvwiL

___________________________________________

¯^v¶xi ¯^v¶vi / A_ev evg nv‡Zi e„Øv½yjxi Qvc ZvwiL

___________________________________________

cÖavb M‡elK ev Zvi cÖwZwbwai ¯^v¶i ZvwiL

cwiwkó-4t wkï‡`i m¤§wZcÎt bgybv msMÖn

M‡elYv b¤^i: 2009-004

M‡elbvi bvgt mvevb w`‡q nvZ †avqvi gva¨‡g †ivM ciewZ© Bbd¬z‡qÄv msµgb cÖwZ‡iva|

†ivM cieZx© Bbd¬z‡qÄv msµgb cÖwZ‡iva msµvš— M‡elbv evsjv‡`k (wemwUm)|

úªavb M†elK: Wvt w÷‡db wc. jzwŸ|

cÖwZôvbt Avš—©RvwZK D`ivgq M‡elYv †K›`ª, evsjv‡`k (AvBwmwWwWAvi,we)|

Avgiv Kviv?

Avgvi bvg_______________ Ges Avwg KvR Kwi_______________

Avgiv †Kb ‡Zvgvi mv‡_ mv¶vr KiwQ?

‡hme †jv‡Ki wbw`©ó wKQy DcmM© t Kvwk I/ A_ev Mjve¨_v Ges R¡i †`Lv hvq Avgiv Zv‡`i wb‡q GKwU M‡elYv cwiPvjbv KiwQ| GmKj DcmM© Bbd¬z‡qÄv bvgK Amy¯’Zvi mv‡_ m¤úwK©Z Avgiv GB Amy¯’ZvwU wb‡q M‡elbv Ki‡Z AvMÖnx|

Avgiv †Kb GB M‡elYv Kg©wU cwiPvjbv KiwQ?

## GKUv evwoi g‡a¨ Bbd¬z‡qÄv fvBivm Qov‡bvi Rb¨ wK wK welq RwoZ Avgiv Zv eyS‡Z †Póv KiwQ| Bbd¬z‡qÄv

## we¯—v‡ii mv‡_ wbw`©ó †Kvb AvPib RwoZ wKbv Avgiv Zv Rvb‡Z PvB|

GB M‡elYvq _vK‡j †Zvgvi wK n‡e?

ïaygvÎ m¤¢ve¨ cÖv_wgK †ivMxi †¶‡Î-

GKRb cÖwkw¶Z M‡elbvKgx© †Zvgvi bvK †_‡K bgybv msMÖn Ki‡e|Avwg †m e¨vcv‡i †Zvgvi AbygwZ Pvw”Q| GB bgybvwU Bbd¬z‡qÄv fvBivm Gi Rb¨ cix¶v Kie|

wØZxqevi AveviI †Zvgvi bvK Ges Mjv †_‡KI bgybv msMÖn Kie|Avgiv GB bgybv ¸‡jv †_‡K †Zvgvi wK ai‡bi **Bbd¬z‡qÄv** n‡q _vK‡Z cv‡i Zv cix¶v K‡i †`L‡ev**|**

Zzwg ivwR _vK‡j Avgiv bgybvwU fwel¨‡Z cix¶v K‡i †`Lvi Rb¨ wek eQi msi¶b Kie Zvici Zv bó K‡i †djv n‡e| Avgiv cieZx©‡Z GB bgybv¸‡jv Bbd¬z‡qÄv **Qvov** k¦vmZ‡š¿i Ab¨vb¨ Amy¯’Zv Rvbvi Rb¨ cix¶v Kie | fwel¨‡Z GB cix¶vi djvdj †Kvb fv‡e †Zvgvi wPwKrmv‡K cÖfvweZ Ke‡e bv ZvB Avgiv †Zvgv‡K †Kvb djvdjI cÖ`vb Kie bv| Zywg msMÖwnZ bgybvwU †h †Kvb mgq msiw¶Z Ae¯’v †_‡K mwi‡q †dj‡Z ejvi Rb¨ †dvb Ki‡Z cvi t

Wvt w÷‡db wc. jzwŸ, 8860523-32 #2502|

‡KejgvÎ cieZx©‡Z AvµvšZ †ivMxi †¶‡Î cÖ‡hvh¨:

Avgv‡`i GKRb cÖwkw¶Z M‡elbvKgx© †Zvgvi Mjv Ges bvK †_‡K bgybv msMÖn Ki‡e| Avwg †m e¨vcv‡i †Zvgvi AbygwZ Pvw”Q| GB bgybv mgy‡n Bbd¬z‡qÄv fvBivm Av‡Q wKbv Zv cix¶v K‡i †`Le|

Zzwg ivwR _vK‡j Avgiv bgybvwU fwel¨‡Z cix¶v K‡i †`Lvi Rb¨ wek eQi msi¶b Kie Zvici Zv bó K‡i †djv n‡e| †mB cix¶v ¸‡jv n‡Z cv‡i Bbd¬z‡qÄv **Qvov** k¦vmZ‡š¿i Amy¯’Zvi Ab¨vb¨ Kvib Rvbvi Rb¨| fwel¨‡Z GB cix¶vi djvdj †Kvb fv‡e †Zvgvi wPwKrmv‡K cÖfvweZ Ke‡e bv ZvB Avgiv †Zvgv‡K †Kvb djvdjI cÖ`vb Kie bv| Zywg msMÖwnZ bgybvwU †h †Kvb mgq msiw¶Z Ae¯’v †_‡K mwi‡q †dj‡Z ejvi Rb¨ †dvb Ki‡Z cvi t

Wvt w÷‡db wc. jzwŸ, 8860523-32 #2502|

fvj ev Lvivc wRwbm¸‡jv wK hv †Zvgvi n‡Z cv‡i?

Avgv‡`i GKRb cÖwkw¶Z M‡elbvKgx© †Zvgvi Mjv Ges bvK †_‡K gv_vq Zzjv c¨uvPv‡bv GKwU KvwVi mvnv‡h¨ wKQy bgybv msMÖn Ki‡e| G‡Z †Zvgvi wKQyUv A¯^w¯— n‡Z cv‡i; wKš‘ Zzwg e¨v_v cv‡e bv|

Zzwg wK Avgv‡`i‡K bgybv msMÖn Ki‡Z w`‡Z ivRx?

bv , Zzwg bgybv msMÖn Ki‡Z w`‡Z ivRx bv| Zzwg bgybv msMÖn Ki‡Z w`‡Z bv PvB‡j †KD ‡Zvgvi cÖwZ gb Lvivc Ki‡e bv ev ivMvwš^Z n‡e bv| AskMÖnY Ki‡Z bv PvB‡j Avgv‡`i‡K ej‡Z cv‡iv| g‡b ‡i‡Lv, Zzwg PvB‡j

c‡iI gZ e`jv‡Z cv‡iv hw` Zzwg wmavšÍ wb‡q _vK †h Avi M‡elYvq AskMÖnY Ki‡e bv|

†Zvgvi wK †Kvb cÖkœ Av‡Qt

Zzwg Zv wbw×©avq †h‡Kvb mgq cÖkœ Ki†Z cv‡ib| Zzwg Zv GLb A_ev c‡iI wR‡Ám Ki‡Z cv‡iv| M‡elYv PjvKv‡j Zzwg Avgvi mv‡_ A_ev Ab¨ Kv‡iv mv‡_ K_v ej‡Z cv‡iv| †Zvgvi Av‡iv wKQy Rvbvi _vK‡j Zzwg wbæwjwLZ e¨w³i mv‡_ †hvMv‡hvM Ki‡Z cv‡ivt

Wvt w÷‡db wc. jzwŸ, †cÖvMÖvg Ab Bb‡dKkvm wWwRR G¨vÛ †fKwmb mvB‡Ým (wc,AvvB.wW.wf.Gm) AvB.wm.wW.wW.Avi.we, gnvLvjx, XvKv 1212, †dvb t 8860523-32 # 2502|

GB M‡elbv Ask wnmv‡e Avcbvi AwaKvi m¤ú‡K© hw` Avcbvi †Kvb cÖkœ _v‡K A_ev hw` M‡elbvi Rb¨ Avcbvi †Kvb ¶wZ n‡q‡Q e‡j g‡b nq Zvn‡j †hvMv‡hvM Ki“b t

wgt Gg.G.mvjvg, wimvP© G¨Û cÖ‡R± mv‡cv©U wWcvU©‡g›U (Avi.wc.Gm.wW) AvB.wm.wW.wW.Avi.we, gnvLvjx, XvKv– 1212, †dvb t 9886489, # 01711428989|

# Zzwg hw` M‡elYvq Ask wb‡Z Ges bgybv msMÖn Ki‡Z w`‡Z ivRx _v‡Kv, Zvn‡j AbyMÖn K‡i bx‡P ‡Zvgvi ¯^v¶i A_ev evg nv‡Zi e„×v½ywji Qvc `vI:

# ‡Zvgvi mn‡hvwMZvi Rb¨ ab¨ev`|

__________________________________ ____________________

AskMÖnYKvixi ¯^v¶i/evg nv‡Zi e„×v½yjxi Qvc ZvwiL

__________________________________ ____________________

Awffve‡Ki ¯^v¶i/evg nv‡Zi e„×v½yjxi Qvc ZvwiL

__________________________________ ____________________

mv¶xi ¯^v¶i/evg nv‡Zi e„×v½yjxi Qvc ZvwiL

__________________________________ ___________________

M‡elK/cÖwZwbwai ¯^v¶i ZvwiL

cwiwkó-5t m¤§wZcÎt M‡elbv ZvwjKvf~w³Kib, Lvbv/ evwo

M‡elYv b¤^i: 2009-004

M‡elbvi bvgt mvevb w`‡q nvZ †avqvi gva¨‡g †ivM ciewZ© Bbd¬z‡qÄv msµgb cÖwZ‡iva|

†ivM cieZx© Bbd¬z‡qÄv msµgb cÖwZ‡iva msµvš— M‡elbv evsjv‡`k (wemwUm)|

úªavb M†elK: Wvt w÷‡db wc. jzwŸ|

cÖwZôvbt Avš—©RvwZK D`ivgq M‡elYv †K›`ª, evsjv‡`k (AvBwmwWwWAvi,we)|

M‡elYvi f~wgKvt

Avgiv Avgv‡`i M‡elbvq Ask MÖnY Kivi Rb¨ Avcbvi evwo‡K Avgš¿b Rvbvw”Q| evsjv‡`‡ki MÖvgvÂ‡ji gvby‡li g‡a¨ Bbd¬z‡qÄv fvBivm Qov‡bvi Rb¨ †Kvb †Kvb welq `vqx †m m¤ú‡K© Rvbvi Rb¨ evsjv‡`‡ki Avš—©RvwZK D`ivgq M‡elYv †K›`ª (AvBwmwWwWAvi,we), hy³iv‡óªi †ivM wbqš¿b I cÖwZ‡iva †K›`ª Ges ev‡d‡jv BDwbfvwm©wUi M‡elKiv wg‡j mw¤§wjZfv‡e GKwU M‡elbv Ki‡Q|

## M‡elbvi D‡Ïk¨t

## GKUv evwoi g‡a¨ Bbd¬z‡qÄv fvBivm Qov‡bvi Rb¨ wK wK welq RwoZ Avgiv Zv eyS‡Z †Póv KiwQ| Avgiv Av‡iv eyS‡Z ‡Póv KiwQ †h, mvevb w`‡q nvZ ay‡q Bbd¬z‡qÄv fvBivmRwbZ †iv‡Mi we¯—vi †iva Kiv m¤¢e wKbv| cÖvq 400 evwo‡K Avgiv Avgv‡`i M‡elbvq Aš—©f~³ Kie| Avgv‡`i j¶¨ _vK‡e mywbw`©ó wKQz AvPib Bbd¬z‡qÄv msµvg‡bi mv‡_ RwoZ wKbv Zv Ly‡Ru †ei Kiv|

**Avcbvi evwo‡K †Kb GB M‡elYvq AšÍ©f~³ Kiv n‡q‡Q?**

Avcbvi evwoi GKRb m`‡m¨i g‡a¨ **Bbd¬z‡qÄv †iv‡Mi j¶Y/DcmM©** cvIqv †M‡Q| ZvB Avcbvi evwo‡K Avgv‡`i‡K mn‡hvwMZv Kivi Rb¨ Aby‡iva KiwQ|

**Avcbvi evwoi m`‡m¨i Kv‡Q Avgiv wK cÖZ¨vkv Kie?**

**Avcwb hw` Avcbvi evwo‡K GB M‡elYvq AšÍ©f~³ Ki‡Z Pvbt**

**Avwg cÖwZwU Lvbvi mKj m`m¨‡K wPwýZ Kie|**

**Avgiv Avcbvi evwoi wewfbœ w`K ch©‡e¶b Kie hv wKbv Bbd¬z‡qÄv †iv‡Mi j¶Y Qov‡bvi mv‡_ RwoZ _vK‡Z cv‡i| Avgiv evwoi cÖwZwU Lvbv m¤ú‡K© cÖkœ wR‡Ám Kie Ges H Lvbvi kvixwiK ˆewkó¨mg~n ch©‡e¶b Kie|**

**Bbd¬z‡qÄv AvµvšÍ e¨w³i j¶bmgyn K‡g hvIqvi w`b †_‡K Av‡iv 10 w`b cÖwZw`b Avgiv Avcbvi evwo cwi`k©b Ki‡ev| GB w`b¸‡jv‡Z Avgiv Avcbvi evwoi Ab¨ †Kvb e¨w³i g‡a¨ Bbd¬z‡qÄvi DcmM© †`Lv w`‡q‡Q wKbv Zv wR‡Ám Ki‡ev|**

**Avgv‡`i GB cwi`k©‡bi mgq hw` evwoi †Kvb m`‡m¨i g‡a¨ Bbd¬z‡qÄv †iv‡Mi j¶Y/DcmM© j¶¨ Kiv hvq, Zvn‡j Avgiv Zv‡K Zvi bvK I Mjv †_‡K bgybv msMÖn Kivi AbygwZ w`‡Z Aby‡iva Rvbve| GB** bgybv Bbd¬z‡qÄv fvBiv‡mi Rb¨ cix¶v Ki‡e|

**GB M‡elYvwU GKwU ga¨eZx© cix¶b c×wZ| Zvi gv‡b wKQy evwo‡Z KZK¸‡jv bZzb AvPib wkLv‡bv n‡e Ges mvevb †`Iqv n‡e evwo cwi`k©‡bi mgq | Avi evwK evwo¸‡jv‡Z mvevb †`Iqv n‡e Avgv‡`i cÖwZw`‡bi evox cwi`k©b KvR †kl Kivi c‡i evwK evwo¸‡jv‡Z fwel¨‡Z AvPib ¸‡jv wkLv‡bv n‡e| ‡hme evwo‡Z bZzb AvPib wkLv‡bv n‡e Ges mvevb †`Iqv n‡e ‡mB evwo¸‡jv ‰`e Pq‡bi wfwË‡Z (jUvix) `‡j wef³ Kiv n‡e| ZvB †Kvb evwo¸‡jv‡Z bZzb AvPib wkLv‡bv n‡e Ges mvevb †`Iqv n‡e ‡mUv Avgv‡`i wbqš¿‡bi evB‡i|**

**SzwKu Ges myweav:**

**evRv‡i cvIqv mvevb e¨env‡i hv‡`i Pvgovq cvk¦©- cÖwZwµqv †`Lv †`q, Avgv‡`i ‡`Iqv mvevb e¨env‡iI Zv‡`i GKB ai‡bi cvk¦©- cÖwZwµqv †`Lv w`‡Z cv‡i| Gai‡bi †jvKR‡bi Avgv‡`i †`Iqv mvevb e¨envi Kiv DwPZ bq|Avcbvi cwiev‡i Kv‡iv cwi`k©‡bi welqwU Avcbv‡`i Rb¨ wKQyUv A¯^w¯Í`vqK n‡Z cv‡i| Z‡e Avcbvi cwiev‡i cwi`k©‡bi d‡j Avcwb ev Avcbvi cwievi †Kvb ai‡bi ¶wZi m¤§yLxb n‡eb bv e‡j Avkv Kwi|**

**GB M‡elYvq AšÍ©f~³ cÖwZwU evwo bZzb AvPib wkLvi I mvevb cvIqvi myweav jvf Ki‡e| GQvov Zvr¶wbK Avi †Kvb myweav cv‡eb bv| GKUv Lvbvi g‡a¨ Bbd¬z‡qÄv fvBivm Qov‡bvi Rb¨ wK wK welq RwoZ GB M‡elYv Avgv‡`i‡K †m m¤ú‡K© Av‡iv fvjfv‡e eyS‡Z mvnvh¨ Ki‡e| GB M‡elYv Avgv‡`i‡K Bbd¬z‡qÄv †iv‡Mi j¶Y /DcmM© we¯Ívi †iva Kivi †KŠkj cix¶v Ki‡Z mvnvh¨ Ki‡e|** evsjv‡`‡ki MÖvgvÂ‡ji cwiev‡ii g‡a¨ Bbd¬z‡qÄv **†iv‡Mi j¶Y/DcmM© we¯Ívi wKfv‡e †iva Kiv hvq GB M‡elYv Zv eyS‡ZI mnvqZv Ki‡Z cv‡i|**

**‡hme †jv‡Ki g‡a¨** Bbd¬z‡qÄv fvBiv‡mi DcmM© †`Lv hv‡e Ges hviv Zv‡`i Mjv Ges bvK †_‡K bgybv msMÖn Ki‡Z w`‡Z m¤§Z n‡e Avgv‡`i GKRb cÖwkw¶Z M‡elbvKgx© Zv‡`i Mjv Ges bv‡K GKwU Zzjv †`qv KvwV w`‡q bgybv msMÖn Ki‡e| GUv Zv‡`i Rb¨ wKQyUv A¯^w¯—`vqK n‡Z cv‡i|Rvbv g‡Z GQvov G‡Z Avi †Kvb ¶wZi m¤¢vebv †bB|

**bgybv msMÖ‡ni ci GK gvm ev K‡qK gv‡mi Rb¨ Bbd¬z‡qÄv cix¶vi djvdj Rvbv hv‡e bv| Bbd¬z‡qÄv cix¶vi GB djvdj †Kvbfv‡eB AvµvšÍ e¨w³i wPwKrmvq †Kvb cÖfve ‡dj‡e bv|**

**‡MvcbxqZv:**

Avcbvi mgv‡Ri mKj m`m¨m¤ú‡K© msM„nxZ mKj Z_¨ †Mvcb ivLv n‡e| mKj Z_¨ GKwU Zvjve× Avjgvix‡Z dvBjeÜx K‡i ivLv n‡e| M‡elYvi mv‡_ mswk­ó bq Ggb KvD‡K Avgiv Avcbvi mgv‡Ri †Kvb Z_¨ mieivn Kie bv|

fwerl¨‡Z Z_¨¸‡jvi e¨envi: fwerl¨‡Z hw` Z_¨¸‡jv Ab¨ M‡el‡Ki cÖ‡qvR‡b e¨envi Kiv nq,Z‡e Avgiv Avcbvi †Kvb e¨w³MZ Z_¨ Zv‡`I mieivn Ki‡ev bv| Avgiv K‡Vvi †MvcbxqZv i¶v Ki†ev|

**M‡elYvq AskMÖnb bv Kiv Ges cÖZ¨vnv‡ii AwaKvi:**

Avcwb Avcbvi evwowU‡K GB M‡elYvq AšÍ©f~³ Ki‡Z m¤§Z n‡Z cv‡ib Avevi bvI cv‡ib| M‡elYv PjvKvjxb †h‡Kvb mg‡q Avcwb AskMÖnY Ki‡Z Am¤§ZI n‡Z cv‡ib| †h‡Kvb mgq Avcwb Avcbvi evwo‡K M‡elYv †_‡K cÖZ¨vnvi K‡i wb‡Z cv‡ib| M‡elYvq AskMÖnY Ki‡Z Am¤§wZ A_ev M‡elYv †_‡K bvg cÖZ¨vnv‡ii d‡j Avcbvi KwgDwbwUi †Kvb m`m¨‡K †Kvb kvw¯Í cÖ`vb Kiv n‡e bv Ges wK¬wbK ev nvmcvZv‡ji cÖvc¨ wPwKrmv †mev †_‡KI ewÂZ n‡eb bv| GgbwK Avcwb hw` Avcbvi evwo‡K GB M‡elYvq AšÍ©f~³ bvI K‡ib, ZeyI Avcbvi evwoi cÖwZwU m`m¨ wK¬wbK ev nvmcvZvj †_‡K h_vh_ ¯^v¯’¨‡mev cv‡e| Avcbvi evwoi cÖwZwU m`m¨ e¨w³MZfv‡eI GB M‡elYvq AskMÖnb Ki‡Z cv‡ib Avevi bvI cv‡ib| GgbwK M‡elYv PjvKvjxb †h‡Kvb mg‡q M‡elYv †_‡K wb‡R‡K cÖZ¨vnvi K‡i wb‡Z cv‡ib|

¶wZc~iY bxwZ t

GB M‡elYvq AskMÖn‡bi Rb¨ Avcbv‡K A_ev Avcbvi evwo‡K †Kvb A_© cÖ`vb Ki‡Z n‡e bv| GKBfv‡e webv g~‡j¨ mvevb cvIqv Qvov GB M‡elYvq AskMÖn†bi Rb¨ Avi wKQy cv‡eb bv|

‡hvMv‡hvMi e¨vw³t

GB cÖwµqv PjvKvjxb Avcbvi †Kvb cÖkœ _vK‡j †h †Kvb mgq Ki‡Z cv‡ib| hw` Avcbvi M‡elbv m¤ú‡K© †Kvb AwZwi³ cÖkœ _v‡K Avcwb †hvMv‡hvM Ki‡Z cv‡ib t

Wvt w÷‡db wc. jzwŸ, †cÖvMÖvg Ab Bb‡dKkvm wWwRR G¨vÛ †fKwmb mvB‡Ým (wc,AvvB.wW.wf.Gm) AvB.wm.wW.wW.Avi.we, gnvLvjx, XvKv 1212, †dvb t 8860523-32 # 2502|

GB M‡elbv Ask wnmv‡e Avcbvi AwaKvi m¤ú‡K© hw` Avcbvi †Kvb cÖkœ _v‡K A_ev hw` M‡elbvi Rb¨ Avcbvi †Kvb ¶wZ n‡q‡Q e‡j g‡b nq Zvn‡j †hvMv‡hvM Ki“b t

wgt Gg.G.mvjvg, wimvP© G¨Û cÖ‡R± mv‡cv©U wWcvU©‡g›U (Avi.wc.Gm.wW) AvB.wm.wW.wW.Avi.we, gnvLvjx, XvKv– 1212, †dvb t 9886489, # 01711428989|

Avcwb hw` GB M‡elYvq Avcbvi evwo‡K AšÍ©f~³ Ki‡Z m¤§Z _v‡Kb**, Zvn‡j AbyMÖn K‡i bx‡P Avcbvi ¯^v¶i A_ev evg nv‡Zi e„×v½ywji Qvc w`b:**

# Avcbvi mn‡hvwMZvi Rb¨ ab¨ev`|

__________________________________ ____________________

AskMÖnYKvixi ¯^v¶i/evg nv‡Zi e„×v½yjxi Qvc ZvwiL

__________________________________ ____________________

mv¶xi ¯^v¶i/evg nv‡Zi e„×v½yjxi Qvc ZvwiL

__________________________________ ___________________

M‡elK/cÖwZwbwai ¯^v¶i ZvwiL

**Appendix 6: evwo Dchy³Zv hvPvB dg© Bari Eligibility Form** VERSION 16.06.10

INDEX CASE ID: ______________________ FRA CODE: _____________________

| 1. wRÁvmv Ki“b: **আইসিপি ছাড়া আইসিপি** Lvbvi †Kv‡bv m`m¨wK MZ 7 w`‡bi g‡a¨ R¡‡i f~‡M‡Q?  1. **ASK:** Apart from the ICP, have any of the residents of the ICP household had a fever in the past 7  days?  Yes (1) No (2)  (ÓnvÓ n‡j evwo Dchy³ bq) (If ‘yes,’ this bari is ineligible) | |  |
| --- | --- | --- |
| **1b. wRÁvmv Ki“b: আইসিপি Lvbv ছাড়া Avcbvi evwoi †Kv‡bv m`m¨wK MZ 7 w`‡bi g‡a¨ R¡‡i f~‡M‡Q?**  1b. **ASK:** Apart from the ICP household, have any of the residents of the bari had a fever in the past 7 days?  Yes (1) No (2)  (ÓnvÓ n‡j 1c Ges 1d ‡Z hvb, bv n‡j 2 G hvb) (If ‘yes,’ go to 1c and 1d. If ‘no,’ go to 2.) | |  |
| **1c. hw` 1b nu¨v nq, wRÁvmv Ki“b: Zvi KZ w`b R¡i wQj ? রেকর্ড করুন: bvg I KZ w`b R¡i wQj (99 = Rvwb bv )**  1c. If 1b is “yes,” **ASK:** How many days did s/he have fever? **RECORD:** Name and days of fever (99=don’t know)  bvg/ Name___________________________ w`b / Days _____  bvg/ Name________________ ___________w`b / Days _____  bvg/ Name___________________________ w`b / Days _____  (1d চালিয়ে যান) (Continue to 1d.) | |  |
| 1d. evoxi Kv‡iv wK MZ 7 w`‡b 2 w`b ev Zvi †ekx w`b a‡i R¡i wQj?  1d. Did anyone in the bari have fever for 2 or more days during the past 7 days?  Yes (1) No (2)  (ÓnvÓ n‡j evwo Dchy³ bq) (If ‘yes,’ this bari is ineligible) | |  |
| 2. wRÁvmv Ki“b: Bb‡W· †Km wK GB evwo‡Z AvMvgx 20 w`b Nygv‡e?  2. **ASK:** Will the index case be sleeping in this bari for the next 20 days?  Yes (1) No (2)  (ÓbvÔ n‡j evwo Dchy³ bq) (If ‘no,’ this bari is ineligible) | |  |
| 3. wRÁvmv Ki“b: Bb‡W· †Km QvovI Av‡iv 2 ev Zvi †ewk †jvKRb wK GB evwo‡Z AvMvgx 20 w`b _vK‡e?  3. **ASK:** Will 2 or more other persons, other than the index case, be living in your *bari* during next 20 days?  Yes (1) No (2)  ÓbvÔ n‡j evwo Dchy³ bq| (If ‘no,’ this bari is ineligible) | |  |
| 4. wRÁvmv Ki“b: Avcbvi evwowU wK Avgv‡`i M‡elYvq BwZc~‡e© ‡bqv n‡qwQ‡jv?  4. **ASK:** Was your Bari enrolled before in our Study?  Yes (1) No (2)  ÓnvÓ n‡j evwo Dchy³ bq| (If ‘yes’, this bari is ineligible) | |  |
| **5. রেকর্ড করুন: evwo wK Dc‡ii AšÍ©f~w³ KiY wbq‡gi g‡a¨ c‡i‡Q?**  5. **RECORD**: Does the bari meet the above inclusion criteria?  Yes (1) No (2)  (ÓnvÓ n‡j বাড়ি সম্মতি চালিয়ে যান।‘না’ হলে বাড়ি উপযুক্ত নয়, ধন্যবাদ দিন। ‌  (If ‘yes’, continue with Bari Consent. If ‘no’, bari is ineligible 🡪 please thank them for their time) |  | |
|  |  | |
| **6. রেকর্ড করুন: evwoi cÖavb ev g‡bvbxZ cÖwZwbwa wK Lvbv AbygwZ c‡Î ¯^v¶i K‡i‡QY?**  6. **RECORD:** Did the head of the bari, or appropriate designee, and any necessary household leaders accept and sign the household consent form?  Yes (1) No (2)  (ÓnvÓ n‡j বাড়ি সম্মতি চালিয়ে যান।‘না’ হলে বাড়ি উপযুক্ত নয়, ধন্যবাদ দিন।  (If ‘yes’, continue with Bari Drawing. If ‘no’, this bari is ineligible🡪 please thank them for their time) |  | |
|  |  | |
| 7**. রেকর্ড করুন: ইনিউমেরেশন ফর্ম পূরণের কোনো পর্যায়ে আইসিপি Lvbvi †Kvb m`m¨i (AvBwmwc e¨vwZZ) MZ 7 w`‡bi g‡a¨ R¡i wQj e‡j Rvbv †M‡Q wK A_ev †Kvb evox m`m¨i (AvBwmwc _vbvi evB‡i) MZ 7w`‡bi g‡a¨ 2 ev Zvi †ekx w`b R¡i wQj e‡j Rvbv †M‡Q wK ?**  7. **RECORD:** At any point while completing the Enumeration Form, was a resident of the ICP household (other than the ICP) found to have fever during the last 7 days, or was a bari resident (outside the ICP household) found to have had a fever for two or more days during the last 7 days?  Yes (1) No (2)  (‘না’ হলে গৃহস্থালী সংক্রান্ত প্রশ্নমালা চালিয়ে যান। ‘হা’ হলে বাড়ি উপযুক্ত নয়, ধন্যবাদ দিন)  (If ‘no’, continue with Household Questionnaires .If ‘yes’, this bari is ineligible 🡪 please thank them for their time) |  | |

**cÖ_g wfwR‡U Rvb‡Z PvB‡Z n‡e:** **To be asked at the first visit to the household:**

**Amy¯’Zv UªvwKs†kl nIqvi ci DËi †`qvi R‡b¨ (A_ev Zvi c~‡e© Wªc AvDU Zvwi‡Li Dci wbf©ikxj)**

To be answered after completion of illness tracking (or before depending on drop out date):

| 8. Amy¯’Zv U¨vwKs Gi †Kv‡bv ch©v‡q wK evwo AskMÖn‡Y A¯^xK…wZ Rvwb‡q‡Q (যে কোনো কারণে)?  8. **RECORD:** Did bari discontinue study participation (for any reason) during illness tracking? Yes (1) No (2)  ÓnvÓ n‡j wb‡P KviY e¨vL¨v Kiæb| (If ‘yes’, explain why below) |  |
| --- | --- |
| 9. Wªc AvD‡Ui KviY 9. Explanation of household drop out:  10. Wªc AvD‡Ui ZvwiL _____/______/_______Date of drop out (dd/mm/yyyy):_____/______/_______ | |

**Appendix 7: Bari Drawing Form** VERSION 18.5.10

Address, Directions and Phone Number of Bari:

Use the symbols below to draw a picture to represent the bari.

**Symbol Definitions:**

= Housing Structure = Cooking Area (Stove) = Toilet Facilities = Entrance to Bari

(draw the most used entrance)

#

= Handwashing station -- -- -- -- = Draw a dashed line between the front entrance of housing structure “01”

and the nearest front entrance of each other numbered structure.. Label each line

with the length in steps.

+

*

SSS

= Entrance(s) to Housing Structure = Water source = Intervention handwashing station (draw next day)

|  |  |  |  |  |  |  |  |  |  |
| --- | --- | --- | --- | --- | --- | --- | --- | --- | --- |

|  |  |  |
| --- | --- | --- |

ICP ID: FRA Code:

**Appendix 8: Household Contact Enumeration Form** (VERSION 07.07.2010) ICP Household (1) Secondary Household (2)

Date (dd/mm/yy) :___ ___/ ___ ___/ ___ ___ Code of FRA:  Household Unique ID# :  Call to FRO: Yes=1, No=2 

Name of Respondent: _________________________ Did household participate in bari consent (1) or consent as an individual household (2)? 

ASK Primary Respondent: “Who are the members of your khana?” List whoever the respondent lists. Include guests if the respondent lists them; otherwise DO NOT.

| Individual Number | (a) Name  (Write in ENGLISH and CAPITAL letters) | (b)  Sex  Male  (1)  Female  (2) | (c)  Age-years (YY) | (d)  Age-months (mm) | Unique ID (10 digits) =  -ICP’s serial number (4 digits)  -Housing structure number  (2 digits)  - Household number (2 digits)  - Individual (enumeration) number  (e) Unique ID | | | | | | | | | | (f) Interaction with ICP  Avcwb Bb‡W· †K‡mi m‡½ w`‡bi g‡a¨ K‡ZvUv mgq KvUvb?  How often does _______ spend time with the index-case patient?*  READ CHOICES  Multiple times per day 1  Around once per day 2  A few times each week 3  Around once per week 4  Rarely 5  Never 6  Self 7  No answer 99 | (g)  Relationship of _____ to Index Case  Self (1)  Parent (2) Child (3)  Sibling (4)  Grandparent (5)  Aunt/Uncle (6)  Cousin (7)  Spouse (8)  Unrelated (9)  Grandchild (10)  Other (specify)  (88)  NOTE: Include in-laws within these categories. For example, code “sister-in-law” as “sibling” | (h)  What is the smoking status of _______?  (Ask of respondents ≥ 15 years of age)  Never (0)  Former (1)  Current(2)  Refused to answer (77)  Not applicable (due to age)  (88)  Don't Know (99) | (j)  On most days, how many minutes per day does this person spend in the cooking area of your household?  NOTE:  Ask this regardless of whether time spent involves cooking | (k)  On most nights, does ______  sleep in the same room as the index case?  Yes(1)  No (2)  Self (3) | (l) Has ____ had a fever in the last 7 days (including today)?  Yes (1)  No (2)  Not Presently living in Bari (3)  Don’t Know (99)  / Date of symptom onset  (dd/mm/yy)  For ICP, put “1” and write onset date | (m) Has _____ had a cough in the last 7 days (including today)?  Yes (1)  No (2)  Not Presently living in Bari (3)  Don’t Know (99)  / Date of symptom onset  dd/mm/yy | (n) Has ____ had a sore throat in the last 7 days (including today)?  Yes (1)  No (2)  Not Presently living in Bari (3)  Don’t Know (99)  / Date of symptom onset  (dd/mm/yy) |
| --- | --- | --- | --- | --- | --- | --- | --- | --- | --- | --- | --- | --- | --- | --- | --- | --- | --- | --- | --- | --- | --- | --- |
|  |  |  | Check vaccination (EPI) card, or birth certificate when household member is <5 years old or if respondent is not certain of household member’s age | |  |  |  |  |  |  |  |  |  |  |  |  |  |  |  |  |  |  |
|  |  |  | For children less than 1 month/30 days old, write “00” for both columns | |  |  |  |  |  |  |  |  |  |  |  |  |  |  |  |  |  |  |
|  |  |  |  |  |  |  |  |  |  |  |  |  |  |  |  |  |  |  |  | *🡪 If “yes,” please record more information on p.3, Sick List* | | |
| 01 |  |  |  |  |  |  |  |  |  |  |  |  |  |  |  |  |  |  |  |  |  |  |
| 02 |  |  |  |  |  |  |  |  |  |  |  |  |  |  |  |  |  |  |  |  |  |  |
| 03 |  |  |  |  |  |  |  |  |  |  |  |  |  |  |  |  |  |  |  |  |  |  |
| 04 |  |  |  |  |  |  |  |  |  |  |  |  |  |  |  |  |  |  |  |  |  |  |
| 05 |  |  |  |  |  |  |  |  |  |  |  |  |  |  |  |  |  |  |  |  |  |  |
| 06 |  |  |  |  |  |  |  |  |  |  |  |  |  |  |  |  |  |  |  |  |  |  |
| 07 |  |  |  |  |  |  |  |  |  |  |  |  |  |  |  |  |  |  |  |  |  |  |
| 08 |  |  |  |  |  |  |  |  |  |  |  |  |  |  |  |  |  |  |  |  |  |  |
| 09 |  |  |  |  |  |  |  |  |  |  |  |  |  |  |  |  |  |  |  |  |  |  |
| * mgq KvUv‡bv ej‡Z K_v ejv, Lvevi LvIqv, Mí Kiv BZ¨vw` †evSv‡bv n‡”Q: A_ev wkï ICP Gi †¶‡Î: †Ljv Kiv, LvIqv‡bv , †Mvmj Kiv‡bv BZ¨vw`  By “spending time”, we mean: talking with the person, sharing meals, gossiping, etc.; or in the case of a child ICP: playing with, feedbing , bathing, etc. | | | | | | | | | | | | | | | | | | | | | | |

**Appendix 8: Household Contact Enumeration Form (PAGE 2, if needed)**

| Individual Number | | (a) Name  (Write in ENGLISH and CAPITAL letters) | | (b)  Sex  Male  (1)  Female  (2) | (c)  Age-years (YY) | (d)  Age-months (mm) | | Unique ID (10 digits) =  -ICP’s serial number (4 digits)  -Housing structure number  (2 digits)  - Household number (2 digits)  - Individual (enumeration) number  (e) Unique ID | | | | | | | | | | | | | | | | | | | | (f) Interaction with ICP  Avcwb Bb‡W· †K‡mi m‡½ w`‡bi g‡a¨ K‡ZvUv mgq KvUvb?  How often does _______ spend time with the index-case patient?*  READ CHOICES  Multiple times per day 1  Around once per day 2  A few times each week 3  Around once per week 4  Rarely 5  Never 6  Self 7  No answer 99 | | (g)  Relationship of _____ to Index Case  Self (1)  Parent (2) Child (3)  Sibling (4)  Grandparent (5)  Aunt/Uncle (6)  Cousin (7)  Spouse (8)  Unrelated (9)  Grandchild (10)  Other (specify)  (88)  NOTE: Include in-laws within these categories. For example, code “sister-in-law” as “sibling” | | (h)  What is the smoking status of _______?  (Ask of respondents ≥ 15 years of age)  Never (0)  Former (1)  Current(2)  Refused to answer (77)  Not applicable (due to age)  (88)  Don't Know (99) | (j)  On most days, how many minutes per day does this person spend in the cooking area of your household?  NOTE:  Ask this regardless of whether time spent involves cooking | | | (k)  On most nights, does ______  sleep in the same room as the index case?  Yes(1)  No (2)  Self (3) | (l) Has ____ had a fever in the last 7 days (including today)?  Yes (1)  No (2)  Not Presently living in Bari (3)  Don’t Know (99)  / Date of symptom onset  (dd/mm/yy)  For ICP, put “1” and write onset date | | (m) Has _____ had a cough in the last 7 days (including today)?  Yes (1)  No (2)  Not Presently living in Bari (3)  Don’t Know (99)  / Date of symptom onset  dd/mm/yy | (n) Has ____ had a sore throat in the last 7 days (including today)?  Yes (1)  No (2)  Not Presently living in Bari (3)  Don’t Know (99)  / Date of symptom onset  (dd/mm/yy) | | |
| --- | --- | --- | --- | --- | --- | --- | --- | --- | --- | --- | --- | --- | --- | --- | --- | --- | --- | --- | --- | --- | --- | --- | --- | --- | --- | --- | --- | --- | --- | --- | --- | --- | --- | --- | --- | --- | --- | --- | --- | --- | --- | --- |
|  |  |  |  |  | Check vaccination (EPI) card, or birth certificate when household member is <5 years old or if respondent is not certain of household member’s age | | |  |  |  |  |  |  |  |  |  |  |  |  |  |  |  |  |  |  |  |  |  |  |  |  |  |  |  |  |  |  |  |  |  |  |  |
|  |  |  |  |  | For children less than 1 month/30 days old, write “00” for both columns | | |  |  |  |  |  |  |  |  |  |  |  |  |  |  |  |  |  |  |  |  |  |  |  |  |  |  |  |  |  |  |  |  |  |  |  |
|  |  |  |  |  |  |  |  |  |  |  |  |  |  |  |  |  |  |  |  |  |  |  |  |  |  |  |  |  |  |  |  |  |  |  |  |  | *🡪 If “yes,” please record more information on p.3, Sick List* | | | | | |
| 10 | |  | |  |  |  | |  | |  | |  | |  | |  | |  | |  | |  | |  | |  | |  | |  | |  |  | | |  |  | |  |  | | |
| 11 | |  | |  |  |  | |  | |  | |  | |  | |  | |  | |  | |  | |  | |  | |  | |  | |  |  | | |  |  | |  |  | | |
| 12 | |  | |  |  |  | |  | |  | |  | |  | |  | |  | |  | |  | |  | |  | |  | |  | |  |  | | |  |  | |  |  | | |
| 13 | |  | |  |  |  | |  | |  | |  | |  | |  | |  | |  | |  | |  | |  | |  | |  | |  |  | | |  |  | |  |  | | |
| 14 | |  | |  |  |  | |  | |  | |  | |  | |  | |  | |  | |  | |  | |  | |  | |  | |  |  | | |  |  | |  |  | | |
| 15 | |  | |  |  |  | |  | |  | |  | |  | |  | |  | |  | |  | |  | |  | |  | |  | |  |  | | |  |  | |  |  | | |
| 16 | |  | |  |  |  | |  | |  | |  | |  | |  | |  | |  | |  | |  | |  | |  | |  | |  |  | | |  |  | |  |  | | |
| 17 | |  | |  |  |  | |  | |  | |  | |  | |  | |  | |  | |  | |  | |  | |  | |  | |  |  | | |  |  | |  |  | | |
| 18 | |  | |  |  |  | |  | |  | |  | |  | |  | |  | |  | |  | |  | |  | |  | |  | |  |  | | |  |  | |  |  | | |
| 19 | |  | |  |  |  | |  | |  | |  | |  | |  | |  | |  | |  | |  | |  | |  | |  | |  |  | | |  |  | |  |  | | |
| * mgq KvUv‡bv ej‡Z K_v ejv, Lvevi LvIqv, Mí Kiv BZ¨vw` †evSv‡bv n‡”Q: A_ev wkï ICP Gi †¶‡Î: †Ljv Kiv, LvIqv‡bv , †Mvmj Kiv‡bv BZ¨vw`  By “spending time”, we mean: talking with the person, sharing meals, gossiping, etc.; or in the case of a child ICP: playing with, feedbing , bathing, etc. | | | | | | | | | | | | | | | | | | | | | | | | | | | | | | | | | | | | | | | | | | |
|  | **Appendix 8b: Enrollment Day Sick List for all Bari members** | | | | | | | | | | | | | | | | | | | | | | | | | | | | | | | | | | | | | | | |  | |
|  | *If any member of the ICP household had fever during the last 7 days, the bari is ineligible for study participation. Do not continue with Sick List* 🡪 Return to Bari Eligibility Form  List any bari member (including the ICP) that had fever, cough or sore throat during the last 7 days (including today), and record the number of days s/he had each symptom. | | | | | | | | | | | | | | | | | | | | | | | | | | | | | | | | | | | | | | | |  | |
|  |  | | Name | | | | Unique ID | | | | | | | | | | | | | | | | | | | | Number of days of fever during last 7 days  Last day of symptoms (dd/mm/yy) or today’s date  99= don’t know  *If any bari member (outside the ICP household) had fever for 2 or more days during the last 7 days, the bari is ineligible for study participation* 🡪 Return to Bari Eligibility Form | | | | Date of Sample Collection (if fever is present on day of enrollment)  (dd/mm/yy) | | | Number of days of cough during last 7 days  Last day of symptoms  (dd/mm/yy)  or today’s date  99= don’t know | | | | Number of days of sore throat during last 7 days  Last day of symptoms  (dd/mm/yy) or today’s date  99= don’t know | | |  | |
|  | 1 | |  | | | |  | |  | |  | |  | |  | |  | |  | |  | |  | |  | |  | |  | |  | | |  |  | | |  |  | | |  |
|  | 2 | |  | | | |  | |  | |  | |  | |  | |  | |  | |  | |  | |  | |  | |  | |  | | |  |  | | |  |  | | |  |
|  | 3 | |  | | | |  | |  | |  | |  | |  | |  | |  | |  | |  | |  | |  | |  | |  | | |  |  | | |  |  | | |  |
|  | 4 | |  | | | |  | |  | |  | |  | |  | |  | |  | |  | |  | |  | |  | |  | |  | | |  |  | | |  |  | | |  |
|  | 5 | |  | | | |  | |  | |  | |  | |  | |  | |  | |  | |  | |  | |  | |  | |  | | |  |  | | |  |  | | |  |
|  | 6 | |  | | | |  | |  | |  | |  | |  | |  | |  | |  | |  | |  | |  | |  | |  | | |  |  | | |  |  | | |  |
|  | 7 | |  | | | |  | |  | |  | |  | |  | |  | |  | |  | |  | |  | |  | |  | |  | | |  |  | | |  |  | | |  |
|  | 8 | |  | | | |  | |  | |  | |  | |  | |  | |  | |  | |  | |  | |  | |  | |  | | |  |  | | |  |  | | |  |
|  | 9 | |  | | | |  | |  | |  | |  | |  | |  | |  | |  | |  | |  | |  | |  | |  | | |  |  | | |  |  | | |  |
|  | 10 | |  | | | |  | |  | |  | |  | |  | |  | |  | |  | |  | |  | |  | |  | |  | | |  |  | | |  |  | | |  |
|  | 11 | |  | | | |  | |  | |  | |  | |  | |  | |  | |  | |  | |  | |  | |  | |  | | |  |  | | |  |  | | |  |
|  | 12 | |  | | | |  | |  | |  | |  | |  | |  | |  | |  | |  | |  | |  | |  | |  | | |  |  | | |  |  | | |  |
|  | 13 | |  | | | |  | |  | |  | |  | |  | |  | |  | |  | |  | |  | |  | |  | |  | | |  |  | | |  |  | | |  |
|  | 14 | |  | | | |  | |  | |  | |  | |  | |  | |  | |  | |  | |  | |  | |  | |  | | |  |  | | |  |  | | |  |
|  | 15 | |  | | | |  | |  | |  | |  | |  | |  | |  | |  | |  | |  | |  | |  | |  | | |  |  | | |  |  | | |  |

M„n¯’vjx msµvšÍ cÖkœgvjv/ ch©‡e¶Y dg©

**Appendix 9: Household Level Questionnaire/Observation Form**

**Start Time (24-hour format): __ __:__ __**

**Section One: Respondent Information**

1.1 AvBwmwc AvBwW ICP ID  1.2 Gd Avi G †KvW FRA Code 

1.3 DËi`vZv i bvg Name of Respondent: _______________________________________

1.4 DËi`vZv i BDwbK AvBwW (BwbD‡g‡ikb dg© †_‡K †bIqv) 

Respondent Unique ID# (Taken from enumeration form)

1.5 DËi`vZv i nvDwRs ÷ªvKPvi AvBwW # (BDwbK AvBwW-i 5g Ges 6ô msL¨v) 

Respondent Housing Structure ID # (5^th^ and 6^th^ digit of Unique ID)

1.6 DËi`vZv i Lvbv AvBwW# (BDwbK AvBwW-i 7g Ges 8g msL¨v) 

Respondent Household ID # (7^th^ and 8^th^ digit of Unique ID)

1.6b DËi`vZv wK AvBwmwc Lvbvi †jvK? 

Is the respondent from the ICP household?

Yes (n¨uv) 01

No (bv) 02

🡪 DËi 2 n‡j, †mKkb `yB-G P‡j hvb If (2), SKIP to SECTION TWO

1.7 BwbD‡g‡ikY dg© †`‡L †ei Ki“b AvBwmwc-i eqm 18 eQ‡ii †ewk wK-bv 

Look on Enumeration Form to see if ICP is 18 or above

Yes (n¨uv) 01

No (bv) 02

🡪 DËi 1 n‡j, †mKkb `yB-G P‡j hvb If (1), SKIP to SECTION TWO

| 1.8 AvBwmwcÕi eqm 18 eQ‡ii bx‡P n‡j wR‡Ám Ki“Y, ÒAvBwmwc Amy¯’ n‡j cÖavbZ ‡K Zv‡K †mev K‡i?Ó (†mev`vbKvixi GKK AvB wW wjLyb) cÖ‡hvR¨ bq =7700000000, ‡Kvb DËi bvB =9900000000  *If ICP < 18 years old,* ASK, “Who is the primary caregiver of the index case patient when s/he is sick?”  *(WRITE UNIQUE ID of caregiver)*  **Not Applicable = 7700000000; No Answer= 9900000000**    1.9 ÒAvBwmwc Amy¯’ n‡j Avi †KD Zvi †mev K‡i?Ó (DËi Ònu¨vÓ n‡j GKK AvBwW wjLyb)  cÖ‡hvR¨ bq bv=**2000000000** =**7700000000,** ‡Kvb DËi bvB **/e¨w³wUBwbDgv‡i‡UW nqwb =9900000000**  ASK, “Does anyone else take care of the index case patient when s/he is sick?”*(WRITE UNIQUE ID IF “YES”)*  **No = 2000000000; Not Applicable = 7700000000; No Answer/person not enumerated write 9900000000**   |
| --- |

**End of Section One**

**‡mKkb 2: Ávb Section Two: Knowledge**

|  | (‡mvqvBb d¬z) **Swine Flu** | | (Bbd¬z‡qÄv) **Influenza Illness** | |
| --- | --- | --- | --- | --- |
| **2.1** DËi`vZv‡K wRÁvmv Ki“b  ÒAvcwb wK KL‡bv _______________ kãwU ï‡b‡Qb?Ó  (ev‡´ †KvW wjLyb )  **ASK:** Have you ever heard of the word_______?  (Record code in box)  ***-*** ***j¶¨ Ki“b: †Kv‡bvfv‡eB †mvBb d¬z ev Bbd¬z‡qÄvwel‡q e¨vL¨v Ki‡eb bv | ïay cÖkœKi“b, Zviv GB kãwUï‡b‡Q wK bv?***  ***--NOTE:*** *Do not explain the terms& “swine flu” or “influenza illness” in any way. Simply ask if they know the word/term.*  Yes (n¨uv) 01  No (bv) 02  Don’t Know/Not Sure (Rvwb bv) 99 |   **🡪** DËi **02** ev **99** n‡j ciewZ© Kjv‡g P‡j hvb  **If the answer is 02 or 99, SKIP to next column**  🡪 DËi **01** n‡j GB Kjvg Pvwj‡q hvq|  **If the answer is 01, continue down This column** | |   **🡪** DËi **02** ev **99** n‡j cªkœ **2.28**-G P‡j hvb  **If the answer is 02 or 99, SKIP to question 2.28**  🡪 DËi **01** n‡j GB Kjvg Pvwj‡q hvq|  **If the answer is 01, continue down This column** | |
| **2.2** Avcwb wK Rv‡bb,-------------G †Kvb j¶Y †`Lv †`q?  **ASK,** Do you know what symptoms can occur with______?  Yes (n¨uv) 01  No (bv) 02  Don’t Know/Not Sure (Rvwb bv) 99 |   **🡪** DËi **02** ev **99** n‡j cªkœ **2.4**-G P‡j hvb  **If the answer is 02 or 99, SKIP to question 2.4** | |   **🡪** DËi **02** ev **99** n‡j cªkœ **2.4**-G P‡j hvb  **If the answer is 02 or 99,**  **SKIP to question 2.4** | |
| **2.3** DËi`vZv‡K wRÁvmv Ki“Y ------- G †Kvb †Kvb j¶Y †`Lv ‡`q ?  **ASK:** What symptoms are associated with _____?  *(Ackb¸‡jv D”P¯^‡i c‡o †kvbv‡eb bv)*  *(G cÖkœwU †Lvjv cÖ‡kœi gZ K‡i wR‡Ám Ki“b, DËi`vZv cv‡ki ZvwjKvi DcmM©¸‡jv D‡jøL Ki‡j n¨uv =1 , bv =2, †KvW Kiyb)|*  *Avcwb cÖ‡qvRb‡ev‡a ÒAvi wKQyÓ K_vwU `yBevi wR‡Ám Ki‡Z cv‡ib|*  *(DO NOT READ RESPONSE OPTIONS ALOUD Ask this as an open-ended question. If the respondent mentions the listed symptom, write “1” for “yes” in the space, write “2” for “no”. May ask “anything else?” twice if appropriate.)*  Yes(n¨uv) 1  No(bv) 2 | Kvwk (Cough) ___  Mjve¨_v (Sore throat) ___  bvK Siv (Runny nose) ___  R¡i (Fever) ___  gv_v e¨_v (Headache) ___  kixi e¨_v (Body Aches) ___  Wvqwiqv(Diarrhea) ___  nuvwP (Sneezing) ___  ewg (Vomiting) ___  ‡Pv‡Li mgm¨v  (Eye Problems) ___  Ab¨vb¨, wb‡P wbw`©ó Ki“b  (Other, specify below) ___  _______________________ | | Kvwk (Cough) ___  Mjve¨_v (Sore throat) ___  bvK Siv (Runny nose) ___  R¡i (Fever) ___  gv_v e¨_v (Headache) ___  kixi e¨_v (Body Aches) ___  Wvqwiqv(Diarrhea) ___  nuvwP (Sneezing) ___  ewg (Vomiting) ___  ‡Pv‡Li mgm¨v  (Eye Problems) ___  Ab¨vb¨, wb‡P wbw`©ó Ki“b  (Other, specify below) ___  _______________________ | |
|  | | **Swine Flu** | | **Influenza Illness** |
| **2.4**  ______ gvby‡li wKfv‡e nq?  **ASK,** How a person can get sick with __________?  *((cQ›`¸‡jv D”P¯^‡i c‡o †kvbv‡eb bv) G cÖkœwU †Lvjv cÖ‡kœi gZ K‡i wR‡Ám Ki“b, DËi`vZv cv‡ki ZvwjKvi DcmM©¸‡jv D‡jøL Ki‡j n¨uv =1 , bv =2, †KvW Kiyb)| Avcwb cÖ‡qvRb‡ev‡a ÒAvi wKQyÓ K_vwU `yBevi wR‡Ám Ki‡Z cv‡ib|)*  *(DO NOT READ RESPONSE OPTIONS ALOUD Ask this as an open-ended question. If the respondent mentions the listed symptom, write “1” for “yes” in the space, write “2” for “no” in the space. May ask “anything else?” twice if appropriate.)*  Yes(n¨uv) 1  No(bv) 2 | |  | |  |
| a. _____†ivMxi ms¯ú‡k© _vK‡j Close contact with ________ patient | | a.  | | a.  |
| b. cÖvbxi ms¯ú‡k© Contact with animals | | b.  | | b.  |
| c. RxevbyMÖ¯Í e¯‘ †_‡K (wUDeI‡q‡ji nvZj, †dvb) Contact with contaminated surfaces (tube well handles, phones) | | c.  | | c.  |
| d.†bvsiv nv‡Z †L‡j ev LvIqv ˆZix Ki‡j Eating or preparing food with dirty hands | | d.  | | d.  |
| e. GKB _vjv-evmb, PvgP (ˆZRmcÎ) fvMvfvwM Ki‡j Sharing utensils | | e.  | | e.  |
| f. Rxevbyhy³ cvwb ev Lvevi †L‡j Contaminated drinking water or food | | f.  | | f.  |
| g. †hŠb m¤úK© Sexual relations | | g.  | | g.  |
| h. †bvsiv _vK‡j/A¯^v¯’¨Ki _vK‡j (¯^vfvweK fv‡e, nvZ cwi®‹vi Kivi K_v ejv nq bvB) Being dirty/poor hygiene (general, no mention of hand cleanliness | | h.  | | h.  |
| i. †bvsiv _vK‡j/A¯^v¯’¨Ki _vK‡j, (we‡klZ nvZ Acwi®‹vi _vK‡j ev evievi nvZ bv ay‡j) Being dirty/poor hygiene (hands specifically – having dirty hands or not washing hands enough) | | i.  | | i.  |
| j. মশা বা অন্য ‡cvKv-gvK‡oi Kvgo †_‡K Insect bite (mosquito or other insect) | | j.  | | j.  |
| k. fvBivm/e¨vK‡Uwiqv/Rxevby Virus/Bacteria/Germs | | k.  | | k.  |
| l. Ab¨vb¨/wbw`©ó Ki“b Other (specify) | | l.   If “other”, specify:  ________________________________ | | l.   If “other”, specify:  ________________________________ |
| m. Rvwb bv/ wbwðZ bB| Don’t know/not sure | | m.  | | m.  |
|  | (‡mvqvBb d¬z) **Swine Flu** | | (Bbd¬z‡qÄv) **Influenza Illness** | |
| **2.5** DËi`vZv‡K wRÁvmv Ki“b Ò­­­­­­­­­­______ wK cÖwZ‡iva/†VKv‡bv hvq?Ó  (ev‡´ †KvW wjLyb)  **ASK: “Can _____________ be prevented?”**  (Record code in box)  Yes (n¨uv) 01  No (bv) 02  Don’t Know/Not Sure (Rvwb bv) 99 |   **If the answer is 02 or 99, skip to top of next column**  DËi **02** ev  **99** n‡j ciewZ© Kjv‡g P‡j hvb | |   **If the answer is 02 or 99, skip to 2.9**  DËi **02** ev  **99** n‡j cÖkœ bs **2.9-**G P‡j hvb | |
| **2.6** Avcbv Kv‡Q wK g‡b nq ________-- প্রতিরোধ/†VKv‡ bv কি সহজ অথবা কঠিন?  **ASK,** Would you say that preventing the spread of __________ is easy or difficult?  সহজ Easy 01  কঠিন Difficult 02  জানি না/নিশ্চিত নই 99  Don’t know/not sure |   **If the answer is 99, skip to 2.8**  DËi  **99** n‡j cÖkœ bs **2.8-**G P‡j hvb | |   **If the answer is 99, skip to 2.8**  DËi **99** n‡j cÖkœ bs **2.8-**G P‡j hvb | |
| **2.7** যদি উত্তরদাতা “সহজ” উত্তর দেয়, তবে জিজ্ঞেস করুন “আপনি কি বলবেন যে এটি মোটামুটি সহজ না খুব সহজ?”    *🡪If respondent answered “easy”,* **ASK,** Would you say that it is somewhat easy or very easy?  যদি উত্তরদাতা “কঠিন” উত্তর দেয়, তবে জিজ্ঞেস করুন “আপনি কি বলবেন এটি মোটামুটি কঠিন না খুবই কঠিন?”  *🡪If respondent answered “difficult”,* **ASK,** Would you say that it is somewhat difficult or very difficult?  মোটামুটি সহজ Somewhat Easy 01  খুব সহজ Very Easy 02  মোটামুটি কঠিন Somewhat Difficult 03  খুবই কঠিন Very Difficult 04  উত্তর দেবেন না Refused to answer 66 |  | |  | |

|  | (‡mvqvBb d¬z) **Swine Flu** | (Bbd¬z‡qÄv) **Influenza Illness** |
| --- | --- | --- |
| **2.8** wRÁvmv Ki“b ______ wKfv‡e cÖwZ‡iva/†VKv‡bv hvq?  **ASK:** How can ___________ be prevented?  *(cQ›`¸‡jv D”P¯^‡i c‡o †kvbv‡eb bv)*  *G cÖkœwU †Lvjv cÖ‡kœi gZ K‡i wR‡Ám Ki“b, DËi`vZv cv‡ki ZvwjKvi DcmM©¸‡jv D‡jøL Ki‡j n¨uv =1 , bv =2, †KvW Kiyb)|*  ***(DO NOT READ RESPONSES ALOUD)***  *(Ask this as an open-ended question. If the respondent mentions the listed prevention, write “1” for “yes”and “2” for “no” in the space.)*  হ্যা Yes 1  না No 2 | Amy¯’ e¨w³ †_‡K `~‡i _vKvi  gva¨‡g  (Keep away from ill persons) ­­____  evievi nvZ †avqvi gva¨‡g  (Wash hands frequently) ____  evievi mvevb w`‡q nvZ †avqvi  gva¨‡g (Wash hand with soap frequently) ____  wUKv †bIqvi gva¨‡g  (Vaccination) ____  VvÛv Lvevi bv †L‡j  (Not taking cold foods) ____  Ab¨vb¨, wbw`©ó K‡i wjLyb  (Other, specify below) ____  ___________________________  Rvwb bv/ wbwðZ bB  (Don’t know/not sure) ____  ÕBbd¬z‡qÄvÕ Kjv‡g hvb  Go to ‘Influenza’ column | Amy¯’ e¨w³ †_‡K `~‡i _vKvi  gva¨‡g  (Keep away from ill persons) ­­____  evievi nvZ †avqvi gva¨‡g  (Wash hands frequently) ____  evievi mvevb w`‡q nvZ †avqvi  gva¨‡g (Wash hand with soap frequently) ____  wUKv †bIqvi gva¨‡g  (Vaccination) ____  VvÛv Lvevi bv †L‡j  (Not taking cold foods) ____  Ab¨vb¨, wbw`©ó K‡i wjLyb  (Other, specify below) ____  ___________________________  Rvwb bv/ wbwðZ bB  (Don’t know/not sure) ____ |

2.9. Avcwb gnvgvix kãwU ï‡b‡Qb? 

**ASK:** Have you heard the word “epidemic”?

Yes (n¨uv) 01

No (bv) 02

Don’t Know/Not Sure (Rvwb bv) 99

2.10. Avcwb c¨vb‡WwgK ev wek¦-gnvgvix kãwU ï‡b‡Qb? 

**ASK:** Have you heard the word “pandemic”?

Yes (n¨uv) 01

No (bv) 02

Don’t Know/Not Sure (Rvwb bv) 99

 DËi (2) ev (9) n‡j cÖkœ bs 2.12 G P‡j hvb (If (2) or (9), SKIP to question 2.12)

| 2.11. †Kvb eQi †kl Bbd¬z‡qÄv wek¦-gnvgvix †`Lv w`‡qwQ‡jv?   **ASK:** In which year did the last influenza pandemic occur?  *--লক্ষ্য করুন: উত্তরদাতা যদি বাংলা সাল বলে, তবে তা ইংরেজীতে বদলে নিন।বর্তমান বাংলা সাল ১৪১৭*  ***NOTE****: If answer is given in Bangla calendar years, convert to Roman calendar (Current Bangla year as of April 14th , 2010: 1417)*  যদি উত্তর জানি না/নিশ্চিত নই হয়, তবে “9999” লিখুন। **Write “9999” if “Don’t Know/Not Sure”** |
| --- |

2.12. †kl 12 gv‡m Avcbvi Lvbvi †KD wK ‡h †Kv‡bv Kvi‡Y nvmcvZv‡j fwZ© n‡qwQ‡jv? 

**ASK,** Has anyone in your household been hospitalized for any reason in the last 12 months?

Yes (n¨uv) 01

No (bv) 02

Don’t Know/Not Sure (Rvwb bv/ wbwðZ bB) 99

🡪 DËi (2) ev (99) n‡j cªkœ 2.15-G P‡j hvb (If (2) or (99), SKIP to question 2.15

| 2.13 Avcbvi Lvbvi KqRb MZ ১২ মাস কোনো কারণে nvmcvZv‡j fwZ© n‡qwQ‡jv?   **ASK**, How many people in your household have been hospitalized for any reason in the last 12 months?  2.14 গত ১২ মাসে hviv fwZ© n‡qwQ‡jv, Avcbvi g‡Z Zv‡`i KqRb শ্বাসকার্যের †Kv‡bv †iv‡Mi   যেমন জ্বর, কাশি, গলা ব্যাথা, নাক ঝরা, শ্বাস কষ্ট ইত্যাদি) Kvi‡Y fwZ© n‡qwQ‡jv?  জানি না/উত্তর নেই এর জন্যে “99” কোড করুন।  **ASK**, Of those people who were admitted to the hospital, in the last 12 months, how many people were hospitalized for a disease related to respiration (for example - fever, cough, sore throat, runny nose, breathing difficulties)?  Write “99” for don't know/no answer |
| --- |
|  |

| 2.15-2.21 Avwg আপনাকে এখন কিছু রোগের নাম বলবো এবং জানতে চাইবো কোন রোগটি আপনার বেশি ভয়ের কারণ?রোগগুলোর নাম আপনার জানা থাকতে পারে আবার নাও থাকতে পারে।আপনি প্রশ্নটি না বুঝতে পারলে আমাকে বলবেন আর কোনো রোগের নাম চেনা না মনে হলেও বলবেন।  **SAY,** I shall tell you name of some diseases and ask you which one causes more worry to you. You may or may not know the names of those diseases. Please let me know if you don’t understand my question and also let me know if any of the diseases are not known to you.  wR‡Ám Ki“b, Òwb‡Pi †Kvb †ivM Avcbvi me‡P‡q †ewk `ywðšÍvi/f‡qi KviY?Ó  **ASK**, Which illness is more concerning/causes more worry to you?  ***--লক্ষ্য করুন:*** *যদি উত্তরদাতা বলে “দু্টার মধ্যে কোন রোগ বেশি দুশ্চিন্তার কারণ, তা জানি না” তবে দয়া করে জোর দিয়ে বলুন এটি একটা মতামত এবং চিন্তা করে বলতে কোন্ রোগ বেশি দুশিন্তার কারণ?*  ***--লক্ষ্য করুন:*** *যদি কেউ একটি রোগ সম্পর্কে জানে, অন্যটি জানে না, তবে লিখুন “99”.*  ***--লক্ষ্য করুন:*** *যদি কেউ একটি রোগ সম্পর্কে না জানে, তবে সেই রোগটি যতোটি অপশনে আছে, তার প্রতিটিতে লিখুন“99”*  ***--NOTE:*** *If respondent says they “do not know which one is more concerning,” please stress that this is an opinion and ask them to think about which one is more concerning for them.*  ***--NOTE****: If someone is concerned about one, but does not know of the other, write “99”.*  ***--NOTE****: If someone does not know one of the diseases, you can automatically write “99” anytime that disease is listed.* | | | | | | | |
| --- | --- | --- | --- | --- | --- | --- | --- |
|  |  |  |  | `yBUvi g‡a¨ GKUv (বা দুটাই)†iv‡Mi bvg ‡kv‡bb bvB  (Don’t know one (or both) of the diseases) | উত্তর দেবেন না  Refused to Answer | উত্তরদাতা প্রশ্ন বোঝেন নাই এবং কোনো উত্তর নাই  Respondent does not understand question and has no response |  |
| 2.15 | Bbd¬z‡qÄv (Influenza) 01 | or | Wvqwiqv (Diarrhea) 02 | 99 | 66 | 33 |  |
| 2.16 | Wvqwiqv (Diarrhea) 01 | or | GBPAvBwf/GBWm (HIV/AIDS) 02 | 99 | 66 | 33 |  |
| 2.17 | Bbd¬z‡qÄv (Influenza) 01 | or | GBPAvBwf/GBWm (HIV/AIDS) 02 | 99 | 66 | 33 |  |
| 2.18 | GBPAvBwf/GBWm (HIV/AIDS) 01 | or | যক্ষা ev wUwe (Tuberculosis/TB) 02 | 99 | 66 | 33 |  |
| 2.19 | যক্ষা ev wUwe (Tuberculosis/TB) 01 | or | Wvqwiqv (Diarrhea) 02 | 99 | 66 | 33 |  |
| 2.20 | যক্ষা ev wUwe (Tuberculosis/TB) 01 | or | Bbd¬z‡qÄv (Influenza) 02 | 99 | 66 | 33 |  |

2.21 Avcwb wK g‡b K‡ib Bbd¬z‡qÄvq AvµvšÍ n‡q GKRb gvbyl gviv †h‡Z cv‡i? 

Do you think a person can die from influenza?

Yes (n¨uv) 01

No (bv) 02

Don’t Know/Not Sure (Rvwb bv/ wbwðZ bB) 99

🡪 DËi (02) ev (99) n‡j cªkœ 2.26-G P‡j hvb (If 02 or 99, SKIP to question 2.26)

2.22-2.25 আমি আপনাকে কতোগুলো বয়স বলবো এবং জানতে চাইবো সেই বয়সের মানুষ ইনফ্লুয়েঞ্জা রোগে মারা যেতে পারে কি না?

**SAY,** I shall tell you some age groups and ask you if you think that they may die from Influenza illness.

Yes (n¨uv) 01

No (bv) 02

Don’t Know/Not Sure (Rvwb bv/ wbwðZ bB) 99

| 2.22 Avcwb wK g‡b K‡ib 5 eQ‡ii Kg eq‡mi wkïi ‡¶‡Î Bbd¬z‡qÄv †iv‡M f~‡M g„Zz¨ NUvi m¤¢ve আছে?  Do you think it is likely that ­­a child less than 5 years old with influenza would die ? |  |
| --- | --- |
| 2.23 Avcwb wK g‡b K‡ib 5 †_‡K 1৭ eQর eqmx ¯‹z‡j hvIqv wkïi †¶‡Î Bbd¬z‡qÄv †iv‡M f~‡M g„Zz¨ NUvi m¤¢vebv আছে?  Do you think it is likely that ­­a child 5-17 years old with influenza would die ,? |  |
| 2.24 Avcwb wK g‡b K‡ib cÖvßeq¯‹ gvby‡li †¶‡Î Bbd¬z‡qÄv †iv‡M f~‡M g„Zz¨ NUvi m¤¢vebv আছে?  Do you think it is likely that ­­a person with influenza would die if they are an adult? |  |
| 2.25 Avcwb wK g‡b K‡ib e„× e¨w³i †¶‡Î Bbd¬z‡qÄv †iv‡M f~‡M g„Zz¨ NUvi m¤¢vebv আছে?  Do you think it is likely that ­­a person with influenza would die if they are an elderly person? |  |

| 2.26 Avcwb ev Avcbvi **Lvbvi** †jvKRb Bbd¬z‡qÄv wel‡q LeivLei †Kgb K‡i ‡c‡q _v‡Kb?  **ASK**, How you or the people living in your household obtained information about influenza?  *(cQ›`¸‡jv D”P¯^‡i c‡o †kvbv‡eb bv)*  *G cÖkœwU †Lvjv cÖ‡kœi gZ K‡i wR‡Ám Ki“b, DËi`vZv নিচের ZvwjKvi লক্ষণ¸‡jv D‡jøL Ki‡j n¨uv =1 , bv =2, †KvW Kiyb)|*  *(DO NOT READ RESPONSE OPTIONS ALOUD)*  *(Ask this as an open-ended question. If the respondent mentions the listed symptom, write “1” for “yes” “2” for “no” .)*  ***--লক্ষ্য করুন:*** *প্রাইমারী রেসপন্ডেন্ট যে উত্তর দেয়, শুধু তাই রেকর্ড করুন।*  ***--NOTE:*** *Please only record responses that the primary respondent says.* | Yes 01  No 02 |
| --- | --- |
| a. (†iwWI) Radio |  |
| b.. (†Uwjwfkb Television |  |
| c.. (Le‡ii KvMR) Newspaper |  |
| d.. (হাসপাতাল/Dc‡Rjv ¯^v¯’¨ †K›`ª ev ¯^v¯’¨ Kgx© Wv³vi /bvm©) Health professional - Hospital, Upazilla Health Center/Healthcare provider (doctor, nurse), Community health worker or pharmacist |  |
| e. (eÜz-evÜe, mnKg©x, evwoi m`m¨, প্রতিবেশী) Social Circle - Family and friends, Co(w)-workers, Bari members or neighbors |  |
| f. (agxq †bZv ev gmwR`/gw›`i) Religious leader or church/temple |  |
| g. ক্লিনিক্যাল সেটিং বা ফিল্ডে বিসটিস দলের সঙ্গে যোগাযোগের মাধ্যমে  Contact with BISTIS team in field or in clinical setting (current or previous contact) |  |
| h. ক্লিনিক্যাল সেটিং বা ফিল্ডে আইসিডিডিআর, বি (বিসটিস নয়) দলের সঙ্গে যোগাযোগের মাধ্যমে  Contact with ICDDR,B in field or clinical setting (other than BISTIS project) |  |
| i.স্কুল/School |  |
| j. (Avwg Bbd¬z‡qÄv wel‡q †Kv‡bv Z_¨ cvB bvB) I have not received any information about influenza |  |
| k. (Rvwb bv/wbwðZ bB) Don’t know/not sure |  |
| l. (উত্তরদাতা প্রশ্ন বোঝেন নাই এবং কোনো উত্তর নাই)  Respondent does not understand question and has no response |  |
| m. (Ab¨vb¨/wbw`©ó Ki“b) Other (specify)  __________________________________________________________________________ |  |

2.27 weMZ 6 gv†m Lvbvi †Kv‡bv m`m¨ Bbd¬‡qÄv ev **সোয়াইন ফ্লূ** wUKv wb‡qwQ‡jv wK? 

ASK: Has anyone in your household received a vaccination for influenza or swine flu in the past six months?

Yes (n¨uv) 01

No (bv) 02

Don’t Know/Not Sure (Rvwb bv/ wbwðZ bB) 99

- *DËi hw` nu¨v nq, Z‡e †h ev hviv wUKv wb‡qwQ‡jv, Zviv †Kv_vq wb‡qwQ‡jv D‡j­L Ki“b| j¶¨ ---****Ki“b****: GwU n‡jv wUKv cÖ`v‡bi ¯’vb, †hgb ¯‹z‡j, Kv‡Ri ¯’v‡b BZ¨vw` kix‡ii †Kv‡bv ¯’vb bq|*
- *If yes, please indicate where that person (those people) received the vaccine:*

***--NOTE****: This is the location, such as school, clinic, etc., where the vaccine was obtained (not the location on the body)*

______________________________________________________________

🡪 DËi (02) ev (99) n‡j cÖkœ bs 2.28 G P‡j hvb (If 02 or 99, SKIP to question 2.28)

| 2.27b wR‡Ám Ki“b: Avcwb wK Avgv‡K wUKv KvW© †`Lv‡Z cv‡ib?   †imc‡Û›U wK Avcbv‡K KvW© †`wL‡q‡Q?  ASK: Can you show me the vaccination card?  Does respondent show you?  Yes (n¨uv) 01  No (bv) 02  Don’t Know/Not Sure (Rvwb bv/ wbwðZ bB) 99 |
| --- |

2.28 hw` DËi`vZv AvBwmwc **না** nq, **তবে** wR‡Ám Ki“b MZ GK gv‡mi g‡a¨ Avcbvi R¡i n‡qwQ‡jv wK? 

hw` DËi`vZv AvBwmwc nq **তবে** wR‡Ám Ki“b eZ©gvb **জ্বর** Qvov MZ GK gv‡mi g‡a¨ Avcbvi

R¡i n‡qwQ‡jv wK?

*If respondent is NOT the ICP* 🡪**ASK:** Have you had a fever within the last month?

*If respondent is the ICP* 🡪**ASK:** Apart from this current fever, have you had a fever within the last month?

*--লক্ষ্য করুন: এক মাস পূর্বে তারিখ কতো ছিলো, তা আপনি জানাতে পারেন।*

*--NOTE: You may indicate what the date was one month prior.*

Yes (n¨uv) 01

No (bv) 02

Don’t Know/Not Sure (Rvwb bv/ wbwðZ bB) 99

🡪 hw` DËi 02 ev 99 nq, cÖkœ 2.43-G hvb| (If 02 or 99, SKIP to question 2.43)

| 2.29-2.42 wR‡Ám Ki“b MZ GK gv‡mi g‡a¨ hLb Avcbvi R¡i n‡qwQ‡jv, wb‡Pi KvR¸‡jvi GKwUI K‡iwQ‡jb wK?  **এরপর একটি একটি করে অপশন পড়ে শোনান এবং উত্তর রেকর্ড করুন।**  **ASK,** “When you had a fever within the last month, did you do any of the following?”  *Then read choices one at a time and record the respondent’s responses.*  *🡪* cÖ‡qvRb n‡j †evSvi myveav‡_© wR‡Ám Ki“bÓ MZ GKgv‡mi g‡a¨ hLb Avcbvi R¡i n‡qwQj Avcwb wK---------------?  *🡪 If necessary, for clarification at any time,* **ASK***,* “When you had a fever within the last month___________?”   | Yes (n¨uv) 01  No (bv) 02  **উত্তরদাতা প্রশ্ন বোঝেন নাই এবং কোনো উত্তর নাই**  Respondent does not understand question and has no response 33  Don’t Know/Not Sure (Rvwb bv/ wbwðZ bB) 99 | | | --- | --- | | **2.29 স্বাভা্বিক সময়ের চেয়ে বেশি বেশি nvZ ধুয়েছিলেন?**  Did you wash your hands more frequently than usual? |  | | 2.30 nuvwP ev Kvwki mg‡q Kvco ev KbyB w`‡q gyL **ঢেকেছিলেন?**  Did you cover your cough or sneeze with tissue or your elbow? |  | | **2.31 wK¬wbK ev nvmcvZvj গিয়েছিলেন?**  Did you visit a clinic or hospital? |  | | **2.32 Ab¨ †jv‡K‡`i Lye KvQvKvwQ যান নি?**  Did you avoid close contact with other people? |  | | **2.33 ¯‹z‡j/Kv‡R/evRv‡i/KwgDwbwU †m›Uv‡i bv wM‡q evwo‡Z থেকেছিলেন?**  Did you stay home from work/school/market/community centers? |  | | **2.34 Wv³v‡ii †cÖmwµckb QvovB Jla †L‡qwQ‡jb? (Ila we‡µZv †_‡K ev †Kvb dvg©vwm÷ ‡_‡K ev wb‡R wb‡R)**  Did you take medicine without a doctor’s prescription (from a medicine seller, a pharmacist or self-medicated)? |  | | **2.35 এমবিবিএস বা অন্য সার্টিফিকেটধারী চিকিৎসকের (যেমন ফার্মেসীস্ট,প্যারামেডিক,নার্স, মেডিকেল এসিসটেন্ট, পরিবারপরিকল্পনা পরামর্শক ইত্যাদি) Dc‡`k Abyhvqx Jla খেয়েছিলেন?**  Did you seek help or take medicine prescribed by a MBBS doctor or other certified medical professionals paramedics, nurses, medical assistants, family welfare visitors, etc.)? |  | | **2.36b ‡nvwgIc¨vw_K Wv³v‡ii বা কবিরাজী/ঝাড়ফুক ইত্যাদির civgk© Abyhvqx Jla খেয়েছিলেন?**  Did you seek help or take medicine from a homeopathic doctor or a traditional healer? |  | | 2.38 †Rjv nvmcvZvj †_‡K webvg~‡j¨ *A‡mëvwgwfi* Jla পেয়েছিলেন?  Did you receive free Oseltamivir from a district hospital? |  | | **2.39 ঘরের evqy PjvPj বাড়ানোর ব্যবস্তা করেছিলেন?**  Did you increase ventilation in your household? |  | | **2.40 Ab¨‡`i সাথে একই _vjv-evm‡b Lvevi bv Lvন নি?**  Did you avoid sharing drink or utensils with others? |  | | 2.41 Ab¨‡`i ‡_‡K Avjv`v Ny**মিয়েছেন?**  Did you sleep separately from others? |  | | 2.42 Ab¨vb¨ (wbw`©ó Ki“b) Other (specify): |  | |
| --- | --- | --- | --- | --- | --- | --- | --- | --- | --- | --- | --- | --- | --- | --- | --- | --- | --- | --- | --- | --- | --- | --- | --- | --- | --- | --- | --- | --- |

[2.43-2.58] BwbDwg‡ikb dg© †`‡L †ei Ki“b Lvbvq (nvDR‡nv‡ì) 5 eQ‡ii Kg eqmx †Kv‡bv wkï i‡q‡Q wK bv| hw`

_v‡K, Z‡e wb‡Pi cÖkœwU Ki“b| hw` bv _v‡K, Z‡e cÖkœ bs 2.59 G P‡j hvb|

*Check Enumeration Form to see if there is a child who is less than 5 years of age in the household.*

*🡪If there is a child under 5, ask this question:*

*🡪If there is no child less than 5 years old,* ***SKIP*** *to question 2.59*

**2.43 Avcwb wK cÖavbZ একজন 5 eQ‡ii Kg eqmx wkïi †`Lv‡kvbvকারী ev প্রাথমিক cwiPh©vকারী?** 

Are you a primary caretaker for a child that is less than 5 years of age?

Yes (n¨uv) 01

No (bv) 02

🡪 hw` 02 DËi nq Z‡e 2.59G P‡j hvb| If 02, SKIP to question 2.59

2.44 hw` 5 eQ‡ii Kg eqmx wkï AvBwmwc **না** nq, Z‡e wR‡Ám Ki“Y Avcbvi cwiPh©vq _vKv 5 eQ‡ii Kg eqmx †Kv‡bv wkïi MZ GK gv‡m R¡i n‡qwQ‡jv wK?

**hw` 5 eQ‡ii Kg eqmx wkï AvBwmwc nq , তবে wR‡Ám Ki“Y (আইসিপির নাম)এর) eZ©gvb জ্বর Qvov Avcbvi cwiPh©vq _vKv 5 eQ‡ii Kg eqmx অন্য†Kv‡bv wkïi MZ GK gv‡m R¡i n‡qwQ‡jv wK?**



*If child less than 5 years old is NOT the ICP*, 🡪**ASK:** Has a child under your care, who is less than 5 years old, had a fever in the past month?

*If child less than 5 years old is the ICP*, 🡪**ASK:** Apart from (name of ICP)’s current fever, has (name of ICP) or another child under your care, who is less than 5 years old, had a fever in the past month?

***-লক্ষ্য করুন:*** *এক মাস পূর্বে তারিখ কতো ছিলো, তা আপনি জানাতে পারেন।*

***--NOTE****: You may indicate what the date was one month prior.*

Yes (n¨uv) 01

No (bv) 02

Don’t know/Refused to answer (Rvwb bv/ DËi †`‡eb bv) 99

🡪 hw` DËi 02 ev 99 nq, cÖkœ 2.59-G hvb| If 02 or 99, SKIP to question 2.59

| [2.45-2.58] wR‡Ám Ki“Y Avcbvi 5 eQ‡ii Kg eqmx mšÍv‡bi MZ GK gv‡mi g‡a¨ hLb R¡i n‡qwQ‡jv, ZLb wb‡Pi KvR¸‡jvi **একটিও** K‡iwQ‡jb wK?  🡪এরপর এক এক করে অপশন পড়ে শোনান এবং উত্তর রেকর্ড করুন।  **ASK,** “When a child under your care, who is less than 5 years old, had a fever within the last month, did you do any of the following?” *Then read choices one at a time and record the respondent’s responses.*  *🡪* cÖ‡qvRb n‡j †evSvi myveav‡_© wR‡Ám Ki“bÓ MZ GKgv‡mi g‡a¨ hLb GB wkïwUi R¡i n‡qwQj Avcwb wK---------------?  *🡪If necessary, for clarification at any time,* **ASK,** “When this child had a fever within the last month,_______________?”  -***j¶¨ Ki“b:*** *hw` DËi`vZvi cwiPh©vq G‡Ki AwaK wkï _v‡K hv‡`i MZ GK gv‡m R¡i n‡qwQ‡jv, †m‡¶‡Î me‡P‡q Kg eqmx wkïi †¶‡Î DËi cÖ‡hvR¨ n‡e|*  *-****NOTE:*** If respondent has more than one child under their care who had a fever within the last month, the answers will be applicable for the youngest child.   | Yes (n¨uv) 01  No (bv) 02  উত্তরদাতা প্রশ্ন বোঝেন নাই এবং কোনো উত্তর নাই  Respondent does not understand question and has no response 33  Don’t Know/Not Sure (Rvwb bv/ wbwðZ bB) 99 | | | --- | --- | | **2.45 স্বাভাবিক সময়ের চেয়ে বেশি বেশি nvZ ধুয়েছিলেন?**  Did you wash your hands more frequently? |  | | 2.46 Avcbvi wkïwU wK¬wbK ev nvmcvZv‡j wM‡qwQj?  Did your child visit a clinic or hospital? |  | | **2.47 Avcbvi wkï‡K Ab¨ †jv‡K‡`i Lye KvQvKvwQ রাখেন নি?**  Did you have your child avoid close contact with other people? |  | | **2.48 Avcbvi wkï‡K ¯‹z‡j/Kv‡R/evRv‡i/KwgDwbwU †m›Uv‡i bv wb‡q evwo‡Z রেখেছিলেন?**  Did you keep your child home from work/school/market/community centers? |  | | **2.49 Wv³v‡ii †cÖmwµckb QvovB dvgv©mx †_‡K Jla wK‡b wkï‡K Lvইয়েছেন ev N‡i ivLv Jla Lvইয়েছেন?**  Did you give your child medicine from home or bought at a pharmacy without a doctor’s Prescription (self-medicate)? |  | | **2.50 Jlawe‡µZvi (mvwU©wd‡KU cvIqv dv‡g©mx÷ bb) m‡½ civgk© K‡i Jla খাইয়েছেন?**  Did you seek help or medicine prescribed by a medicine seller (not a certified pharmacist)? |  | | **2.51এমবিবিএস বা অন্য সার্টিফিকেটধারী চিকিৎসকের (যেমন ফার্মেসীস্ট,প্যারামেডিক,নার্স, মেডিকেল এসিসটেন্ট, পরিবারপরিকল্পনা পরামর্শক ইত্যাদি) Dc‡`k Abyhvqx Jla খেয়েছিলেন?**  Did you seek help or take medicine prescribed by a MBBS doctor or other certified medical professionals (certified pharmacist, paramedics, nurses, medical assistants, family welfare visitors, etc.)? |  | | **2.52 ‡nvwgIc¨vw_K Wv³v‡ii বা কবিরাজী/ঝাড়ফুক ইত্যাদির civgk© Abyhvqx Jla খেয়েছিলেন?**  Did you seek help or medicine from a homeopathic doctor or a traditional healer? |  | | **2.53 †Rjv nvmcvZvj †_‡K webvg~‡j¨ *A‡mëvwgwfi* Jla পেয়েছিলেন?**  Did you receive free Oseltamivir from a district hospital? |  | | **2.54 ঘরের evqy PjvPj বাড়ানোর ব্যবস্থা করেছিলেন?**  Did you increase ventilation in your housing structure? |  | | **2.55 Ab¨‡`i সাথে একই _vjv-evm‡b wkï‡K Lvevi bv LvIqvন নি?**  Did you avoid sharing drink or utensils with your child? |  | | **2.56 Avcbvi wkï‡K Ab¨‡`i ‡_‡K ‡_‡K Avjv`v Nygv‡Z দিয়েছিলেন?**  Did you have your child sleep separately from others? |  | | 2.57 Ab¨vb¨ (wbw`©ó Ki“b) Other (specify): |  | |
| --- | --- | --- | --- | --- | --- | --- | --- | --- | --- | --- | --- | --- | --- | --- | --- | --- | --- | --- | --- | --- | --- | --- | --- | --- | --- | --- | --- | --- |

[2.59- 2.74] *BwbDwg‡ikb dg© †`‡L †ei Ki“b Lvbvq 5-17 eQi eqmx (5 Ges 17 eQimn) †Kv‡bv wkï i‡q‡Q wK bv| hw` _v‡K, Z‡e wb‡Pi cÖkœwU Ki“b| hw` bv _v‡K, Z‡e cÖkœ bs 2.84 G P‡j hvb|*

*Check Enumeration Form to see if there is a child who 5-17 years of age (including those that are 5 and 17) in the*

*household.*

*🡪If there is no child 5-17 years old,* ***SKIP*** *to question 2.84*

*🡪If there is a child 5-17 years old, ask this question:*

2.59 Avcwb wK cÖavbZ একজন 5-17 eQi eqmx wkïi †`Lv‡kvbvকারী ev প্রাথমিক cwiPh©vকারী? 

Are you a primary caretaker for a child that is 5-17 years of age?

Yes (n¨uv) 01

No (bv) 02

🡪 hw` **02** DËi nq Z‡e **2.75**G P‡j hvb| (If 02, SKIP to question 2.75)

2.60 hw` 5-17 eQi eqmx wkï AvBwmwc না nq তবে wR‡Ám Ki“b Avcbvi cwiPh©vq _vKv 5-17 eQi eqmx †Kv‡bv 

wkïi MZ GK gv‡m R¡i n‡qwQ‡jv wK?

hw` 5-17 eQi eqmx wkï AvBwmwc **হয়, তবে জিজ্ঞেস করুন, বর্তমান জ্বর ছাড়া (আইসিপির নাম)-এর বা অন্য কোনো**

**5-17 বছর বয়সী শিশু যাকে আপনি পরিচর্যা করেন, গত মাসে জ্বর হয়েছিলো কি?**

*If child between 5 and 17 years old is NOT the ICP*, 🡪**ASK:** Has a child under your care, who is between 5 and 17 years old, had a fever in the past month?

*If child between 5 and 17 years old is the ICP*, 🡪**ASK:** Apart from (name of ICP)’s current fever, has (name of ICP) or another child under your care, who is between 5 and 17 years old, had a fever in the past month?

***-লক্ষ্য করুন:*** *এক মাস পূর্বে তারিখ কতো ছিলো, তা আপনি জানাতে পারেন।*

***--NOTE****: You may indicate what the date was one month prior.*

Yes (n¨uv) 01

No (bv) 02

Don’t know/Refused to answer (Rvwb bv/ DËi †`‡eb bv) 99

🡪 hw` DËi 02 ev 99 nq, cÖkœ 2.75-G hvb| If 02 or 99, SKIP to question 2.75

| [2.61-2.74] wR‡Ám Ki“b Avcbvi পরিচর্যায় থাকা 5-17 eQ‡ii eqmx †Kv‡bv mšÍv‡bi hLb MZ মাসে R¡i n‡qwQ‡jv ZLb wb‡Pi KvR¸‡jvi †Kvb †KvbwU K‡iwQ‡jb?  🡪এরপর এক এক করে অপশন পড়ে শোনান এবং উত্তর রেকর্ড করুন। প্রয়োজনবোধে প্রশ্নটি প্রতিটি অপশনের সঙ্গে প্রশ্নটি পড়তে পারেন।  **ASK,** “When a child under your care, who is 5-17 years old, had a fever within the last month, did you do any of the following?” *Then read choices one at a time and record the respondent’s responses.*  *🡪If necessary, for clarification at any time,* **ASK,** “When this child had a fever within the last month,_______________?”  -***j¶¨ Ki“b:*** *hw` DËi`vZvi cwiPh©vq 5-17 eQi eqmx G‡Ki AwaK wkï _v‡K hv‡`i MZ GK gv‡m R¡i n‡qwQ‡jv, †m‡¶‡Î me‡P‡q Kg eqmx wkïi †¶‡Î DËi cÖ‡hvR¨ n‡e|*  *-****NOTE:*** *If respondent has more than one child, between 5-17 years old, under their care wh*o had a fever within the last two weeks, please report on their actions regarding the YOUNGEST of those children.   | Yes (n¨uv) 01  No (bv) 02  উত্তরদাতা প্রশ্ন বোঝেন নাই এবং কোনো উত্তর নাই  Respondent does not understand question and has no response 33  Don’t Know/Not Sure (Rvwb bv/ wbwðZ bB) 99 | | | --- | --- | | 2.61 স্বাভাবিকের চেয়ে বেশি বেশি nvZ ধুয়েছিলেন?  Did you wash your hands more frequently? |  | | 2.62 Avcbvi wkïwU wK¬wbK ev nvmcvZv‡j wM‡qwQj?  Did your child visit a clinic or hospital? |  | | 2.63 Avcbvi wkï‡K Ab¨ †jv‡K‡`i Lye KvQvKvwQ রাখেন নি?  Did you have your child avoid close contact with other people? |  | | 2.64 Avcbvi wkï‡K ¯‹z‡j/Kv‡R/evRv‡i/KwgDwbwU †m›Uv‡i bv wb‡q evwo‡Z রেখেছিলেন?  Did you keep your child home from work/school/market/community centers? |  | | 2.65 Wv³v‡ii †cÖmwµckb QvovB dvgv©mx †_‡K Jla wK‡b wkï‡K LvIqv‡bv ev N‡i ivLv Jla Lvইয়েছেন?  Did you give your child medicine from home or bought at a pharmacy without a doctor’s Prescription (self-medicate)? |  | | 2.66 Jlawe‡µZvi (mvwU©wd‡KU cvIqv dv‡g©mx÷ bb) m‡½ civgk© K‡i Jla খাইয়েছেন?  Did you seek help or medicine prescribed by a medicine seller (not a certified pharmacist)? |  | | 2.67 এমবিবিএস বা অন্য সার্টিফিকেটধারী চিকিৎসকের (যেমন ফার্মেসীস্ট,প্যারামেডিক,নার্স, মেডিকেল এসিসটেন্ট, পরিবারপরিকল্পনা পরামর্শক ইত্যাদি) Dc‡`k Abyhvqx Jla খাইয়েছিলেন?  Did you seek help or medicine prescribed by a MBBS doctor or from other certified medical professionals (certified pharmacist, paramedics, nurses, medical assistants, family welfare visitors, etc.)? |  | | 2.68 ‡nvwgIc¨vw_K Wv³v‡ii বা কবিরাজী/ঝাড়ফুক ইত্যাদির civgk© Abyhvqx Jla খাইয়েছিলেন?  Did you seek help or medicine from a homeopathic doctor or a traditional healer? |  | | 2.70 †Rjv nvmcvZvj †_‡K webvg~‡j¨ *A‡mëvwgwfi* Jla পেয়েছিলেন?  Did you receive free Oseltamivir from a district hospital? |  | | 2.71 evwoi evqy PjvPj বাড়ানোর ব্যবস্থা করেছিলেন?  Did you increase ventilation in your household? |  | | 2.72 Ab¨‡`i সাথে একই _vjv-evm‡b wkï‡K Lvevi খাওয়ান নি?  Did you avoid sharing drink or utensils with your child? |  | | 2.73 Avcbvi wkï‡K Ab¨‡`i ‡_‡K ‡_‡K Avjv`v Nygv‡Z দিয়েছিলেন?  Did you have your child sleep separately from others? |  | | 2.74 Ab¨vb¨ (wbw`©ó Ki“b) Other (specify): |  | |
| --- | --- | --- | --- | --- | --- | --- | --- | --- | --- | --- | --- | --- | --- | --- | --- | --- | --- | --- | --- | --- | --- | --- | --- | --- | --- | --- | --- | --- |

[2.75-2.83]Avwg Avcbv‡K wKQy AvPiY m¤ú©‡K ej‡ev `qv K‡I, Avgv‡K ejyb wb‡Pi †Kvb †Kvb e¨envi Avcbv‡K **শ্বাস-কাশির** †ivM **(যেমন জ্বর, কাশি, গলাব্যথা, নাক ঝরা, শ্বাসকষ্ট ইত্যাদি)** Qov‡bvi nvZ †_‡K euvP‡Z **সাহায্য করে?**

**SAY,** I shall tell you about some behaviors. For the following, tell me if you think that the behavior would help you avoid the spread of repiratory diseases (including such as fever, cough, sore throat, runny nose, breathing difficulties).

cÖ‡hvR¨ †¶‡Î wR‡Ám Ki“b: ÒAvcwb wK g‡b K‡ib _______ Avcbv‡K †ivM Qov‡bvi †_‡K euvP‡Z সাহায্য করে?

🡪 *If necessary, for clarification at any time,* **ASK** Do you think __________ help you avoid the transmission of disease ?

| Yes (n¨uv) 01  No (bv) 02  Don’t Know/Not Sure (Rvwb bv/ wbwðZ bB) 99 | |
| --- | --- |
| 2.75 Amy¯’ †jv‡Ki Lye KvQvKvwQ bv **যাওয়া**  Avoiding close contact with persons that are sick? |  |
| 2.76 cvwb w`‡q nvZ †avqv  Washing your hands with water? |  |
| 2.77 mvevb **পানি** w`‡q nvZ †avqv  Washing your hands with soap and water? |  |
| 2.78 evRvi Ges Ab¨ Rbeûj ¯’vb Gwo‡q Pjv  Avoiding going to the market and other public meeting places? |  |
| 2.79 †KD Amy¯’ n‡j N‡ii evqy PjvPj evov‡bvi e¨e¯’v Kiv  Increasing ventilation in the household when a member is sick? |  |
| 2.80 nvZ bv †gjv‡bv**/হ্যান্ডশেক না করা**  Avoid shaking hands? |  |
| 2.81 Amy¯’ e¨w³‡K Avjv`v ivLv  Isolating a sick person from other individuals? |  |
| 2.82 wUKv †bqv  Getting a vaccine? |  |
| 2.83 gvm&K **বা অন্য কিছু দিয়ে মুখ-নাক ঢেকে রাখা *(হাত দিয়ে মুখ ঢেকে উত্তরদাতাকে দেখান)***  Covering your face or nose with a mask or other cloth? (PUT HAND OVER MOUTH TO DEMONSTRATE) |  |

**[2.84-2.93] আমি আপনাকে কিছু বাধা সম্পর্কে বলবো যেগুলো হয়তো রোগ থেকে নিজে বা পরিবারকে বাচাতে আপনার জন্যে সমস্যার কারণ হয়ে দেখা দিয়েছিলো।**

**বর্তমান অসুস্থতা ছাড়া** ‡klevi hLb Avcwb ev Avcbvi Lvbvi †Kv‡bv wkï Amy¯’ n‡qwQ‡jv, ZLb †iv**গের সংক্রমণ** ‡VKv‡bvi R‡b¨ ev wPwKrmvi ‡¶‡Î wb‡Pi †Kv**নো**evavi m¤§yLxb n‡qwQ‡jb? (Ackb¸‡jv c‡o †kvbvb)

**SAY**, I shall tell you some barriers which might become a problem for protecting yourself and your family from diseases. Think about the last time (apart from this current illness) you or a child from your household was sick. Please tell me if you encountered any of the following barriers to preventing or treating the disease.

*(READ RESPONSE OPTIONS )*

cÖ‡qvRb n‡j †evSvi myveav‡_© wR‡Ám Ki“b †klevi hLb Avcwb ev Avcbvi Lvbi †Kvb wkï Amy¯’ n‡qwQj ZLb wK--------------?*🡪If necessary, for clarification at any time,* **ASK**, “The last time you or a child from your household was sick _________________________________?”

| Yes (n¨uv) 01  No (bv) 02  **উত্তরদাতা প্রশ্ন বোঝেন নাই এবং কোনো উত্তর নাই**  Respondent does not understand question and has no response 33  (cÖ‡hvR¨ bq) **এর প্রয়োজন ছিলো না** Not applicable (illness did not require this) 55  Don’t know/ Don’t remember (Rvwb bv/g‡b †bB) 99 | |
| --- | --- |
| 2.84 wPwKrmvi LiP †ewk **ছিলো?**  Was healthcare too expensive? |  |
| 2.85 Jl‡ai `vg Lye †ewk **ছিলো?**  Was medicine too expensive? |  |
| 2.86 wbR‡K ev cwievi‡K wKfv‡e †iv‡Mi nvZ †_‡K euvPv‡eb †m m¤ú‡K© LeivLe‡ii Afve **ছিলো?**  Was there was a lack of information about what you could do to protect yourself or your family? |  |
| 2.87 ‡iv‡Mi nvZ †_‡K euvPvi Dcvq¸‡jv m¤ú‡K© cwi®‹vi Lei cvb bvB?  Was information received about how to prevent or treat disease unclear? |  |
| 2.88 nvZ †avqvi cvwb wQ‡jv bv?  Was water to wash hands was not available? |  |
| 2.89 nvZ †avqvi mvev‡bi `vg †ewk **ছিলো** A_ev cvIqv hv**য় নি?**  Was soap to clean hands was too expensive or not available? |  |
| 2.90 Kv‡Ri R‡b¨ evwo‡Z _vKv m¤¢e nq wb?  Were you unable to stay in the house because you had to go to work? |  |
| 2.91 evmvq wkï‡`i ‡`Lvi †KD wQ‡jv bv weavq Zv‡`i evwo‡Z ivLv m¤¢e nq wb?  Were you unable keep the kids at home because there was nobody to watch them? |  |
| 2.92 Avjv`v K¶ bv _vKvq Amy¯’ e¨w³‡K Avjv`v ivLv m¤¢e nq wb ?  Were you unable to isolate sick people because there is no separate room in your home? |  |
| 2.93 Ab¨vb¨, wbw`©ó Ki“b Other (Specify: _____________________) |  |

**‡mKkb 3t ivbœvNi I cÖavb Ny‡gi N‡ii evqy mÂvjb e¨e¯’vi g~j¨vqb**

**Section 3: Ventilation Assessment of Cooking area and Main Sleeping Place**

DËi`vZv‡K cÖkœ Kiæb t Ò`qv K‡i Avcwb Avgv‡K Avcbvi Lvbvi ivbœv Kivi ¯’vbwU †`Lv‡eb wK?Ó

**Ask to respondent: “Can you show me the cooking area that your household uses most often?”**

***j¶¨ Ki“b:*** *ivbœvi ¯’vb ej‡Z ‡ewk e¨eüZ PzjvwU †h ¯’v‡b Aew¯’Z Zv‡K eySv‡bv n‡q‡Q|*

***--NOTE: Cooking area is defined by the location of the primary stove.***

**3.1** ch©‡e¶b: ivbœvN‡ii Ae¯’vb (ev‡´ †KvW wjLyb ) 

**Observation: Cooking area location (Record code in box):**

evm¯’v‡bi Pvi †`Iqv‡ji wfZ‡i

(Within four walls of housing structure)……. 01

evm¯’v‡bi cv‡k Ges mivmwi evm¯’v‡bi mv‡_ mshy³

(Attached to and directly next to the housing structure) 02

evm¯’v‡bi cv‡k wKš‘ mivmwi evm¯’v‡bi mv‡_ mshy³ bq

(Attached to but NOT directly next to the housing structure) 03

***j¶¨ Ki“b: GB †KvWwU e¨eüZ n‡e hLb ivbœvi ¯’vbwU N‡ii KvVv‡gvi mv‡_ mivmwi mshy³ wKšÍ ga¨eZ©x Ab¨ †Kvb K¶ i‡q‡Q (†hgb †÷vi i“g A_ev Mi“ QvM‡ji Ni) hv †kvevi Ni I ivbœvi ¯’vb‡K Avjv`v Ki‡Q***

--NOTE: Use this code when the cooking area is attached to the housing structure, but there is some other room

(such as a storage area or animal room) separating the cooking area from where people sleep

evm¯’vb †_‡K Avjv`v

(Separate from living space) 04

***j¶¨ Ki“b: ivbœvi ¯’vb I †kvevi N‡ii ga¨eZ©x ‡h‡Kvb cwigvb Lvwj RvqMv _vK‡j*** 04 ***†KvW© cÖ‡hvR¨ n‡e***

--NOTE: Any separation between the cooking area and the housing structure should be coded as “04”

Ab¨vb¨, wb‡P wbw`©ó K‡i wjLyb

(Other, specify below) 99

_____________________________________________________________________________

**3.2a** ch©‡e¶b: ivbœvN‡ii QvDwb ev Pvjvi aib (ev‡´ †KvW wjLyb ) 

**Observe: Roof of cooking area (Record number in box):**

*--লক্ষ্য করুন: †h †Kv‡bv ai‡bi †`qvj GgbwK Kvc‡oi ˆZix n‡jI Zv Ò†`qvjÓ iæ‡c Mb¨ n‡e*

***--NOTE: Any material, even a tarp, should be considered a “roof”***

Pvjv †bB No roof 01

AvswkK Pvjv Av‡Q Partial roof present 02

cwic~Y© Pvjv Av‡Q Complete roof present 03

🡪 hw` DËi 3.2a Ò01Ó nq, Z‡e **3.6**bs cÖ‡kœ P‡j hvb **If answer to 3.2b is “01”, then SKIP to 3.6**

**3.2b** ch©‡e¶b:ivbœvN‡ii ‡`qv‡ji aiY (ev‡· †KvW wjLyb) 

**Observe: Walls of cooking area (Record number in box):**

-jÿ¨ Kiæb: †h †Kv‡bv ai‡bi †`qvj GgbwK Kvc‡oi ˆZix n‡jI Zv Ò†`qvjÓ iæ‡c Mb¨ n‡e

**--NOTE: Any material, even a tarp, should be considered a “wall”**

-jÿ¨ Kiæb: hw` Kgc‡ÿ¨ GKwU †`qvj AvswkK nq (A‡a©K D”PZvi), Ò2Ó wjLyb|

**--NOTE: If at least one wall is a partial wall (1/2 height), select “02”**

†Kv‡bv †`qvj †bB

(No walls) 01

AvswkK †`qvj (AvswkK eÜ)

[Partial walls (partially enclosed)] 02

c~Y© †`qvj (`‡ivRv Ges Rvbvjv ev‡` cy‡ivcywi eÜ)

[Full walls (completely enclosed (besides doors and windows)] 03

Ab¨vb¨ (wb‡P wbw`©ó Kiæb)

[Other (specify below)] 99

________________________________________________________________________

🡪 hw` DËi 3.2b Ò1Ó nq, Z‡e **3.7**bs cÖ‡kœ P‡j hvb **If answer to 3.2b is 1, then SKIP to 3.7**

🡪 hw` DËi 3.2b Ò2Ó ev Ò9Ó nq, Z‡e **3.6**bs cÖ‡kœ P‡j hvb **If answer to 3.2b is 2 or 9, then SKIP to 3.6**

**3.3** ch©‡e¶b:ivbœvN‡ii evqy mÂvjb e¨e¯’v (ev‡´ †KvW wjLyb ) 

**Observe: Ventilation of the cooking area (Record code in box):**

**--** ***j¶¨ Ki“b:***: †Kv‡bv Ò4Ó bs †KvW †bB|

--**NOTE: There is no choice “4”**

***j¶¨ Ki“b:¯’vqxfv‡e eÜ Rvbvjv ev `iRv Mb¨ n‡e bv***

--NOTE: Do not count windows or doors that appear to be permanently closed or blocked.

***j¶¨ Ki“b:Ab¨ N‡ii mv‡_ ms‡hvMKvix Rvbvjv ev `iRv Mb¨ Ki‡Z n‡e***

--NOTE: Doorways or windows leading to other rooms should be observed for this question.

ivbœvN‡ii Pvi w`‡K Kgc‡¶ GKwU K‡i Rvbvjv A_ev `iRv Av‡Q

At least one window or door in each of the 4 directions (in each of four walls) 01

ivbœvN‡ii wZb w`‡K Kgc‡¶ GKwU K‡i Rvbvjv A_ev `iRv Av‡Q

At least one window or door in each of 3 directions (in three walls) 02

ivbœvN‡ii wecixZ `yB w`‡K Kgc‡¶ GKwU K‡i Rvbvjv A_ev `iRv Av‡Q (DËi I `w¶Y)

At least one window or door in each of 2 opposing directions (in two opposing walls) 03

ivbœvN‡ii ‡h‡Kvb non-wecixZ w`‡K Kgc‡¶ GKwU K‡i Rvbvjv A_ev `iRv Av‡Q

At least one window or door in each of 2 non-opposing directions (in two non-opposing walls) 05

ivbœvN‡ii GKB w`‡K gyL K‡i `yBwU Rvbvjv ev `iRv Av‡Q (GKB †`qv‡j)

At least two windows/doors in only 1 direction (in one wall) 06

ivbœvN‡ii GKB w`‡K gyL K‡iGKwU Rvbvjv ev `iRv Av‡Q (GKB †`qv‡j)

Only one door or window in only 1 direction (in one wall) 07

Ab¨vb¨, wb‡P wbw`©ó K‡i wjLyb

Other (specify below) 99

__________________________________________________________________________

**3.4** ch©‡e¶Y: ivbœvN‡ii evq~ mÂvjb e¨e¯’v, †`Iqvj I Qv‡`i ga¨eZ©x RvqMv (ev‡´ †KvW wjLyb) 

**Observe: Ventilation of cooking area, space between walls and roof (Record code in box):**

--ga¨eZ©x RvqMv ej‡Z †`qvj I Qv‡`i g‡a¨ e¨eavb †evSv‡bv n‡q‡Q hv Avcbvi nv‡Zi cvÄvi me‡P‡q PIov ¯’v‡bi mgvb ev †ewk (সবচেয়ে চওড়া অংশটি মাপুন।)

**--NOTE: A space is defined as any separation between the wall/partition and roof that is equal or greater to the widest part of your hand (measure the widest space)**

PviwU †`Iqvj I Qv‡`i ga¨eZ©x RvqMv Av‡Q Space between all 4 walls and roof………… 01

wZbwU †`Iqvj I Qv‡`i ga¨eZ©x RvqMv Av‡Q Space between 3 walls and roof………………. 02

`yBwU †`Iqvj I Qv‡`i ga¨eZ©x RvqMv Av‡Q Space between 2 walls and roof……………. 03

GKwU †`Iqvj I Qv‡`i ga¨eZ©x RvqMv Av‡Q Space between 1 wall and roof ……………. 04

‡Kvb †`Iqvj I Qv‡`i ga¨eZ©x RvqMv bvB No space between any walls and roof……… 05

**3.5** ch©‡e¶Y: ivbœvi ¯’v‡bi evqy PjvPj, †`qvj Ges †g‡Si ga¨eZ©x ¯’vb 

**Observe: Ventilation of cooking area, space between walls and floor (Record code in box)**

**--j¶¨ Ki“b**: ga¨eZ©x RvqMv ej‡Z †`qvj I †g‡Si g‡a¨ e¨eavb †evSv‡bv n‡q‡Q hv Avcbvi nv‡Zi cvÄvi me‡P‡q PIov ¯’v‡bi mgvb ev †ewk

**--NOTE: A space is defined as any separation between the wall/partition and floor that is equal or greater to the widest part of your hand**

PviwU †`Iqvj I †g‡Si ga¨eZ©x RvqMv Av‡Q Space between all 4 walls and floor…… 01

wZbwU †`Iqvj I †g‡Si ga¨eZ©x RvqMv Av‡Q Space between 3 walls and floor………… 02

`yBwU †`Iqvj I †g‡Si ga¨eZ©x RvqMv Av‡Q Space between 2 walls and floor………… 03

GKwU †`Iqvj I †g‡Si ga¨eZ©x RvqMv Av‡Q Space between 1 wall and floor………… 04

‡Kvb †`Iqvj I †g‡Si ga¨eZ©x RvqMv bvB No space between any walls and floor… 05

**3.6** ch©‡e¶Y: ivbœvi ¯’vb cwigvc, ˆ`N©¨ Ges cÖ¯’ (K`g/cv w`‡q) msL¨vq wjLyb

**Observe: Measurement of cooking space, length by width (in steps): Record Number Below**

***-- j¶¨ Ki“b****: me‡P‡q j¤^v ˆ`N©¨ Ges me‡P‡q PIov cÖ¯’ Mb¨ Kiæb|*

***--NOTE: Measure longest length and widest width present***

***j¶¨ Ki“b:AvswkK K`‡gi Rb¨ wbKU¯’ c~Y© wjLyb ( †hgb 5.5 Gi Rb¨ 6 wjLyb)***

***--NOTE: Round up to the nearest step (for example, record 5.5 steps as 06)***

 (‰`N©¨ length)  (cÖ¯’ width)

**3.7** ch©‡e¶Y: ivbœvi ¯’vb †_‡K evm¯’v‡bi `–iZ¡ K`g/cv w`‡q(ivbœvi ¯’v‡bi me‡P‡q Kv‡Qi N‡ii `iRv †_‡K Pzjv ch©šZ cwigvc Kiæb)

**Observation: Distance from cooking area to housing structure in steps (measure from the stove to the entrance of housing structure that most frequently entered when returning from the cooking area)Record number below:** 

***j¶¨ Ki“b****: ivbœv Kivi ¯’vb hw` Lvbvi wfZ‡i nq, Z‡e* ***00*** *†KvW Kiæb|*

***--NOTE: If the cooking area is inside the household, code 00.***

***j¶¨ Ki“b:AvswkK K`‡gi Rb¨ wbKU¯’ c~Y© wjLyb ( †hgb 5.5 Gi Rb¨ 6 wjLyb)***

**--NOTE: Round up to the nearest step (for example, record 5.5 steps as 06)**

**3.8** DËi`vZv wK AvBwmwc Lvbvi †jvK? 

**Is the respondent from the ICP household?**

Yes (n¨uv) 01

No (bv) 02

**🡪** DËi ÒbvÓ n‡j †mKkb **4**  G P‡j hvb **If the answer to question 3.8 is “no”, SKIP to section 4**

**3.9 `qv K‡i** (**Index ‡ivMx**) †h **NiwU‡ †ewkifvM mgq Nygvq ‡mwU Avgv‡K †`Lv‡eb wK**? 

**Ask the respondent: “Can you show me the room where the index case most often sleeps?”**

Yes (n¨uv) 01

No (bv) 02

**🡪** DËi ÒbvÓ n‡j †mKkb **4**  G P‡j hvb **If the answer to question 3.9 is “no”, SKIP to section 4**

**3.10** ch©‡e¶Y: Nygv‡bvi ¯’v‡bi evqy mÂvjb e¨e¯’v (ev‡´ †KvW wjLyb ) 

Observation: Ventilation of the sleeping space (Record code in box):

-- **j¶¨ Ki“b**: †Kv‡bv Ò4Ó bs AckY †bB|

--NOTE: There is no choice “4”

--Ny‡gi ¯’vb ej‡Z m¤¢ve¨ cÖv_wgK/Bb‡W· ‡ivMx †h ¯’v‡b Nygvq †mB ¯’vb‡K eySv‡bv n‡q‡Q

--NOTE: The “sleeping space” is the room in which the index case patient sleeps in. Count rooms as separate when you cannot see over the partition separating the two rooms while standing in front of partition

***j¶¨ Ki“b:¯’vqxfv‡e eÜ Rvbvjv ev `iRv Mb¨ n‡e bv***

*--NOTE: Do not count windows or doors that appear to be permanently closed or blocked.*

***j¶¨ Ki“b:Ab¨ N‡ii mv‡_ ms‡hvMKvix Rvbvjv ev `iRv Mb¨ Ki‡Z n‡e***

*--NOTE: Doorways or windows leading to other rooms should be included in this question.*

***j¶¨ Ki“b: AvBwmwc †h N‡i Nygvq Zv ch©‡e¶Y Ki“b***

*--NOTE: Observe the room in which the ICP sleeps.*

Nygv‡bvi N‡ii Pvi w`‡K Kgc‡¶ GKwU K‡i Rvbvjv A_ev `iRv Av‡Q

At least one window or door in each of the 4 directions (in each of four walls) 01

Nygv‡bvi N‡ii wZb w`‡K Kgc‡¶ GKwU K‡i Rvbvjv A_ev `iRv Av‡Q

At least one window or door in each of 3 directions (in three walls) 02

Nygv‡bvi N‡ii wecixZ `yB w`‡K Kgc‡¶ GKwU K‡i Rvbvjv A_ev `iRv Av‡Q (DËi I `w¶Y)

At least one window or door in each of 2 opposing directions (in two opposing walls) 03

Nygv‡bvi N‡ii ‡h‡Kvb non-wecixZ w`‡K Kgc‡¶ GKwU K‡i Rvbvjv A_ev `iRv Av‡Q

At least one window or door in each of 2 non-opposing directions (in two non-opposing walls) 05

Nygv‡bvi N‡ii GKB w`‡K gyL K‡i `yBwU Rvbvjv ev `iRv Av‡Q (GKB †`qv‡j)

At least two windows/doors in 1 direction (in one wall) 06

Nygv‡bvi N‡ii GKB w`‡K gyL K‡iGKwU Rvbvjv ev `iRv Av‡Q (GKB †`qv‡j)

Only one door or window in 1 direction (in one wall) 07

Ab¨vb¨, wb‡P wbw`©ó K‡i wjLyb

Other (specify below) 99

____________________________________________________________________

**3.11** ch©‡e¶Y: Nygv‡bvi N‡ii evqy mÂvjb e¨e¯’v, †`qvj Ges Qv‡`i ga¨eZx© ¯’vb (ev‡´ †KvW wjLyb ) 

**Observation: Ventilation of sleeping space, space between walls and roof (Record code in box):**

**--** **j¶¨ Ki“b**: ga¨eZ©x RvqMv ej‡Z ‡eov/†`qvj I Qv‡`i g‡a¨ e¨eavb †evSv‡bv n‡q‡Q hv Avcbvi nv‡Zi me‡P‡q PIov ¯’v‡bi mgvb ev †ewk

**--NOTE: A space is defined as any separation between the wall/partition and roof that is equal or greater to the widest part of your hand**

**--j¶¨ Ki“b**: সবচেয়ে বড়ো স্থানটিকে পরিমাপ করুন।

**--NOTE: Please observe the largest spaces and use these to determine whether a space is present or not.**

PviwU †`Iqvj I Qv‡`i ga¨eZ©x RvqMv Av‡Q Space between all 4 walls and roof………… 01

wZbwU †`Iqvj I Qv‡`i ga¨eZ©x RvqMv Av‡Q Space between 3 walls and roof………………. 02

`yBwU †`Iqvj I Qv‡`i ga¨eZ©x RvqMv Av‡Q Space between 2 walls and roof……………. 03

GKwU †`Iqvj I Qv‡`i ga¨eZ©x RvqMv Av‡Q Space between 1 wall and roof ……………. 04

‡Kvb †`Iqvj I Qv‡`i ga¨eZ©x RvqMv bvB No space between any walls and roof……… 05

**3.12** ch©‡e¶Y: g~j evm¯’v‡bi evqy mÂvjb e¨e¯’v, †eov/†`qvj I ‡g‡Si ga¨Kvi RvqMv (ev‡´ †KvW wjLyb|)

**Observation: Ventilation of main living room, space between walls and floor (Record code in box):**

--( **j¶¨ Ki“b**: ga¨eZ©x RvqMv ej‡Z ‡eov/†`qvj I ‡g‡Si g‡a¨ e¨eavb †evSv‡bv n‡q‡Q hv Avcbvi nv‡Zi me‡P‡q PIov ¯’v‡bi mgvb ev †ewk)

**--NOTE: A space is defined as any separation between the wall/partition and floor that is equal or greater to the widest part of your hand**

PviwU †`Iqvj I †g‡Si ga¨eZ©x RvqMv Av‡Q Space between all 4 walls and floor…… 01

wZbwU †`Iqvj I †g‡Si ga¨eZ©x RvqMv Av‡Q Space between 3 walls and floor………… 02

`yBwU †`Iqvj I †g‡Si ga¨eZ©x RvqMv Av‡Q Space between 2 walls and floor………… 03

GKwU †`Iqvj I †g‡Si ga¨eZ©x RvqMv Av‡Q Space between 1 wall and floor………… 04

‡Kvb †`Iqvj I †g‡Si ga¨eZ©x RvqMv bvB No space between any walls and floor… 05

**3.13** ch©‡e¶Y: Nygv‡bvi N‡ii cwigvc, ˆ`N¨©-cÖ¯’ cwigvc (K`g/ cv w`‡q ) msL¨vq wjLyb t

**Observation: Measurement of sleeping space, length by width (in steps)** **Record numbers below:**

***-- j¶¨ Ki“b****: me‡P‡q j¤^v ˆ`N©¨ Ges me‡P‡q PIov cÖ¯’ Mb¨ Kiæb|*

***--NOTE: Measure longest length and widest width present***

***j¶¨ Ki“b:AvswkK K`‡gi Rb¨ wbKU¯’ c~Y© wjLyb ( †hgb 5.5 Gi Rb¨ 6 wjLyb)***

***--NOTE: Round up to the nearest step (for example, record 5.5 steps as 06)***

 (‰`N©¨ length)  (cÖ¯’ width)

3.14 Bb‡W· †Km †ivMx hLb GLv‡b Nygvb, ZLb Zvi mv‡_ mvavibZ Avi K‡ZvRb †jvK GLv‡b GKBmg‡q Nygvb?

ASK: “When the index case patient sleeps in this space, how many other people typically sleep in this space at the same time?” **Record numbers below:**

**--j¶¨ Ki“b**: ইনডেক্স কেস রোগী যে রুমে ঘুমায় সেটি গন্য করুন। পুরো স্ট্রাকচার গন্য করুবেন না।

--NOTE: Look at the room where the index case sleeps, not the entire housing structure.



**End of Section Three**

**‡mKkb 4t nvZ‡avqvi ¯’v‡b mvev‡bi mnRcÖvc¨Zv (ch©‡e¶bK…Z)**

**Section 4: Availability of Soap at Handwashing Stations (observed)**

DËi`vZv‡K wRÁvmv Ki“b -ÒAvcwb †ekxi fvM mgq ‡h ¯’v‡b Avcbvi nvZ †avb `qv K‡i Avgv‡K †mB ¯’vbwU ‡`Lv‡eb wK?

**Ask the respondent: “Can you please show me where you most often wash your hands?”**

**4.1** ch©‡e¶Y: nvZ‡avqvi ¯’vbwU g~jZt †Kv_vq Aew¯’Z? (ev‡´ †KvW wjLyb ) 

**Observation: Record the location of the primary handwashing station: (Record code in box)**

***j¶¨ Ki“b-hw` Avcbv‡K nvZ †avqvi ¯’vbwU e¨env‡ii Rb¨ N‡ii KvVv‡gvi evB‡i †n‡U †h‡Z nq Zvn‡j nvZ †avqvi ¯’vbwU N‡ii evB‡i e‡j a‡i wbb|nvZ †avqvi ¯’vbwU GKwU Qv‡`i wb‡P n‡jI Zv Mb¨ bq| N‡ii evB‡i ej‡Z g~j KvVv‡gvi evB‡i †evSv‡bv n‡”Q|***

--NOTE: If you have to walk outside the housing structure to use the handwashing station, consider the handwashing station to be outdoors.

--NOTE: It does not matter whether the handwashing station is under a roof or not, outdoors means outside the main housing structure.

N‡ii wfZ‡i (cÖavb Kv‡Vv‡gvi wfZ‡i) /Indoors (within main housing structure) 01

N‡ii evB‡i-cÖavb Kv‡Vv‡gvi evB‡i Z‡e evoxi wfZ‡i/

Outdoors (outside main housing structure, within bari)

02

N‡ii evB‡i -evoxi evB‡i, cÖwZ‡ewki DVv‡b A_ev cyKz‡i/

Outdoors (outside bari, in neighbor’s yard or in pond)

03

†Kv‡bv wbw`©È ¯’v‡b bq/ No specific place 04

†`Lvi AbygwZ †bB/ No permission to see 05

Ab¨vb¨ (wbw`©ó Kiæb)/ Other (specify below) 88

_____________________________________________________________

🡪4.1 bs cÖ‡kœi DËi 3, 4 ev 5 n‡j 4.8 bs cÖ‡kœ P‡j hvb| (If the answer to question 4.1a is 3, 4 or 5, SKIP to question 4.8)

**4.2** ch©‡e¶Y: nvZ †avqvi Rb¨ wbw`©ó ¯’v‡b cvwb Av‡Q wK?

**Observation: Is water present at the specific place for handwashing?**

ev‡´ †KvW wjLyb (Record code in box) 

wb‡Ri †Pv‡L cvwb †`‡L ev‡´ n¨uv †KvW wjLyb

**--NOTE: You must actually see water to record “yes”**

*j¶¨ Ki“b cwigvb A_ev gvb †Kvb mgm¨v bq| hw` †Kvb cvwb _v‡K Zvn‡j n¨v †KvW Ki“b|*

**--NOTE: The quantity and quality does NOT matter. If any water is present, code “yes”**

Yes (n¨uv) 01

No (bv) 02

**4.3a** ch©‡e¶Y: nvZ †avqvi ¯’v‡b wb‡Pi †KvbwU Av‡Q? ( hw` Avcwb †KvbwU ch©‡e¶b K‡i _v‡Kb, Zvn‡j n¨uv =1, bv =2 ev‡· †KvW Ki“b)|

**Observation: Which of the following are present at the handwashing station? (If you observe the listed item, write “1” for “yes” in the box below. If you do not observe the listed item, write “2” for “no” in the box below.)**

*j¶¨ Ki“b nvZ †avqvi ¯’v‡bi Pvi cv‡ki gvwU / evjyi Rb¨ gvwU / evjy †KvW Ki‡eb bv| hw` GKwU wbw`©ó cv‡Î ivLv _v‡K Ges nvZ †avqvi ¯’v‡b e¨eüZ nq Zvn‡j †KvW Ki“b*

**--NOTE: Do not record “mud/sand” if there is mud on the ground around the handwashing station. Record it if it is kept in a specific place or container and if there is evidence that it is kept intentionally to be used at the handwashing station for any purpose.**

Yes (n¨uv) 01

No (bv) 02

mvevb (Bar soap) 

cvDWvi/wWUvi‡R›U (Detergent powder) 

Zij mvevb (Liquid soap) 

QvB (Ash) 

gvwU/evjy (Mud/Sand) 

wKQzB †bB (None) 

Ab¨vb¨, wbw`©ó Kiæb (Other, specify below) 

____________________________________________

**4.3b ch©‡e¶Y**: g~j nvZ †avqvi ¯’vb †_‡K ivbœv Kivi ¯’v‡bi `yiæZ¡ (K`g w`‡q cwigvc Kiæb Ges wjLyb)

**MEASURE: Distance from primary handwashing station to cooking area (in steps, measure to stove in cooking area)**

ev‡´ †KvW wjLyb (Record code in box) 

**4.3c** ch©‡e¶Y: g~j nvZ †avqvi ¯’vb †_‡K j¨vwUª‡bi ¯’v‡bi `yiæZ¡ (K`g w`‡q cwigvc Kiæb Ges wjLyb) †Kv‡bv j¨vwUªb bv _vK‡j wUK w`b|

**MEASURE: Distance from primary handwashing station to latrine (in steps, measure to entrance of latrine) (Record number below)**

**If there is NO LATRINE, code “99”**

ev‡´ †KvW wjLyb (Record code in box) 

**4.4** DËi`vZv‡K: wRÁvmv Ki“bt ÒGQvov Avi †Kv_vqI Avcwb nvZ †avb wK?

**Ask the respondent: “Is there anywhere else you wash your hands?”**

ev‡´ †KvW wjLyb (Record code in box) 

Yes (n¨uv) 01

No (bv) 02

- hw` 4.4 Gi DËi 2 nqZ‡ e cÖkœ 4.8 G hvb (If answer to 4.4 is 2, skip to question 4.8)

**4.5** ch©‡e¶Y: nvZ †avqvi wØZxq ¯’vbwU †Kv_vq Aew¯’Z? (ev‡´ †KvW wjLyb )

**Observation: Record the location of the secondary handwashing station:**

ev‡´ †KvW wjLyb (Record code in box) 

N‡ii wfZ‡i /Indoors (within main housing structure) 01

N‡ii evB‡i-cÖavb Kv‡Vv‡gvi evB‡i Z‡e evoxi wfZ‡i/

Outdoors (outside main housing structure, within bari) 02

N‡ii evB‡i -evoxi evB‡i, cÖwZ‡ewki DVv‡b A_ev cyKz‡i/

Outdoors (outside bari, in neighbor’s yard or in pond) 03

†Kv‡bv wbw`©È ¯’v‡b bq/ No specific place 04

†`Lvi AbygwZ †bB/ No permission to see 05

Ab¨vb¨ (wbw`©ó Kiæb)/ Other (specify below) 88

_____________________________________________________________

🡪 hw` 4.5 bs cÖ‡kœi DËi 3, 4 ev 9 nq, cÖkœ 4.8 G hvb (If the answer to question 4.5a is 3, 4 or 9, SKIP to question 4.8 )

**4.6** ch©‡e¶Y: nvZ †avqvi Rb¨ wbw`©ó ¯’v‡b cvwb Av‡Q wK? (wb‡Ri †Pv‡L cvwb †`‡L ev‡´ n¨uv †KvW wjLyb )

**Observation: Record if water is present at the specific place for handwashing? (You must actually see water to record “yes”):**

ev‡´ †KvW wjLyb (Record code in box) 

Yes (n¨uv) 01

No (bv) 02

**4.7a ch©‡e¶Y:** nvZ †avqvi ¯’v‡b wb‡Pi †KvbwU Av‡Q? (hw` Avcwb †KvbwU ch©‡e¶b K‡i _v‡Kb, Zvn‡j n¨uv =1 , bv =2 ev‡· †KvW Ki“b)| **Observation: Which of the following are present at the handwashing station? (If you observe the listed item, write “1” for “yes” in the box below. If you do not observe the listed item, write “2” for “no” in the box below.)**

*j¶¨ Ki“b nvZ †avqvi ¯’v‡bi Pvi cv‡ki gvwU / evjyi Rb¨ gvwU / evjy †KvW Ki‡eb bv| hw` GKwU wbw`©ó cv‡Î ivLv _v‡K Ges nvZ †avqvi ¯’v‡b e¨eüZ nq Zvn‡j †KvW Ki“b*

**--NOTE: Do not record “mud/sand” if there is mud on the ground around the handwashing station. Record it if it is kept in a specific place or container and if there is evidence that it is kept intentionally to be used at the handwashing station for any purpose.**

Yes (n¨uv) 01

No (bv) 02

mvevb (Bar soap) 

cvDWvi/wWUvi‡R›U (Detergent powder) 

Zij mvevb (Liquid soap) 

QvB (Ash) 

gvwU/evjy (Mud/Sand) 

wKQzB †bB (None) 

Ab¨vb¨, wbw`©ó Kiæb (Other, specify below) 

___________________

**4.7b ch©‡e¶Y**: †m‡KÛvix nvZ †avqvi ¯’vb †_‡K ivbœv Kivi ¯’v‡bi `yiæZ¡ (K`g w`‡q cwigvc Kiæb Ges wjLyb)

**Observation: Distance from secondary handwashing station to cooking area (in steps, measure to stove in cooking area) Record number below:**



**4.7c ch©‡e¶Y**: †m‡KÛvix nvZ †avqvi ¯’vb †_‡K j¨vwUª‡bi ¯’v‡bi `yiæZ¡ (K`g w`‡q cwigvc Kiæb Ges wjLyb) †Kv‡bv j¨vwUªb bv _vK‡j wUK w`b|

**Observation: Distance from secondary handwashing station to latrine (in steps, measure to entrance of latrine) Record number below:**

*j¨vwUªb bv _vK‡j* **99** *†KvW© Ki“b*

**If there is NO LATRINE, code “99”**



**4.8** DËi`vZv‡K wRÁvmv Ki“b - H¯’vb Qvov N‡ii wfZ‡i wb‡Pi †Kvb Dcv`vbwU i‡q‡Q? (DËi`vZv‡K †mUv Avcbv‡K †`Lv‡Z ejyb) ? (hw` Avcwb †KvbwU ch©‡e¶b K‡i _v‡Kb, Zvn‡j n¨uv =1 , bv =2 ev‡· †KvW Ki“b)|

**Ask the respondent: Which of the following are present in the household regardless of place? (If you observe the listed item, write “1” for “yes” in the box below. If you do not observe the listed item, write “2” for “no” in the box below.)**

*DËi`vZv‡K ‡`Lv‡bvi Rb¨ ej‡Z n‡e|*

**You should ask the respondent to show you.**

Yes (n¨uv) 01

No (bv) 02

mvevb (Bar soap) 

cvDWvi/wWUvi‡R›U (Detergent powder) 

Zij mvevb (Liquid soap) 

QvB (Ash) 

gvwU/evjy (Mud/Sand) 

wKQzB †bB (None) 

Ab¨vb¨, wbw`©ó Kiæb (Other, specify below) 

__________________________________________

**End of Section Four**

**‡mKkb 5t M„n¯’vjx‡Z aygcvb msµvšÍ cÖkœmg~n**

**Section 5: Housing structure Smoking Status Questions**

**5.1** DËi`vZv‡K wRÁvmv Ki“b - Avcbvi N‡ii KvVv‡gv‡Z aygcv‡bi †¶‡Î wb‡Pi †Kvb wbqgwU †ekx cÖ‡hvR¨?

**ASK, Which of the following best describes the rules about smoking inside your housing structure?**

ev‡´ †KvW wjLyb (Record number in box): 

me¸‡jv Ackb D”P¯^‡i c‡o †kvbvb

(Read all options out loud)

Avcbvi N‡ii KvVv‡gvi g‡a¨ aygcvb Kivi AbygwZ Av‡Q

Smoking is allowed in your housing structure 01

Avcbvi N‡ii KvVv‡gvi g‡a¨ mvavibZ aygcvb Kivi AbygwZ ‡bB, Z‡e gv‡S gv‡S e¨wZµg N‡U

Smoking is generally not allowed inside of your housing structure but there are exceptions 02

Avcbvi N‡ii KvVv‡gvi g‡a¨ KL‡bvB a~gcv‡bi AbygwZ ‡bB

Smoking is never allowed inside your housing structure 03

aygcv‡bi R‡b¨ Avcbvi N‡i aivevav †Kvb wbqg †bB

There are no rules about smoking in your housing structure 04

Rvwb bv Don't know 99

- hw` 5.1 Gi DËi 3 nqZ ত‡e 6 bs †mKk‡b P‡j hvb If answer to 5.1 is 3 then skip to section 6

**5.2** DËi`vZv‡K wRÁvmv Ki“b - Avcbvi N‡ii KvVv‡gvi wfZ‡i mKj i“‡gB wK aygcvb MÖnY‡hvM¨?

**ASK,**  **Inside your housing structur is smoking allowed in every room?**

***--j¶¨ Ki“b:*** *hw` ïaygvÎ GKwU wbw`©ó i“‡g GKRb gvÎ †jv‡KiI a~gcv‡bi AbygwZ _v‡K (†hgb wcZv), wKš‘ Ab¨ †KD (†hgb mšÍv‡biv) †mLv‡b a~gcvb bv K‡i, Zvic‡iI †mB i“‡g a~gcv‡bi AbygwZ i‡q‡Q e‡j Mb¨ Ki‡Z n‡e|*

***--NOTE:*** *If any person is allowed to smoke inside a particular room for one person (for example, a father), even if other people (for example, the kids) are not allowed to smoke inside that same room, then smoking is allowed in that room.*

ev‡´ †KvW wjLyb (Record code in box) 

Yes (n¨uv) 01

No (bv) 02

Don’t know/Refused to answer (Rvwb bv/ DËi †`‡eb bv) 99

**5.3** DËi`vZv‡K wRÁvmv Ki“b KZ mgq ci ci Avcbvi N‡ii KvVv‡gvi g‡a¨ †KD aygcvb K‡i?

**ASK, How often does anyone smoke inside your housing structure?**

ev‡´ †KvW wjLyb (Record code in box) 

‰`wbK Daily 01

mvßvwnK Weekly 02

gvwmK Monthly 03

gvwm‡Ki †P‡q Kg mgq Less than monthly 04

KL‡bvB bv Never 05

bv Rvwb bv Don’t Know 99

**End of Section Five**

**‡mKkb 6: Av_©-mvgvwRK Ae¯’v**

**Section 6: Socioeconomic Status**

**6.1** DËi`vZv‡K wRÁvmv Ki“b - Avcbvi খানার (A_ev খানার †Kvb m`‡m¨i) Av‡Q wK? (cÖ‡Z¨KwU †¶‡Î hw` Lvbvi wb‡Pi †h †Kv‡bv AvB‡Ug _v‡K, Z‡e n¨uv =1 , bv =2 ev‡· wjLyb| hw` DËi`vZvi bv Rvbv _v‡K Z‡e ev‡· Ó9”†KvW Ki“b)|

**ASK, Does your household (or any member of your household from the Enumeration Form) have:** *(If the household has the listed item, write “1” for “yes” in the box below. If the household does not have the listed item, write “2” for “no” in the box below, if the respondent does not know, write “9” for “Don’t know” in the box below.)*

*j¶¨ Ki“b-ïaygvÎ †mB Dcv`vb¸wj AšÍf©y³ Ki“b hv evox‡Z Dcw¯’Z Av‡Q, XvKv wKsev we‡`‡k bq*

--***NOTE:*** *Include items that are present in bari, not items owned in Dhaka or abroad.*

Yes (n¨uv) 01

No (bv) 02

Don’t know/Refused to answer (Rvwb bv/ DËi †`‡eb bv) 99

- 1. we`yr (Electricity)? 
  2. Avjgvix (Almirah or wardrobe)? 
  3. †Uwej (A table)? 
  4. ‡Pqvi ev †eÂ (A chair or bench)? 
  5. Nwo (A watch or clock)? 
  6. LvU (*Khat)?* 
  7. †PŠwK *(Chouki)?* 
  8. mPj †iwWI (A radio that is working)? 
  9. mPj †UwjwflY (mv`v-Kv‡jv) (A television (B/W) that is working)? 
  10. mPj ‡UwjwflY (iwOb) (A television (Color) that is working)? 
  11. wd«R (A refrigerator)? 
  12. ev&BmvB‡Kj (A bicycle)? 
  13. gUi mvB‡Kj (A motorcycle)? 
  14. †mjvB‡gwkb (A sewing machine)? 
  15. j¨vÛ‡dvb (A land phone)? 
  16. †gvevBj †dvb (A mobile phone)? 

**6.2** ch©‡e¶Y: DËi`vZvi nvDwRs ÷ªvKPv‡ii Qv` ˆZix‡Z wK Dcv`vb e¨envi Kiv n‡q‡Q ?

**Observation: Material of the Roof of respondent’s housing structure:**

***--j¶¨ Ki“b****:* ***সবচেয়ে দামী উপাদান লিপিবদ্ধ করুন।***

***--NOTE: Please record the COSTLIEST material present***

ev‡´ †KvW wjLyb (Record code in box) 

mvavib Qv`Natural roof

KvuPv (evuk/Lo) Katcha (bamboo / thatch) 11

‡gŠwjK Qv` Rudimentary roof

wUb Tin 21

m¤cbœ Qv` (cvKv) Finished roof (pukka)

wm‡g›U/ KswµU/ Uvwj Cement / concrete / tiled 31

(Ab¨vb¨t wb‡P wbw`©ó K‡i wjLyb) Other: Specify below 41

_____________________________________

**6.3** ch©‡e¶Y: DËi`vZv nvDwRs ÷ªvKPv‡ii †`qvj ˆZix‡Z c wK Dcv`vb e¨envi Kiv n‡qQ? (ch©‡e¶bK…Z Z_¨ wjwce× Ki“b)

**Observation: Material of the walls of the respondent’s housing structure**

***--j¶¨ Ki“b: সবচেয়ে দামী উপাদান লিপিবদ্ধ করুন।***

***--NOTE: Please record the COSTLIEST material present***

ev‡´ †KvW wjLyb (Record code in box) 

mvavib ‡`qvj Natural walls

cvU/evuk/কাদামাটি (KvuPv)

Jute / bamboo / mud (*katcha*) 11

‡gŠwjK ‡`qvj Rudimentary walls

KvV Wood 21

m¤úbœ ‡`qvj (cvKv) Finished walls

BU/wm‡g›U Brick / cement 31

wUb Tin 32

(Ab¨vb¨t wb‡P wbw`©ó K‡i wjLyb) Other: Specify below 41

__________________________________________________

**6.4** ch©‡e¶Y: DËi`vZv nvDwRs ÷ªvKPv‡ii ‡g‡S ˆZix‡Z wK Dcv`vb e¨envi Kiv n‡qQ ?

**Observation: Main material of the floor of the respondent’s housing structure**

***--j¶¨ Ki“b: সবচেয়ে দামী উপাদান লিপিবদ্ধ করুন।***

***--NOTE: Please record the COSTLIEST material present***

ev‡´ †KvW wjLyb (Record code in box) 

mvavib ‡g‡S Natural floor

gvwU/evuk(KvuPv)Earth / bamboo (katcha) 11

‡gŠwjK ‡g‡S Rudimentary floor

KvV Wood 21

m¤cbœ ‡g‡S (cvKv)Finished floor (pukka)

wm‡g›U/ KswK&ªU Cement / concrete 31

(Ab¨vb¨t wb‡P wbw`©ó K‡i wjLyb)Other: Specify below 41

_________________________________________________

**6.5** DËi`vZv‡K wRÁvmv Ki“b - †MvmjLvbv I ivbœvi ¯’vb ev‡` Avcbvi Ggb KZwU i“g Av‡Q hv N‡ii KvVv‡gvi AšÍf~©³?

**ASK, Excluding the bathroom and cooking area, how many rooms are there in your housing structure?**

***--লক্ষ্য করুন:*** *ঘর কি কাজে ব্যবহার হয়, তা জিজ্ঞেস করবেন না। তারা আপনাকে যতোটি রুমের কথা বলে, লিখুন। উত্তরদাতাকে কেবল একটা সংখ্যা জানাতে বলুন।*

***--NOTE:*** *Do not ask what the rooms are used for. Record the number that they give you. Ask them to give you a number.*

***-লক্ষ্য করুন:*** *খানার লোকেরা বসবাস করে, শুধুমাত্র ঘরের এমন রুমগুলোকে রুম হিসেবে গন্য করুন। স্টোর রুম বা অন্যান্য রুম যদি ঘরের অন্তর্ভুক্ত হয়, তবে তা গন্য হবে।*

***--NOTE:*** *Only count rooms that are within the housing structures where household members live. Animal houses are not included. Store rooms or other rooms, that are within the housing structure, are included.*

***-লক্ষ্য করুন:*** *যদি কমপক্ষে অর্ধেক উচ্চতার দেয়াল হয়, তবে দুটি আলাদা রুম গন্য করুন। নয়তো একটা রুম হিসেবে গন্য হবে।*

*--****NOTE:*** *Count two rooms as separate if the partition that divides them covers >50% of the length of the wall. Otherwise consider as one room.*

ev‡´ †KvW wjLyb (Record code in box) 

**6.6** DËi`vZv‡K wRÁvmv Ki“b - Avcbvi N‡i (হাউজিং স্ট্রাকচারে)Nygv‡bvi Rb¨ KZwU iæg Av‡Q?

**ASK, How many rooms does your housing structure have for sleeping?**

***-লক্ষ্য করুন:*** *৫০% এর বেশি দিন ধরে যদি কোনো রুমে ঘুমানো হয়, তবে তাকে “ঘুমানোর রুম” হিসেবে গন্য করুন।*

***--NOTE****: Count a room as “for sleeping” if it is slept in >50% of days*

***-লক্ষ্য করুন:*** *jÿ¨ Kiæb: hLb Avcwb †`qv‡ji mvg‡b `uvwo‡qI `yB iæ‡gi ga¨eZx© cvwU©k‡bi Dci w`‡q ‡`L‡Z cvi‡eb bv, ZLbB `ywU iæg wn‡m‡e Mb¨ Ki‡eb|*

***--NOTE:*** *Count rooms as separate when you cannot see over the partition separating the two rooms while standing in front of partition*

ev‡´ †KvW wjLyb (Record code in box) 

**6.7** DËi`vZv‡K wRÁvmv Ki“b - Avcwb †Kvb K¬vk ch©šÍ †jLv-cov K‡i‡Qb? (msL¨vq wjLyb) (hw` DËi`vZvi bv Rvbv _v‡K Z‡e ev‡· Ó99”†KvW Ki“b)|

**Ask the respondent: How many years of education have you completed? (record number of years completed below. If “don’t know”, record 99.)**

*-লক্ষ্য করুন: যদি উত্তরদাতা স্কুলে পড়ে, তবে যেই ক্লাসে পড়ছে সেই সংখ্যাটি লিখুন।উদাহরণস্বরুপ, ক্লাস ০৬ এ পড়লে ৬ লিখুন। এইচএসসি-র জন্যে ১২, ব্যাচেলর ডিগ্রির জন্যে ১৪ এবং মাস্টার্স-এর জন্যে ১৬ লিখুন।*

*-NOTE: If a respondent is in school, record the number corresponding to their current class number. (For example, for someone in class six, reply “06”. For HSS graduates, record “12”; for Bachelor’s graduates record “14”; for Master’s level record “16”.*

ev‡´ †KvW wjLyb (Record code in box) 

**6.8** DËi`vZv‡K wRÁvmv Ki“b - Avcwb wK **Lvbvi** cÖavb?

**Ask the respondent: Are you the head of household?**

ev‡´ †KvW wjLyb (Record code in box) 

Yes (n¨uv) 01

No (bv) 02

Don’t know/Refused to answer (Rvwb bv/ DËi †`‡eb bv) 99

🡪6.8 Gi DËi hw` 1 nq, Z‡e cÖkœ bs 6.10 †Z P‡j hvb (If answer to 6.8 is 1, skip to question 6.10)

**6.9** DËi`vZv‡K wRÁvmv Ki“b - **Lvbv** cÖavb †Kvb K¬vk ch©šÍ †jLv-cov m¤úbœ K‡i‡Qb? (wb‡P msL¨vq wjLyb) hw` DËi`vZvi bv Rvbv _v‡K Z‡e ev‡· Ó99”†KvW Ki“b)|

**Ask the respondent: How many years of education has the head of household completed? (record number below. If “don’t know”, record 99.)**

ev‡´ †KvW wjLyb (Record code in box) 

**6.10** DËi`vZv‡K wRÁvmv Ki“b - Avcbvi **Lvbv**i wbR¯^ **emZwfUv** Av‡Q wK?

**Ask the respondent: Does your household own homestead land?**

ev‡´ †KvW wjLyb (Record code in box) 

Yes (n¨uv) 01

No (bv) 02

Don’t know/Refused to answer (Rvwb bv/ DËi †`‡eb bv) 99

🡪6.10 Gi DËi hw` 2 ev 99 nq, Z‡e cÖkœ bs 6.12 †Z P‡j hvb (If answer to 6.10 is 2 or 99, skip to question 6.12)

**6.11** DËi`vZv‡K wRÁvmv Ki“b - Avcbvi Lvbvর †gvU KZUzKz emZভিটা (†Wwm‡gj) Av‡Q? (Rvwb bv n‡j **“9999”** †KvW Ki“b)

**Ask the respondent: How much homestead land does your household own? If “don’t know” code “9999”.**

Rwgi †gvU ¯’vbxq GKK Total local land unitt

AMOUNT cwigvb: __________________ SPECIFY UNIT GKK wbw`©ó K‡i wjLyb: _______________

স্থানীয় একক থেকে ডেসিমেল (বিসটিস অফিসে পূরণ করুন)

Conversion from Local Land Unit to Decimals (complete in BISTIS office)

স্থানান্তর রেট: Conversion Rate:

মোট জমির পরিমাণ ডেসিমেলে Total Land in Decimalst

AMOUNT cwigvb: __________________ decimals

**6.12** DËi`vZv‡K wRÁvmv Ki“b - emZভিটা Qvov Avcbvi Lvbvর Avi †Kvb Rwg Av‡Q wK?

**Ask the respondent: Does your household own any land, other than homestead land?**

ev‡´ †KvW wjLyb (Record code in box) 

Yes (n¨uv) 01

No (bv) 02

Don’t know/Refused to answer (Rvwb bv/ DËi †`‡eb bv) 99

🡪 6.12 Gi DËi hw` 2 ev 9 nq, Z‡e cÖkœ bs 6.14 †Z P‡j hvb (If answer to 6.12 is 2 or 9, skip to question 6.14)

**6.13** DËi`vZv‡K wRÁvmv Ki“b - emZwfUv Qvov Avcbvi Lvbvর wK cwigvb Ab¨vb¨ Rwg (†Wwm‡gj) Av‡Q**?** (Rvwb bv n‡j **“9999”** †KvW Ki“b)

**Ask the respondent: How much land, other than homestead land, does your household own? If “don’t know” code “9999”.**

Rwgi †gvU ¯’vbxq GKK Total local land unitt

AMOUNT cwigvb: __________________ SPECIFY UNIT GKK wbw`©ó K‡i wjLyb: _______________

স্থানীয় একক থেকে ডেসিমেল (বিসটিস অফিসে পূরণ করুন)

Conversion from Local Land Unit to Decimals (complete in BISTIS office)

স্থানান্তর রেট: Conversion Rate:

মোট জমির পরিমাণ ডেসিমেলে Total Land in Decimalst

AMOUNT cwigvb: __________________ decimals

**6.14** DËi`vZv‡K wRÁvmv Ki“b - Avcbvi Lvbvq ivbœvi Rb¨ cÖavbZ wK ai‡bi R¡vjvbx e¨envi Kiv nq?

**Ask the respondent: What type of fuel does your household mainly use for cooking?**

ev‡´ †KvW wjLyb (Record code in box) 

KvV Wood 01

k‡m¨i Aewkóvsk/Nvm/শুকনো পাতা/পাটের পাতা

Crop residue / grass/dried leaves/jute leaves 02

ïKbv †Mvei Dung cakes 03

Kqjv/KK/wjMbvBU Coal / coke / lignite 04

KvV Kqjv Charcoal 05

‡K‡ivwmb Kerosene 06

we`y¨r Electricity 07

Zij M¨vm/cÖvK…wZK M¨vm Liquid gas / gas 08

ev‡qv-M¨vm Bio-gas 09

Ab¨vb¨, wb‡P wbw`©ó K‡i wjLyb Other, specify below 88

________________________________________________________

Rvwb bv Don’t know 99

**6.15** DËi`vZv‡K wRÁvmv Ki“b - Avcbvi Lvbvর Lvevi Rb¨ cvwbi cÖavb Drm Kx?

**Ask the respondent: What is the main source of water your household uses for drinking?**

ev‡´ †KvW wjLyb (Record code in box) 

AMfxi wUDeI‡qj Shallow tube well. 01

Mfxi wUDeI‡qj Deep tube well. 02

myiw¶Z cvZK~qv Protected ring/dug well 03

Amyiw¶Z cvZK~qv Unprotected dug well ………………. 04

Zviv cv¤ú Tara pump 05

Av‡m©wbK †kvabvMvi Arsenic free treatment plant 06

myiw¶Z Sbv©i cvwb Water from protected spring … 07

Amyiw¶Z Sbv©i cvwb Water from unprotected spring … 08

f~c„‡ôi cvwbt Surface water

e„wói cvwb Rainwater ……………………………….. 09

U¨vsKvi UªvK Tanker truck ………………………….. 10

†QvU U¨vsKhy³ KvU Cart with small tank ………………… 11

Rxevbygy³Kib cvwbt Pathogen treatment plant (Pond Sand Filter) 12

mivmwi msM„nxZ cvwbt Directly from:

b`x/eva/†jK/cyKzi/†mP bvjv ‡_‡K River/dam/lake/ponds/stream/canal/irrigation channel 13

‡evZjRvZ cvwb Bottled water …………………..…….. 14

N‡ii wfZi U¨vc ev cvB‡ci cvwb Piped water into housing structure ………… 15

DVv‡b U¨vc ev cvB‡ci cvwb Piped water into yard/plot … … 16

cvewjK U¨vc Public tap/stand pipe …………………. 17

Ab¨vb¨, wb‡P wbw`©ó K‡i wjLyb Other: specify below 88

_______________________________________________________________________________________

**6.16** AbyMÖnK‡i Avgv‡K †`Lv‡ib wK Avcbviv †Kv_vq cvqLvbv K‡ib ? ch©‡e¶Y: cvqLvbvi aib †iKW© Ki“b|

**Say: “Please show me the place where you go to defecate.” Observation: Record type of toilet facility**

ev‡´ †KvW wjLyb (Record code in box) 

**DbœZgv‡bi Uq‡jU/ cvqLvbv** Improved sanitation facilities**:**

d¬vk Uq‡jU A_ev cvwb †X‡j d¬vk Kiv Uq‡jU hv cqwb¯‹vkb cvB‡ci mv‡_ ms‡hvM K‡i †`qv

Flush / pour flush to piped sewer system 01

d¬vk Uq‡jU A_ev cvwb †X‡j †mcwUK U¨vs†K hvevi e¨e¯’¨v Av‡Q

Flush / pour flush to septic tank 02

d¬vk Uq‡jU A_ev cvwb †X‡j cvqLvbv wc‡Ui g‡a¨ mwi‡q †`qv hvq Flush / pour flush to pit latrine 03

¯­ve mn wcU Uq‡jU /j¨vwUªb Pit latrine with slab 04

**AbDbœZgv‡bi Uq‡jU/ cvqLvbv** *Unimproved sanitation facilities***:**

d¬vk Uq‡jU A_ev cvwb †X‡j d¬vk Kiv Uq‡jU hv cqwb¯‹vkb cvBc, †mcwUK U¨vsK ev j¨vwUª‡bi mv‡_ ms‡hvRb ‡bB| ( Uq‡jU hv †Kvb Lvj,†Wªb,b`x BZ¨vw`imv‡_ mshy³)

Flush or pour flush toilet not to sewer system, septic tank, or pit latrine (e.g., to canal, ditch,

river, etc.).. 05

wcU j¨vwUªb /Uq‡jU hvi ¯­ve †bB(†Lvjv wcU) Pit latrine without slab (i.e., open pit).. 06

SzwoÍ /cvÎ ivLv Bucket 07

SzjšÍ Uq‡jU / cvqLvbv Hanging toilet/latrine… 08

**†Lvjv RvqMvq cvqLvbv** *Open defecation:*

‡Kvb cvqLvbv †bB/R½‡j/†Sv‡cSv‡o/gvV No facility/bush/field ………………………………… 09

Ab¨vb¨:( D‡j­L Ki“b) others (Specify below) 88

__________________________________________________________________________________

**6.17** DËi`vZv‡K wRÁvmv Ki“b - Avcbvi Lvbv e¨vZxZ Avi KZ¸‡jv Lvbv wg‡j GB cvqLvbvwU e¨envi K‡ib ?

**Ask the respondent: How many households, other than your own, use this toilet facility?**

ev‡´ †KvW wjLyb (Record code in box) 

**6.18** wRÁvmv Ki“b Avcbvi Lvbvi Lv`¨vfvm / Lv`¨ MÖn‡bi aiY Abyhvqx wKfv‡e Avcwb Avcbvi Lvbvi †kªYx weY¨vm Ki‡eb? mviv eQiB wK Lv`¨ Afve _v‡K? KL‡bv KL‡bv Lv`¨ Afve _v‡K, Lv`¨ AfveI _v‡K bv DØ„Ë _v‡K bv,Lv`¨ DØ„Ë _v‡K?

**ASK, “In terms of household food consumption, how do you classify your household: deficit the whole year, sometimes deficit, neither deficit nor surplus, surplus?**

ev‡´ †KvW wjLyb (Record code in box) 

mviv eQiB Lv`¨ Afve _v‡K (Deficit the whole year) 01

KL‡bv KL‡bv Lv`¨ Afve _v‡K (Sometimes deficit) 02

Lv`¨ AfveI _v‡K bv DØ„Ë _v‡K bv (Neither deficit nor surplus) 03

Lv`¨ DØ„Ë _v‡K (Surplus) 04

DËi`vZv cªkœ eyS‡Z cv‡I bv Ges †Kvb dËi †`q bv

(Respondent does not understand question and has no response) 33

Rvwb bv/ wbwðZ bB (Don’t know/not sure) 99

***ধন্যবাদ। এই অংশটুকু শেষ হয়েছে। Thank you. This part is finished.***

**এফআরএ কোড এবং স্বাক্ষর** Code and signature of FRA:

**Ending Time (24-hour format): __ __:__ __**

| Checked by: | FRA/FRO/MO Code | Signature | Date (dd/mm) |
| --- | --- | --- | --- |
| FRA |  |  |  |
| FRO |  |  |  |
| MO |  |  |  |

**Appendix 10: Illness Tracking Form (for all ages)** Version 18.5.10

Name of Contact: ___________________________________ Is this the Index-Case? (Yes (1) or No (2)): ____

Respondents (by age of contact)

≤7 🡪 guardian

7-17 🡪 self, verified by guardian

18+ 🡪 self

Not Present 🡪 Head Mother/HOH

Contact Unique ID___ ___ ___ ___ ___ ___ ___ ___ ___ ___ Age(years)at enrollment: **__________________**

Date of Call to FRO for Specimen Collection (dd/mm/yy) ___ ___/ ___ ___/ ___ ___ Time (24-hour format) ___ ___: ___ ___

First Day of Illness Tracking (dd/mm/yy): ___ ___/ ___ ___/ ___ ___ Last Day of Illness Tracking (dd/mm/yy): ___ ___/___ ___/___ ___

(ICP ONLY): Date of Second Fever-Free Day (dd/mm/yy): ___ ___/___ ___/___ ___

**Mark 1 for “yes”, 2 for “no”, 99 for “don’t know”, for SKIP, “55” for “No household member present”, and for Danger Signs Sections only: “0”for observed danger sign and “3” for reported danger sign**

| **Day of Observation** | | **11** | **2** | **13** | **14** | **15** | **16** | **17** | **18** | **19** | **110** | **211** | **212** | **113** | **114** | **115** | **16** | **17** | **18** | **19** | **20** |
| --- | --- | --- | --- | --- | --- | --- | --- | --- | --- | --- | --- | --- | --- | --- | --- | --- | --- | --- | --- | --- | --- |
| **Present in Bari during past 24 hours?** (If “2”, “55” or “99” **SKIP** column to “FRA code” and “DATE”) | |  |  |  |  |  |  |  |  |  |  |  |  |  |  |  |  |  |  |  |  |
| **Contact present at time of illness tracking**  (If “2”, collect symptom information from head mother/household head) | |  |  |  |  |  |  |  |  |  |  |  |  |  |  |  |  |  |  |  |  |
| **Eligibility Symptoms & Specimen Collection ASK contact, “In the past 24 hours have you had fever?”** | | | | | | | | | | | | | | | | | | | | | |
| Fever (If “no”, **SKIP** to “Other Symptoms”) | |  |  |  |  |  |  |  |  |  |  |  |  |  |  |  |  |  |  |  |  |
| If “yes” specimen collection needed—**call to FRO***  Record date and time of call above. | |  |  |  |  |  |  |  |  |  |  |  |  |  |  |  |  |  |  |  |  |
| **Other Symptoms ASK contact, “In the past 24 hours, have you had _________?”** | | | | | | | | | | | | | | | | | | | | | |
| Cough | |  |  |  |  |  |  |  |  |  |  |  |  |  |  |  |  |  |  |  |  |
| Sore Throat | |  |  |  |  |  |  |  |  |  |  |  |  |  |  |  |  |  |  |  |  |
| Watery Diarrhea (≥ 3 loose stools within 24 hours) | |  |  |  |  |  |  |  |  |  |  |  |  |  |  |  |  |  |  |  |  |
| Bloody Diarrhea (watery diarrhea (≥ 3) with blood in it) | |  |  |  |  |  |  |  |  |  |  |  |  |  |  |  |  |  |  |  |  |
| Injury | |  |  |  |  |  |  |  |  |  |  |  |  |  |  |  |  |  |  |  |  |
| **Danger Signs* for AGES ≥ 5**  **SKIP** if Contact is < 5. Enter “0” if danger signs (DSs) are directly observed, “3” if DSs are only reported, “2”, “99” or when applicable  **Refer to JIMCH (unless patient already sought care and Danger Sign is no longer present) & Call FRO*** | | | | | | | | | | | | | | | | | | | | | |
| Cyanosis | |  |  |  |  |  |  |  |  |  |  |  |  |  |  |  |  |  |  |  |  |
| Severe respiratory distress | |  |  |  |  |  |  |  |  |  |  |  |  |  |  |  |  |  |  |  |  |
| Convulsions | |  |  |  |  |  |  |  |  |  |  |  |  |  |  |  |  |  |  |  |  |
| Altered mental status | |  |  |  |  |  |  |  |  |  |  |  |  |  |  |  |  |  |  |  |  |
| **Danger Signs* for AGES < 5**  **SKIP** if Contact is ≥ 5. Enter “0” if danger signs (DSs) are directly observed, “3” if DSs are only reported, “2”, “99” or when applicable  **Refer to JIMCH (unless patient already sought care and Danger Sign is no longer present) & Call FRO*** | | | | | | | | | | | | | | | | | | | | | |
| Chest In-drawing | |  |  |  |  |  |  |  |  |  |  |  |  |  |  |  |  |  |  |  |  |
| Lethargy | |  |  |  |  |  |  |  |  |  |  |  |  |  |  |  |  |  |  |  |  |
| Cyanosis | |  |  |  |  |  |  |  |  |  |  |  |  |  |  |  |  |  |  |  |  |
| Inability to drink | |  |  |  |  |  |  |  |  |  |  |  |  |  |  |  |  |  |  |  |  |
| Convulsions | |  |  |  |  |  |  |  |  |  |  |  |  |  |  |  |  |  |  |  |  |
| Fever for ages < 2 months | |  |  |  |  |  |  |  |  |  |  |  |  |  |  |  |  |  |  |  |  |
| **Danger Sign Actions SKIP** If no danger signs observed or reported on this day of illness tracking. | | | | | | | | | | | | | | | | | | | | | |
| Danger Sign Present 🡪 Called to FRO | Patient referred & went to hospital w/ FRA |  |  |  |  |  |  |  |  |  |  |  |  |  |  |  |  |  |  |  |  |
|  | Patient referred & will go to hospital w/o FRA |  |  |  |  |  |  |  |  |  |  |  |  |  |  |  |  |  |  |  |  |
|  | Patient referred & refused to go to hospital |  |  |  |  |  |  |  |  |  |  |  |  |  |  |  |  |  |  |  |  |
|  | Patient visited hospital before FRA visit (referred if symptoms remain) |  |  |  |  |  |  |  |  |  |  |  |  |  |  |  |  |  |  |  |  |
| **Outcomes SKIP** if none of the above symptoms (FEVER or “Other Symptoms”) are reported on this day of illness tracking.  **ASK**, “Did you miss school/work, take medication or seek medical care as a result of the above symptoms you identified?” | | | | | | | | | | | | | | | | | | | | | |
| Missed work/school | |  |  |  |  |  |  |  |  |  |  |  |  |  |  |  |  |  |  |  |  |
| Took medication | |  |  |  |  |  |  |  |  |  |  |  |  |  |  |  |  |  |  |  |  |
| Sought medical care independently (from any source) | |  |  |  |  |  |  |  |  |  |  |  |  |  |  |  |  |  |  |  |  |
| **Adverse Events SKIP** only if “2”, “55” or “99” entered in first row. Otherwise, always RECORD either “yes” (1) or “no” (2) | | | | | | | | | | | | | | | | | | | | | |
| **Hospitalized**** | |  |  |  |  |  |  |  |  |  |  |  |  |  |  |  |  |  |  |  |  |
| **Death**** | |  |  |  |  |  |  |  |  |  |  |  |  |  |  |  |  |  |  |  |  |
| **Daily FRA Code** | |  |  |  |  |  |  |  |  |  |  |  |  |  |  |  |  |  |  |  |  |
| **Date (dd/mm)** | |  |  |  |  |  |  |  |  |  |  |  |  |  |  |  |  |  |  |  |  |

*If (1) sample collection is necessary, (2) danger signs are present, or (3) a patient refused to go to the hospital, the FRA will notify the FRO. The FRO will then notify the MT and MO when necessary. The MT will call the FRA to obtain the bari address.

****CALL FRO IMMEDIATELY. THESE ARE SERIOUS ADVERSE EVENTS AND THE FRO MUST FOLLOW UP. ADVERSE EVENT FORM IS NECESSARY.**

# Appendix 11: Secondary/Follow Up Case Specimen Collection Form

| **Contact Name:** | | **Contact Unique ID#:** | |  |  |  |  |  | |  |  |  |  |  |
| --- | --- | --- | --- | --- | --- | --- | --- | --- | --- | --- | --- | --- | --- | --- |
| **Medical Officer/ Medical Technologist ID** | | | | | | | | | | |  |  |  |  |
| **Date of call (dd/mm/yy)** |  | | **Time of call (24 hour format)** | | | | | |  | | | | | |

| **Household ID#** |  |  |  |  |  |  |  |  | **Upazila** | Bajitpur / Kuliar Char |
| --- | --- | --- | --- | --- | --- | --- | --- | --- | --- | --- |
| **Village / Para / Mahalla** |  | | | | | | | | **District** | Kishorgonj |
| **Union / Ward** |  | | | | | | | | **Phone number** |  |

**What type of case is this?**  

**Secondary Case = 1, Follow Up Case = 2, Sick Bari member = 3**

**Did the possible case or his/her guardian give consent/assent?** 

**(Yes = 1, No = 2)**

**Case age ≥18 years, self consent** 

**(Yes = 1, No = 2)**

**Case age < 18 years, guardian consent** 

**(Yes = 1, No = 2)**

**Case age 7 to < 18 years, self assent** 

(Yes = 1, No = 2)

| **Date of collection (dd/mm/yy)** |  | | | | **Time of collection (24 hour format)** |  | |
| --- | --- | --- | --- | --- | --- | --- | --- |
| **Age** (YY-MM) |  |  |  |  | **Sex** (Male=1; Female=2) | |  |

**If the possible case/guardian did NOT give consent/assent, or specimen wasn’t collected, record why below:**

**______________________________________________________________________________**

**______________________________________________________________________________**

**Code of Medical Officer/Technologist: _____________**

**Signature: _____________________________________**

**Date: _________________________________________**

BISTIS: Facilitating Tools Tracking Form

Unique ID: __________ Name of Bari Leader: _____________________________________________

evwoi cÖav‡bi bvg

Code of FIS: _____ Name of FIS: _________________________________________________

Bari location evwoi Ae¯’vb: ______________________________________________________________________________________________________

Record Date and Time of FIS visit to bari on DAY ONE: (dd/mm/yyyy) ______________

GdAvBGm-Gi wfwR‡Ui ZvwiL Ges mgq

**Complete this section on Day One of intervention.**

GB AskwU B›Uvi‡fbk‡bi **cÖ_g** w`‡b mgvß Ki“b

Write the number of the location in the boxes below: Ae¯’v‡bi msL¨v wb‡Pi ev‡· wjLyb

1 next to cooking area (ivbœvN‡ii cv‡k) 4 Uthaan (DVv‡b)

2 next to toilet area (cvqLvbvi cv‡k) 5 next to tubewell (wUDeI‡q‡ji cv‡k)

3 next to household structure (N‡ii KvVv‡gvi cv‡k) 6 other (write in other location on line below) (Ab¨vb¨, wb‡P wjLyb)

7 not found ( ‡`Lv hvqwb )

1. Site of bari’s handwashing station - must include water and soap □

(evwoi nvZ †avqvi RvqMvi Ae¯’vb -Aek¨B Zvi g‡a¨ cvwb I mvevb _vK‡Z n‡e ):

Other: (Ab¨vb¨: wb‡Pi jvB‡b wbw`©ó K‡i wjLyb)

________________________________________________________________________

2. Site of primary intervention handwashing station (cÖvBgvix B›Uvi‡fbkY nvZ †avqvi RvqMvi Ae¯’vb): □

Other: (Ab¨vb¨: wb‡Pi jvB‡b wbw`©ó K‡i wjLyb)

________________________

3. Site of secondary intervention handwashing station (†m‡KÛvix B›Uvi‡fbkY nvZ †avqvi RvqMvi Ae¯’vb): □

cÖ‡hvR¨ bq (Not Applicable):........................8

(Other: (Ab¨vb¨: wb‡Pi jvB‡b wbw`©ó K‡i wjLyb)

________________________________________________________________________

Number of Bari Members present for intervention on Day 1: _______ children (2-17 years of age) from Bari _______ adults (**>**18 years of age) from Bari

**Complete this section on day one, and all additional days as benefits and barriers are identified.**

(GB AskUzKz **cÖ_g w`b Ges Ab¨vb¨ AwZwi³ w`‡b** c~iY Kiæb hLb myweav Ges Amyweav wPwýZ n‡e)

**Benefits** of handwashing with soap identified by bari members evwoi m`m¨iv mvevb w`‡q nvZ †avqvi †h ‡h **myweav** wPwýZ K‡i‡Q

(If bari members mention the listed benefit, write “1” for “yes” in the space. At the END of the Intervention Period, if bari members did not mention the listed benefit, write “2” for “no” in the space. hw` evwoi m`m¨ wb‡gœv³ myweav mg~n wPwýZ K‡i Zvn‡j n¨v DË‡ii Rb¨ 1 †KvW wjLyb B›Uvi‡fkb †k‡l †h †h myweav wPwýZ nqwb Zvi R‡b¨ 2 †KvW Ki“b ):

□ 4a. Nurture child or family (cwievi ev wkïi hZœ †bqv)

□ 4b. Be accepted member of society (mgv‡Ri m`m¨ wn‡m‡e MÖnb‡hvM¨Zv)

□ 4c. Reduced diarrhoeal disease (Wvqwiqv Kg nq)

□ 4d. Remove disgusting substances (†bvsiv †_‡K cwi®‹vi _vKv hvq)

□ 4e. Improved health (DbœZ ¯^v¯’¨)

□ 4f. Reduced respiratory illness (k¦v‡mi/Kvwki AmyL Kg nq)

□ 4g. Look, feel, smell clean (cwi®‹vi-cwi”Qbœ †`Lvq Ges fv‡jv †eva nq)

□ 4h. Enhanced social status (mvgvwRK gh©v`v ev‡o)

□ 4i. Soap repels germs ( mvevb Rxevby `~i K‡i)

□ 4j . Write any additional benefits identified below: (Ab¨vb¨ †Kv‡bv myweavi K_v e‡j _vK‡j wb‡P wjLyb)

#### Identify barriers and solutions of handwashing with soap. Identify those barriers stated by bari members as reasons they find it difficult to wash hands with soap. (If a barrier is mentioned, write “1” for “yes” in the space. At the END of the Intervention Period, if bari members did not mention a barrier, write “2” for “no” in the space.) Identify solution/s that bari members indicate are most appropriate for the stated barrier. (Focus solutions on the most appropriate solution/s, not all solutions.) (If a solution is mentioned, write “1” for “yes” in the space. At the END of the Intervention Period, if bari members did not mention a solution, write “2” for “no” in the space.)

#### mvevb w`‡q nvZ †avqvi ‡¶‡Î evav Ges Zvi mgvavb wPwýZ Ki“b| evwoi m`m¨iv †h †h Kvi‡Y mvevb w`‡q nvZ ay‡Z mgm¨vi g‡a¨ c‡ib, †m¸‡jv‡K evav wnmv‡e wPwýZ Ki“b (hw` evwoi m`m¨ wb‡gœv³ evav mg~n wPwýZ K‡i Zvn‡j n¨v DË‡ii Rb¨ 1 †KvW wjLyb B›Uvi‡fkb †k‡l †h †h evav wPwýZ nqwb Zvi R‡b¨ 2 †KvW Ki“b ) evox m`m¨iv wbw`©ó evavi Rb¨ †h †h mgvavb D‡j­L K‡i Zv wPwýZ Ki“b|( me mgvavb bq eis †h mgvavbwU †ewk cÖ‡hvR¨ †mUv‡K †ewk ¸i“Z¡ w`b) hw` evwoi m`m¨ wb‡gœv³ mgvavb mg~n wPwýZ K‡i Zvn‡j n¨v DË‡ii Rb¨ 1 †KvW wjLyb B›Uvi‡fkb †k‡l †h †h mgvavb wPwýZ nqwb Zvi R‡b¨ 2 †KvW Ki“b )

□ 5. Not part of routine or habit Solution/s: □ 5a. BISTIS cue cards as reminders wemwUm wKD KvW©

Af¨vm †bB mgvavb □ 5b. BISTIS FIS frequent reminders wemwUm GdAvBGm evievi g‡b Kwi‡q †`qv

□ 5c. Bari leader / elder daily or frequent reminders evwo cÖavb ev e‡qv‡Rô¨ †KD

cÖwZw`b g‡b Kwi‡q †`qv

□ 5d. Teach children now so it becomes habit wkï‡`i GLb †_‡KB wk¶v †`qv hv‡Z Af¨v‡m cwiYZ nq

□ 5e. Place handwashing station in common area in central location mevB e¨envi Ki‡Z cv‡i Ggb ¯’v‡b nvZ †avqvi RvqMv ivLv

□ 5f. Other: Ab¨vb¨: wb†Pi jvB‡b wjLyb| ______________________________________________________

□ 6. Unaware of benefits/lack of knowledge Solution/s: □ 6a. Benefits fact sheet review wemwUm d¨v± wkU cov

Áv‡bi Afve/myweav Rv‡bb bv mgvavb □ 6b. Influenza education flash cards, influenza fact sheet review Bbd¬z‡qÄv

m¤úwK©Z Z‡_¨i wd¬c KvW©, Bbd¬‡qÄv d¨v± wkU cov

□ 6c. BISTIS cue cards for important times review ¸iZ¡c~Y© mg‡qi R‡b¨ wemwUm wKD KvW© wiwfD

□ 6d. Other: Ab¨vb¨: wb†Pi jvB‡b wjLyb|

______________________________________________________

□ 7. Too much energy / laziness Solution/s: □ 7a. Place handwashing station in common area in central location

AZ¨waK PÂjZv ev AjmZv mgvavb mevB e¨envi Ki‡Z cv‡i Ggb ¯’v‡b nvZ †avqvi RvqMv ivLv

□ 7b. Benefits fact sheet review: handwashing can improve health, save more time and energy d¨v± wkU wiwfD: nvZ ay‡j ¯^v¯’¨ fv‡jv _v‡K, mgq Ges kw³ euv‡P|

□ 7c. Benefits fact sheet review: handwashing steps take only a few seconds

d¨v± wkU wiwfD: nvZ ay‡Z gvÎ K‡qK †m‡KÛ mgq jv‡M

□ 7d. Other: Ab¨vb¨, wb‡Pi jvB‡b wjLyb

______________________________________________________

□ 8. Soap costs too much Solution/s: □ 8a. Benefits fact sheet review: soap costs little, can improve health,

mvev‡bi LiP AZ¨waK †ewk mgvavb people can work more to earn more when they’re healthier

d¨v± wkU wiwfD: mvev‡bi `vg Kg, GwU ¯^v¯’¨ fv‡jv iv‡L, my¯’¨ gvbyl ‡ewk KvR K‡i d‡j †ewk DcvR©Y K‡i|

□ 8b. FIS will give soap when needed for handwashing for 2 weeks hLb cÖ‡qvRb, ZLb GdAvBGm `yB mßv‡ni R‡b¨ nvZ †avqvi mvevb w`‡Z cv‡i|

□ 8c. Other: Ab¨vb¨, wb‡Pi jvB‡b wjLyb

______________________________________________________

□ 9. Won’t prevent illness Solution/s: □ 9a. Influenza education flash cards, benefits fact sheet review

mvevb †iv‡Mi nvZ †_‡K euvPvq bv mgvavb Bbd¬z‡qÄv welqK wk¶vi wd¬c KvW©, d¨v± wkU wiwfD

□ 9b. Other: Ab¨vb¨, wb‡Pi jvB‡b wjLyb

______________________________________________________

□ 10. Ash or mud cleans Solution/s: □ 10a. Barriers and solutions fact sheet review: ash & mud may not

QvB ev Kuv`v A`„k¨ Rxevby `~i K‡i mgvavb clean invisible germs

cÖwZeÜKZv Ges mgvavb d¨v± wkU wiwfD: QvB Ges Kuv`v cwi®‹vi K‡i bv|

□ 10b. Other: Ab¨vb¨, wb‡Pi jvB‡b wjLyb

______________________________________________________

□ 11. Water by itself cleans Solution/s: □ 11a. Barriers and solutions fact sheet review: water alone may not

cvwb wb‡R †_‡KB A`„k¨ Rxevby `~i K‡i mgvavb clean invisible germs

cÖwZeÜKZv Ges mgvavb d¨v± wkU wiwfD: ïay cvwb Rxevby cwi®‹vi K‡i bv|

□11b. Other: Ab¨vb¨, wb‡Pi jvB‡b wjLyb

______________________________________________________

□ 12. Too busy / no time Solution/s: □ 12a. Place handwashing station in common area in central location

AZ¨waK e¨¯ÍZv/mg‡qi Afve mgvavb mevB e¨envi Ki‡Z cv‡i Ggb ¯’v‡b nvZ †avqvi RvqMv ivLv

□ 12b. Benefits fact sheet review: handwashing can improve health, more time and energy for daily tasks

d¨v± wkU wiwfD: nvZ ay‡j ¯^v¯’¨i DbœwZ nq, ˆ`bw›`b Kv‡Ri R‡b¨ mgq Ges kw³ euv‡P

□ 12c. Benefits fact sheet review: handwashing steps take only a few seconds

d¨v± wkU wiwfD: nvZ ay‡Z gvÎ K‡qK †m‡KÛ mgq jv‡M

□ 12d. Other: Ab¨vb¨, wb‡Pi jvB‡b wjLyb

______________________________________________________

□ 13. Soap will be stolen Solution/s: □ 13a. BISTIS soap box can protect soap from animals and crows

mvevb Pzwi n‡q hvq mgvavb wemwUm mvev‡bi ev‡·i Kvi‡Y KvK ev Ab¨ cÖvbx mvevb wb‡Z cvi‡e bv|

□ 13b. Take soap in at night to prevent theft iv‡Z Pzwii nvZ †_‡K evP‡Z mvevb N‡i ivLv

□13c. Other: Ab¨vb¨, wb‡Pi jvB‡b wjLyb

______________________________________________________

□ 14. No handwashing station/too far away Solution/s: □ 14a. BISTIS handwashing station will be given to keep in bari

nvZ †avqvi †Kv‡bv RvqMv †bB ev A‡bK `~‡i mgvavb wemwUm nvZ †avqvi ¯’vb evwo‡Z †`‡e

□ 14b. Place handwashing station in common area in central location mevB e¨envi Ki‡Z cv‡i Ggb ¯’v‡b nvZ †avqvi RvqMv ivLv

□14c. Other: Ab¨vb¨, wb‡Pi jvB‡b wjLyb

_____________________________________________________

□ 15. Don’t care about handwashing Solution/s: □ 15a. Barriers and solutions fact sheet review: handwashing with soap

with soap mgvavb can improve health, save cost of medicine / clinic visits

mvevb w`‡q nvZ †avqv `iKvix g‡b Kwi bv cÖwZeÜKZv Ges mgvavb d¨v± wkU wiwfD: mvevb w`‡q nvZ a~‡j ¯^v¯’¨ fv‡jv _v‡K, Jla ev wPwKrmvi LiP euv‡P

□ 15b. Barriers and solutions fact sheet review: handwashing with soap may prevent illness, more time to work or spend in fields

cÖwZeÜKZv Ges mgvavb d¨v± wkU wiwfD: mvevb w`‡q nvZ a~‡j †ivM cÖwZ‡iva nq, †¶‡Z ev KvR Kivi Rb¨ †ewk mgq cvIqv hvq|

□ 15c. Other: Ab¨vb¨, wb‡Pi jvB‡b wjLyb

______________________________________________________

□ 16. Forget to wash hands with soap Solution/s: □ 16a. Place handwashing station in common area in central location

mvevb w`‡q nvZ a~‡Z fz‡j hvb mgvavb mevB e¨envi Ki‡Z cv‡i Ggb ¯’v‡b nvZ †avqvi RvqMv ivLv

□ 16b. BISTIS cue cards as reminders wemwUm wKD KvW

□ 16c. Benefits fact sheet review: handwashing can improve health, more time and energy for daily tasks

d¨v± wkU wiwfD: nvZ ay‡j ¯^v¯’¨i DbœwZ nq, ˆ`bw›`b Kv‡Ri R‡b¨ mgq Ges kw³ euv‡P

□ 16d. Benefits fact sheet review: handwashing steps take only a few seconds

d¨v± wkU wiwfD: nvZ ay‡Z gvÎ K‡qK †m‡KÛ mgq jv‡M

□ 16e. Other: Ab¨vb¨, wb‡Pi jvB‡b wjLyb

______________________________________________________

□ 17. Write any additional barriers and solutions identified below: GQvovI †Kv‡bv AwZwi³ cÖwZeÜKZv ev mgvavb _vK‡j wb‡P wjLyb|

**COMPLETE NEXT SECTION DAILY DURING INTERVENTION. Yes = 1 No = 2**

**wb‡Pi AskwU cÖwZw`b B›Uvi‡fbk‡bi mgq c~iY Ki“b| nu¨v = 1, bv = 2**

| Day of field visit | Date (dd/mm) | Time (24 hour format) | Group Level Education delivered | Soap present at main HW station | Soap weighed at main HW station | Weight of soap at main HW station  (in grams) | Soap replaced at main HW station | Water present at main HW station | Weight of soap at secondary HW station (in grams) | Number of soap bars given | Group Skill Training delivered | Role Modeling delivered | Individual Demonstration delivered | **** RASH or other skin problem due to our soap** | Time spent in bari | FIS Code | FRO Code |
| --- | --- | --- | --- | --- | --- | --- | --- | --- | --- | --- | --- | --- | --- | --- | --- | --- | --- |
| *Deliver on days:* |  |  | *Day 1 & 2 and 7 (Use Influenza fact sheet only)* | *All days* | *As needed* | *As needed* | *As needed* | *All days* | *As needed* | ***MUST FILL IN DAILY*** | *Days 1, 2, 3 or 4 and 9* | *Days 1, 2, 3 or 4 and 9* | *Days1,2, 3 or 4, & 6, 8, 10, 12,14 and 16* | ***MUST FILL IN DAILY*** | ***In minutes*** | ***MUST FILL IN DAILY*** | ***MUST FILL IN DAILY*** |
| 1 |  |  |  |  |  |  |  |  |  |  |  |  |  |  |  |  |  |
| 2 |  |  |  |  |  |  |  |  |  |  |  |  |  |  |  |  |  |
| 3 |  |  |  |  |  |  |  |  |  |  |  |  |  |  |  |  |  |
| 4 |  |  |  |  |  |  |  |  |  |  |  |  |  |  |  |  |  |
| 5 |  |  |  |  |  |  |  |  |  |  |  |  |  |  |  |  |  |
| 6 |  |  |  |  |  |  |  |  |  |  |  |  |  |  |  |  |  |
| 7 |  |  |  |  |  |  |  |  |  |  |  |  |  |  |  |  |  |
| 8 |  |  |  |  |  |  |  |  |  |  |  |  |  |  |  |  |  |
| 9 |  |  |  |  |  |  |  |  |  |  |  |  |  |  |  |  |  |
| 10 |  |  |  |  |  |  |  |  |  |  |  |  |  |  |  |  |  |
| 11 |  |  |  |  |  |  |  |  |  |  |  |  |  |  |  |  |  |
| 12 |  |  |  |  |  |  |  |  |  |  |  |  |  |  |  |  |  |
| 13 |  |  |  |  |  |  |  |  |  |  |  |  |  |  |  |  |  |
| 14 |  |  |  |  |  |  |  |  |  |  |  |  |  |  |  |  |  |
| 15 |  |  |  |  |  |  |  |  |  |  |  |  |  |  |  |  |  |
| 16 |  |  |  |  |  |  |  |  |  |  |  |  |  |  |  |  |  |

** If any bari member develops a skin rash or other problem as a result of the intervention soap, FIS MUST CALL THE M.O. IMMEDIATELY.

* Write 999 if soap is stolen, taken away by crow or lost

*Replace soap at 20 grams or less.

Last date of intervention _______________________________

FIS Signature on last date of intervention ________________________________________________________

FRO Signature on last date of intervention _______________________________________________________

Appendix 13: AQM Tracking Form

**Device ID: __________ FRA Name:______________________ FRA Code:**

**Placement instructions**:

**Tips for good time measures**:

1) Use a reliable time source such as the internet to set the computer’s clock.

2) Synchronize field watch to computer’s clock.

1) When possible, place the monitor on the wall of the room of interest (instead of hanging the monitor).

2) Locate the monitor 100 centimeters from the edge of the combustion zone of the stove. Measure distance as the shortest, horizontal line

possible (i.e., parallel to the floor, from the closest edge of the combustion zone to the wall underneath where the monitor is to be placed).

3) Locate the monitor at a height of 145 centimeters above the floor. Correctly positioning the height of the monitor is important.

4) Locate the monitor at least 150 centimeters away (horizontally) from openable doors and windows, where possible.

Since it may be difficult to simultaneously satisfy all recommendations, simply choose the best possible location.

For additional instructions, see UC Berkeley manual of standard operating procedures for *Installing Indoor Air Pollution Instruments in a Home*

**|------------------FRO Responsibilities---------­-------------| |-----------------------------------FRA Responsibilities---------------------------------------------| |------FRO Responsibilities---|**

| **Battery change (Yes / No)** | **Device**  **Initialization Date** | **Device Launch Date & Time** | **Zeroing**  (Before the monitor leaves the office) | | **HHID** | | | | | | | | **Sleeping Room**  **or**  **Kitchen** | | **Monitor Placement Distances**  **(centimeters)** | | | **Monitoring** | | **Zeroing**  (Zeroing after retrieval / post placement) | | **Data Download**  **(Yes/No)** |
| --- | --- | --- | --- | --- | --- | --- | --- | --- | --- | --- | --- | --- | --- | --- | --- | --- | --- | --- | --- | --- | --- | --- |
|  |  |  | **Start Date & Time**  (hh:mm) | **End Date & Time** (hh:mm) |  |  |  |  |  |  |  |  |  |  | **To edge of stove** | **From floor** | **To nearest**  **door or**  **window** | **Placement Date & Time (hh:mm)** | **Retrieval Date & Time**  (hh:mm) | **Start Date & Time**  (hh:mm) | **End Date & Time** (hh:mm) |  |
|  |  |  |  |  |  |  |  |  |  |  |  |  | |  |  |  |  |  |  |  |  |  |
|  |  |  |  |  |  |  |  |  |  |  |  |  | |  |  |  |  |  |  |  |  |  |

**Maintenance and storage:** The photoelectric (PE) chamber should be cleaned at least every 10 uses to ensure that the particle measurements are accurate. See UC Berkeley *User Manual.* All monitors should be stored in sealed Ziplock bags to prevent dust from accumulating inside the monitor. Also prevent the instruments from being bumped or dropped.

**Comments:**

**________________________________________________________________________________________________________________________________________________**

#

**Appendix 14: Hospital Check List Form Version 23.07.10**

| **Eligibility Section**   |  | | | | | | | | | | | | | | | | | --- | --- | --- | --- | --- | --- | --- | --- | --- | --- | --- | --- | --- | --- | --- | --- | | **1. MO Code:** | | | | | | | | | | | | | | | | | **4. Which recruitment site? Bajitpur = 1 Kuliachar = 2 JIMCH = 3 Pharmacy = 42. Name: 3. Date:** | | | | |  | | | |  | **5.Time (24 hour format) :** |  |  |  |  |  | | **6. Age** (YY-mm): | | | | | |  | |  |  |  | | | | | | | 7. Sex (Male =1, female =2) |  |  |  |  |  | |  | |  | | | | | | | | | | |
| --- | --- | --- | --- | --- | --- | --- | --- | --- | --- | --- | --- | --- | --- | --- | --- | --- | --- | --- | --- | --- | --- | --- | --- | --- | --- | --- | --- | --- | --- | --- | --- | --- | --- | --- | --- | --- | --- | --- | --- | --- | --- | --- | --- | --- | --- | --- | --- | --- | --- | --- | --- | --- | --- | --- | --- | --- | --- | --- | --- | --- | --- | --- | --- | --- | --- | --- | --- | --- | --- | --- | --- | --- | --- | --- | --- | --- | --- | --- | --- | --- | --- | --- | --- |
| **Age-specific case definitions for index case-patients:**  (Yes=1, No=2, Don’t Know = 9) | **Patient > 5 years**  **Yes / No** | **Patient < 5 years**  **Yes / No** | **Date of onset**  **(**dd-mm-yy)  Approximate time of onset  (00:00 24-hour format) |
| 8. Fever? |  |  | 9. Date: __ __/__ __/__ __  10. Time: __ __ : __ __  11. MO: Did onset occur within the last 48 hours? Yes (1)/ No(2) |
| 12. Cough? |  |  | 13. Date: __ __/__ __/__ __  14.Time: __ __ : __ __  15. MO: Did onset occur within the last 48 hours? Yes (1)/ No(2) |
| 16. Sore throat? |  |  | 17. Date: __ __/__ __/__ __  18. Time: __ __ : __ __  19. MO: Did onset occur within the last 48 hours? Yes (1)/ No(2) |
| **20. Does the patient meet**  **age-specific case definition?** |  |  | **21. Comment (if any):** |
| **Instruction:** If the patient meets the case definition, then consider him/her a **‘suspected index case-patient’** and ask questions below under **“Additional inclusion criteria”**; otherwise thank the patient and stop the interview at this point.  **The age-specific case definitions for index case-patients are:**   - **Persons≥ 5 years old: Influenza-like illness (ILI), defined as history of fever, and either cough *or* sore throat with onset within the last 48 hours.** - **Persons < 5 years old: any child with acute fever with onset within the last 48 hours** | | | |

| **Additional inclusion criteria**  22. Has any other resident in your *household* had **fever** during the past 7 days?  (yes=1, no = 2, don’t know = 9)  *🡪If yes, patient is ineligible, SKIP the remaining questions and record ‘No’ for the question ‘Does the patient meet the additional inclusion criteria’ below. If no, then continue.*  22a. Has any other resident in your *bari* had **fever** during the past 7 days?  (yes=1, no = 2, don’t know = 9)  *🡪If no or don’t know, SKIP to Question 23. If yes, proceed to Question 22b.*  22b. Who had fever and for how many days did s/he have fever?  1. Name/Relation: _______________________ Days of fever (1-7, don’t know=99) ________  2. Name/Relation: _______________________ Days of fever (1-7, don’t know=99) ________  3. Name/Relation: _______________________ Days of fever (1-7, don’t know=99) ________  *🡪If any bari member had fever for 2 or more days out of the last 7 days, patient is ineligible, SKIP the remaining questions and record ‘No’ for the question ‘Does the patient meet the additional inclusion criteria’ below. If bari members(s) had fever for only 1 day or the patient does not know the duration of fever, then continue.* |
| --- |
| 23. Does the patient live within 30 minutes of either UHC or JIMCH? (Yes = 1, No = 2)  *🡪If no, patient is ineligible, SKIP the remaining questions and record ‘No’ for the question ‘Does the patient meet the additional inclusion criteria’ below. If yes, then continue.*  24. Which upazilla do you live in? Bajitpur = 1/Kuliachar = 2/Others = 8  If other then please specify ___________________________________________  25. Will you be living in your *bari* during the next 20 days? (yes=1, no = 2)      *🡪If no, patient is ineligible, SKIP the remaining questions and record ‘No’ for the question ‘Does the patient meet the additional inclusion criteria’ below. If yes, then continue.*  26. Will 2 or more other persons be living in your *bari* during next 20 days? (yes=1, no = 2)    *🡪If no, patient is ineligible, SKIP the remaining questions and record ‘No’ for the question ‘Does the patient meet the additional inclusion criteria’ below. If yes, then continue.*  27. Was the patient previously enrolled in our study? (yes = 1, no = 2**)**  *🡪If yes, patient is ineligible, SKIP the remaining questions and record ‘No’ for the question ‘Does the patient meet the additional inclusion criteria’ below. If no, then continue.* |
|
| 28. Patient order for hospitalization and was hospitalized? (yes = 1, no = 2**)**  *🡪If yes, patient is ineligible, SKIP the remaining questions and record ‘No’ for the question ‘Does the patient meet the additional inclusion criteria’ below. If no, then continue.*  29. Patient ordered for admission but refused admission (yes=1, no=2)  *🡪If yes (patient refused admission), then eligible and continue* |
| *is ineligible, SKIP the remaining questions and record ‘No’ for the question ‘Does the patient meet the additional inclusion criteria’ below. If yes, then continue.* |
| **30. Does the patient meet the additional inclusion criteria?** (yes=1, no = 2) |
|  |

| 31. Have you received a vaccination for influenza or Swine Flu/H1N1 in the past six months?  (Yes=1, No=2) | | | |
| --- | --- | --- | --- |
| **Possible Treatments:** | | | |
| 32. Is the patient being treated with medications for the symptoms indicated above? (Yes=1, No=2)  **If no, skip following section** | | | |
| What medications is the patient being treated with? List below | | | |
|  | | | |
| 33. Name of the Drug | 34. Was the patient prescribed the drug?  (Yes = 1, No = 2) | 35. Code of the Drug  (Couldn’t mention Drug name = 999) | 36. Has the patient begun treatment with the drug?  (Yes = 1, No = 2) |
|  |  |  |  |
|  |  |  |  |
|  |  |  |  |
|  |  |  |  |

**PCR Specimen Collection Section**

**37. Unique ID #**

|  |  |  |  |
| --- | --- | --- | --- |

| **38. Time Sample collected (24 hour format):** | Time: __ __ : __ __ |
| --- | --- |

Signature of Medical Officer:___________________________ Date: ___________________

### Appendix 15a: VENTILATION ASSESSMENT FORM: SLEEPING ROOM

***Note: Complete one ventilation assessment form for each room where a particle monitor is placed.***

Household ID:  Date: ___ /___/___ FRA code: 

**1.** Measure the dimensions of the room in centimeters. Width (centimeters): __________________ Length (centimeters): __________________

**2.** How many people of any age sleep there:  **3**. How many children < 5 years old sleep there: 

**4.** How many **hours** during the 24 hours the AQM was present in the room was the **ceiling** fan turned on (record 99 for “NA”): 

**5.** How many **hours** during the 24 hours the AQM was present in the room was the **table** fan turned on (record 99 for “NA”): 

**6*.* Drawing Instructions**: *Using the symbols below, indicate the locations of stoves or burners, existing windows, doors, ventilators, indoor walls, index case-patient’s bed, and the particle monitoring device. The wall on which the particle monitor is placed should be drawn as the top wall on the diagram.*


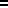


Stove/Burner Window Door Ventilators Indoor Wall Study Child’s Bed AQM

CF

TF

**CF TF**

Ceiling Fan Table Fan

### Appendix 15b: VENTILATION ASSESSMENT FORM: COOKING AREA

ONLY TO BE ASSESSED IN BARIS WITH FULLY ENCLOSED KITCHENS

***Note: Complete one ventilation assessment form for each room where a particle monitor is placed.***

Household ID:  Date: ___ /___/___ FRA code: 

**1.** Measure the dimensions of the room in centimeters. Width (centimeters): __________________ Length (centimeters): __________________

**2.** How many people use this cooking area: 

**3.** How many **hours** during the 24 hours the AQM was present in the room was the **ceiling** fan turned on (record 99 for “NA”): 

**4.** How many **hours** during the 24 hours the AQM was present in the room was the **table** fan turned on (record 99 for “NA”): 

**5*.* Drawing Instructions**: *Using the symbols below, indicate the locations of stoves or burners, existing windows, doors, ventilators, indoor walls, and the particle monitoring device. The wall on which the particle monitor is placed should be drawn as the top wall on the diagram.*


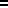


CF

Stove/Burner Window Door Ventilators Indoor Wall Particle Monitor Ceiling Fan

TF

Table Fan

# Appendix 16: Consent Form: Follow Up Study Enrollment for Bari

**Protocol Number: 2009-004**

**Protocol Title:** Prevention of secondary transmission of human influenza by promoting handwashing with soap: The Bangladesh Interruption of Secondary Transmission of Influenza Study (BISTIS) Follow Up Study

**Investigator’s name**: Dr. Eduardo Azizz-Baumgartner

**Organization:** ICDDR,B

**Introduction**

In 2009, your Bari took part in a research study about influenza. The research study was conducted by scientists from the International Centre for Diarrhoeal Diseases Research, Bangladesh (ICDDR,B), Centers for Disease Control and Prevention (CDC), and University at Buffalo, a university in the USA. We are now here to talk with you about a second part of that study. We would like to understand household practices and illness experiences of members of your bari.

**Purpose of the research**

We are trying to how household practices can change over time and how these practices may affect certain illnesses, like diarrhea and respiratory illness.

**Why selected**

Your Bari took part in the first part of our study last year. This second part is being completed only in baris that were enrolled in the first part of the study.

**What is expected from the members of your Bari?**

If you agree to enrolling your Bari in the study:

I will visit your bari to observe your bari’s household practices three times over the next three or four months.

At two of the visits, I will speak to the person who came to the Jahurul Islam Medical College Hospital with influenza or another respiratory illness in (month) 2009. If that person was a child under 15 years old, I will speak to his/her parent or guardian. I will ask some questions about household practices. I will also make some quick observations of your bari at each of the visits. At one of the visits, I will also sit in the uthaan for about 90 minutes to observe your bari’s household practices.

During these visits I will also ask each member of your bari if they have had symptoms of fever, respiratory illness, or gastrointestinal illness, in the previous 48 hours.

**(Following paragraph to be read aloud if SmartSoap will be used in the bari.)**

During one of the visits, I will give your bari a bar of special Lifebuoy soap to use for three days, after which I will return to collect the bar of soap two days later. The special Lifebuoy soap is almost the same as Lifebuoy soap available in the market and provides the same benefits. You will likely not be able to tell the difference between the special and regular soap. The special Lifebuoy soap should be used as soap is usually used in your bari. The special Lifebuoy soap that we are giving you has an electronic device that collects information on soap use. People who are sensitive to Lifebuoy soap or who experience skin reactions to Lifebuoy soap will experience similar reactions to the Lifebuoy soap that we are giving you and should not use it.

Also at the third visit, I will ask two members of your bari to give me their mobile numbers. I will thereafter call that person twice per week for six months to determine if any of the members of your bari have a fever.

After the third visit, for those people who have a fever, we will ask them to allow us to take a nose and throat swab; one of our trained research personnel will have to place a swab into their nose and a different swab into their throat.

**Risk and benefits**

The process of having someone visit your home may be uncomfortable to you. However, we do not expect any harm to come to you or your family because of being visited.

**(Following paragraph to be read aloud if SmartSoap will be used in the bari.)**

In people who have skin reactions to Lifebuoy soap available in the market, the special Lifebuoy soap may cause similar skin reactions. These people should not use the Lifebuoy soap that we give you. The Lifebuoy soap that we are giving you has a battery in it. The battery is like batteries that are used in watches and cameras. The battery is protected inside a case within the soap. It is very unlikely that you will see the battery. If you do see the battery case within the soap, you may continue to use the soap normally. Please try to remember on which day you saw the case sticking out through the soap. As with any soap, please do not allow children to put it into their mouths.

All Baris that take part in the study will receive the benefit of bars of soap; however, there will be no other immediate benefits. However, this study will help us better understand if handwashing behavior can change over time. It will also help us understand if handwashing behavior is related to respiratory illness and diarrhea.

For those people who have symptoms and who allow us to take a nose and throat swab, one of our trained research personnel will have to place a swab into their nose and a different swab into their throat. This may be uncomfortable. There are no other known risks for this procedure.

The results of the test will not alter in any way the treatment of the person who has symptoms and, thus, the results will not be given to the person who has symptoms.

**Privacy, anonymity and confidentiality**

All of the information we collect about the members of your community will be kept private and confidential. We will keep all data in a locked cabinet. We will not give any information about your community to anyone not involved in the study.

**Future use of information**

If the information we collect needs to be used for future use by other researchers, we will not supply any personal information and will maintain strict privacy.

**Right not to participate and withdraw**

You may choose to allow your Bari to take part or not to take part in this study. You may refuse to take part at any time. You may also withdraw your Bari from the study at any time. Refusal to participate or withdrawal from the study will involve no penalty or loss of benefits for the members of your community at the clinic or hospital. Even if you do not enroll your Bari in the study, everyone in your Bari will still receive the usual care at the clinic. Each individual in your Bari may choose to participate or not participate, and may choose to withdraw from the study at any time.

**Principle of compensation**

There is no cost to you or your bari for participation in this study. Other than receiving free soap, you will not receive any compensation for being in the study.

**Persons to Contact:**

If you have questions during the procedure, ask at any time. If you have any additional questions about the surveillance you may contact:

Dr. Eduardo Azizz-Baumgartner, Programme on Infectious Diseases and Vaccine Sciences (PIDVS), ICDDR,B, Mohakhali, Dhaka 1212. Phone: 8860523-32 # 250025, 01711697962

If you have questions about your rights in regards to being part of this research surveillance or if you think some harm has been done to you because of the surveillance you may contact:

Mr. M. A. Salam, Research and Project Support Department (RPSD), ICDDR,B, Mohakhali, Dhaka 1212. Phone: 9886489, 01711428989

If you agree to enrolling your Bari in our study, please indicate that by putting your signature or your left thumb impression at the specified space below.

Thank you for your cooperation.

_______________________________________ ____________________

Signature or left thumb impression of subject Date

_______________________________________ ____________________

Signature or left thumb impression Date

of attendant/Guardian

_______________________________________ ____________________

Signature or left thumb impression of the witness Date

_______________________________________ ___________________

Signature of the PI or his/her representative Date

### Appendix 17: Follow Up Bari Eligibility Form

1. Index case ID: __ __ __ __

2. FRA code: __ __ __

**First Visit**

**3. Date of first visit (dd/mm/yy) ___/ ___/ ___**

| 4. Did bari enrollment occur?  Yes (1) No (2)  (If ‘yes’, skip remaining questions |  |
| --- | --- |

| 5. Why did bari enrollment not occur?    No adults present to sign consent form………………….1  Head of bari would not sign consent form………………2  Unable to find bari members…………………………….3  Other, specify below…………………………………….8    **__________________________________________________________** |  |
| --- | --- |

**Second Visit**

**6. Date of second visit (dd/mm/yy) ___/ ___/ ___**

| 7. Did bari enrollment occur?  Yes (1) No (2)  (If ‘yes’, skip remaining questions |  |
| --- | --- |

| 8. Why did bari enrollment not occur?    No adults present to sign consent form………………….1  Head of bari would not sign consent form………………2  Unable to find bari members…………………………….3  Other, specify below…………………………………….8    **__________________________________________________________** |  |
| --- | --- |

To be answered after completion of follow up study (or before depending on drop out date):

| 9. Did the household decide not to participate at some point during the follow up study?  Yes (1) No (2)  (If ‘yes’, explain why below) |  |
| --- | --- |

| 10. Explanation of household drop out:  11. Date of drop out (dd/mm/yyyy):_____/______/_______ |
| --- |

# Appendix 18: BISTIS Follow Up Survey Form

**1. Ask the respondent: Name of Respondent: _______________________**

**2. How is the respondent related to the index case?**

**Record code in box.**

Self…………………………….…………... 1

Parent/Guardian..…………………………………. 2

Fellow bari member………………………. 3

**3. Respondent (Index case/ Guardian/ Bari member) Unique ID# (Taken from enumeration form) ___ ___ ___ ___ ___ ___ ___ ___ ___ ___**

**4. Index Case Patient Unique ID# ___ ___ ___ ___**

**5. Follow Up Visit Number (1, 2, or 3 ): ______**

**6. Follow Up Visit Date (dd/mm/yy): ___/___/______**

**7. FRA code ___ ___ ___**

**Section One: HAND WASHING STATION QUESTIONS**

**Ask: “Can you please show me where you most often wash your hands?”**

**8. Observation: Is there a designated place for hand washing?**

**(Record number in box):**

Yes……………………………………………….1

No..…………………………..………………….2 🡪 **If the answer to question 8 is “no”, skip to question 12**

**9. Observation: Is the BISTIS intervention handwashing container present at the bari handwashing place?**

**(Record number in box):**

Yes………………………………………………. 1

No..………………………………………………. 2

**10**. **Observation: Is water present at the bari handwashing place?**

**(Record code in box) (You must actually see water to record “yes”):**

Yes………………………………………………. 1

No..………………………………………………. 2

**11. Observation of items present at the handwashing place (Look for each items individually and please do not prompt)**

**(If you observe the listed item, write “1” for “yes” in the box below.**

**If you do not observe the listed item, write “2” for “no” in the box below.)**

Bar soap 

Detergent (powder) 

Liquid soap 

Ash 

Mud/Sand 

Other cleansing agent, specify 

**_____________________________________________**

**12. Ask: Can you show me any items used in your household for washing hands?**

**(If the respondent can show you the item within one minute after s/he goes to get it write “1” for “yes” in the box below.**

**If you do not observe the listed item within one minute after the respondent goes to get the item, write “2” for “no” in the box below. Do NOT prompt)**

Bar soap 

Detergent (powder) 

Liquid soap 

Ash 

Mud/Sand 

Other, specify below 

______________________

**13. Observe: Are intervention cue cards visible in the bari?**

**(Record number in box)):**

Yes 1

No 2 **🡪 If the answer to question 13 is “yes”, skip to section 2**

**Show the respondent an example of the BISITS Intervention Cue Cards.**

**14. Ask the respondent: “Last year, did anyone give your bari cards that looked like this?**

**(Record number in box)):**

Yes 1

No 2 **🡪 If the answer to question 14 is “no”, skip to section 2**

**15. Ask the respondent: “Can you please show me the cards?” Record whether or not the respondent can show you the BISTIS Intervention Cue Cards. (Record number in box)):**

Yes 1

No 2

**Section Two: STRUCTURED OBSERVATION**

**16. Record: Is this the first follow up visit at the bari?**

**(Record number in box):**

Yes 1

No 2🡪 **If the answer to question 16 is “no”, skip to section three**

**16. Record which household is to be observed.**

Record number in box

Index case household 1

Head of bari household 2

Secondary household 3

**17. Record: Total no. of members of household to be observed (if this is ICP household, include ICP): ______**

18. Record Number of members of household to be observed present at the time of observation

**(if this is ICP household, include ICP if present): ______________**

**19. Record the number of individuals in the household to be observed**

Record number in boxes

Children < 5  2.0 Children ≥ 5 to < 18  2.1 Persons ≥ 18 

Say to the respondent: “*I would like to stay with you until about* current time plus 2.5 hours*. I would only like to observe and will not interrupt. If that would be okay now, you may please continue with your normal daily routine. After about 1.5 hours I will ask some people in the bari some questions.”*

**20. Time of beginning observation (24 hrs): hh:mm** :

**21. Time of ending observation (24 hrs): hh:mm**  :

**Comments:** ______________________________________________________________

__________________________________________________________________________

**Section 2. Observation of Hand Wash Opportunities and Behaviors**

| **Line #** | **Exposure:**  Before preparing  /serving food 1  Before eating 2  After cleaning child’s  bottom/nappy 3  After using the toilet 4  After coughing / sneezing 5  After blowing or wiping own  nose 6  After blowing or wiping  child's nose 7  If the hands get visibly soiled... 9  Others: Specify 8 | **Time of observation**  24-hours time  (hh:mm) | **Bari member:**  Child < 5 yrs 1  Child ≥ 5 yrs to < 18 2  Person ≥ 18………………3 | **Gender of bari member:**  Male 1  Female. 2 | **Did bari member clean his/her hand(s)?**  Yes 1  No 2  DK 9  *If 2 or 9 then skip to next episode.* | **Location of hand cleaning**  At a handwashing station belonging to the household (located in or immediately outside household) 1  At a handwashing station belonging to the uthaan/bari 2  In the kitchen area, but not at a handwashing station 3  In the toilet area, but not at a handwashing station 4  In the yard, but not at a handwashing  station. 5  Other: Specify 8 | **Were both hands cleaned?**  Yes 1  No 2  DK 9 | **Hand cleaning materials:**  Bar soap 1  Liquid/Powder soap 2  Ash 3  Mud/Sand 4  Only water 5  Wipe with cloth without water 6  Other:  Specify below 8  Do not know 9 | **Comments:** |
| --- | --- | --- | --- | --- | --- | --- | --- | --- | --- |
| 01 |  |  |  |  |  |  |  |  |  |
| 02 |  |  |  |  |  |  |  |  |  |
| 03 |  |  |  |  |  |  |  |  |  |
| 04 |  |  |  |  |  |  |  |  |  |
| 05 |  |  |  |  |  |  |  |  |  |
| 06 |  |  |  |  |  |  |  |  |  |
| 07 |  |  |  |  |  |  |  |  |  |
| 08 |  |  |  |  |  |  |  |  |  |
| 09 |  |  |  |  |  |  |  |  |  |
| 10 |  |  |  |  |  |  |  |  |  |

**Name of FRA:** _______________________________ **Signature of FRA**: __________________________________ **Date:** ______/______/____________

| **Line #** | **Exposure:**  Before preparing  /serving food 1  Before eating 2  After cleaning child’s  bottom/nappy 3  After using the toilet 4  After coughing / sneezing 5  After blowing or wiping own  nose 6  After blowing or wiping  child's nose 7  If the hands get visibly soiled... 9  Others: Specify 8 | **Time of observation**  24-hours time  (hh:mm) | **Bari member:**  Child < 5 yrs 1  Child ≥ 5 yrs to < 18 2  Person ≥ 18………………3 | **Gender of bari member:**  Male 1  Female. 2 | **Did bari member clean his/her hand(s)?**  Yes 1  No 2  DK 9  *If 2 or 9 then skip to next episode.* | **Location of hand cleaning**  At a handwashing station belonging to the household (located in or immediately outside household) 1  At a handwashing station belonging to the uthaan/bari 2  In the kitchen area, but not at a handwashing station 3  In the toilet area, but not at a handwashing station 4  In the yard, but not at a handwashing  station. 5  Other: Specify 8 | **Were both hands cleaned?**  Yes 1  No 2  DK 9 | **Hand cleaning materials:**  Bar soap 1  Liquid/Powder soap 2  Ash 3  Mud/Sand 4  Only water 5  Wipe with cloth without water 6  Other:  Specify below 8  Do not know 9 | **Comments:** |
| --- | --- | --- | --- | --- | --- | --- | --- | --- | --- |
| 11 |  |  |  |  |  |  |  |  |  |
| 12 |  |  |  |  |  |  |  |  |  |
| 13 |  |  |  |  |  |  |  |  |  |
| 14 |  |  |  |  |  |  |  |  |  |
| 15 |  |  |  |  |  |  |  |  |  |
| 16 |  |  |  |  |  |  |  |  |  |
| 17 |  |  |  |  |  |  |  |  |  |
| 18 |  |  |  |  |  |  |  |  |  |
| 19 |  |  |  |  |  |  |  |  |  |
| 20 |  |  |  |  |  |  |  |  |  |

**Name of FRA:** _______________________________ **Signature of FRA**: ___________________

**Date:** ______/______/____________

| **Line #** | **Exposure:**  Before preparing  /serving food 1  Before eating 2  After cleaning child’s  bottom/nappy 3  After using the toilet 4  After coughing / sneezing 5  After blowing or wiping own  nose 6  After blowing or wiping  child's nose 7  If the hands get visibly soiled... 9  Others: Specify 8 | **Time of observation**  24-hours time  (hh:mm) | **Bari member:**  Child < 5 yrs 1  Child ≥ 5 yrs to < 18 2  Person ≥ 18………………3 | **Gender of bari member:**  Male 1  Female. 2 | **Did bari member clean his/her hand(s)?**  Yes 1  No 2  DK 9  *If 2 or 9 then skip to next episode.* | **Location of hand cleaning**  At a handwashing station belonging to the household (located in or immediately outside household) 1  At a handwashing station belonging to the uthaan/bari 2  In the kitchen area, but not at a handwashing station 3  In the toilet area, but not at a handwashing station 4  In the yard, but not at a handwashing  station. 5  Other: Specify 8 | **Were both hands cleaned?**  Yes 1  No 2  DK 9 | **Hand cleaning materials:**  Bar soap 1  Liquid/Powder soap 2  Ash 3  Mud/Sand 4  Only water 5  Wipe with cloth without water 6  Other:  Specify below 8  Do not know 9 | **Comments:** |
| --- | --- | --- | --- | --- | --- | --- | --- | --- | --- |
| 21 |  |  |  |  |  |  |  |  |  |
| 22 |  |  |  |  |  |  |  |  |  |
| 23 |  |  |  |  |  |  |  |  |  |
| 24 |  |  |  |  |  |  |  |  |  |
| 25 |  |  |  |  |  |  |  |  |  |
| 26 |  |  |  |  |  |  |  |  |  |
| 27 |  |  |  |  |  |  |  |  |  |
| 28 |  |  |  |  |  |  |  |  |  |
| 29 |  |  |  |  |  |  |  |  |  |
| 30 |  |  |  |  |  |  |  |  |  |

**Name of FRA:** ____________________ **Signature of FRA**: ___________________ **Date:** ______/______/___________

| **Line #** | **Exposure:**  Before preparing  /serving food 1  Before eating 2  After cleaning child’s  bottom/nappy 3  After using the toilet 4  After coughing / sneezing 5  After blowing or wiping own  nose 6  After blowing or wiping  child's nose 7  If the hands get visibly soiled... 9  Others: Specify 8 | **Time of observation**  24-hours time  (hh:mm) | **Bari member:**  Child < 5 yrs 1  Child ≥ 5 yrs to < 18 2  Person ≥ 18………………3 | **Gender of bari member:**  Male 1  Female. 2 | **Did bari member clean his/her hand(s)?**  Yes 1  No 2  DK 9  *If 2 or 9 then skip to next episode.* | **Location of hand cleaning**  At a handwashing station belonging to the household (located in or immediately outside household) 1  At a handwashing station belonging to the uthaan/bari 2  In the kitchen area, but not at a handwashing station 3  In the toilet area, but not at a handwashing station 4  In the yard, but not at a handwashing  station. 5  Other: Specify 8 | **Were both hands cleaned?**  Yes 1  No 2  DK 9 | **Hand cleaning materials:**  Bar soap 1  Liquid/Powder soap 2  Ash 3  Mud/Sand 4  Only water 5  Wipe with cloth without water 6  Other:  Specify below 8  Do not know 9 | **Comments:** |
| --- | --- | --- | --- | --- | --- | --- | --- | --- | --- |
| 31 |  |  |  |  |  |  |  |  |  |
| 32 |  |  |  |  |  |  |  |  |  |
| 33 |  |  |  |  |  |  |  |  |  |
| 34 |  |  |  |  |  |  |  |  |  |
| 35 |  |  |  |  |  |  |  |  |  |
| 36 |  |  |  |  |  |  |  |  |  |
| 37 |  |  |  |  |  |  |  |  |  |
| 38 |  |  |  |  |  |  |  |  |  |
| 39 |  |  |  |  |  |  |  |  |  |
| 40 |  |  |  |  |  |  |  |  |  |

**Thank you, that is the end of my observations.**

**Section Three: HAND WASHING DEMONSTRATION**

**Say to the respondent: “Can you please show me exactly what you do when you cough?  Please think about your typical routine, and show me whatever actions are common for you in this situation.  There is no wrong response.  Please do not describe what you would do, but rather, actually show me."**

**22. Record which bari member will demonstrate coughing/sneezing?**

**Record number in box**

Index Case Patient 1

Guardian of Index Case Patient 2

Other, specify below 8

**______________________________________________**

**23. Member who demonstrates coughing/sneezing unique ID: ___ ___ ___ ___ ___ ___ ___ ___ ___ ___**

**24. Record age of member who demonstrates coughing/sneezing (YY/MM): ___ ___/ ___ ___**

**25. Record Time of Observation of coughing/sneezing behavior (24 hour format): ___ ___:___ ___**

**26. Observe: Did the index case patient/guardian/respondent do or say any of the following?**

**If the index case patient/guardian/respondent completes the physical action below, write “1” for “yes” in the column labeled “Observation” below.**

**If the index case patient/guardian/respondent does NOT complete the physical action below, write “2” for “no” in the column labeled “Observation” below.**

**If the index case patient/guardian/respondent says the following, write “1” in the column labeled “Verbal” below.**

**If the index case patient/guardian/respondent does NOT say following, write “2” in the column labeled “Verbal” below.**

**If the index case patient/guardian/respondent BOTH does and says the following, put a “1” in both columns.**

| **Action** | **Observation** | **Verbal** |
| --- | --- | --- |
| Sneeze or cough into shoulder/elbow |  |  |
| Sneeze or cough into sleeve/sari |  |  |
| Sneeze or cough into hands |  |  |
| Sneeze or cough the air |  |  |
| Wash his/her hands with soap and water (type of soap need not be specified)  Time action is done or said (24-hour format): ___:___ |  |  |
| Wash hands with ash/mud/sand and water (use or mention of ash/mud/sand and water, but not soap)          Time of washing hands with water but not soap (24 hour format) ___:___ |  |  |
| Wash hands with water only (use or mention of water but not soap)  Time action is done or said (24-hour format): ___:___ |  |  |
| Wash hands with ash/mud/sand/cloth but no water (no use or mention of water or soap)  Time action is done or said (24-hour format): ___:___ |  |  |

**27. Observe: Did the index case patient/guardian/respondent actually wash hands with at least water within the 5 minutes after coughing?**

**(Record number in box):**

Yes 1

No 2 🡪 **If the answer to question 20 is “no”, skip to section 3**

**28. Observe: Did the index case patient/guardian/respondent use any of the following when s/he washed his/her hands within the 5 minutes after coughing?**

**(If the index case patient/guardian/respondent used the item, write “1” for “yes” in the box below.**

**If the index case patient/guardian/respondent did NOT use the item, write “2” for “no” in the box below.)**

Bar soap 

Detergent (powder) 

Liquid soap 

Ash 

Mud/Sand 

Other, specify below 

______________________

**Section Four: HAND WASHING DEMONSTRATION – Cleaning child after defecation**

**If ICP is < 5years then demonstration should be done with him/her. If ICP is > 5years then look for any < 5 years child in ICP’s household. If no < 5 years child present in ICP’s household then ask the respondent: “Can you bring me to the mother or other primary caregiver of the youngest child who lives in your bari?” If the primary caregiver of the youngest child is not present, ask about the primary caregiver of the next youngest child, until you are able to identify a primary caregiver who is present during the interview time**

**29. Is there any <5years child in the bari?**

**(Record the number in box)**

Yes 1

No 2 🡪 **If answer to the question 22 is “no”, skip to question 31**

**30. Is the child the index case patient?**

Yes 1

No 2

**31. Ask the primary caregiver: “How old is your child?” (YY/MM) ____ ____/____ ____**

**32. Ask the primary caregiver: “How old are you?” (YY/MM) ____ ____ ____ ____**

**33. Record the sex of the primary caregiver:**

**(Record number in box)**

Male 1

Female 2

**34. Record the unique ID of the primary caregiver: ___ ___ ___ ___ ___ ___ ___ ___ ___ ___**

**Say to the primary caregiver: "Can you please show me exactly what you do after your child defecates?  Please think about your typical routine, and show me whatever actions are common for you in this situation.  There is no wrong response.  Please do not describe what you would do, but rather, actually take me through the steps of what you do."**

**Observe whether s/he washes her hands during the 5 minutes after the demonstration. The caregiver should clean the baby just as she would if the baby had actually defecated, including removing the child’s clothes.**

**35. Record time of observation of cleaning the baby’s bottom (24 hour format): ____ ____:____ ____**

**If the person demonstrating completes the physical action below, write “1” for “yes” in the column labeled “Observation” below.**

**If the person demonstrating does NOT complete the physical action below, write “2” for “no” in the column labeled “Observation” below.**

**If the person demonstrating says the following, write “1” in the column labeled “Verbal” below.**

**If the person demonstrating does NOT say following, write “2” in the column labeled “Verbal” below.**

**If the primary caregiver BOTH does and says the following, put a “1” in both columns.**

| **Action** | **Observation** | **Verbal** |
| --- | --- | --- |
| Touch baby’s bottom for cleaning |  |  |
| Use water to clean the baby’s bottom |  |  |
| Use soap to clean the baby’s bottom |  |  |
| Wash his/her hands with soap and water (type of soap need not be specified)  Time action is done or said (24-hour format): ___:___ |  |  |
| Wash hands with ash/mud/sand and water (use or mention of ash/mud/sand and water, but not soap)          Time of washing hands with water but not soap (24 hour format) ___:___ |  |  |
| Wash hands with water only (use or mention of water but not soap)  Time action is done or said (24-hour format): ___:___ |  |  |
| Wash hands with ash/mud/sand/cloth but no water (no use or mention of water or soap)  Time action is done or said (24-hour format): ___:___ |  |  |

**36. Observe: Did the primary caregiver wash hands with water within the 5 minutes after cleaning the child’s bottom?**

**(Record number in box):**

Yes 1

No 2 🡪 **If the answer to question 28 is “no”, skip to question 31**

**37. Observe: Did the primary caregiver use any of the following when s/he washed hands after cleaning the child’s bottom?**

**(If the index case patient/guardian used the item, write “1” for “yes” in the box below.**

**If the index case patient/guardian did NOT use the item, write “2” for “no” in the box below.)**

Bar soap 

Detergent (powder) 

Liquid soap 

Ash 

Mud/Sand 

Other, specify below 

______________________

**Thank the primary caregiver for his/her time. The rest of the interview will be with the original respondent.**

**38. Record: Is this the final follow up visit at the bari?**

**(Record number in box):**

Yes 1

No 2🡪 **If the answer to question 38 is “no”, skip remaining questions**

**Section Four: KNOWLEDGE OF HANDWASHING SECTION**

**39. Ask: “When should you wash your hands?” (Ask this only of the respondent.)**

**If the respondent says the listed item, write “1” for “yes” in the box below.**

**If the respondent does NOT say the listed item, write “2” for “no” in the box below. Do NOT prompt.**

Before cooking food 

Before eating food 

After sneezing or coughing into hands 

After cleaning my or my child’s nose 

After my hands get visibly soiled 

After defecation 

After cleaning a child who has defecated 

During prayer 

During bath 

After waking up from bed 

Other, specify below 

_________________________

**40. Ask: What are the steps you take when you wash your hands?**

**(If the respondent says the listed item, write “1” for “yes” in the box below.**

**If the respondent does NOT say the listed item, write “2” for “no” in the box below. Do NOT prompt.)**

Get hands wet 

Lather hands with soap 

Scrub for 10 seconds 

Rinse hands with water 

Dry Hands 

Other, specify below 

_________________________

**Benefits and Barriers Section**

**41. Ask: “What are the benefits of washing your hands with soap?”**

**(If the respondent says the listed item, write “1” for “yes” in the box below.**

**If the respondent does NOT say the listed item, write “2” for “no” in the box below.)**

Nurture child or family 

Be accepted member of society 

Look, feel, smell clean 

Remove disgusting substances 

Improved Health 

Enhanced social status 

Reduced diarrheal disease 

Reduce respiratory illness 

Other, specify below 

______________________

**42. Ask: “What are the barriers to you washing your hands with soap?”**

**(If the respondent says the listed item, write “1” for “yes” in the box below.**

**If the respondent does NOT say the listed item, write “2” for “no” in the box below.)**

Not part of routine or habit 

Unaware of benefits/lack of knowledge 

Too much energy/laziness 

Soap costs too much 

Won’t prevent illness 

No handwashing station or too far away 

Don’t care about handwashing with soap 

Ash or mud cleans 

Water by itself cleans 

Too busy/no time 

Forgets to wash hands with soap 

Soap will be stolen 

Other, specify  ____________________

No barriers 

**Section Five: KNOWLEDGE OF INFLUENZA SECTION**

**43. Ask: “Have you ever heard of influenza illness?”**

**(Record code in box)**

Yes 1

No 2 🡪 **If the answer to 43 is 2, skip to section six**

Don’t Know/Not Sure 9 **🡪 If the answer to 43 is 9, skip to section six**

**44. Ask: “What symptoms are associated with influenza illness?”**

**(Ask this as an open ended question. If the respondent mentions the listed symptom, write “1” for “yes” in the box below.**

**If the respondent does not mention the listed symptom, write “2” for “no” in the box below.)**

Cough 

Sore throat 

Runny nose 

Fever 

Headache 

Body aches 

Other, specify below 

_______________________________________________________________________

**45. Ask: “Can influenza illness be prevented?”**

**(Record code in box)**

Yes 1

No 2 🡪 **If the answer to 45 is 2, skip to section six**

Don’t Know/Not Sure 9 🡪 **If the answer to 45 is 9, skip to section six**

**46. Ask: “How can influenza illness be prevented?“**

**(Ask this as an open ended question. If the respondent mentions the listed prevention, write “1” for “yes” in the box below.**

**If the does not mention the listed prevention, write “2” for “no” in the box below.)**

Yes 1

No 2

Keep away from ill persons 

Wash hands frequently 

Wash hands frequently with soap 

Vaccination 

Avoid cold foods 

By taking doctor’s advice 

By maintaining cleanliness…………………. 

Other, specify below 

________________________________

Section Five: PHONE NUMBER RECORDING

| **Contact Information for Follow Up Influenza Season Calls**  **(Use during final visit to bari)**  Objectives:  -Choose respondents and phone owners within the Index Case’s household when possible  -Choose respondents and phone owners who are likely to be at the bari during the day  -Collect mobile numbers from two/three bari members who are willing to take BISTIS calls on their phone  -Ensure that the bari members who provide mobile numbers are willing to allow other bari members to speak with the BISTIS team member  -When possible, find a primary respondent that will be available to provide information for all the follow up calls | | Relationship to  to index case-patient  Self 1  Parent/Guardian 2  Other household member 3  Fellow bari member 4 |
| --- | --- | --- |
| Primary Contact 1  Phone Number:  Phone Owner’s Name:  Relationship to index case-patient:  Phone number confirmed as working? YES NO | Respondent 1  Name:  Relationship to index case-patient:___________________  Respondent 2  Name:  Relationship to index case-patient:___________________  Respondent 3  Name:  Relationship to index case-patient:___________________  Respondent 4  Name:  Relationship to index case-patient:___________________ | |
| Backup Contact 2  Phone Number:  Phone Owner’s Name:  Relationship to index case-patient:  Phone number confirmed as working? YES NO |
| Backup Contact 3  Phone Number:  Phone Owner’s Name:  Relationship to index case-patient:  Phone number confirmed as working? YES NO |

***For office use***

**Name of FRA:** ___________________________________________

**Signature of FRA**: ___________________ **Date:** ______/______/__________

**Checked by:** ______________________ **Date:** ______/______/_________

| Appendix 19: Follow Up Soap Tracking Form | | | | | | | | | | | | | |
| --- | --- | --- | --- | --- | --- | --- | --- | --- | --- | --- | --- | --- | --- |
| **Index Case Patient Unique ID:** | | | | | | **Distribution FRA (Name): ____________** | | | | **Collection FRA (Name): _____________** | | | |
| **ASK** “Can you please show me where you most often wash your hands.”  Was a specific place shown? Yes  No    - If YES, proceed to ROW 1, Column B. - If NO, **ASK** “I am going to provide your bari with a bar of soap. Where is the best place to put this bar of soap so that it is accessible to all bari members?”   Place ONE bar of soap in designated location, record notes on where soap was placed, and proceed to ROW 1, completing Columns E-G.  Record where soap was placed so the location can be remembered for second visit: _________________________________________________________________ | | | | | | | | | | | | | |
| **Information collected during Soap Distribution (Second FU Visit)**  DATE OF SOAP DISTRIBUTION (dd/mm/yyyy): _______________________ | | | | | | | | | **Soap Collection (2 days after soap distribution)**  DATE OF SOAP COLLECTION (dd/mm/yyyy): ____________ | | | | |
| A | B | C | D | E | F | | G | H | J | K | L | M | N |
|  | **OBSERVE**  Record the location of the handwashing station:  Indoors 1  Outdoors 2  No permission  to see 3  Other  (specify below) 9  (Add notes so that location can be easily identified at next visit.) | **OBSERVE**  Is water present at this specific place for hand washing?  (You must actually see water to record “yes”)  Yes 1  No 2 | **OBSERVE**  Which of the following are present at the handwashing station  (If observed, write “1” for “yes”; if not observed, write “2” for “no”):  Yes 1  No 2  🡪If bars of soap are observed, record how many  🡪 At this point, replace each bar of soap identified with a new BISTIS bar | **OBSERVE**  Distance (in steps) between handwashing station (or location of new BISTIS bar) and:  1. stove in cooking area of ICP household  2. entrance of latrine of ICP household  Record numbers below. | **RECORD** Weight of BISTIS study soap given to bari. | | **OBSERVE**  Where was the BISTIS soap placed?  Near shallow tube well 1  Near deep tube  well 2  Near piped water source 3  Other location (specify) 9 | If handwashing place was shown in initial question, **ASK**  “Is there anywhere else you wash your hands?”  Yes 1  No 2  (If YES, complete row for additional handwashing stations) | **RECORD**  Weight of BISTIS study bars still present at handwashing location  (If a BISTIS bar is not present that was weighed in column F, enter NA for “not available”) | **ASK**  “How many households used this soap?” | **ASK**  “How many people in your bari used this soap?” | **ASK**  “Was the bar soap at this location used to…”  **READ ALL CHOICES**  Yes 1  No 2 | **ASK**  “Did anyone develop a rash or other skin problem due to using this soap?”  Yes** 1  No 2 |
| **Handwashing Station #1**  **(Primary)** |   __________________________________________ |  | Bar soap __  # of bars ______  Powder soap __  Liquid soap __  Ash/Mud/Sand __  None __  Other __  (specify)_______________ | Stove:  ______steps  Latrine Entrance: ______steps  If no latrine,  check box:  | 1. g  2. g  3. g  4. g  5. g  6. g | |   _______________ |  | 1. g  2. g  3. g  4. g  5. g  6. g |  |  | Wash hands __  Bathe __  Do laundry __  Other __  (specify)_______ |  |
| **Handwashing Station #2**  **(Secondary)** |   __________________________________________ |  | Bar soap __  # of bars ______  Powder soap __  Liquid soap __  Ash/Mud/Sand __  None __  Other __  (specify)_______________ | Stove:  ______steps  Latrine Entrance: ______steps  If no latrine,  check box:  | 1. g  2. g  3. g  4. g  5. g  6. g | |   _______________ |  | 1. g  2. g  3. g  4. g  5. g  6. g |  |  | Wash hands __  Bathe __  Do laundry __  Other __  (specify)_______ |  |
| ** If any bari member develops a skin rash or other problem as a result of the intervention soap, FRA MUST CALL THE M.O. IMMEDIATELY.  **Note: Describe any disturbances to the soap placement in the household**: | | | | | | | | | | | | | |

Appendix 20: Follow Up Visit Illness Tracking Form

Name of Index case-patient: _______________________ Date of Follow Up Visit (dd/mm/yy): ____/_____/______

Respondent Unique ID # ___ ___ ___ ___ ___ ___ ___ ___ ___ ___ Number of Follow Up Visit (1, 2, o r 3): ______

FRA Code: _____________

**Index Case Household** Household Unique ID: ___ ___ ___ ___ ___ ___ ___ ___

**In the past 48 hours, has ____________ had any of the following symptoms?** Yes = 1 No = 2 Not Present = 3 Don’t Know = 9, record the number

| Name | Has bari member been present for last 48 hours? | If bari member not present, why? No longer lives there = 1  Died = 2  Other = 8, specify | Is this a new bari mem-ber? | Unique ID | | | | | | | | | | Age(yy/mm) | | Fever | Cough | Sore Throat | Difficulty Breathing | Watery Diarrhea* | Bloody Diarrhea** |
| --- | --- | --- | --- | --- | --- | --- | --- | --- | --- | --- | --- | --- | --- | --- | --- | --- | --- | --- | --- | --- | --- |
|  |  |  |  |  |  |  |  |  |  |  |  |  |  |  |  |  |  |  |  |  |  |
|  |  |  |  |  |  |  |  |  |  |  |  |  |  |  |  |  |  |  |  |  |  |
|  |  |  |  |  |  |  |  |  |  |  |  |  |  |  |  |  |  |  |  |  |  |
|  |  |  |  |  |  |  |  |  |  |  |  |  |  |  |  |  |  |  |  |  |  |
|  |  |  |  |  |  |  |  |  |  |  |  |  |  |  |  |  |  |  |  |  |  |
|  |  |  |  |  |  |  |  |  |  |  |  |  |  |  |  |  |  |  |  |  |  |
|  |  |  |  |  |  |  |  |  |  |  |  |  |  |  |  |  |  |  |  |  |  |
|  |  |  |  |  |  |  |  |  |  |  |  |  |  |  |  |  |  |  |  |  |  |
|  |  |  |  |  |  |  |  |  |  |  |  |  |  |  |  |  |  |  |  |  |  |
|  |  |  |  |  |  |  |  |  |  |  |  |  |  |  |  |  |  |  |  |  |  |
|  |  |  |  |  |  |  |  |  |  |  |  |  |  |  |  |  |  |  |  |  |  |
|  |  |  |  |  |  |  |  |  |  |  |  |  |  |  |  |  |  |  |  |  |  |
|  |  |  |  |  |  |  |  |  |  |  |  |  |  |  |  |  |  |  |  |  |  |
|  |  |  |  |  |  |  |  |  |  |  |  |  |  |  |  |  |  |  |  |  |  |
|  |  |  |  |  |  |  |  |  |  |  |  |  |  |  |  |  |  |  |  |  |  |

*Watery diarrhea is defined as 3 or more loose or watery stools in one day

** Bloody diarrhea is defined as watery diarrhea with blood in it

**Second Household**

Household Unique ID: ___ ___ ___ ___ ___ ___ ___ ___

**In the past 48 hours, has ____________ had any of the following symptoms?** Yes = 1 No = 2 Not Present = 3Don’t Know = 9, record the number

| Name | Has bari member been present for last 48 hours? | If bari member not present, why? No longer lives there = 1  Died = 2  Other = 8, specify | Is this a new bari mem-ber? | Unique ID | | | | | | | | | | Age(yy/mm) | | Fever | Cough | Sore Throat | Difficulty Breathing | Watery Diarrhea* | Bloody Diarrhea** |
| --- | --- | --- | --- | --- | --- | --- | --- | --- | --- | --- | --- | --- | --- | --- | --- | --- | --- | --- | --- | --- | --- |
|  |  |  |  |  |  |  |  |  |  |  |  |  |  |  |  |  |  |  |  |  |  |
|  |  |  |  |  |  |  |  |  |  |  |  |  |  |  |  |  |  |  |  |  |  |
|  |  |  |  |  |  |  |  |  |  |  |  |  |  |  |  |  |  |  |  |  |  |
|  |  |  |  |  |  |  |  |  |  |  |  |  |  |  |  |  |  |  |  |  |  |
|  |  |  |  |  |  |  |  |  |  |  |  |  |  |  |  |  |  |  |  |  |  |
|  |  |  |  |  |  |  |  |  |  |  |  |  |  |  |  |  |  |  |  |  |  |
|  |  |  |  |  |  |  |  |  |  |  |  |  |  |  |  |  |  |  |  |  |  |
|  |  |  |  |  |  |  |  |  |  |  |  |  |  |  |  |  |  |  |  |  |  |
|  |  |  |  |  |  |  |  |  |  |  |  |  |  |  |  |  |  |  |  |  |  |
|  |  |  |  |  |  |  |  |  |  |  |  |  |  |  |  |  |  |  |  |  |  |
|  |  |  |  |  |  |  |  |  |  |  |  |  |  |  |  |  |  |  |  |  |  |
|  |  |  |  |  |  |  |  |  |  |  |  |  |  |  |  |  |  |  |  |  |  |
|  |  |  |  |  |  |  |  |  |  |  |  |  |  |  |  |  |  |  |  |  |  |
|  |  |  |  |  |  |  |  |  |  |  |  |  |  |  |  |  |  |  |  |  |  |
|  |  |  |  |  |  |  |  |  |  |  |  |  |  |  |  |  |  |  |  |  |  |

*Watery diarrhea is defined as 3 or more loose or watery stools in one day

** Bloody diarrhea is defined as watery diarrhea with blood in it

# Appendix 21a: Follow Up Phone Call Illness Tracking Form: Ages ≥ 5 Years Old, Page 1

Name of Contact: ___________________________ Unique ID of Contact: ___ ___ ___ ___ ___ ___ ___ ___ ___ ___

Age of Contact (mmyy): _________________ Record if the contact has the following symptoms **Yes = 1 No = 2 Don’t Know = 9, record the number**

| **Number of week** | 1 | | 2 | | 3 | | 4 | | 5 | | 6 | | 7 | | 8 | | 9 | | 10 | | 11 | | 12 | |
| --- | --- | --- | --- | --- | --- | --- | --- | --- | --- | --- | --- | --- | --- | --- | --- | --- | --- | --- | --- | --- | --- | --- | --- | --- |
| **Number of Phone Call per Week** | 1 | 2 | 1 | 2 | 1 | 2 | 1 | 2 | 1 | 2 | 1 | 2 | 1 | 2 | 1 | 2 | 1 | 2 | 1 | 2 | 1 | 2 | 1 | 2 |
| **Date of phone call** |  |  |  |  |  |  |  |  |  |  |  |  |  |  |  |  |  |  |  |  |  |  |  |  |
| **Eligibility Symptom** | | | | | | | | | | | | | | | | | | | | | | | | |
| Fever |  |  |  |  |  |  |  |  |  |  |  |  |  |  |  |  |  |  |  |  |  |  |  |  |
|  |  |  |  |  |  |  |  |  |  |  |  |  |  |  |  |  |  |  |  |  |  |  |  |  |
| **Other Symptoms** | | | | | | | | | | | | | | | | | | | | | | | | |
| Cough |  |  |  |  |  |  |  |  |  |  |  |  |  |  |  |  |  |  |  |  |  |  |  |  |
| Sore Throat |  |  |  |  |  |  |  |  |  |  |  |  |  |  |  |  |  |  |  |  |  |  |  |  |
| Difficulty Breathing |  |  |  |  |  |  |  |  |  |  |  |  |  |  |  |  |  |  |  |  |  |  |  |  |
| Watery Diarrhea* |  |  |  |  |  |  |  |  |  |  |  |  |  |  |  |  |  |  |  |  |  |  |  |  |
| Bloody Diarrhea** |  |  |  |  |  |  |  |  |  |  |  |  |  |  |  |  |  |  |  |  |  |  |  |  |
|  |  |  |  |  |  |  |  |  |  |  |  |  |  |  |  |  |  |  |  |  |  |  |  |  |
| **Danger Signs (Refer to JIMCH)** | | | | | | | | | | | | | | | | | | | | | | | | |
| Cyanosis |  |  |  |  |  |  |  |  |  |  |  |  |  |  |  |  |  |  |  |  |  |  |  |  |
| Severe respiratory distress |  |  |  |  |  |  |  |  |  |  |  |  |  |  |  |  |  |  |  |  |  |  |  |  |
| Convulsions |  |  |  |  |  |  |  |  |  |  |  |  |  |  |  |  |  |  |  |  |  |  |  |  |
| Altered mental status |  |  |  |  |  |  |  |  |  |  |  |  |  |  |  |  |  |  |  |  |  |  |  |  |
|  |  |  |  |  |  |  |  |  |  |  |  |  |  |  |  |  |  |  |  |  |  |  |  |  |
| **Actions** | | | | | | | | | | | | | | | | | | | | | | | | |
| Refer to MT for sample |  |  |  |  |  |  |  |  |  |  |  |  |  |  |  |  |  |  |  |  |  |  |  |  |
| Date of Sample Collection |  |  |  |  |  |  |  |  |  |  |  |  |  |  |  |  |  |  |  |  |  |  |  |  |
| MO/MT Code |  |  |  |  |  |  |  |  |  |  |  |  |  |  |  |  |  |  |  |  |  |  |  |  |
| Hospitalized |  |  |  |  |  |  |  |  |  |  |  |  |  |  |  |  |  |  |  |  |  |  |  |  |
|  |  |  |  |  |  |  |  |  |  |  |  |  |  |  |  |  |  |  |  |  |  |  |  |  |
| **FRA Code** |  |  |  |  |  |  |  |  |  |  |  |  |  |  |  |  |  |  |  |  |  |  |  |  |

*Watery diarrhea is defined as 3 or more loose or watery stools in one day

** Bloody diarrhea is defined as watery diarrhea with blood in it

# Appendix 21a: Follow Up Phone Call Illness Tracking Form: Ages ≥ 5 Years Old, Page 2

Name of Contact: ___________________________ Unique ID of Contact: ___ ___ ___ ___ ___ ___ ___ ___ ___ ___

Age of Contact (mmyy): _________________ Symptom information available for this contact? _________ **Yes = 1 No = 2 Don’t Know = 9, record the number**

| **Number of week** | 13 | | 14 | | 15 | | 16 | | 17 | | 18 | | 19 | | 20 | | 21 | | 22 | | 23 | | 24 | |
| --- | --- | --- | --- | --- | --- | --- | --- | --- | --- | --- | --- | --- | --- | --- | --- | --- | --- | --- | --- | --- | --- | --- | --- | --- |
| **Number of Phone Call per Week** | 1 | 2 | 1 | 2 | 1 | 2 | 1 | 2 | 1 | 2 | 1 | 2 | 1 | 2 | 1 | 2 | 1 | 2 | 1 | 2 | 1 | 2 | 1 | 2 |
| **Date of phone call** |  |  |  |  |  |  |  |  |  |  |  |  |  |  |  |  |  |  |  |  |  |  |  |  |
| **Eligibility Symptom** | | | | | | | | | | | | | | | | | | | | | | | | |
| Fever |  |  |  |  |  |  |  |  |  |  |  |  |  |  |  |  |  |  |  |  |  |  |  |  |
|  |  |  |  |  |  |  |  |  |  |  |  |  |  |  |  |  |  |  |  |  |  |  |  |  |
| **Other Symptoms** | | | | | | | | | | | | | | | | | | | | | | | | |
| Cough |  |  |  |  |  |  |  |  |  |  |  |  |  |  |  |  |  |  |  |  |  |  |  |  |
| Sore Throat |  |  |  |  |  |  |  |  |  |  |  |  |  |  |  |  |  |  |  |  |  |  |  |  |
| Difficulty Breathing |  |  |  |  |  |  |  |  |  |  |  |  |  |  |  |  |  |  |  |  |  |  |  |  |
| Watery Diarrhea* |  |  |  |  |  |  |  |  |  |  |  |  |  |  |  |  |  |  |  |  |  |  |  |  |
| Bloody Diarrhea** |  |  |  |  |  |  |  |  |  |  |  |  |  |  |  |  |  |  |  |  |  |  |  |  |
|  |  |  |  |  |  |  |  |  |  |  |  |  |  |  |  |  |  |  |  |  |  |  |  |  |
| **Danger Signs (Refer to JIMCH)** | | | | | | | | | | | | | | | | | | | | | | | | |
| Cyanosis |  |  |  |  |  |  |  |  |  |  |  |  |  |  |  |  |  |  |  |  |  |  |  |  |
| Severe respiratory distress |  |  |  |  |  |  |  |  |  |  |  |  |  |  |  |  |  |  |  |  |  |  |  |  |
| Convulsions |  |  |  |  |  |  |  |  |  |  |  |  |  |  |  |  |  |  |  |  |  |  |  |  |
| Altered mental status |  |  |  |  |  |  |  |  |  |  |  |  |  |  |  |  |  |  |  |  |  |  |  |  |
|  |  |  |  |  |  |  |  |  |  |  |  |  |  |  |  |  |  |  |  |  |  |  |  |  |
| **Actions** | | | | | | | | | | | | | | | | | | | | | | | | |
| Refer to MT for sample |  |  |  |  |  |  |  |  |  |  |  |  |  |  |  |  |  |  |  |  |  |  |  |  |
| Date of Sample Collection |  |  |  |  |  |  |  |  |  |  |  |  |  |  |  |  |  |  |  |  |  |  |  |  |
| MO/MT Code |  |  |  |  |  |  |  |  |  |  |  |  |  |  |  |  |  |  |  |  |  |  |  |  |
| Hospitalized |  |  |  |  |  |  |  |  |  |  |  |  |  |  |  |  |  |  |  |  |  |  |  |  |
|  |  |  |  |  |  |  |  |  |  |  |  |  |  |  |  |  |  |  |  |  |  |  |  |  |
| **FRA Code** |  |  |  |  |  |  |  |  |  |  |  |  |  |  |  |  |  |  |  |  |  |  |  |  |

*Watery diarrhea is defined as 3 or more loose or watery stools in one day

** Bloody diarrhea is defined as watery diarrhea with blood in it

**G¨v‡cwÛ· - 16 t m¤§wZcÎ t d‡jvAvc ¯UvwW‡Z evox Aš—©fy³Kib|**

cÖUKj b¤^i t 2009 -004

**cÖUKj UvB‡Uj (**M‡elbvi **bvg) t-**

wcÖ‡fbkb Ad †m‡KÛvwi UªvÝwgkb Ae wnDg¨vb Bbd¬z‡qÁv evB cÖgwUs n¨vÛ Iqvwks evD †mvct w` evsjv‡`k BbUvivckb Ae †m‡KÛvwi UªvÛwgkb Ae Bbd¬z‡qÁv ÷vwW (BISTIS) d‡jvv Avc ÷vwW-2010|

mvevb w`‡q nvZ †avqvi gva¨‡g †ivM cieZx© Bbd¬z‡qÁv msµgb cÖwZ‡iva msµvš— M‡elbv evsjv‡`k (wem&wUm&), d‡jv Avc M‡elbv-2010|

M‡el‡Ki **bvg** t Wvt GWyqv‡Wv© AvwRR †evgMvUv©bvi |

cÖwZôvb t Avš—©RvwZK D`ivgq M‡elYv †K›`ª, evsjv‡`k (AvB.wm.wW.wW.Avi,we)

fywgKvt

2009 mv‡j Avcbvi evox Bbd¬z‡qÁv msµvš— GKwU M‡elbvq Ask MÖnb K‡iwQj| M‡elbvwU AvB.wm.wW.wW.Avi,we, wm.wW.wm I evdv‡jv wek¦we`¨vj‡qi (Av‡gwiKvi GKvwU wek¦we`¨vjq)i M‡elKiv cwiPvjbv K‡ib| eZ©gv‡b Avgiv GLv‡b G‡mwQ M‡elbvi wØZxq Ask wb‡q Avcbvi mv‡_ K_v ej‡Z| Avgiv Lvbv m`m †`i wewfbœ Af¨vm I evox m`m¨‡` Amy¯’Zvi AwfÁZv m¤ú‡K© eyS‡Z †Póv eie|

M‡elbvi D‡Ïk¨ t

Avgiv eyS‡Z †Póv KiwQ wKfv‡e mg‡qi mv‡_ Lvbv m`m¨‡`i Af¨vm e`jvq Ges wKfv‡e Zv wewfbœ Amy¯’Zv‡K cÖfvweZ K‡i, †hgb WvBwiqv Ges k¦vmZ‡Z&ªi Amy¯’Zv|

**‡Kb g‡bvwbZ Kiv n‡q‡Q t**

Avcbvi evox MZ ermi Avgv‡`i M‡elbvi cÖ_g As‡k AskMÖnb K‡iwQj| wØZxq Ask ïaygvÎ †mB evox ¸‡jv DciB n‡e hviv cÖ_g As‡k Aš—©fy³ n‡qwQj|

Avcbvi evox m`m¨‡`i KvQ †_‡K wK Avkv Kiv n‡e?

Avcwb hw` Avcbvi evox Aš—f ©y³ Ki‡Z ivRx _v‡Kb t

Avwg AvMvgx 3-4 gv‡mi g‡a¨ Av‡iv wZb evi Avcbvi evox cwi`k©b Kie|

`ywU cwi`k‡b©i mgq,

Avwg ‡h e¨w³ 2009 (-------------------gv‡m) Rwni“j Bmjvg †gwW‡Kj K‡jR nvmcvZv‡j Bbd¬z‡qÁv ev Ab¨ †Kvb k¦vmZ‡š¿i Amy¯’Zv wb‡q wM‡qwQ‡jb Zvi mv‡_ Avwg K_v eje| hw` †mB e¨vw³ 18 eQ‡ii Kg nq Zvn‡j Avwg Zvi Awffve‡Ki mv‡_ K_v eje| Avwg Lvbv m`m¨†`i wewfbœ Af¨vm m¤ú‡K© wKQz cÖkœ Kie| Avwg cÖwZ cwi`k©‡bi mgq Avcbvi evoxi wKQy `ª“Z ch©‡e¶b Kie| GKwU cwi`k©‡b Avcbiv evoxi Lvbv m`m¨‡`i Af¨vm ¸‡jv ch©‡e¶‡bi Rb¨ Avwg Avcbvi evoxi DVv‡b cÖvq 90 wgwbU eme|

GB cwi`kb© ¸‡jvi mgq Avwg evox m`m¨‡`i MZ 48 N›Uvi g‡a¨ R¡i, k¦vmZ‡š¿i Amy¯’Zv ev cwicvKZ‡š¿i Amy¯’Zvi †Kvb j¶b wQj wK bv Zv wRÁvmv Kie|

(hw` evox‡Z Smart Soap e¨eüZ nq Zvn‡j wb¤§ wjwLZ Aby‡”Q`wU †Rv‡i c‡o ïbv‡Z n‡e|)

GKwU cwi`k‡b©i mgq Avwg Avcbvi evox‡Z GKwU we‡kl ai‡bi mvevb †`e wZb w`b e¨env‡ii Rb¨ | Zvi ci Avwg Zv Avevi 2 w`b ci mvevbwU msMÖn Kivi Rb¨ Avme| we‡kl jvBdeq mvevbwU A‡bKUv mvavib jvBdeq mvev‡bi ‡gZB hv evRv‡i cvIqv hvq Ges GKB ai‡bi myweav †`q| Avcwwb m¤¢eZ we‡kl mvevb I mvaviY mvev‡bi cv_K¨© ej‡Z cvi‡eb bv| we‡kl jvBdeq mvevbU †hfv‡e mvevb Avcbvi evox‡Z e¨eüZ nq ‡mfv‡e e¨envi Ki‡Z n‡e| we‡kl jvBdeq mvevb hv Avgiv Avcbv‡K w`w”Q Zvi i‡q‡Q GKwU ‰e`~¨wZK hš¿ hv, mvevb e¨envi m¤ú‡K© Z_¨ msMÖn K‡i| †h mKj gvbyl jvBdeq mvev‡bi cÖwZ ms‡e`bkxj, jvBdeq e¨env‡i Z¡‡K cÖwZwµqvi AwfÁv i‡q‡Q, Zviv Avgv‡`i †`qv jvBdeq mvev‡bI Abyiƒc AwfÁZv †c‡Z cv‡ib Ges e¨envi Kiv DwPZ n‡e bv|

Z„Zxq cwi`k‡b©, Avcbvi evoxi `yÕRb m`m¨‡K Avwg Zv‡`i †gvevBj b¤^i Avgv‡K w`‡Z eje| Zvici Zv‡K Avwg 6 gvm chšÍ©, mßv‡n `yBevi †dvb K‡i evox m`¨‡m¨‡`i Kv‡iv R¡i Av‡Q wKbv Zv wbb©q Kie|

Z…Zxq cwi`k‡b©i c‡i, hv‡`i R¡i i‡q‡Q, Avgiv Zv‡`i Kv‡Q bvK I Mjv †_‡K bgybv wb‡Z PvBe, Avgv‡`i GKRb cÖwk¶Z M‡elbv Kgx© GKwU Zzjv c¨vuPv‡bv KvwV w`‡q bvK †_‡K Ges Ab¨ GKwU KvwV w`‡q Mjv †_‡K bgybv msMÖn Ki‡e|

SzuwK I myweav t

Avcbvi evox cwi`k‡b©i cØwZwU Avcbvi Kv‡Q A¯^w¯—Ki n‡Z cv‡i | Z‡e Avgiv GB cwi`k‡b©i Rb¨ Avcwb ev Avcbvi cwiev‡ii †Kvb ¶wZ Avk¼v Kwi bv|

(hw` evox‡Z Smart Soap e¨eüZ nq Zvn‡j wb¤§ wjwLZ Aby‡”Q`wU †Rv‡i c‡o ïbv‡Z n‡e|)

evRv‡i cÖvß jvBdeq mvev‡b hv‡`i Z¡‡K cÖwZwµqv nq, we‡kl jvBdeq mvevbI GKB ai‡bi cÖwZwµqv m„wó Ki‡Z cv‡i| †m mKj e¨vw³‡`i Avgv‡`i †`qv jvBdeq mvevb e¨envi Kiv DwPZ n‡e bv | †h jvBdeq mvevbwU Avgiv Avcbv‡K †`e Zvi †fZi GKwU e¨vUvix Av‡Q| H e¨vUvixwU Nwo ev K¨v‡giv‡Z e¨eüZ e¨vUvixi gZ| e¨vUvixwU mvev‡bi †fZ‡i GKwU ev‡· myiw¶Z Av‡Q| GUv LyeB AcÖZ¨vwkZ †h Avcwb e¨vUvixwU †`L‡Z cv‡eb| Avcwb hw` e¨vUvixi ev·wU †`L‡Z I cvb ZeyI Avcwb ¯^vfvweK fv‡eB mvevb e¨envi K‡i †h‡Z cv‡ib| `qv K‡i g‡b ivL‡Z †Póv Ki‡eb, ‡Kvb w`b Avcwb mvev‡bi †fZi †_‡K ev·wU †ewi‡q Avm‡Z ïi“ K‡i‡Q e‡j †`‡L‡Qb| Ab¨ mvev‡bi gZB `qv K‡i mvevbwU ev”Pv‡`i gy‡L w`‡Z w`‡eb bv|

GB M‡elbvq Ask MÖnbKvix mKj evox webvg~‡j¨ mvevb cvIqvi myweav cv‡eb| GQvov Avi †Kvb Zvr¶wbK myweav †bB| Z‡e GB M‡elbv Avgv‡`i eyS‡Z mvnvh¨ Ki‡e †h, nvZ †avqvi AvPiY mg‡qi mv‡_ e`‡j †h‡Z cv‡i wKbv| GUv Avgv‡`i Av‡iv eyS‡Z mvnvh¨ Ki‡e †h, nvZ †avqvi AvPib k¦vmZ‡š¿i Amy¯’Zv Ges Wvqwiqvi mv‡_ RwoZ wKbv|

Z…Zxq cwi`k‡b©i c‡i, hv‡`i R¡i i‡q‡Q, Avgiv Zv‡`i Kv‡Q bvK I Mjv †_‡K bgybv wb‡Z PvBe, Avgv‡`i GKRb cÖwk¶Z M‡elbv Kgx© GKwU Zzjv c¨vuPv‡bv KvwV w`‡q bvK †_‡K Ges Ab¨ GKwU KvwV w`‡q Mjv †_‡K bgybv msMÖn Ki‡e| GUv A¯^w¯—Ki n‡Z cv‡i| GQvov Rvbv g‡Z Gi Avi †Kvb SzwK †bB|

GB cix¶vi djvdj j¶bhy³ e¨vw³i wPwKrmvi †Kvb ZviZg¨ Ki‡e bv Ges †mB Rb¨ j¶bhy³ e¨w³‡K cix¶vi †Kvb djvdjI ‡`qv n‡e bv|

‡MvcbxqZv t

mg¯— Z_¨ hv Avgiv Avcbvi evox m`m¨‡`i KvQ †_‡K msMÖn Kie Zv †Mvcb ivLv n‡e| Avgiv me DcvË GKwU ZvjveØ Avjgvix‡Z ivLe| Avgiv Avcivi evoxi †Kvb Z_¨ GB M‡elbvi Kv‡R mshy³ bq Ggb KvD‡K †`e bv|

Z‡_¨i fwel¨Z e¨envi t

Avgiv †h mg¯— Z_¨ msMÖn K‡iwQ Zv hw` fwel¨‡Z Ab¨ †Kvb M‡elK‡`i e¨envi Kiv cÖ‡qvRb nq, Avgiv †Kvb e¨vw³MZ Z_¨ mieivn Kie bv Ges KwVb †MvcbxqZv cvjb Kie|

AskMÖnb bv Kivi AwaKvi I AskMÖnb †_‡K m‡i hvIqv t

Avcwb Avcbvi evox‡K GB M‡elbvq AskMÖnb Ki‡Z w`‡Z cv‡ib A_ev bvI cv‡ib | Avcwb †h †Kvb mgq Ask MÖnb Ki‡Z Am¤§wZ Rvbv‡Z cv‡ib| Avcwb Avcbvi evoxI †h †Kvb mgq M‡elbv †_‡K mwi‡q wb‡Z cv‡ib| AskMÖn‡b Am¤§wZ ev M‡elbv †_‡K m‡i hvIqvi Rb¨ Avcbvi /evoxi m`m¨‡`i †Kvb Rwigvbv A_ev nvmcvZv‡ji ¯^vfvweK myweav †_‡K ewÂZ n‡eb bv | hw` Avcwb Avcbvi evox M‡elbvq Aš—©fy³ bvI Kivb ZeyI Avcbvi evoxi mKj m`m¨ nvmcvZv‡Ki ¯^vfvweK mg¯— myweav cv‡eb| Avcbvi evoxi mKj m`m¨ Avjv`vfv‡e Ask MÖnb Kiv ev bv Kiv †K ‡e‡Q wb‡Z cv‡ib, Ges †h †Kvb mgq M‡elbv †_‡K m‡i hvIqv‡KI †e‡Q wb‡Z cv‡i|

¶wZc~ib bxwZt

GB M‡elbvq AskMÖn‡bi Rb¨ Avcwb ev Avcbvi evoxi †Kvb LiP †bB | ïaygvÎ webvg~‡j¨ mvevb cvIqv Qvov GB M‡elbvq _vKvi Rb¨ Avcwb Avi †Kvb ¶wZc~ib cv‡eb bv|

‡hvMv‡hv‡Mi e¨vw³ t

GB cÖwµqv PjvKvjxb Avcbvi †Kvb cÖkœ _vK‡j †h †Kvb mgq Ki‡Z cv‡ib| hw` Avcbvi M‡elbv m¤ú‡K© †Kvb AwZwi³ cÖkœ _v‡K Avcwb †hvMv‡hvM Ki‡Z cv‡ib t

Wvt GWyqv‡Wv© AvwRR †evgMvUv©bvi, †cÖvMÖvg Ab Bb‡dKkvm wWwRR G¨vÛ †fKwmb mvB‡Ým (wc,AvvB.wW.wf.Gm) AvB.wm.wW.wW.Avi.we, gnvLvjx, XvKv 1212, †dvb t 8860523-32 # 2500|

GB M‡elbv Ask wnmv‡e Avcbvi AwaKvi m¤ú‡K© hw` Avcbvi †Kvb cÖkœ _v‡K A_ev hw` M‡elbvi Rb¨ Avcbvi †Kvb ¶wZ n‡q‡Q e‡j g‡b nq Zvn‡j †hvMv‡hvM Ki“b t

wgt Gg.G.mvjvg, wimvP© G¨Û cÖ‡R± mv‡cv©U wWcvU©‡g›U (Avi.wc.Gm.wW) AvB.wm.wW.wW.Avi.we, gnvLvjx, XvKv– 1212, †dvb t 9886489, # 01711428989|

hw` Avcwb Avcbvi evox Avgv‡`i M‡elYvq Aš—f©y³ Ki‡Z Pvb, `qv K‡i Avcbii ¯^v¶i w`‡q A_ev Avcbvi evgnv‡Zi ey‡ov Av½y‡ji Qvc w`‡q Zv wb‡`©k Ki“b|

Avcbvi mn‡hvMxZvi Rb¨ ab¨ev`|

___________________________________________ ___________________

¯^v¶i/ AskMÖnbKvixi evg nv‡Zi e„Øv½yjxi Qvc ZvwiL

___________________________________________ ___________________

ïkªlvKvix / AwffveK Gi evg nv‡Zi e„Øv½yjxi Qvc ZvwiL

___________________________________________ ___________________

¯^v¶xi ¯^v¶vi / A_ev evg nv‡Zi e„Øv½yjxi Qvc ZvwiL

___________________________________________ ___________________

cÖavb M‡elK ev Zvi cÖwZwbwai ¯^v¶i ZvwiL

**wemwUm& t d‡jv Avc mv‡f© dig**

1. **DËi`vZv‡K wRÁvmv Kiyb t DËi`vZvi bvgt .............................................................................**
2. **wKfv‡e DËi`vZv Bb‡W· †Km Gi mv‡_ m¤úwK©Z?**

**(e‡· †KvW wjL~b)**

**wbR .......................................... 1**

**AwffveK .................................. 2**

**evoxi m`m¨ ….............................. 3**

1. **DËi`vZvi (Bb‡W· †Km/ AwffveK / evoxi m`m¨) BDwbK AvB. wW (BwbDgv‡ikY dig †_‡K †bIqv)t**

**.............................................................................**

1. **Bb‡W· †Km †ivMxi BDwbK AvB.wWt ……… ............................................................**
2. **d‡jv Avc wfwRU b¤^i (1, 2 A_ev 3) t ......................................................**
3. **d‡jv Avc wfwR‡Ui ZvwiL t (w`b/gvm/eQi) ......../......../........**
4. **FRA †KvW t ...................................................**

wefvM GK t nvZ †avqvi ¯’vb m¤úwK©Z cÖkœvejx

**DËi`vZv†K wRÁvmv Ki“b t Avcwb wK `qv K‡i Avgv‡K †`Lv‡Z cv‡ib mPivPi Avcwb †Kv_vq nvZ ay‡q _v‡Kb ?**

1. **wjLyb: nvZ ‡avqvi Rb¨ mywbw`ªó †Kvb RvqMv Av‡Q wK?**

**(e‡· b¤^i wjLyb)**

**n¨vu-------------------- 1**

**bv--------------------- 2**

***hw` 8 Gi DËi bv nq Zv n‡j 12 b¤^i cÖ‡kœ hvb|***

1. **wemwUm Gi B›Uvi‡fbkb n¨vÛIqvwks K‡›UBbviwU evoxi nvZ †avqvi ¯’vbwU‡Z i‡q‡Q wK?**

**(e‡· †KvW wjL~b)**

**n¨vu-------------------- 1**

**bv--------------------- 2**

**cÖ‡hvR¨ bq ÑÑÑÑÑÑÑ 8**

1. **ch©‡e¶Y t evoxi nvZ †avqvi ¯’vbwU‡Z cvwb Av‡Q wK?**

**(e‡· †KvW wjLyb n¨vu wjL‡Z n‡j Avcbv‡K Aek¨B cvwb Av‡Q wK bv Zv wbwðZ n‡Z n‡e)**

**n¨vu-------------------- 1**

**bv--------------------- 2**

1. **ch©‡e¶Y t nvZ †avqvi ¯’v‡b wb‡¤§i †Kvb Dcv`vb ¸‡jv Av‡Q? cÖwZwU Dcv`vb Avjv`v fv‡e j¶¨ Ki“b `qv K‡i gy‡L D”Pvib Ki‡eb bv| (ZvwjKvf~³ Dcv`vb ¸‡jv ch©‡¶Y Ki‡j Òn¨vuÓ Gi Rb¨ bx‡P cÖ`Ë ev‡Kª Ò1Ó wjLyb| ZvwjKv f~³ Dcv`vb ¸‡jv ch©‡e¶Y bv Ki‡j ÒbvÓ Gi Rb¨ bx‡P cÖ`Ë ev‡Kª Ò2Ó wjLyb )**

**evi mvevb / mvaviY mvevb ÑÑÑÑÑÑÑÑÑÑÑÑÑÑÑÑÑÑÑÑÑÑÑÑÑÑÑ**

**¸ov mvevb ÑÑÑÑÑÑÑÑÑÑÑÑÑÑÑÑÑÑÑÑÑÑÑÑÑÑÑ**

**Zij mvevb ÑÑÑÑÑÑÑÑÑÑÑÑÑÑÑÑÑÑÑÑÑÑÑÑÑÑÑ**

**QvB ÑÑÑÑÑÑÑÑÑÑÑÑÑÑÑÑÑÑÑÑÑÑÑÑÑÑÑ**

**gvwU / evwj ÑÑÑÑÑÑÑÑÑÑÑÑÑÑÑÑÑÑÑÑÑÑÑÑÑÑÑ**

**Ab¨vb¨ cwi¯‹viK Dcv`vb (my wbw`©ó Kiƒb) ÑÑÑÑÑÑÑÑÑÑÑÑÑÑÑÑÑÑÑ**

**12. wRÁvmv Ki“b t nvZ‡avqvi Rb¨ Avcbvi Lvbv‡Z e¨eüZ nq &Ggb †Kvb Dcv`vb Avgv‡K †`Lv‡Z cv‡ib?**

**(hw` DËi`vZv GK wgwb‡Ui †fZ‡i Avcbv‡K †Kvb Dcv`vb †`Lv‡Z cv‡i Zvn‡j Òn¨vuÓ Gi Rb¨ bx‡P cÖ`Ë e‡· Ò1Ó wjLyb| hw` Avcwb GK wgwb‡Ui †fZ‡i wb‡¤§ ewb©Z †Kvb Dcv`vb ch©‡e¶b bv K‡ib Zvn‡j ÒbvÒ Gi Rb¨ Ò2Ó bx‡Pi cÖ`Ë e‡· Ò2Ó wjLyb| Avcwb DËi`vZv‡K ïa~gvÎ †`Lv‡Z ej‡eb , `qv K‡i gy‡L D”Pvib Ki‡eb bv)**

**evi mvevb / mvaviY mvevb ÑÑÑÑÑÑÑÑÑÑÑÑÑÑÑÑÑÑÑÑÑÑÑÑÑÑÑ**

**¸ov mvevb ÑÑÑÑÑÑÑÑÑÑÑÑÑÑÑÑÑÑÑÑÑÑÑÑÑÑÑ**

**Zij mvevb ÑÑÑÑÑÑÑÑÑÑÑÑÑÑÑÑÑÑÑÑÑÑÑÑÑÑÑ**

**QvB ÑÑÑÑÑÑÑÑÑÑÑÑÑÑÑÑÑÑÑÑÑÑÑÑÑÑÑ**

**gvwU / evwj ÑÑÑÑÑÑÑÑÑÑÑÑÑÑÑÑÑÑÑÑÑÑÑÑÑÑÑ**

**Ab¨vb¨ (wbw`©ó Kiƒb) ÑÑÑÑÑÑÑÑÑÑÑÑÑÑÑÑÑÑÑÑÑÑÑÑÑÑÑ**

**13. ch©‡e¶Y t BbUvi‡fbkb wKD KvW© ¸‡jv evox‡Z †`Lv hv‡”Q wK? (e‡· †KvW wjL~b)**

**n¨vu-------------------- 1**

**bv--------------------- 2**

**DËi `vZv‡K wemwUm B›Uvi‡fbkb Gi GKwU wKD KvW© D`vinib wnmv‡e †`Lvb|**

**14| DËi `vZv‡K wRÁvm Kiæb ÒMZ** ermi  **GB iKg wKD KvW© Avcbv‡K ‡KD w`‡q‡Q wK?**

**n¨vu-------------------- 1**

**bv--------------------- 2**

***hw` 14 Gi DËi bv nq Zv n‡j* w*efvM - `yB G hvb|***

**15| DËi `vZv‡K wRÁvm Kiæb Ò Avcwb wK wKD KvW© ¸wj ‡`Lv†Z cv‡ib?**

**n¨vu-------------------- 1**

**bv--------------------- 2**

**wefvM - `yB t ÷ªvKPviW Aemvi‡fkb**

**16.GBwU wK evox‡Z 1g d‡jv Avc ? (e‡· †KvW wjL~b)**

**n¨vu-------------------- 1**

**bv--------------------- 2**

***hw` 16 Gi DËi bv nq Zvn‡j wefvM -3 G hvb|***

17. †Kvb **Lvbv** ch©‡e¶b Kiv n†e : 

**ICP Gi Lvbv...................1**

evox cav**†**bi **Lvbv...............2**

**2**q **Lvbv...........................3**

**17. DËi `vZvi AvB.wW** # 

18. cÖv_wgK cwiPhv©Kvixi/Awffv‡Ki bvg (hw` **ICP** < 18 ermi nq) : **______________________**

**19.** ch©‡e¶bKyZ **Lvbv‡Z †gvU m`m¨ msL¨v:** 

**20. mv¶vrKvi MÖn‡bi mgq** ch©‡e¶bKZ **Lvbv‡Z Dcw¯’Z m`‡mi msL¨v (ICP Gi Lvbv‡Z ICP mn hw` Dcw¯’Z _v‡K) t**

< 5 ermi wkï  > 5 ermi < 18 erm‡ii wkï  > 18 ermi e¨vw³ : 

AvwgGLb †_‡K Av‡iv AvovB N›Uv Avcbv‡`i mv‡_ _vK‡Z PvBe| Avwg ïay ch©‡e¶b Kie Ges †Kvb evav cÖ`vb Kie bv| hw` Zv‡Z †Kvb Amyweav bv nq Zvn‡j Avcwb Avcbvi mvavib ‰`bw›`b KvR Pvwj‡q †h‡Z cv‡ib | cÖvq †`o N›Uv ci Avwg Avcbvi evoxi wKQz gvbyl‡K wKQz cÖkœ Kie|

21. ch©‡e¶b ïi“b mgq (24 N›Uv di‡gU)t N›Uv / wgwbU : :

22. ch©‡e¶b †k‡li mgq (24 N›Uv di‡gU)t N›Uv / wgwbU : :

gš—e¨ t ______________________________________________________________

__________________________________________________________________________

__________________________________________________________________________

_________________________________________________________________________

**wefvM `yB t nvZ †avqvi my‡hvM I AvPib ch©‡e¶K t**

| **jvBb b¤^i** | **NUbv**  1.Lvevi †Zix / cwi‡ek‡bi Av‡M ............................. 1  2.LvIqvi Av‡M ..... 2  3.cvqLvbvi ci wkï‡K / b¨vwc cwi¯Kvi Kivi ci ........ 3  4.cvqLvbv e¨env‡ii ci ...........4  5.Kvwk/nvwPi ci ......5  6.bvK Svivi ci ..... 6  7.ev”Pvi bvK Svivi ci .......7  8. nv‡Z gqjv jvM‡j .....10  9.Ab¨vb¨ wbw`ó© Kiƒb ............8 | **ch©‡e¶‡bi mgq**  24 N›Uv di‡gU  N›Uv : wg: | **evoxi m`m¨**  < 5 ermi wkï - 1  > 5 ermi < 18 ermi wkï - 2  > 18 ermi e¨vw³ - 3 | **evoxi m`‡m¨i †RÛvi:**  **cyi“l – - 1**  **gwnjv - 2** | **evox m`m¨ wK nvZ ay‡q‡Q?**  *n¨vu -- 1*  *bv -- 2*  *Rvwb bv -- 9*  *hw` 2 A_ev 9 nq Zvn‡j cieZx© NUbvq hvb|* | **nvZ †avqvi ¯’vb**  ivbœvN‡i -- 1  cvqLvbvq -- 2  cÖv_wgK nvZ †avqvi ¯’v‡b -- 3  cieZx© nvZ †avqvi ¯’v‡b -- 4  DVv‡b Ab¨ †Kv_vI nvZ †avqvi ¯’v‡b bq, -- 6  ivbœvN‡I Ab¨ †Kv_vI nvZ †avqvi ¯’v‡b bq -- 7  Ab¨vb¨, wbw`©wó Ki“b -- 8 | **`yB nvZ wK ay‡q‡Q?**  n¨vu -- 1  bv -- 2  Rvwb bv -- 3 | **nvZ †avqvi Dcv`vb t**  evi mvevb ----- 1  QvB --------- 2  gvwU ------- 3  ïay cvwb ----- 4  Ab¨vb¨ wbwÏ©ó Kiƒb ------ 8  cÖhR¨ bq ------ 9 | **gš—e¨** |
| --- | --- | --- | --- | --- | --- | --- | --- | --- | --- |
| 01 |  |  |  |  |  |  |  |  |  |
| 02 |  |  |  |  |  |  |  |  |  |
| 03 |  |  |  |  |  |  |  |  |  |
| 04 |  |  |  |  |  |  |  |  |  |
| 05 |  |  |  |  |  |  |  |  |  |
| 06 |  |  |  |  |  |  |  |  |  |
| 07 |  |  |  |  |  |  |  |  |  |
| 08 |  |  |  |  |  |  |  |  |  |
| 09 |  |  |  |  |  |  |  |  |  |
| 10 |  |  |  |  |  |  |  |  |  |

| **jvBb b¤^i** | **NUbv**  1.Lvevi †Zix / cwi‡ek‡bi Av‡M ............................. 1  2.LvIqvi Av‡M ..... 2  3.cvqLvbvi ci wkï‡K / b¨vwc cwi¯Kvi Kivi ci ........ 3  4.cvqLvbv e¨env‡ii ci ...........4  5.Kvwk/nvwPi ci ......5  6.bvK Svivi ci ..... 6  7.ev”Pvi bvK Svivi ci .......7  8.Ab¨vb¨ wbw`ó© Kiƒb ............99 | **ch©‡e¶‡bi mgq**  24 N›Uv di‡gU  N›Uv : wg: | **evoxi m`m¨**  < 5 ermi wkï - 1  > 5 ermi < 18 ermi wkï - 2  > 18 ermi e¨vw³ - 3 | **evoxi m`‡m¨i †RÛvi:**  **gwnjv - 0**  **cyi“l – - 1** | **evox m`m¨ wK nvZ ay‡q‡Q?**  *n¨vu -- 1*  *bv -- 0*  *Rvwb bv -- 9*  *hw` 0 A_ev 9 nq Zvn‡j cieZx© NUbvq hvb|* | **nvZ †avqvi ¯’vb**  ivbœvN‡i -- 1  cvqLvbvq -- 2  cÖv_wgK nvZ †avqvi ¯’v‡b -- 4  evjwZ / Ab¨ wKQz -- 5  DVv‡b Ab¨ †Kv_vI nvZ †avqvi ¯’v‡b bq, -- 6  ivbœvN‡I Ab¨ †Kv_vI nvZ †avqvi ¯’v‡b bq -- 7  Ab¨vb¨, wbw`©wó Ki“b -- 8 | **`yB nvZ wK ay‡q‡Q?**  n¨vu -- 1  bv -- 2  Rvwb bv -- 3  cÖhR¨ bq – -- 0 | **nvZ †avqvi Dcv`vb t**  evi mvevb ----- 1  QvB --------- 2  gvwU ------- 3  ïay cvwb ----- 4  Ab¨vb¨ wbwÏ©ó Kiƒb ------ 8  cÖhR¨ bq ------ 9 | **gš—e¨** |
| --- | --- | --- | --- | --- | --- | --- | --- | --- | --- |
| 11 |  |  |  |  |  |  |  |  |  |
| 12 |  |  |  |  |  |  |  |  |  |
| 13 |  |  |  |  |  |  |  |  |  |
| 14 |  |  |  |  |  |  |  |  |  |
| 15 |  |  |  |  |  |  |  |  |  |
| 16 |  |  |  |  |  |  |  |  |  |
| 17 |  |  |  |  |  |  |  |  |  |
| 18 |  |  |  |  |  |  |  |  |  |
| 19 |  |  |  |  |  |  |  |  |  |
| 20 |  |  |  |  |  |  |  |  |  |

| **jvBb b¤^i** | **NUbv**  1.Lvevi †Zix / cwi‡ek‡bi Av‡M ............................. 1  2.LvIqvi Av‡M ..... 2  3.cvqLvbvi ci wkï‡K / b¨vwc cwi¯Kvi Kivi ci ........ 3  4.cvqLvbv e¨env‡ii ci ...........4  5.Kvwk/nvwPi ci ......5  6.bvK Svivi ci ..... 6  7.ev”Pvi bvK Svivi ci .......7  8.Ab¨vb¨ wbw`ó© Kiƒb ............99 | **ch©‡e¶‡bi mgq**  24 N›Uv di‡gU  N›Uv : wg: | **evoxi m`m¨**  < 5 ermi wkï - 1  > 5 ermi < 18 ermi wkï - 2  > 18 ermi e¨vw³ - 3 | **evoxi m`‡m¨i †RÛvi:**  **gwnjv - 0**  **cyi“l – - 1** | **evox m`m¨ wK nvZ ay‡q‡Q?**  *n¨vu -- 1*  *bv -- 0*  *Rvwb bv -- 9*  *hw` 0 A_ev 9 nq Zvn‡j cieZx© NUbvq hvb|* | **nvZ †avqvi ¯’vb**  ivbœvN‡i -- 1  cvqLvbvq -- 2  cÖv_wgK nvZ †avqvi ¯’v‡b -- 4  evjwZ / Ab¨ wKQz -- 5  DVv‡b Ab¨ †Kv_vI nvZ †avqvi ¯’v‡b bq, -- 6  ivbœvN‡I Ab¨ †Kv_vI nvZ †avqvi ¯’v‡b bq -- 7  Ab¨vb¨, wbw`©wó Ki“b -- 8 | **`yB nvZ wK ay‡q‡Q?**  n¨vu -- 1  bv -- 2  Rvwb bv -- 3  cÖhR¨ bq – -- 0 | **nvZ †avqvi Dcv`vb t**  evi mvevb ----- 1  QvB --------- 2  gvwU ------- 3  ïay cvwb ----- 4  Ab¨vb¨ wbwÏ©ó Kiƒb ------ 8  cÖhR¨ bq ------ 9 | **gš—e¨** |
| --- | --- | --- | --- | --- | --- | --- | --- | --- | --- |
| 21 |  |  |  |  |  |  |  |  |  |
| 22 |  |  |  |  |  |  |  |  |  |
| 23 |  |  |  |  |  |  |  |  |  |
| 24 |  |  |  |  |  |  |  |  |  |
| 25 |  |  |  |  |  |  |  |  |  |
| 26 |  |  |  |  |  |  |  |  |  |
| 27 |  |  |  |  |  |  |  |  |  |
| 28 |  |  |  |  |  |  |  |  |  |
| 29 |  |  |  |  |  |  |  |  |  |
| 30 |  |  |  |  |  |  |  |  |  |

| **jvBb b¤^i** | **NUbv**  1.Lvevi †Zix / cwi‡ek‡bi Av‡M ............................. 1  2.LvIqvi Av‡M ..... 2  3.cvqLvbvi ci wkï‡K / b¨vwc cwi¯Kvi Kivi ci ........ 3  4.cvqLvbv e¨env‡ii ci ...........4  5.Kvwk/nvwPi ci ......5  6.bvK Svivi ci ..... 6  7.ev”Pvi bvK Svivi ci .......7  8.Ab¨vb¨ wbw`ó© Kiƒb ............99 | **ch©‡e¶‡bi mgq**  24 N›Uv di‡gU  N›Uv : wg: | **evoxi m`m¨**  < 5 ermi wkï - 1  > 5 ermi < 18 ermi wkï - 2  > 18 ermi e¨vw³ - 3 | **evoxi m`‡m¨i †RÛvi:**  **gwnjv - 0**  **cyi“l – - 1** | **evox m`m¨ wK nvZ ay‡q‡Q?**  *n¨vu -- 1*  *bv -- 0*  *Rvwb bv -- 9*  *hw` 0 A_ev 9 nq Zvn‡j cieZx© NUbvq hvb|* | **nvZ †avqvi ¯’vb**  ivbœvN‡i -- 1  cvqLvbvq -- 2  cÖv_wgK nvZ †avqvi ¯’v‡b -- 4  evjwZ / Ab¨ wKQz -- 5  DVv‡b Ab¨ †Kv_vI nvZ †avqvi ¯’v‡b bq, -- 6  ivbœvN‡I Ab¨ †Kv_vI nvZ †avqvi ¯’v‡b bq -- 7  Ab¨vb¨, wbw`©wó Ki“b -- 8 | **`yB nvZ wK ay‡q‡Q?**  n¨vu -- 1  bv -- 2  Rvwb bv -- 3  cÖhR¨ bq – -- 0 | **nvZ †avqvi Dcv`vb t**  evi mvevb ----- 1  QvB --------- 2  gvwU ------- 3  ïay cvwb ----- 4  Ab¨vb¨ wbwÏ©ó Kiƒb ------ 8  cÖhR¨ bq ------ 9 | **gš—e¨** |
| --- | --- | --- | --- | --- | --- | --- | --- | --- | --- |
| 31 |  |  |  |  |  |  |  |  |  |
| 32 |  |  |  |  |  |  |  |  |  |
| 33 |  |  |  |  |  |  |  |  |  |
| 34 |  |  |  |  |  |  |  |  |  |
| 35 |  |  |  |  |  |  |  |  |  |
| 36 |  |  |  |  |  |  |  |  |  |
| 37 |  |  |  |  |  |  |  |  |  |
| 38 |  |  |  |  |  |  |  |  |  |
| 39 |  |  |  |  |  |  |  |  |  |
| 40 |  |  |  |  |  |  |  |  |  |

ab¨ev`, Avgvi ch©‡e¶b GLv‡bB †kl|

**wefvM - wZ©b t nvZ †avqv cÖ`k©b**

**DËi `vZv‡K ejyb: Avcwb wK Avgv‡K AbyMÖn K‡i †`Lv‡Z cv‡ib †h Kvwk †`Iqvi mgq Avcwb wVK wK K‡ib? AbyMÖn K‡i Avcbvi cÖwZw`‡bi Af¨vm m¤ú‡K© †f‡e †`Lyb Ges hv Avcbvi Kv‡Q mvavib ZvB K‡i †`Lvb| GLv‡b †Kvb fzj DËi †bB| AbyMÖn K‡i Avcwb hv hv K‡ib Zv eY©bv bv K‡i Avgv‡K K‡i †`Lvb|**

**23. evoxi †Kvb m`m¨ Kvwk ev nvwP cÖ`k©b Ki‡eb? (e‡· †KvW wjL~b)**

**Bb‡W· †Km †ivMx .......................................... 1**

**AwffveK .................................. 2**

**evoxi m`m¨ ….............................. 3**

**24. †h m`m¨ Kvwk ev nvwP cÖ`k©b K‡i‡Qb Zvi BDwbK AvB. wW. t ÑÑÑÑÑÑÑÑÑÑÑÑÑÑÑÑÑÑÑÑÑÑÑÑÑ**

**25. ‡h m`m¨ Kvwk ev nvwP cÖ`k©b K‡i‡Qb Zvi eqm wjLyb t ÑÑÑÑ/ÑÑÑÑÑÑ(ermi/ gvm)**

**26. Kvwk ev nvwP welqK AvPiY cÖ`k©‡bi mgq wjLyb t (24 N›Uv di‡gU) ÑÑÑÑÑ/ÑÑÑÑÑÑÑÑ**

**27. ch©‡e¶Yt Bb‡W· †Km / AwffveK / DËi`vZv bx‡Pi †KvbwU K‡i‡Q wK ?**

**(Bb‡W· †Km / AwffveK / DËi`vZv bx‡Pi †KvbwU K‡i _v‡K Zvn‡j bx‡Pi e‡· Gi Rb¨ Ò1Ó wjLyb| hw` Bb‡W· †Km / AwffveK / DËi`vZv bx‡Pi †KvbwU bv K‡i _v‡K Zvn‡j bx‡Pi e‡· ÒbvÓ Gi Rb¨ Ò2Ó wjLyb)**

hw` Bb‡W· †Kvb iæMx / AwffveK bx‡Qi †KvbwU K‡i bv †`Lvq Zvn‡j ÒbvÓ Gi Rb Ò2Ó wjLyb) ch©‡e¶YKjv‡g hw` Bb‡W· ‡Km iæMx/ AwffveK g~‡L e‡j K‡i bv †`Lvq Zvn‡j †gŠwLK Kjv‡g *Òn¨vuÕGi Rb¨ 1 wjLyb| hw` AwffveK g~‡L bv e‡j Ges K‡I bv †`Lvq Zvn‡j ch©‡eÿb Kjv‡g bv Gi Rb¨ 2 wjL~b|*

NUbv ch©‡eÿb ‡gŠwLK

| **Kva / evû‡Z nvuwP/ Kvwk w`‡q‡Q** |  |  |  |  |  |
| --- | --- | --- | --- | --- | --- |
| **Rvgvi nvZv / kvox‡Z nvuwP/ Kvwk w`‡q‡Q** |  |  |  |  |  |
| **nv‡Z nvwPu / Kvwk w`‡q‡Q** |  |  |  |  |  |
| **evZv‡m nvwPu / Kvwk w`‡q‡Q** |  |  |  |  |  |
| **D‡j­L K‡i‡Q †h †m Zvici nvZ †av‡e** |  |  |  |  |  |
| **Ab¨vb¨ (wbw`wó Ki“b)** |  |  |  |  |  |

**28. ch©‡e¶Y t Kvwk ev nvuwP †`Iqvi 5 wg: Gi g‡a¨ wK DËi`vZv nvZ ay‡q‡Q? (e‡· b¤^i wjLyb)**

**n¨vu-------------------- 1**

**bv--------------------- 2**

***hw` 28 Gi DËi bv nq Zv n‡j wefvM-wZb G hvb|***

**29. ch©‡e¶Y t Kvwk ev nvuwP †`Iqvi 5 wg: Gi g‡a¨ nvZ †avqvi mgq DËi `vZv bx‡Pi †KvbwU e¨envi K‡i‡Q wK ?**

**(hw` Bb‡W· †Km / AwffveK / DËi`vZv hw` e¨envi K‡i _v‡K Zvn‡j bx‡Pi e‡· n¨vu Gi Rb¨ Ò1Ó wjLyb| hw` Bb‡W· †Km / AwffveK / DËi`vZv e¨envi bv K‡i _v‡K Zvn‡j bx‡Pi e‡· ÒbvÓ Gi Rb¨ Ò2Ó wjLyb|)**

**evi mvevb / mvaviY mvevb ÑÑÑÑÑÑÑÑÑÑÑÑÑÑÑÑÑÑÑÑÑÑÑÑÑÑÑ**

**¸ov mvevb ÑÑÑÑÑÑÑÑÑÑÑÑÑÑÑÑÑÑÑÑÑÑÑÑÑÑÑ**

**Zij mvevb ÑÑÑÑÑÑÑÑÑÑÑÑÑÑÑÑÑÑÑÑÑÑÑÑÑÑÑ**

**QvB ÑÑÑÑÑÑÑÑÑÑÑÑÑÑÑÑÑÑÑÑÑÑÑÑÑÑÑ**

**gvwU / evwj ÑÑÑÑÑÑÑÑÑÑÑÑÑÑÑÑÑÑÑÑÑÑÑÑÑÑÑ**

**Ab¨vb¨ (wbw`©ó Kiƒb) ÑÑÑÑÑÑÑÑÑÑÑÑÑÑÑÑÑÑÑÑÑÑÑÑÑÑÑ**

**wefvM Pvi t nvZ †avqv cÖ`k©b - cvqLvbvi ci wkï‡K cwi¯‹^^vi Kiv |**

**hw` ICP <5 ermi nq Zvn‡j Zv‡K wb‡q cÖ`kbwU Ki‡Z n‡e| hw` ICP > 5ermi nq Zvn‡j Zvi Lvbv‡Z < 5 ermi †Kvb wkï Av‡Q wK bv Zv †`L‡Z n‡e| hw` ICP Lvbv‡Z < 5 ermi †Kvb wkï bv _v‡K Zvn‡j**  **DËi`vZv‡K wRÁvmv Kiyb t Avcwb wK Avcbvi evoxi me‡P‡q †QvU wkïwUi gv A_ev Zvi cÖv_wgK cwiPhv©Kvixi Kv‡Q Avgv‡K wb‡q †h‡Z cv‡ib?**

**hw` me‡P‡q †QvU wkïwUi cÖv_wgK cwiPhv©Kvix Dcw¯’Z bv _v‡Kb , Zvn‡j cieZx© me‡P‡q Aí eqmx wkïwUi cªv_wgK cwiPhvKvix m¤ú‡K© wRÁvmv Ki“b, hZ¶b bv Avcwb GKRb cÖv_wgK cwiPhv©Kvix‡K mbv³ Ki‡Z cvi‡Qb †h mv¶vZKvi MÖn‡bi mgq Dcw¯’Z i‡q‡Q|**

**30. evox‡Z <5 eQ‡ii †Kvb wkï Av‡Q wK? (e‡· b¤^i wjLyb)**

**n¨vu-------------------- 1**

**bv--------------------- 2**

*hw` 30 Gi DËi bv nq Zv n‡j 39 b¤^i cÖ‡kœ hvb|*

**31. wkïwU wK ICP? (e‡· b¤^i wjLyb)**

**n¨vu-------------------- 1**

**bv--------------------- 2**

**hw` Bb‡W· †Kvb iæMx /AwfeveK bx‡Pi †KvbwU K‡i bv †`Lvq Zvn‡j bv Gi Rb¨ 2 wjLyb ch©‡eÿY Kjv‡g hw` Bb‡W· †Kvb iæMx/ AwfeveK gy‡L e‡j K‡i bv †`Lvq Zvn‡j †gŠwLK Kjv‡g *Òn¨vuÕÕcÖ‡qvR‡b 1 wjLyb|hw` AwffveK g~‡K bv e‡j Ges Kv‡R bv †`Lvq Zvn‡j ch©‡eÿb Kjv‡g bv Gi Rb¨ 2 wj_~b|***

NUbv ch©‡eÿb ‡gŠwLK

| **cwi¯‹‡ii mgq wkï‡K ¯úk© K‡i‡Q** |  |  |
| --- | --- | --- |
| **cwi¯‹vi Kivi mgq cvwb e¨venvi K‡i‡Q** |  |  |
| **mvevb I cvwb w`‡q (mvev‡bi cÖKvi D‡jø‡Li cÖ‡qvRb †bB )** |  |  |
| **mvevb w`‡q nvZ †avqvi mgq 24 N›Uv di‡gU** |  |  |
| **nvZ ay‡q‡Q (cwb e¨venvi K‡i‡Q ev D‡jøL K‡i‡Q wKš‘ mvevb bq)|** |  |  |
| **cvwb w`‡q nvZ †avqvi mgq 24 N›Uv di‡gU** |  |  |

**32. cÖv_wgK cwiPhv©Kvix‡K wRÁvmv Ki“b t Avcbvi wkïi eqm KZ? (ermi / gvm) ÑÑÑ/ÑÑÑ**

**33. cÖv_wgK cwiPhv©Kvix‡K wRÁvmv Ki“b t Avcbvi eqm KZ? (ermi / gvm) ÑÑÑ/ÑÑÑ**

**34. cÖv_wgK cwiPhv© Kvixi †RÛvi wjL~b : (e‡· †KvW wjL~b)**

**cyi“l ................................................... 1**

**gwnjv .................................................. 2**

**35. cÖv_wgK cwiPhv© Kvixi BDwbK AvB. wW. wjL~b t ÑÑÑÑÑÑÑÑÑÑÑÑÑÑÑÑÑÑÑÑÑÑÑÑÑÑÑÑÑÑÑÑ**

**cÖv_wgK cwiPhv©Kvix‡K wRÁvmv Ki“b AbyMÖn K‡i wkï†K cvqLvbvi ci wKfv‡e Zv‡K cwi¯Kvi K‡ib Zv †`Lv‡Z cv‡ib wK? | ch©‡eÿb Kiæb ch©‡eÿ‡bi 5 wg: g‡a¨ wZwb mn nvZ ay‡q‡Qb wK bv| cwiPhv©Kvix Ggb fv‡e wkï‡K cwi¯^vi Kiv‡e hv wVK ev¯Í‡e wkïwU cvqLvbv Ki‡j wZwb Ki‡Zb †hgb cÖ_‡g wkïi Kvco Ly‡j †`Iqv|**

**36. wkï‡K cwi¯^vi Kiv cÖ`k‡bi mgq wjL~b ( 24 N›Uv di‡gU) ÑÑÑ/ÑÑÑÑ**

**37. ch©‡e¶Yt wkï cwiPhv©Kvix wK wkï‡K cwi¯‹vi Kivi 5 wg: Gi g‡a¨ nvZ ay‡q‡Q? (e‡· †KvW wjL~b)**

**n¨vu-------------------- 1**

**bv--------------------- 2**

***hw` 37 Gi DËi bv nq Zv n‡j 39 b¤^i cÖ‡kœ hvb***

**38. ch©‡e¶Yt wkï cwiPhv©Kvix 5 wg: Gi g‡a¨ nvZ †avqvi mgq bx‡Pi †KvbwU e¨envi K‡i‡Q? (hw` wkï cwiPhv©Kvix †Kvb Dcv`vb e¨envi K‡i _v‡K Zvn‡j bx‡Pi e‡· Òn¨vuÓ &Gi Rb¨ Ò1Ó wjLyb| hw` wkï cwiPhv©Kvix †Kvb Dcv`vb e¨envi bv K‡i _v‡K Zvn‡j bx‡Pi e‡· Òbv Ò Gi Rb¨ Ò2Ó wjLyb)**

**evi mvevb / mvaviY mvevb ÑÑÑÑÑÑÑÑÑÑÑÑÑÑÑÑÑÑÑÑÑÑÑÑÑÑÑ**

**¸ov mvevb ÑÑÑÑÑÑÑÑÑÑÑÑÑÑÑÑÑÑÑÑÑÑÑÑÑÑÑ**

**Zij mvevb ÑÑÑÑÑÑÑÑÑÑÑÑÑÑÑÑÑÑÑÑÑÑÑÑÑÑÑ**

**QvB ÑÑÑÑÑÑÑÑÑÑÑÑÑÑÑÑÑÑÑÑÑÑÑÑÑÑÑ**

**gvwU / evwj ÑÑÑÑÑÑÑÑÑÑÑÑÑÑÑÑÑÑÑÑÑÑÑÑÑÑÑ**

**Ab¨vb¨ (wbw`©ó Kiƒb) ÑÑÑÑÑÑÑÑÑÑÑÑÑÑÑÑÑÑÑÑÑÑÑÑÑÑÑ**

**cÖv_wgK cweP©hvKvix‡K mgq †`qvi Rb¨ ab¨ev` Rvbvb | mv¶vZKv‡ii evKx Ask n‡e g~j DËi`vZvi mv‡_|**

**39.GBwU wK evox‡Z P’ovš— d‡jv Avc ? (e‡· †KvW wjL~b)**

**n¨vu-------------------- 1**

**bv--------------------- 2**

***hw` Ò39Ó Gi DËi ÓbvÓ nq Zvn‡j mv¶vZKvi MÖnb GLv‡bB †kl Ki“b |***

**wefvM cvuP t nvZ †avqv m¤úwK©Z Ávb**

**40.wRÁvmv Ki“b: KLb Avcbvi nvZ a~qv DwPZ? ïa DËi `vZv‡K wRÁvmv Ki“b**

**(hw` Bb‡W· †Km/ AwffveK / DËi `vZv wbgœewb©Z mgq ¸‡jv D‡jL K‡i Zvn‡j bx‡Pi e‡· Òn¨vuÓ &Gi Rb¨ Ò1Ó wjLyb| hw` Bb‡W· †Km/ AwffveK / DËi `vZv wbgœewb©Z mgq ¸‡jv D‡jL bv K‡i _v‡K Zvn‡j bx‡Pi e‡· Òbv Ò Gi Rb¨ Ò2Ó wjLyb)**

**ivbœv Kivi Av‡M ÑÑÑÑÑÑÑÑÑÑÑÑÑÑÑÑÑÑÑÑÑÑÑÑÑÑÑ**

**LvIqvi Av‡M ÑÑÑÑÑÑÑÑÑÑÑÑÑÑÑÑÑÑÑÑÑÑÑÑÑÑÑ**

**nv‡Z nvuwP ev Kvwk †`Iqvi c‡i ÑÑÑÑÑÑÑÑÑÑÑÑÑ**

**wb‡Ri A_ev wkïi bvK cwi¯‹vi Kivi c‡i ÑÑÑÑÑÑÑÑÑÑÑÑ**

**nv‡Z gqjv jvM‡j ÑÑÑÑÑÑÑÑÑÑÑÑÑÑÑÑÑÑÑÑÑÑÑÑÑÑÑ**

**cvqLvbvi c‡i ÑÑÑÑÑÑÑÑÑÑÑÑÑÑÑÑÑÑÑÑÑÑÑÑÑÑÑ**

**cvqLvbi ci wkï†K cwi¯‹vi Kivi c‡i ÑÑÑÑÑÑÑÑÑÑÑÑÑÑ**

**bvgv‡hi mgq ÑÑÑÑÑÑÑÑÑÑÑÑÑÑÑÑÑÑÑÑÑÑÑÑÑÑÑ**

**†Mvm‡ji mgq ÑÑÑÑÑÑÑÑÑÑÑÑÑÑÑÑÑÑÑÑÑÑÑÑÑÑÑ**

**mKv‡j Nyg †_‡K D‡VÑÑÑÑÑÑÑÑÑÑÑÑÑÑÑÑÑÑÑÑÑÑÑÑÑÑÑ**

**Ab¨vb¨ (wbwÏó KiyY) ÑÑÑÑÑÑÑÑÑÑÑÑÑÑÑÑÑÑÑÑÑÑÑÑÑÑÑ**

**41. wRÁvmv Ki“b : nvZ †avqvi mgq wK wK avc Avcwb K‡ib?**

**(hw` Bb‡W· †Km/ AwffveK / DËi `vZv wb‡¤§ewb©Z avc ¸‡jv D‡j­L K‡i Zv n‡j bx‡Pi e‡· Òn¨vuÓ &Gi Rb¨ Ò1Ó wjLyb| hw` Bb‡W· †Km/ AwffveK / DËi `vZv wbgœewb©Z avc ¸‡jv D‡jL bv K‡i _v‡K Zvn‡j bx‡Pi e‡· Òbv Ò Gi Rb¨ Ò2Ó wjLyb)**

**nvZ †fRv‡bv ÑÑÑÑÑÑÑÑÑÑÑÑÑÑÑÑÑÑÑÑÑÑÑÑÑÑÑÑ**

**nv‡Z mvev‡bi †dbv †Zvjv ÑÑÑÑÑÑÑÑÑÑÑÑÑÑÑÑÑÑÑÑÑÑÑ**

**10 †m‡KÛ nvZ Nmv ÑÑÑÑÑÑÑÑÑÑÑÑÑÑÑÑÑÑÑÑÑÑÑÑÑÑÑ**

**cvwb w`‡q nvZ ay‡q †djv ÑÑÑÑÑÑÑÑÑÑÑÑÑÑÑÑÑÑÑÑÑÑÑÑÑ**

**nvZ ïKv‡bv ÑÑÑÑÑÑÑÑÑÑÑÑÑÑÑÑÑÑÑÑÑÑÑÑÑÑÑÑÑÑÑÑ**

**Ab¨vb¨ (wbwÏ©ó Ki“b) ÑÑÑÑÑÑÑÑÑÑÑÑÑÑÑÑÑÑÑÑÑÑÑÑÑÑÑ**

**myweav I evav mg~n t**

**42. wRÁvmv Ki“b mvevb w`‡q Avcbvi nvZ †avqvi myweav¸wj wK wK ? (hw` DËi`vZv bx‡Pi †KvbwU e‡j Zvn‡j bx‡Pi e‡· Òn¨vuÓ &Gi Rb¨ Ò1Ó wjLyb| hw` DËi `vZv bx‡Pi †KvbwU bv e‡j Zvn‡j bx‡Pi e‡· Òbv Ò Gi Rb¨ Ò2Ó wjLyb)**

**wkï I cwiev‡ii jvjb cvjb ÑÑÑÑÑÑÑÑÑÑÑÑÑÑÑÑÑÑÑÑÑÑÑÑÑÑÑÑ**

**mgv‡Ri GKRb MÖnb‡hvM¨ e¨w³ nIqv ÑÑÑÑÑÑÑÑÑÑÑÑÑÑÑÑÑÑÑÑÑÑ**

**wb‡R‡K cwi¯‹vi †`Lv‡bv I Abyfe Kiv ÑÑÑÑÑÑÑÑÑÑÑÑÑÑÑÑÑÑÑÑÑÑ**

**gqjv `~i K‡i ÑÑÑÑÑÑÑÑÑÑÑÑÑÑÑÑÑÑÑÑÑÑÑÑÑÑÑÑÑÑÑÑÑÑÑÑÑÑÑ**

**¯^v‡¯’¨i DbœwZ K‡i ÑÑÑÑÑÑÑÑÑÑÑÑÑÑÑÑÑÑÑÑÑÑÑÑÑÑÑÑÑÑÑÑÑÑÑÑ**

**mvgvwRK ghv©`v evovq ÑÑÑÑÑÑÑÑÑÑÑÑÑÑÑÑÑÑÑÑÑÑÑÑÑÑÑÑÑÑÑÑÑÑÑ**

**Wvqwiqv RwbZ Amy¯’Zv Kgvq ÑÑÑÑÑÑÑÑÑÑÑÑÑÑÑÑÑÑÑÑÑÑÑÑÑÑÑÑÑÑ**

**k¦vmZ‡š¿i Amy¯’Zv Kgvq ÑÑÑÑÑÑÑÑÑÑÑÑÑÑÑÑÑÑÑÑÑÑÑÑÑÑÑÑÑÑÑÑ**

**Ab¨vb¨ (wbw`ó Ki“b) ÑÑÑÑÑÑÑÑÑÑÑÑÑÑÑÑÑÑÑÑÑÑÑÑÑÑÑÑÑÑÑÑÑÑÑ**

**43. wRÁvmv Ki“b mvevb w`‡q nvZ †avqvi Rb¨ Avcbvi evav mg~n wK wK? (hw` DËi`vZv bx‡Pi †KvbwU e‡j Zvn‡j bx‡Pi e‡· Òn¨vuÓ &Gi Rb¨ Ò1Ó wjLyb| hw` DËi `vZv bx‡Pi †KvbwU bv e‡j Zvn‡j bx‡Pi e‡· Òbv Ò Gi Rb¨ Ò2Ó wjLyb)**

**‰`bw›`b Kv‡Ri A¯—©MZ bq ev Af¨vm bq ÑÑÑÑÑÑÑÑÑÑÑÑÑÑÑÑÑÑÑÑÑÑ**

**m~weavw` m¤ú‡K© AewnZ bq / Áv‡bi Afve ÑÑÑÑÑÑÑÑÑÑÑÑÑÑÑÑÑÑÑÑÑ**

**AwZwi³ kw³ LiP nq/ AjmZv ÑÑÑÑÑÑÑÑÑÑÑÑÑÑÑÑÑÑÑÑÑÑÑÑÑÑÑÑÑÑ**

**mvevb wKb‡Z AwZwi³ LiP nq ÑÑÑÑÑÑÑÑÑÑÑÑÑÑÑÑÑÑÑÑÑÑÑÑÑÑÑÑÑÑ**

**Amy¯’Zv cÖwZ‡iva K‡i bv ÑÑÑÑÑÑÑÑÑÑÑÑÑÑÑÑÑÑÑÑÑÑÑÑÑÑÑÑÑÑÑÑÑÑÑ**

**nvZ †avqvi wbwÏó †Kvb ¯’vb †bB ev Zv A‡bK `~‡i ÑÑÑÑÑÑÑÑÑÑÑÑÑÑÑÑÑÑÑÑ**

**mvevb w`‡q nvZ †avqv m¤ú‡K© †Kvb weKvi bvB ÑÑÑÑÑÑÑÑÑÑÑÑÑÑÑÑÑÑÑÑÑÑ**

**QvB A_ev gvwU w`‡qB cwi¯‹vi nh ÑÑÑÑÑÑÑÑÑÑÑÑÑÑÑÑÑÑÑÑÑÑÑÑÑÑÑÑÑÑ**

**cvwb wb‡RB cwi¯‹vi K‡i ÑÑÑÑÑÑÑÑÑÑÑÑÑÑÑÑÑÑÑÑÑÑÑÑÑÑÑÑÑÑÑÑÑÑÑ**

**Lye e¨¯’ / mgq bvB ÑÑÑÑÑÑÑÑÑÑÑÑÑÑÑÑÑÑÑÑÑÑÑÑÑÑÑÑÑÑÑÑÑÑÑÑÑÑÑÑ**

**mvevb w`‡q nvZ ay‡Z f~‡j hvq ÑÑÑÑÑÑÑÑÑÑÑÑÑÑÑÑÑÑÑÑÑÑÑÑÑÑÑÑÑÑÑÑÑ**

**mvevb Pzwi n‡q hv‡e ÑÑÑÑÑÑÑÑÑÑÑÑÑÑÑÑÑÑÑÑÑÑÑÑÑÑÑÑÑÑÑÑÑÑÑÑÑÑÑÑ**

**Ab¨vb¨ (wbwÏó K‡i wjLyb) ÑÑÑÑÑÑÑÑÑÑÑÑÑÑÑÑÑÑÑÑÑÑÑÑÑÑÑÑÑÑÑÑÑÑÑÑ**

**‡Kvb evav bvB ÑÑÑÑÑÑÑÑÑÑÑÑÑÑÑÑÑÑÑÑÑÑÑÑÑÑÑÑÑÑÑÑÑÑÑÑÑÑÑÑÑÑÑ**

**wefvM cvuP t Bbd¬z‡qÁv m¤úwK©Z Ávb**

**44. Avcwb wK Bbd¬z‡qÁv bvgK Amy¯’Zvi K_v KLbI ï‡b‡Qb? (e‡· †KvW wjLyb)**

**n¨vu -------------------------- 1**

**bv -------------------------- 2**

**Rvwb bv / wbwðZ bv ------------- 9**

***hw` Ó44Ó Gi DËi 2 A_ev 9 nq Zvn‡j mv¶vZKvi Mªnb GLv‡bB †kl Ki“b***

**45. wRÁvmv Ki“b Bd¬z‡qÁv RwbZ Amy¯’vZvi mv‡_ wK wK j¶Y RwoZ ? (cÖkœwU GKwU †Lvjv cÖkœ wnmv‡e wRÁvmv Ki“b hw` Bb‡W· †Km / DËi`vZv bx‡Pi †Kvb j¶Y D‡j­L K‡ib Zvn‡j bx‡Pi e‡· Òn¨vuÓ &Gi Rb¨ Ò1Ó wjLyb| hw` DËi `vZv bx‡Pi †KvbwU bv e‡j Zvn‡j bx‡Pi e‡· Òbv Ò Gi Rb¨ Ò2Ó wjLyb)**

**Kvwk .............................................................................**

**Mjv e¨v_v......................................................................**

**bvK w`‡q cvwb cov .........................................................**

**R¡i..............................................................................**

**gv_v e¨v_v .....................................................................**

**kixi e¨v_v ....................................................................**

**Ab¨vb¨ (wbwÏ®U K‡i wjLyb) ..............................................**

**46. wRÁvmv Ki“b t Bd¬z‡qÁv RwbZ Amy¯’Zv wK cÖwZ‡iva Kiv hvq ? (e‡· †KvW wjLyb)**

**n¨vu -------------------------- 1**

**bv -------------------------- 2**

**Rvwb bv / wbwðZ bv ------------- 9**

***hw` Ó46Ó Gi DËi 2 A_ev 9 nq Zvn‡j mv¶vZKvi Mªnb GLv‡bB †kl Ki“b***

**47. Bbd¬z‡qÁv RwbZ Amy¯’Zv wKfv‡e cÖwZ‡iva Kiv hvq?**

**(cÖkœwU GKwU †Lvjv cÖkœ wnmv‡e wRÁvmv Ki“b hw` Bb‡W· †Km / DËi`vZv bx‡Pi †Kvb cÖwZ‡iva D‡j­L K‡ib Zvn‡j bx‡Pi e‡· Òn¨vuÓ &Gi Rb¨ Ò1Ó wjLyb| hw` DËi `vZv bx‡Pi †KvbwU bv e‡j Zvn‡j bx‡Pi e‡· Òbv Ò Gi Rb¨ Ò2Ó wjLyb)**

**Amy¯’ e¨w³i KvQ †_‡K `~‡i hvevi gva¨‡g ÑÑÑÑÑÑÑÑÑÑÑÑÑÑÑÑÑÑÑÑÑÑÑÑÑÑ**

**evi evi nvZ †avqvi gva¨‡g ÑÑÑÑÑÑÑÑÑÑÑÑÑÑÑÑÑÑÑÑÑÑÑÑÑÑÑÑÑÑÑÑÑÑÑ**

**evi evi mvevb w`‡q nvZ †avqvi gva¨‡g ÑÑÑÑÑÑÑÑÑÑÑÑÑÑÑÑÑÑÑÑÑÑÑÑÑÑÑ**

**wUKv MÖn‡bi gva¨‡g ÑÑÑÑÑÑÑÑÑÑÑÑÑÑÑÑÑÑÑÑÑÑÑÑÑÑÑÑÑÑÑÑÑÑÑÑÑÑÑÑ**

**VvÛv Lvevi MÖnb Gwo‡q Pjvi gva¨‡g ÑÑÑÑÑÑÑÑÑÑÑÑÑÑÑÑÑÑÑÑÑÑÑÑÑÑÑÑÑ**

**cwi¯‹vi cwi”QbœZv eRvq ivLvi gva¨‡g ÑÑÑÑÑÑÑÑÑÑÑÑÑÑÑÑÑÑÑÑÑÑÑÑÑÑÑÑ**

**Ab¨vb¨ (wbwÏó K‡i wjLyb) ÑÑÑÑÑÑÑÑÑÑÑÑÑÑÑÑÑÑÑÑÑÑÑÑÑÑÑÑÑÑÑÑÑÑÑÑ**

Awd‡m e¨env‡ii Rb¨

Gd.Avi.G bvg : ___________________________________________

Gd.Avi.G ¯^v¶i: ___________________ ZvwiL t ______/______/____________

**cixw¶Z :** ______________________ ZvwiL t ______/______/____________

# Appendix 22: Responses to the comments from the external reviewers

**A. Responses to the comments from Dr. BJ Cowling**

This protocol describes a study to evaluate whether improved hand hygiene can prevent transmission of influenza within households in Bangladesh. The study is ambitious in the intensity and duration of follow-up of

a moderate number of households, and may be logistically challenging although it has the potential to generate invaluable data on influenza transmission and prevention in a subtropical low-income setting.

***We thank the reviewer sincerely for his thoughtful comments. We have attempted to respond to specific comments below. Overall, we believe that our proposed study design is logistically feasible. Moreover, the proposed study design will allow us to collect detailed information on secondary transmission at the household / bari level.***

**Major comments**

A strength of the proposed study is the timeliness of applying interventions following recruitment, where it is proposed that two team members will accompany a recruited case home immediately. The schedule also includes repeat home visits on a daily basis by the FRA to evaluate outcomes and in the intervention group by the FRO to maintain adherence to the intervention; my immediate concern is firstly whether this is feasible, and secondly whether it is necessary. I would be particularly concerned about the resources required to continue FRO daily visits for as long as 10 days after illness resolution in the index case to maintain the intervention; this design seems like overkill.

***To date, there is really only one study (ongoing) to examine the efficacy of hand hygiene promotion for prevention of secondary transmission of influenza. That study is being conducted in Bangkok, Thailand. There, only one intervention session is being held with households in the treatment arm. Handwashing is a difficult behavior to change. We have seen that, even after intensive interpersonal education over a yearlong period, improved handwashing behavior is not necessarily sustained (Luby, ASTMH, 2008). However, based on data regarding the impact of handwashing on all-cause respiratory illness, we hypothesize that improving hand hygiene will reduce influenza transmission at the household level. Our intent with daily visits by an FRO to reinforce the intervention is that we will demonstrate proof of the concept that improved handwashing behavior will, indeed, reduce secondary transmission. With a less than intensive, aggressive hand hygiene intervention, if we find no or little reduction of secondary transmission in the treatment arm, we will be left to wonder whether our findings were due a true lack of efficacy of hand hygiene or whether our intervention was inadequate. We have conducted the logistical exercises to determine whether we have the person-power and budget to afford daily intervention and illness tracking visits. We do have both person-power and budget.***

The inclusion criteria specify that an index case is eligible provided they have experienced symptom onset within 7 days. Unpublished data from the Hong Kong NPI study suggests that interventions are most effective if implemented within at most 36-48 hours of symptom onset in an index case. Narrowing the inclusion criteria to say symptom onset within 2 days would lead to a greater likelihood of detecting intervention effects, but would adversely impact recruitment rates. If a primary motivation for this study is pandemic preparedness, then one of the most interesting outputs must be the potential (theoretical) impact of immediate use of interventions following symptom onset.

***This is useful input. In the ongoing surveillance, we encounter many patients who come days after symptom onset. As Dr. Cowling notes, it would be interesting and important to understand whether intervening early would prevent secondary transmission. For this reason, we propose to retain the currently specified eligibility criteria but also propose to conduct subgroup analyses based on the duration of symptoms preceding enrollment.***

In the Hong Kong NPI study, children under the age of 15 were found to shed influenza virus for on average 7 days after symptom onset (unpublished data). Symptoms typically did not resolve until after cessation of viral shedding, and there are data in the literature suggesting children can have symptoms for 14 days or longer. In the current design, this would lead to daily visits for 24 days or more, which seems an excessive use of resources to study transmission in just one household.

***If we were to stop assessing illness in contacts before their symptoms were resolved, we could miss secondary cases. Since the unit of observation is the bari, each index case could potentially transmit to 10 or more susceptible persons. This large pool of susceptibles makes daily visits to the bari extremely worthwhile. Again, as noted above, we have the person-power and budget to pursue this strategy.***

The evaluation of primary outcome may be suboptimal particularly to answer Specific Aim 2 and bearing in mind the limited sample size. Influenza infection is associated with a wide variety and combination of symptoms. In the Hong Kong NPI study, only 8 out of 21 laboratory-confirmed secondary cases met the strict clinical definition of fever plus cough or sore throat in the pilot study (published in PLoS ONE), and 34 out of 75 in the main study (unpublished data). Approximately 20% of laboratory-confirmed secondary cases did not report any symptoms. In the proposed study, only household contacts who meet the strict clinical definition will provide specimens, and therefore more than half of all secondary cases will probably be missed (and any intervention effect will be less clear / the study will have less power). Clearly there would be logistical and budgetary implications if all household contacts were swabbed whether symptomatic or not; but perhaps a looser definition could be used for the purpose of collecting swabs

***We have updated the protocol to include in the case definition cough/sore throat OR fever in the last 7 days. We hope this will allow us to identify more symptomatic secondary cases. While identifying asymptomatic secondary cases would be interesting and allow for a more “complete” estimation of secondary transmission, it would not modify the identification of clinically relevant (i.e. symptomatic) illnesses among household contacts.***

The targeted ‘maximum’ sample size of up to 100 households per arm (200 in total) appears adequate while the ‘minimum’ sample size is unlikely to be sufficient. It is likely that secondary attack ratios will be under 10% rather than as high as 30% particularly if ascertainment is limited to contacts meeting the fever+cough/sore throat definition (see #4 above) and given the likely lower contact rates in the bahi compared to single households. It would seem optimistic to obtain the maximum sample size if the pool of potential recruits is at most 240. The sample size calculation does not allow for dropouts post-recruitment.

***We have read Dr. Cowling’s paper published in PLoS ONE with interest. More recently, we have heard oral presentation of data from the HITS study from Bangkok, Thailand, in which handwashing and face mask use are being assessed for efficacy against secondary transmission of influenza. The rates of secondary transmission in Bangkok are about 30%, much higher than the 8% seen in the Hong Kong NPI study. Baris represent familial units and there is substantial daily ongoing interaction between most bari members. We believe that the Bangladeshi population will more closely resemble the Bangkok population, based on socioeconomic status indicators. Thus, we anticipate that our power calculations will hold true. We have modeled our sample size calculations on a range of secondary transmission rates and find that our estimated sample size will be adequate to demonstrate 33% risk reduction, in case the SAR in the routine practices group is only 20% (between the SAR found in Hong Kong and that found in Bangkok).***

The lack of serology is a limitation; baseline serology can help to clarify immunity/susceptibility and comparison with post-study serology could allow optimal detection of influenza virus infections during the study. Would it be possible to collect baseline and post-study serology even only in a subset of households?

***We agree that this may be a limitation. However, at present, the budget available to us will not allow for serological testing of household contacts.***

Is there a strong reason for using bar soap rather than liquid hand soap?

***We are using bar soap because that is the kind of soap commonly used in Bangladesh. Liquid soap is unusual in this setting and does not represent the intervention that would be implemented in the case of a pandemic,.***

**Minor comments**

The investigative team does not seem to have a designated statistician.

***Margaret DiVita is a PhD student at the University at Buffalo. Margaret will be analyzing data from this study for her doctoral dissertation. Dr. Jihnhee Yu, a biostatistician in the Department of Biostatistics at the University at Buffalo, will advise Margaret on the statistical analysis of these data.***

The assessment of ventilation is fairly simplistic although pragmatic; the protocol is missing an interesting opportunity to really make a comprehensive study of environmental factors affecting transmission. New technologies are not particularly expensive and might allow tracking of carbon dioxide levels, or a tracer substance can be steadily released in one room and detected in other rooms. Timelines may not allow this to be set up this year, but the investigators can consider contacting a specialist engineer for future studies.

***This is useful input. We are planning on measuring PM50 using the Berkeley Particle Monitor, as detailed in the Measures of Interest section.***

It could be interesting to make a more detailed study of contact patterns within households and between households within a bari, perhaps in a subset. For example ask each member to report the number of skin-to-skin or conversational contacts with each other member, each day.

***This is an interesting suggestion. However we feel that it would require hours spent in direct observation of each household to accurately collect this data, which would not be feasible given the remainder of our proposed study methods****.*

In terms of logistics, is there a strong rationale for sending the FRO to all households, and then asking them to call the physician and depart if the household is not in the intervention group. This may protect against certain biases, but on the other hand will the FRO be required to carry bottles of soap etc. to all households?

***The randomization is occurring at the bari level after the eligibility of the bari has been confirmed and baseline data has been collected. This will minimize interviewer bias during the baseline data collection***

Will specimens from symptomatic household contacts be tested for all influenza subtypes or only for the subtype corresponding to the strain detected in the index case? E.g. if the index case has influenza B, will the symptomatic household contacts only be tested for influenza B – or perhaps a pragmatic approach could be to test only for influenza B in the first instance, and test for A H1N1 or A H3N2 if the flu B test is negative?

***All swabs obtained from potential secondary cases will be tested for all influenza subtypes, except Influenza A H5N1. The latter will be tested for in the event that PCR is negative for Influenza A H1N1 and A H3N2, and Influenza B..***

Is influenza vaccination history so uncommon as not to warrant mention in Specific Aim 3? Perhaps it should be clarified in the protocol that vaccination rates are so low that the potential effects are unlikely to be observable.

***Influenza vaccination in this population is virtually nonexistent.***

The data collected in this study could be very amenable to mathematical modeling, although this does not necessarily need to be part of the study protocol and could be a separate supplementary project, if the investigators can find a modeler to collaborate with.

***We’d be interested in discussing this further with Dr. Cowling to learn his insights about the purpose and methods of such modeling. We will initiate this conversation separately.***

It seems strange to discard specimens corresponding to negative QuickVue tests, rather than freezing them. Funds might be available later to confirm test performance, and certainly the authors should keep and test the initial set of negative swabs to confirm that test performance is acceptable – there are recent reports from NPI studies in the US that QuickVue sensitivity was just 20%-30% in large sample sizes, although in most previous studies sensitivity has been 60%-70%.

***Members of our study team have assessed the sensitivity of QuickVue among residents of a slum community in Dhaka, Bangladesh’s capital and found it to be around 90%. We appreciate the caution that QuickVue’s sensitivity may be lower in other populations. We will measure and report the sensitivity and positive predictive value of QuickVue, as compared with PCR, upon conclusion of our study. We also will make an effort to freeze around 20 negative QuickVue specimens per month; should funds become available in the future, we will use these specimens to test for specificity and NPV.***

The Secondary Attack Rate is more of a ratio than a rate since time is not included in the denominator, and so it may be more appropriate to use Secondary Attack Ratio throughout (but ‘rate’ has been a common usage historically).

***We have updated the protocol to reflect “secondary attack ratio”.***

Sample size calculation for Specific Aims 2 and 3, “We assume 10 susceptible contacts per household…” should not refer to susceptibility/immunity as this is not evaluated in the study and is not involved in calculation of primary outcome measures.

***We have updated to protocol to say “We assume 10 contacts per household…”***

**B. Responses to the comments from Dr. Elaine Larson**

Good idea to obtain both nasal and OP swab, although it might be very difficult to obtain the OP.  Consider a deep nasal swab.  Provide rationale for both.

***These specimen collection procedures are already in place for national hospital-based influenza surveillance, which includes Jahurul Islam Medical College hospital where this study will be based. We have not had difficulty in obtaining OP swabs there. The sensitivity and specificity of this combination of specimens and, therefore, we do not see a need for a deep nasal swab. Our rationale for obtaining these samples is detailed in the “laboratory methods” section.***

In our recent CDC studies, QuickVue had a sensitivity of <50%--if possible with the funds you have, do lab tests on everybody who meets ILI definition.  At the very least, test the sens and specificity of QuickVue in a small sample of your population before deciding not to culture or do PCR.

***Members of our study team have assessed the sensitivity of QuickVue among residents of a slum community in Dhaka, Bangladesh’s capital and found it to be around 90%. We appreciate the caution that QuickVue’s sensitivity may be lower in other populations. Given the nature of the intervention (immediately upon identification of an index case-patient), the rapidity of secondary transmission (days after illness onset in the index case-patient), and the time frame needed to get PCR results (several weeks), we will need to rely on QuickVue results in order to make a decision on enrollment of a given index case-patient and his/her household contacts. Therefore, we propose to retain use of the QuickVue for identification of index case-patients. A lower than anticipated sensitivity of QuickVue would mean that a number of index case-patients who might otherwise be eligible for inclusion in the study would not be identified as influenza positive. This will not adversely affect our ability to answer the study questions, since we aim to assess the rate and prevention of secondary transmission, and not primary illness. To address this concern, we will measure and report the sensitivity and positive predictive value of QuickVue, as compared with PCR, upon conclusion of our study. Unfortunately, given the brevity of the influenza season (3-4 months), we will be unable to test the sensitivity of QuickVue in this particular population before initiating the proposed study.***

I don't think that your sample size calculation is convincing that you will have sufficient power.  In addition to the data provided, what is the anticipated incidence of flu in the community?  Will you have sufficient index cases during your time line?

***In the sample size calculation for specific aim two, we estimated that we will detect around 80 index cases of influenza per month, or a total of 240 cases during a three month influenza season. This data was extrapolated from a current study that is being conducted in the same population, from last year’s flu season (2008). Unfortunately, the study of influenza in Bangladesh is relatively young and we do not have population-based estimates of influenza incidence in all age groups in the community.***

Am I reading this correctly that the control group will not sign consents, only the intervention group?  That doesn't seem acceptable by US standards, but perhaps different in Bangladesh?

***We will be obtaining written consent from baris enrolled in both intervention and control arms. We have updated the protocol to make this clearer*.**

Is the same person doing the interviews, assessing the outcome, and taking swabs who is also doing the intervention?  It would be stronger if that were not the case, particularly since you cannot blind.

***The study workers that are implementing the intervention will be separate from the workers that are assessing illness in the household members. We have updated the protocol with new titles for our study workers to make this clearer. The study worker who will carry out the intervention will now be called the “field intervention specialist” or “FIS”. This person is distinct from the “field research assistant” or “FRA”, who will collect all study measures, including questionnaire administration and illness tracking..***

It would be great if you could comment briefly on sustainability of the intervention after the study is completed.

***This study is intended to demonstrate a proof of concept: does intensive handwashing promotion reduce the transmission of influenza from an index case-patient to household contacts? We do not make assumptions about the scalability or sustainability of such an intervention. If we can demonstrate that an intensive, well-designed, and well-executed handwashing promotion intervention does indeed reduce secondary transmission of influenza, we would then propose to examine the impact of a more scalable, and hopefully sustainable, handwashing promotion intervention. We believe that it is premature to discuss issues of sustainability and scalability at this time.***

**CHECK-LIST FOR SUBMISSION OF RESEARCH PROTOCOL**

**FOR CONSIDERATION OF RESEARCH REVIEW COMMITTEE (RRC)**

**[Please check (X) appropriate box]**

| 1. Has the proposal been reviewed, discussed and cleared at the Division level? | | | | | | |
| --- | --- | --- | --- | --- | --- | --- |
| Yes | ☒ | | No | | ☐ | |
| If No, please clarify the reasons: | | | | | | |
| 2. Has the proposal been peer-reviewed externally? | | | | | | |
| Yes | ☒ | | No | | ☐ | |
| If the answer is ‘No’, please explain the reasons: | | | | | | |
| If yes, have the external reviews’ comments and their responses been attached | | | | | | |
| Yes | | ☒ | | No | | ☐ |
| 3. Has the budget been cleared by Finance Department? | | | | | | |
| Yes | ☒ | | No | | ☐ | |
| If the answer is ‘No’, reasons thereof be indicated: | | | | | | |
| 4. Does the study involve any procedure employing hazardous materials, or equipments? | | | | | | |
| Yes | ☐ | | No | | ☒ | |
| If ‘Yes’, fill the necessary form. | | | | | | |
| ______________ _________ Signature of the Principal Investigator Date | | | | | | |
