# Supplementary material for: Impact of Intensive Handwashing Promotion on Secondary Household Influenza-Like Illness in Rural Bangladesh: Findings from a Randomized Controlled Trial
Source: PLoS One. 2015 Jun 11;10(6):e0125200. doi: 10.1371/journal.pone.0125200 (PMC4465839; doi:10.1371/journal.pone.0125200)
Supplement: S1 Text — (DOCX) [file pone.0125200.s003.docx]

# Modifications to eligibility criteria and changes based on recruitment

When the study protocol was initially developed, we specified that index case-patients were eligible if the index case-patient met the case definition (specified above), and if the index case-patient’shousehold compound was located within 30 minutes travel time one-way from the health facility *and* none of the index case-patient’s fellow compound members were reported to have fever within the 3 days before the index case-patient’s enrollment (Supplemental Table 1).

Within the first month following the study launch in July 2009 (Phase 1), we realized that we were at substantial risk of not meeting the desired sample size based on the pace of identification of eligible index case-patients. In an effort to salvage the investments made in study personnel and logistics and to increase recruitment, we requested and were granted approval from the research review committee at icddr,b to loosen the eligibility criteria for the remainder of the study (Phase 2): specifically, we allowed all index case-patients meeting the case definition irrespective of illness among household contacts, and allowed for the household compound to be located within 2 hours travel time one-way from the health facility.

By the end of the 2009 Influenza season (November 2009), we had randomized only 174 index case-patients (of a desired 400 index case patients). Additional funds became available from the US Centers for Disease Control and Prevention, allowing us to resume and continue the study during the 2010 Influenza season (Phase 3). In the intervening period between the 2009 and 2010 influenza seasons, we became aware of the data from Cowling and colleagues^[8](#_ENREF_8)^, demonstrating that handwashing promotion prevented secondary transmission of influenza if applied early in the course of the index case-patient’s illness (within 36 hours). Therefore, we altered the eligibility criteria for index case-patients for Phase 3: symptoms in the index case-patient consistent with influenza like-illness with symptom onset during the 48 hours prior to enrollment. We also allowed for fever to have been reported among secondary household compound members (i.e. members of the index case-patient’s household should not have experienced fever during the 7 days prior to enrollment but members of secondary households could have had fever during this period). A summary of the phase-specific eligibility criteria are provided in Supplemental Table 1.

# Table S1. Phases of enrollment of index case-patients and susceptible contacts, Bangladesh Interruption of Secondary Transmission of Influenza Study (BISTIS), Kishoregonj, Bangladesh, 2009-2010

| **Phase** | Total | I | II | III |
| --- | --- | --- | --- | --- |
| **Dates** | July 19 2009 – October 30, 2010 | July 19 – Sep 3, 2009 | Sep 4 – Nov 9, 2009 | May 6 – October 30, 2010 |
| **Case definition of index case-patient** |  | Age-specific definition for influenza-like illness* | Age-specific definition for influenza-like illness | Age-specific definition for influenza-like illness, with symptom onset during 48 hours preceding enrollment |
| **Exclusion criteria for illness in household compound members** | *--* | Compounds excluded if any compound member(s) reported to have fever within 3 days before index case-patient enrollment | Compounds included irrespective of illness in primary and secondary household members; (illness occurring within 7 days prior to enrollment recorded) | Compounds excluded if any primary household member reported to have fever  (fever occurring within 48 hours prior to enrollment recorded) |
| **Distance from health facility** | *--* | Compounds located within 30 minutes travel time one-way from health facility | Compounds located within 2 hours travel time one-way from health facility | Compounds located within 2 hours travel time one-way from health facility |
| **Inclusion in model 1 (intent to treat)** | All | All | All | All |
| **Inclusion in model 2 (restricted to those meeting 2010 criteria)** | Only those compounds meeting 2010 criteria for illness in household compound members | Only those compounds meeting 2010 criteria for fever occurring within 48 hours of enrollment (In 2009, we did not collect information about illness in individual compound members, since the exclusion criteria were evaluated at the time of index case-patient recruitment) | Only those compounds meeting 2010 criteria for illness in household compound members and fever occurring within 48 hours of enrollment | All |

*Age-specific case definition for influenza-like illness: 1) fever for children < 5 years old; 2) fever with cough *or* sore throat for persons > 5 years old

# Table S2. Randomization of household compounds, and exclusion of compounds and household members

|  | **Total** | | **Phase 1** | | **Phase 2** | | **Phase 3** | |
| --- | --- | --- | --- | --- | --- | --- | --- | --- |
|  | Intervention | Control | Intervention | Control | Intervention | Control | Intervention | Control |
| **Number of household compounds randomized** | 195 | 186 | 22 | 18 | 69 | 65 | 104 | 103 |
| **Number of household compounds remaining after withdrawals / exclusions after randomization* (explanation in footnote)** | 193 / 195  (99%) | 184/186  (99%) | 22/22  (100%) | 18/18  (100%) | 69/69  (100%) | 63/65  (97%) | 102/104 (98%) | 103/103  (100%) |
| **Number of household compound members enumerated in compounds after removal of withdrawn / excluded compounds (not including index case-patient)** | 1814 | 1607 | 184 | 178 | 617 | 479 | 1013 | 950 |
| **Number of compound members with fever at enrollment** | 78 / 1814  (4%) | 53/1607  (3%) | 0/184  (0%) | 0/178  (0%) | 66/617  (11%) | 46/479  (10%) | 12/1013  (1%) | 7/950  (1%) |
| **Number of susceptible compound members** | 1736 / 1814 (96%) | 1554/1607  (97%) | 184/184  (100%) | 178/178  (100%) | 551/617  (89%) | 433/479  (90%) | 1001/1013  (99%) | 943/950  (99%) |
| **Number of susceptible compound members without syndromic surveillance data** | 75/1736  (4%) | 56/1554  (4%) | 5/184  (3%) | 2/178  (1%) | 3/551  (1%) | 1/433  (0%) | 67/1001  (7%) | 53/943  (6%) |
| **Number of susceptible compound members with syndromic surveillance information for >1 days** | 1661/1736  (96%) | 1498/1554  (96%) | 179/184  (97%) | 176/178  (99%) | 548/551  (99%) | 432/433  (100%) | 934/1001  (93%) | 890/943  (94%) |
| **Number of susceptible members in index case-patient households** | 863/1661  (52%) | 727/1498  (49%) | 95/179  (53%) | 89/176  (51%) | 284/548  (52%) | 224/432  (52%) | 484/934  (52%) | 414/890  (47%) |
| **Number of susceptible members in other households** | 798/1661  (48%) | 771/1498  (51%) | 84/179  (47%) | 87/176  (49%) | 264/548  (48%) | 208/432  (48%) | 450/934  (48%) | 476/890  (53%) |
|  |  |  |  |  |  |  |  |  |
| **Number of index case-patients with PCR-confirmed influenza (% of total randomized)** | 24/193  (12%) | 36/184  (20%) | 2/22  (9%) | 3/18  (17%) | 11/69  (16%) | 17/63  (27%) | 11/102  (11%) | 16/103  (16%) |
| **Number of susceptible members in compounds of index case-patients with PCR-confirmed influenza** | 177/1661  (11%) | 250/1498  (17%) | 15/179  (8%) | 39/176  (22%) | 77/548  (14%) | 105/432  (24%) | 85/934  (9%) | 106/890  (12%) |

*excluded compounds that had travel time > 2 hours (# 1009), the compound withdrew from the study (# 3119), index case-patient was not present for > 3 days (# 2124 – removed because hh members not susceptible if index case-patient not present), and one compound was found to have fever among a member of the index case-patient household (4020 - intervention)

# Table S3. Secondary attack ratios of influenza-like illness, and influenza, among household compound members of index case-patients, by study phase and in total, Kishoregonj, Bangladesh, 2009-2010

| **Model** | Phase 1 | | | Phase 2 | | Phase 3 | | |
| --- | --- | --- | --- | --- | --- | --- | --- | --- |
| **Secondary transmission of influenza-like illness** | Intervention | | Control | Intervention | Control | Intervention | | Control |
| **Index case-patients (N)** | 22 | | 18 | 69 | 63 | 102 | 103 | |
| **Susceptible household members (N)** | 179 | | 176 | 548 | 432 | 934 | 890 | |
| **Secondary attack ratio** | 4/179 (2.2%) | | 6/176 (3.4%) | 44/548 (8.0%) | 25/432 (5.8%) | 110/934 (11.8%) | 84/890 (9.4%) | |
| **SAR ratio (95% CI)**** | 0.66 (0.21 – 2.09) | | | 1.39 (0.79 – 2.42) | | 1.25 (0.89 – 1.74) | | |
| **p-value**** | .48 | | | .25 | | .19 | | |
| **Secondary transmission of PCR-confirmed Influenza** | Intervention | Control | | Intervention | Control | Intervention | Control | |
| **Index case-patients (N) with PCR-confirmed influenza** | 2 | 3 | | 11 | 17 | 11 | 16 | |
| **Susceptible household members (N)** | 15 | 39 | | 77 | 105 | 87 | 106 | |
| **Secondary attack ratio** | 0/15 | 0/39 | | 6/77 (8%) | 1/105 (1%) | 11/85 (12.9%) | 9/106 (8.5%) | |
| **SAR ratio (95% CI)**** | Undefined | | | 8.33 (1.05 – 50.0) | | 1.49 (0.38 – 6.25) | | |
| **p-value**** | Undefined | | | .04 | | .55 | | |

* No illness in index case-patient household at the time of enrollment and index case-patient illness onset within 48 hours

**Confidence intervals and P-values generated using log binomial regression model with generalized estimating equations to estimate significance of ratio of secondary attack ratios in treatment arms.
