# Supplementary material for: Impact of Intensive Handwashing Promotion on Secondary Household Influenza-Like Illness in Rural Bangladesh: Findings from a Randomized Controlled Trial
Source: PLoS One. 2015 Jun 11;10(6):e0125200. doi: 10.1371/journal.pone.0125200 (PMC4465839; doi:10.1371/journal.pone.0125200)
Supplement: S1 Table — (DOCX) [file pone.0125200.s004.docx]

# Table S1. Phases of enrollment of index case-patients and susceptible contacts, Bangladesh Interruption of Secondary Transmission of Influenza Study (BISTIS), Kishoregonj, Bangladesh, 2009-2010

| **Phase** | Total | I | II | III |
| --- | --- | --- | --- | --- |
| **Dates** | July 19 2009 – October 30, 2010 | July 19 – Sep 3, 2009 | Sep 4 – Nov 9, 2009 | May 6 – October 30, 2010 |
| **Case definition of index case-patient** |  | Age-specific definition for influenza-like illness* | Age-specific definition for influenza-like illness | Age-specific definition for influenza-like illness, with symptom onset during 48 hours preceding enrollment |
| **Exclusion criteria for illness in household compound members** | *--* | Compounds excluded if any compound member(s) reported to have fever within 3 days before index case-patient enrollment | Compounds included irrespective of illness in primary and secondary household members; (illness occurring within 7 days prior to enrollment recorded) | Compounds excluded if any primary household member reported to have fever  (fever occurring within 48 hours prior to enrollment recorded) |
| **Distance from health facility** | *--* | Compounds located within 30 minutes travel time one-way from health facility | Compounds located within 2 hours travel time one-way from health facility | Compounds located within 2 hours travel time one-way from health facility |
| **Inclusion in model 1 (intent to treat)** | All | All | All | All |
| **Inclusion in model 2 (restricted to those meeting 2010 criteria)** | Only those compounds meeting 2010 criteria for illness in household compound members | Only those compounds meeting 2010 criteria for fever occurring within 48 hours of enrollment (In 2009, we did not collect information about illness in individual compound members, since the exclusion criteria were evaluated at the time of index case-patient recruitment) | Only those compounds meeting 2010 criteria for illness in household compound members and fever occurring within 48 hours of enrollment | All |

*Age-specific case definition for influenza-like illness: 1) fever for children < 5 years old; 2) fever with cough *or* sore throat for persons > 5 years old
