# Supplementary material for: Impact of Intensive Handwashing Promotion on Secondary Household Influenza-Like Illness in Rural Bangladesh: Findings from a Randomized Controlled Trial
Source: PLoS One. 2015 Jun 11;10(6):e0125200. doi: 10.1371/journal.pone.0125200 (PMC4465839; doi:10.1371/journal.pone.0125200)
Supplement: S2 Table — (DOCX) [file pone.0125200.s005.docx]

# Table S2. Randomization of household compounds, and exclusion of compounds and household members

|  | **Total** | | **Phase 1** | | **Phase 2** | | **Phase 3** | |
| --- | --- | --- | --- | --- | --- | --- | --- | --- |
|  | Intervention | Control | Intervention | Control | Intervention | Control | Intervention | Control |
| **Number of household compounds randomized** | 195 | 186 | 22 | 18 | 69 | 65 | 104 | 103 |
| **Number of household compounds remaining after withdrawals / exclusions after randomization* (explanation in footnote)** | 193 / 195  (99%) | 184/186  (99%) | 22/22  (100%) | 18/18  (100%) | 69/69  (100%) | 63/65  (97%) | 102/104 (98%) | 103/103  (100%) |
| **Number of household compound members enumerated in compounds after removal of withdrawn / excluded compounds (not including index case-patient)** | 1814 | 1607 | 184 | 178 | 617 | 479 | 1013 | 950 |
| **Number of compound members with fever at enrollment** | 78 / 1814  (4%) | 53/1607  (3%) | 0/184  (0%) | 0/178  (0%) | 66/617  (11%) | 46/479  (10%) | 12/1013  (1%) | 7/950  (1%) |
| **Number of susceptible compound members** | 1736 / 1814 (96%) | 1554/1607  (97%) | 184/184  (100%) | 178/178  (100%) | 551/617  (89%) | 433/479  (90%) | 1001/1013  (99%) | 943/950  (99%) |
| **Number of susceptible compound members without syndromic surveillance data** | 75/1736  (4%) | 56/1554  (4%) | 5/184  (3%) | 2/178  (1%) | 3/551  (1%) | 1/433  (0%) | 67/1001  (7%) | 53/943  (6%) |
| **Number of susceptible compound members with syndromic surveillance information for >1 days** | 1661/1736  (96%) | 1498/1554  (96%) | 179/184  (97%) | 176/178  (99%) | 548/551  (99%) | 432/433  (100%) | 934/1001  (93%) | 890/943  (94%) |
| **Number of susceptible members in index case-patient households** | 863/1661  (52%) | 727/1498  (49%) | 95/179  (53%) | 89/176  (51%) | 284/548  (52%) | 224/432  (52%) | 484/934  (52%) | 414/890  (47%) |
| **Number of susceptible members in other households** | 798/1661  (48%) | 771/1498  (51%) | 84/179  (47%) | 87/176  (49%) | 264/548  (48%) | 208/432  (48%) | 450/934  (48%) | 476/890  (53%) |
|  |  |  |  |  |  |  |  |  |
| **Number of index case-patients with PCR-confirmed influenza (% of total randomized)** | 24/193  (12%) | 36/184  (20%) | 2/22  (9%) | 3/18  (17%) | 11/69  (16%) | 17/63  (27%) | 11/102  (11%) | 16/103  (16%) |
| **Number of susceptible members in compounds of index case-patients with PCR-confirmed influenza** | 177/1661  (11%) | 250/1498  (17%) | 15/179  (8%) | 39/176  (22%) | 77/548  (14%) | 105/432  (24%) | 85/934  (9%) | 106/890  (12%) |

*excluded compounds that had travel time > 2 hours (# 1009), the compound withdrew from the study (# 3119), index case-patient was not present for > 3 days (# 2124 – removed because hh members not susceptible if index case-patient not present), and one compound was found to have fever among a member of the index case-patient household (4020 - intervention)
