# Supplementary material for: Impact of Intensive Handwashing Promotion on Secondary Household Influenza-Like Illness in Rural Bangladesh: Findings from a Randomized Controlled Trial
Source: PLoS One. 2015 Jun 11;10(6):e0125200. doi: 10.1371/journal.pone.0125200 (PMC4465839; doi:10.1371/journal.pone.0125200)
Supplement: S3 Table — (DOCX) [file pone.0125200.s006.docx]

# Table S3. Secondary attack ratios of influenza-like illness, and influenza, among household compound members of index case-patients, by study phase and in total, Kishoregonj, Bangladesh, 2009-2010

| **Model** | Phase 1 | | | Phase 2 | | Phase 3 | | |
| --- | --- | --- | --- | --- | --- | --- | --- | --- |
| **Secondary transmission of influenza-like illness** | Intervention | | Control | Intervention | Control | Intervention | | Control |
| **Index case-patients (N)** | 22 | | 18 | 69 | 63 | 102 | 103 | |
| **Susceptible household members (N)** | 179 | | 176 | 548 | 432 | 934 | 890 | |
| **Secondary attack ratio** | 4/179 (2.2%) | | 6/176 (3.4%) | 44/548 (8.0%) | 25/432 (5.8%) | 110/934 (11.8%) | 84/890 (9.4%) | |
| **SAR ratio (95% CI)**** | 0.66 (0.21 – 2.09) | | | 1.39 (0.79 – 2.42) | | 1.25 (0.89 – 1.74) | | |
| **p-value**** | .48 | | | .25 | | .19 | | |
| **Secondary transmission of PCR-confirmed Influenza** | Intervention | Control | | Intervention | Control | Intervention | Control | |
| **Index case-patients (N) with PCR-confirmed influenza** | 2 | 3 | | 11 | 17 | 11 | 16 | |
| **Susceptible household members (N)** | 15 | 39 | | 77 | 105 | 87 | 106 | |
| **Secondary attack ratio** | 0/15 | 0/39 | | 6/77 (8%) | 1/105 (1%) | 11/85 (12.9%) | 9/106 (8.5%) | |
| **SAR ratio (95% CI)**** | Undefined | | | 8.33 (1.05 – 50.0) | | 1.49 (0.38 – 6.25) | | |
| **p-value**** | Undefined | | | .04 | | .55 | | |

* No illness in index case-patient household at the time of enrollment and index case-patient illness onset within 48 hours

**Confidence intervals and P-values generated using log binomial regression model with generalized estimating equations to estimate significance of ratio of secondary attack ratios in treatment arms.
